# Global burden of benign prostatic hyperplasia, urinary tract infections, urolithiasis, bladder cancer, kidney cancer, and prostate cancer from 1990 to 2021

**DOI:** 10.1186/s40779-024-00569-w

**Published:** 2024-09-18

**Authors:** Hao Zi, Meng-Yang Liu, Li-Sha Luo, Qiao Huang, Peng-Cheng Luo, Hang-Hang Luan, Jiao Huang, Dan-Qi Wang, Yong-Bo Wang, Yuan-Yuan Zhang, Ren-Peng Yu, Yi-Tong Li, Hang Zheng, Tong-Zu Liu, Yu Fan, Xian-Tao Zeng

**Affiliations:** 1 Center for Evidence-Based and Translational Medicine, Zhongnan Hospital of Wuhan University, Wuhan, 430071 China; 2 Evidence-Based Medicine Center, Xiangyang No. 1 People’s Hospital, Hubei University of Medicine, Xiangyang, 441000 Hubei China; 3 Department of Urology, Wuhan Clinical Research Center of Tumors of the Urinary System and Male Genital Organs, Hubei Key Laboratory of Urinary System Diseases, Zhongnan Hospital of Wuhan University, Wuhan, 430071 China; 4 Department of Urology, Wuhan Third Hospital, Tongren Hospital of Wuhan University, Wuhan, 430060 China; 5 Department of Forensic Medicine, Zhongnan Hospital of Wuhan University, Wuhan, 430071 China; 6 School of Clinical Medicine, Hubei University of Arts and Science, Xiangyang, 441053 Hubei China; 7 Department of Urology, Institute of Urology, Peking University First Hospital, Peking University, The National Urological Cancer Center of China, Beijing, 100034 China

**Keywords:** Benign prostatic hyperplasia (BPH), Urinary tract infections (UTI), Urolithiasis, Bladder cancer, Kidney cancer, Prostate cancer, Disability-adjusted life-years (DALYs), Burden of disease

## Abstract

**Background:**

The burden of common urologic diseases, including benign prostatic hyperplasia (BPH), urinary tract infections (UTI), urolithiasis, bladder cancer, kidney cancer, and prostate cancer, varies both geographically and within specific regions. It is essential to conduct a comprehensive and precise assessment of the global burden of urologic diseases.

**Methods:**

We obtained data on incidence, prevalence, mortality, and disability-adjusted life-years (DALYs) for the aforementioned urologic diseases by age, sex, location, and year from the Global Burden of Disease (GBD) 2021. We analyzed the burden associated with urologic diseases based on socio-demographic index (SDI) and attributable risk factors. The trends in burden over time were assessed using estimated annual percentage changes (EAPC) along with a 95% confidence interval (CI).

**Results:**

In 2021, BPH and UTI were the leading causes of age-standardized incidence rate (ASIR) and age-standardized prevalence rate (ASPR), with rates of 5531.88 and 2782.59 per 100,000 persons, respectively. Prostate cancer was the leading cause of both age-standardized mortality rate (ASMR) and age-standardized DALYs rate (ASDR), with rates of 12.63 and 217.83 per 100,000 persons, respectively. From 1990 to 2021, there was an upward trend in ASIR, ASPR, ASMR, and ASDR for UTI, while urolithiasis showed a downward trend. The middle and low-middle SDI quintile levels exhibited higher incidence, prevalence, mortality, and DALYs related to UTI, urolithiasis, and BPH, while the high and high-middle SDI quintile levels showed higher rates for the three cancers. The burden of these six urologic diseases displayed diverse age and sex distribution patterns. In 2021, a high body mass index (BMI) contributed to 20.07% of kidney cancer deaths worldwide, while smoking accounted for 26.48% of bladder cancer deaths and 3.00% of prostate cancer deaths.

**Conclusions:**

The global burden of 6 urologic diseases presents a significant public health challenge. Urgent international collaboration is essential to advance the improvement of urologic disease management, encompassing the development of effective diagnostic screening tools and the implementation of high-quality prevention and treatment strategies.

**Supplementary Information:**

The online version contains supplementary material available at 10.1186/s40779-024-00569-w.

## Background

Benign prostatic hyperplasia (BPH), urinary tract infections (UTI), urolithiasis, bladder cancer, kidney cancer, and prostate cancer constitute the six prevalent urologic diseases [1]. These conditions not only impact patients’ quality of life but also reduce their life expectancy [2]. Managing these diseases requires substantial financial resources, often leading to a significant economic burden on patients, their families, and society as a whole [3]. As the global population grows older, the prevalence of urologic diseases will continue to rise. However, the burden of these diseases varies across regions and within them. It is crucial to conduct a comprehensive and accurate assessment of the global disease burden posed by urologic conditions.

In 2001, the Urologic Diseases in America project was launched to quantify the burden of urologic diseases in the United States, encompassing both their medical and financial impact [1]. In recent years, the Global Burden of Disease (GBD) studies have provided comprehensive data on the disease burden related to these diseases, serving as a foundation for health policy development [4, 5]. According to GBD 2019 findings, BPH exhibited the largest increase (110.56%) in disability-adjusted life-years (DALYs) from 1990 to 2019, followed by UTI (68.89%) and urolithiasis (16.95%) [5]. According to the Global Cancer Statistics 2022, prostate cancer ranks as the second most common cancer and the 5th leading cause of cancer mortality among men [6]. Additionally, bladder and kidney cancers are respectively ranked as the 9th and 14th most commonly diagnosed cancers worldwide. Previous studies have indicated that there is significant variation in the burden of urologic diseases across different regions [4–6]. However, comprehensive and up-to-date epidemiological data on urologic diseases remain limited.

The GBD 2021 presents an updated dataset on the global burden of urologic diseases across 204 countries and territories from 1990 to 2021 [7–9]. Compared to previous GBD studies, this cycle has integrated new available data sources and improved methodological approaches to offer the most current estimates. This study aims to present the incidence, prevalence, mortality, and DALYs of 6 urologic diseases as well as their evolving trends from 1990 to 2021. Our objective is to delineate the burden and trends of these diseases globally, regionally, and nationally, by socio-demographic index (SDI), age, sex, and their associated risk factors.

## Methods

### Data sources

The study used anonymized data from GBD 2021, a comprehensive database that measured the impact of 371 diseases, 88 risk factors, and injuries across 5 SDI and 204 countries and territories [7]. This information, accessible at https://vizhub.healthdata.org/gbd-results, was overseen by the Institute for Health Metrics and Evaluation at the University of Washington in the USA [7, 8]. The GBD 2021 findings were crucial for policymakers, public health professionals, and researchers as they facilitated the identification of health disparities within and between populations, monitoring changes over time, gauging health advancements, and shaping strategies to address post-COVID-19 health inequalities [7, 9].

In our study, we obtained the incidence, prevalence, mortality, and DALY estimates for 3 benign diseases involving BPH, UTI, and urolithiasis, and for 3 urologic cancers including bladder cancer, kidney cancer, and prostate cancer. The death burden related to BPH was reported as 0 in the GBD study due to its chronic and non-fatal nature. In GBD 2021, data sources for the three benign urologic diseases included hospital discharges and claims. Additionally, a systematic literature review was used for urolithiasis estimation. Estimates by age, sex, year, and country were produced using a Bayesian meta-regression model named DisMod-MR 2.1 [5, 7]. Data sources for malignant cancers comprised household surveys, censuses, vital statistics, and other health-related data sources. The Cause of Death Ensemble model was utilized to estimate cause-specific death rates [4, 8]. Disability weight (DW) represented the degree of health impairment or non-fatal disability, and years lived with disability (YLDs) were calculated as the total number of cases multiplied by the duration until remission or death, further multiplied by the DW [7, 10]. Years of life lost (YLLs) were determined by multiplying the number of deaths by predicted life expectancy based on age, sex, location, and year. DALYs were computed by summing YLLs and YLDs (The DALYs for BPH were equivalent to YLD) [10]. The methodology employed in GBD 2021 closely mirrors that of GBD 2019, with detailed descriptions available elsewhere [11, 12].

The SDI served as a comprehensive indicator that reflects a country’s overall development status, considering parameters such as income per capita, average years of schooling, and fertility rates in younger females [4, 7]. The SDI ranged from 0 to 1 and was categorized into high (0.805129–1), high-middle (0.689504–0.805129), middle (0.607679–0.689504), low-middle (0.454743–0.607679), and low (0–0.454743) levels [13].

### Definition of the six urologic diseases

In GBD 2021, all six urologic diseases were identified according to the International Classification of Diseases (ICD), Tenth Revision (ICD-10), and ICD-9. For 3 benign urologic diseases, BPH was defined as a non-cancerous overgrowth of prostatic tissue that often leads to symptoms such as urinary retention, bladder outlet obstruction, or urinary tract infection (coded as N40–N40.9, 600–600.91). UTI was defined as a kidney infection that can cause systemic symptoms like fever and weakness and may result in discomfort and difficulty with daily activities (coded as N10–N12.9, N13.6, N15, N15.1–N16.8, N30–N30.31, N30.8–N30.91, N34–N34.3, N39.0, 590–590.9, 595–595.81, 595.89–595.9, 597–597.9, 599.0), while Urolithiasis was defined as the formation of stone anywhere along the genitourinary tract (coded as N20–N23.0, 592–592.9, 594–594.9) [5, 7]. Three urologic cancers refer to malignancies affecting the organs of the genitourinary tract. In GBD 2021, the ICD-10 codes of these three urologic cancers were as follows: bladder cancer (C67–C67.9, 188–188.9, V10.51, V16.52, V76.3), kidney cancer (C64–C64.2, C64.9–C65.9, 189–189.1, 189.5–189.6, 209.24), and prostate cancer (C61–C61.9, 185–185.9, V10.46, V16.42, V76.44) [4, 5].

### Attributable risk factors

The conceptual framework of comparative risk assessment was developed to generate estimates of the burden attributable to risk factors, which were categorized into 4 hierarchical levels. This study specifically focused on risks at level 4 [11]. In the GBD 2021 analysis, the process of estimating risk factor burden involved a series of 7 interconnected methodological procedures. Initially, the evaluation entailed estimating effect size by calculating the relative risk (RR) associated with specific health outcomes resulting from exposure to identified risk factors. Subsequently, data on exposure were collected and their distribution across risk factors was assessed using Bayesian statistical methodologies. Following this, Theoretical Minimum Risk Exposure Levels (TMRELs) were established based on cumulative epidemiological findings. Then, population-attributable fractions (PAFs) were computed for each risk-outcome pairing to serve as a metric for assessing potential health improvements if risk exposure was reduced to the TMREL. Age-specific exposure values (SEVs) were then calculated to reflect the prevalence of exposure while adjusting for age-specific risk factors. Additionally, mediation factors were estimated to address any potential overestimations in PAFs. Finally, attributable burden estimates were determined by multiplying PAF values with the deaths or DALYs for each specific combination of age group, sex, geographical location, and calendar year [14].

In our study, smoking was found to be a risk factor for prostate cancer. Additionally, both smoking and high fasting plasma glucose (FPG) were identified as risk factors for bladder cancer. Furthermore, kidney cancer was found to be associated with smoking, high body mass index (BMI), and occupational exposure to trichloroethylene. High BMI was defined as a BMI greater than 20–23 kg/m^2^ for individuals aged 20 years and above, while high FPG was categorized as any level above 4.9–5.3 mmol/L. Current smokers were defined as individuals who currently use any tobacco product that is smoked on a daily or occasional basis, while former smokers were those who had ceased using all smoked tobacco products for at least 6 months. Occupational exposure to trichloroethylene was defined as the percentage of individuals aged 15 and older who have had previous occupational exposure to trichloroethylene at varying levels of intensity [11].

### Statistical analysis

Between 1990 and 2021, an analysis was conducted to assess the impact of burden from 6 different urologic diseases. This analysis presented all estimates as counts, age-specific rates, and age-standardized rates (ASRs) per 100,000 persons for disease burden. In the GBD 2021 study, the ASR was calculated using the following formula: ${\text{ASR}} = \frac{{\mathop \sum \nolimits_{i = 1}^{A} a_{i} w_{i} }}{{\mathop \sum \nolimits_{i = 1}^{A} w_{i} }} \times 100,000$, where a_*i*_ represents the *i*th age group and the number of populations (or weight *w*_*i*_) in the same age group *i* of the reference standard population [4]. The GBD standard population structure was utilized as a reference population to calculate ASRs [7]. All estimates for disease burden were presented as mean values with 95% uncertainty intervals (UIs), and the attributable burden of 3 types of urologic cancers to various risk factors was expressed as a percentage of total deaths (%) and DALYs (%) along with 95% UI. The 95% UIs were displayed by the 25th and 95th ordered values across all 1000 draws. Additionally, we examined the correlations between the health measures of these six urologic diseases and the SDI. Trends over time were assessed using the estimated annual percentage changes (EAPC) with 95% confidence intervals (CIs), which was achieved through a linear regression model based on the equation *Y* = *α* + *βx* + *ε*, where *Y* was the natural logarithm of ASR, *X* denoted the calendar year, and *ε* indicated the error term. The EAPC was then determined as 100 × [exp(*β*) − 1] [5, 15]. Statistical analysis and visual representation were performed using R software (version 4.3.2) and Microsoft Excel (version 2019), with the R packages utilized including “ggplot2”, “RColorBrewer”, “patchwork”, and “ggrepel”.

## Results

### Global incidence, prevalence, mortality and DALYs

**Fig. 1:**
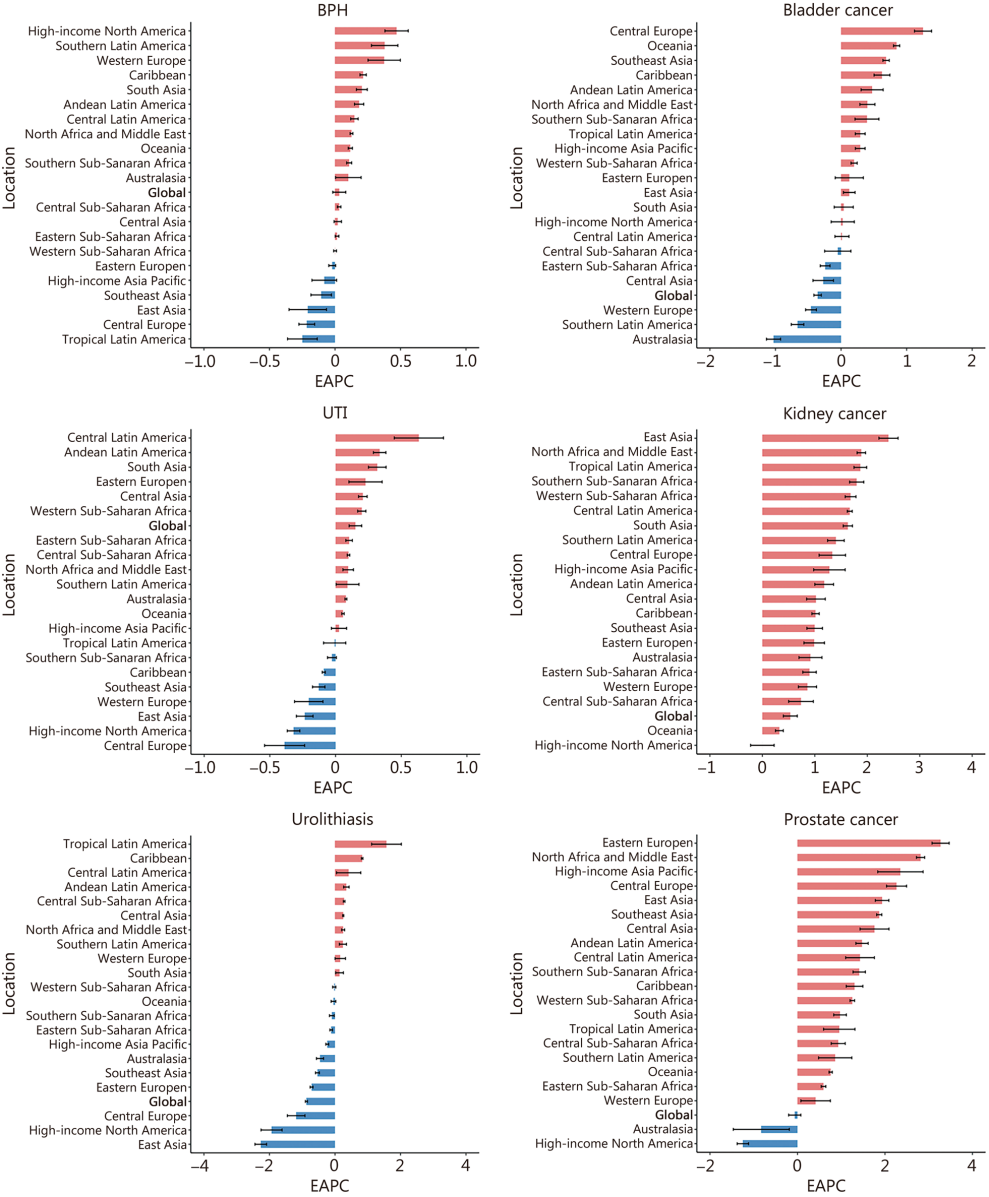
The EAPC of ASIR for 6 urologic diseases in global and 21 regions. ASIR age-standardized incidence rate, EAPC estimated annual percentage change, BPH benign prostatic hyperplasia, UTI urinary tract infections

In 2021, the global incidence of BPH was 137.88 × 10^5^ (95% UI 109.08–170.15), UTI was 4491.02 × 10^5^ (95% UI 4008.94–4998.43), and urolithiasis was 1059.84 × 10^5^ (95% UI 883.49–1286.45). The age-standardized incidence rate (ASIR) for UTI was the highest at 5531.88 per 100,000 persons (95% UI 4965.44–6161.01) (Table 1). From 1990 to 2021, the ASIR for UTI exhibited an upward trend with EAPC of 0.15 (95% CI 0.10–0.20), while the ASIR for urolithiasis showed a decreasing trend with EAPC of − 0.87 (95% CI − 0.91 to − 0.84), and the ASIR for BPH demonstrated no statistically significant trend (Table 1, Fig. 1; Additional file 1: Table S1). In 2021, the global incidence of bladder cancer, kidney cancer, and prostate cancer was estimated at 5.40 × 10^5^ (95% UI 4.95–5.83), 3.88 × 10^5^ (95% UI 3.65–4.07), and 13.24 × 10^5^ (95% UI 12.17–14.00), respectively, with the ASIR of prostate cancer (34.05 per 100,000 persons, 95% UI 31.27–36.00) higher than that of kidney cancer (4.52 per 100,000 persons, 95% UI 4.26–4.75) and bladder cancers (6.35 per 100,000 persons, 95% UI 5.80–6.85) (Table 1). From 1990 to 2021, the ASIR of kidney cancer showed a significant increasing trend with EAPC of 0.53 (95% CI 0.40–0.66), while bladder cancer and prostate cancer showed decreasing trends with EAPC of − 0.36 (95% CI − 0.41 to − 0.30) and − 0.06 (95% CI − 0.20 to 0.08) (Table 1, Fig. 1; Additional file 1: Table S2).

**Table 1:** Global incidence, prevalence, mortality, and DALYs of 6 urologic diseases from 1990 to 2021

Year	BPH	UTI	Urolithiasis	Bladder cancer	Kidney cancer	Prostate cancer
1990						
Incidence (× 10^5^, 95% UI)	64.06 (50.00–79.95)	2698.15 (2417.07–2992.89)	731.16 (604.47–900.45)	2.60 (2.43–2.72)	1.60 (1.55–1.64)	5.06 (4.81–5.25)
Prevalence (× 10^5^, 95% UI)	507.06 (387.36–656.93)	51.37 (46.11–56.88)	27.63 (22.67–33.75)	13.29 (12.49–13.84)	7.52 (7.28–7.71)	35.96 (34.45–37.05)
Mortality (× 10^5^, 95% UI)	–	1.03 (0.94–1.16)	0.11 (0.07–0.13)	1.23 (1.13–1.30)	0.77 (0.75–0.80)	2.12 (1.94 − 2.24)
DALYs (× 10^5^, 95% UI)	10.11 (6.06–15.56)	33.93 (30.33–37.19)	4.95 (3.79–6.10)	27.33 (24.72–28.94)	22.92 (21.9–23.86)	41.47 (37.54–44.02)
ASIR (1/100,000, 95% UI)	335.00 (262.28–414.71)	5294.50 (4784.14–5869.53)	1602.48 (1315.54–1983.36)	6.90 (6.46–7.23)	3.89 (3.75–3.99)	32.64 (30.86–33.86)
ASPR (1/100,000, 95% UI)	2899.84 (2240.05–3682.58)	100.96 (91.2–111.55)	60.72 (49.44–74.41)	33.41 (31.49–34.81)	17.26 (16.76–17.67)	218.33 (208.48–225.67)
ASMR (1/100,000, 95% UI)	–	2.84 (2.58–3.20)	0.29 (0.20–0.35)	3.51 (3.23–3.70)	1.99 (1.91–2.06)	16.35 (15.02–17.28)
ASDR (1/100,000, 95% UI)	57.48 (34.56–87.77)	74.98 (67.72–81.95)	11.38 (8.72–13.99)	71.05 (64.72–75.17)	53.02 (50.96–54.93)	275.30 (251.66–292.14)
2021						
Incidence (× 10^5^, 95% UI)	137.88 (109.08–170.15)	4491.02 (4008.94–4998.43)	1059.84 (883.49–1286.45)	5.40 (4.95–5.83)	3.88 (3.65–4.07)	13.24 (12.17–14.00)
Prevalence (× 10^5^, 95% UI)	1125.02 (881.32–1426.34)	85.61 (76.74–94.97)	40.21 (33.64–48.16)	30.26 (28.23–32.24)	19.61 (18.62–20.52)	103.88 (97.06–109.04)
Mortality (× 10^5^, 95% UI)	–	3.00 (2.68–3.24)	0.18 (0.14–0.21)	2.22 (2.01–2.42)	1.61 (1.50–1.69)	4.32 (3.82–4.64)
DALYs (× 10^5^)	22.36 (13.46–34.03)	68.48 (61.75–73.69)	6.93 (5.68–8.50)	43.97 (40.64–48.14)	40.16 (38.07–42.47)	81.42 (71.77–88.09)
ASIR (1/100,000, 95% UI)	326.12 (258.88–400.32)	5531.88 (4965.44–6161.01)	1242.84 (1034.94–1506.99)	6.35 (5.80–6.85)	4.52 (4.26–4.75)	34.05 (31.27–36.00)
ASPR (1/100,000, 95% UI)	2782.59 (2191.58–3508.04)	105.35 (94.44–116.98)	47.10 (39.41–56.31)	34.91 (32.54–37.19)	22.70 (21.54–23.76)	260.05 (243.39–272.68)
ASMR (1/100,000, 95% UI)	–	3.71 (3.31–4.01)	0.21 (0.17–0.25)	2.68 (2.42–2.93)	1.91 (1.78–2.01)	12.63 (11.16–13.55)
ASDR (1/100,000, 95% UI)	55.12 (33.21–83.48)	83.74 (75.54–90.22)	8.15 (6.68–9.99)	51.58 (47.56–56.42)	47.33 (44.76–50.07)	217.83 (192.65–235.53)
1990–2021						
ASIR (EAPC, 95% CI)	0.03 (− 0.02–0.08)	0.15 (0.10–0.20)	− 0.87 (− 0.91 to − 0.84)	− 0.36 (− 0.41 to − 0.30)	0.53 (0.40–0.66)	− 0.06 (− 0.20–0.08)
ASPR (EAPC, 95% CI)	− 0.01 (− 0.06–0.04)	0.15 (0.10–0.19)	− 0.87 (− 0.90 to − 0.84)	0.10 (0.01–0.19)	0.98 (0.80–1.16)	0.42 (0.27–0.58)
ASMR (EAPC, 95% CI)	–	1.02 (0.95–1.10)	− 1.02 (− 1.24 to − 0.80)	− 0.98 (− 1.03 to − 0.94)	− 0.14 (− 0.21 to − 0.07)	− 1.05 (− 1.14 to − 0.95)
ASDR (EAPC, 95% CI)	0.00 (− 0.05–0.05)	0.42 (0.35–0.49)	− 1.15 (− 1.28 to − 1.02)	− 1.19 (− 1.24 to − 1.13)	− 0.37 (− 0.43 to − 0.30)	− 0.96 (− 1.05 to − 0.88)

*BPH* benign prostatic hyperplasia, *UTI* urinary tract infections*, DALYs* disability-adjusted life-years, *ASIR* age-standardized incidence rate, *ASPR* age-standardized prevalence rate, *ASMR* age-standardized mortality rate, *ASDR* age-standardized DALYs rate, *EAPC* estimated annual percentage change, *CI* confidence interval, *UI* uncertainty intervals

Globally, in 2021, there were 1125.02 × 10^5^ (95% UI 881.32–1426.34) prevalence of BPH, with an age-standardized prevalence rate (ASPR) of 2782.59 (95% UI 2191.58–3508.04) per 100,000 persons, surpassing the rates for UTI and urolithiasis (Table 1). From 1990 to 2021, the ASPR for UTI exhibited a significant increasing trend (EAPC = 0.15, 95% CI 0.10–0.19), while urolithiasis showed a decreasing trend (EAPC =  − 0.87, 95% CI − 0.90 to − 0.84) (Table 1; Additional file 1: Table S1 and Fig S1). In the same year, the global prevalence and ASPR of prostate cancer exceeded those of bladder cancer and kidney cancer; and from 1990 to 2021, the ASPRs for all three urologic cancers demonstrated significant increasing trends, with kidney cancer showing the highest increase (EAPC = 0.98, 95% CI 0.80–1.16) (Table 1; Additional file 1: Table S2 and Fig. S1).

In 2021, an estimated 3.00 × 10^5^ (95% UI 2.68–3.24) mortality was attributed to UTI, and approximately 0.18 × 10^5^ (95% UI 0.14–0.21) cases died from urolithiasis globally. The age-standardized mortality rate (ASMR) for UTI exhibited a positive trend (EAPC = 1.02, 95% CI 0.95–1.10), while the ASMR for urolithiasis showed a declining pattern (EAPC =  − 1.02, 95% CI − 1.24 to − 0.80) over recent decades (Table 1; Additional file 1: Table S1 and Fig. S2). In 2021, prostate cancer (12.63, 95% UI 11.16–13.55) exhibited a significantly higher ASMR compared to the other two types of cancers. However, from 1990 to 2021, all three cancers showed declining trends in ASMR with EAPC of − 0.98 (95% CI − 1.03 to − 0.94) for bladder cancer, − 0.14 (95% CI − 0.21 to − 0.07) for kidney cancer and − 1.05 (95% CI − 1.14 to − 0.95) for prostate cancer (Table 1; Additional file 1: Table S2 and Fig. S2).

Globally, UTI accounted for the highest number of DALYs among the three urologic benign diseases in 2021 (68.48 × 10^5^, 95% UI 61.75–73.69), and it also exhibited the highest positive EAPC of the age-standardized DALYs rate (ASDR, 0.42, 95% CI 0.35–0.49) (Table 1; Additional file 1: Table S1 and Fig. S3). Among cancers, prostate cancer had the highest ASDR value (217.83, 95% UI 192.65–235.53), and all three cancers demonstrated decreasing ASDR trends from 1990 to 2021, with bladder cancer showing the most significant EAPC (− 1.19, 95% CI − 1.24 to − 1.13) (Table 1; Additional file 1: Table S2 and Fig. S3).

### Regional incidence, prevalence, mortality, and DALYs

When analyzed by geographic regions, East Asia and South Asia exhibited the highest number of incidence, prevalence, mortality, and DALYs of BPH, UTI, and urolithiasis in 2021. Specifically, East Asia had the highest incidence of BPH (33.96 × 10^5^, 95% UI 26.04–42.46), while South Asia recorded the highest incidence of UTI (1666.39 × 10^5^, 95% UI 1466.27–1873.36) and urolithiasis (228.13 × 10^5^, 95% UI 185.92–281.74) (Table 2). Moreover, South Asia also had the highest prevalence of BPH (261.16 × 10^5^, 95% UI 192.77–339.67), UTI (31.69 × 10^5^, 95% UI 27.91–35.66), and urolithiasis (8.64 × 10^5^, 95% UI 7.01–10.65), as well as the highest death cases for UTI (0.80 × 10^5^, 95% UI 0.66–0.90) and urolithiasis (0.04 × 10^5^, 95% UI 0.02–0.06) (Additional file 1: Tables S3, S4). Furthermore, South Asia exhibited the highest DALYs of BPH (5.17 × 10^5^, 95% UI 3.11–7.94), UTI (23.97 × 10^5^, 95% UI 20.00–26.87) and urolithiasis (1.66 × 10^5^, 95% UI 1.14–2.31) (Additional file 1: Table S5). In the year 2021, Eastern Europe exhibited the highest ASIR for BPH at a value of 661.12 per 100,000 persons (95% UI 527.06–792.25), as well as the highest ASPR at 6262.23 per 100,000 persons (95% UI 4821.08–7834.28), and ASDR at 123.56 per 100,000 persons (95% UI 75.40–185.08). Conversely, Tropical Latin America reported the highest ASIR for UTI (13,021.38 per 100,000 persons, 95% UI 11,715.27–14,448.99), ASPR (248.35 per 100,000 persons, 95% UI 223.35–276.03), ASMR (11.74 per 100,000 persons, 95% UI 10.16–12.66) and ASDR (217.07 per 100,000 persons, 95% UI 198.1–229.94). Eastern Europe also exhibited the highest ASIR for urolithiasis (3557.08 per 100,000 persons, 95% UI 2986.04–4230.09), ASPR (134.55 per 100,000 persons, 95% UI 113.03–158.80), ASMR (0.62 per 100,000 persons, 95% UI 0.56–0.72) and ASDR (22.82 per 100,000 persons, 95% UI 19.42–28.00) (Table 2; Additional file 1: Tables S3–S5 and Figs. S4–S7). Between 1990 and 2021, the burden of these three urologic benign diseases exhibited increasing trends in over half of GBD regions. High-income North America and Southern Latin America experienced the most significant increases in BPH burden. The greatest rise in UTI burden was observed in Central and Southern Latin America, while Tropical Latin America saw the highest increase in urolithiasis burden (Fig. 1; Additional file 1: Figs. S1–S3).

**Table 2:** Regional incidence and ASIR of the 6 urologic diseases in 2021

Location	BPH		UTI		Urolithiasis		Bladder cancer		Kidney cancer		Prostate cancer	
	Incidence (× 10^5^, 95% UI)	ASIR (1/100,000, 95% UI)	Incidence (× 10^5^, 95% UI)	ASIR (1/100,000, 95% UI)	Incidence (× 10^5^, 95% UI)	ASIR (1/100,000, 95% UI)	Incidence (× 10^5^, 95% UI)	ASIR (1/100,000, 95% UI)	Incidence (× 10^5^, 95% UI)	ASIR (1/100,000, 95% UI)	Incidence (× 10^5^, 95% UI)	ASIR (1/100,000, 95% UI)
Global	137.88 (109.08–170.15)	326.12 (258.88–400.32)	4491.02 (4008.94–4998.43)	5531.88 (4965.44–6161.01)	1059.84 (883.49–1286.45)	1242.84 (1034.94–1506.99)	5.40 (4.95–5.83)	6.35 (5.8–6.85)	3.88 (3.65–4.07)	4.52 (4.26–4.75)	13.24 (12.17–14)	34.05 (31.27–36.00)
High SDI	20.84 (17.17–25.23)	223.24 (185.43–269.31)	742.24 (677.46–814.86)	6100.68 (5533.03–6705.59)	174.08 (143.87–211.89)	1169.61 (975.93–1415.84)	2.29 (2.08–2.41)	10.60 (9.78–11.14)	1.73 (1.62–1.81)	8.97 (8.46–9.33)	6.95 (6.47–7.28)	70.92 (66.29–74.22)
High-middle SDI	32.51 (25.58–40.12)	341.28 (270.68–417.58)	557.28 (504.43–614.80)	4023.84 (3634.33–4430.86)	252.22 (210.68–302.63)	1443.36 (1209.76–1734.02)	1.64 (1.49–1.82)	8.24 (7.53–9.15)	1.19 (1.11–1.27)	6.27 (5.84–6.69)	2.68 (2.38–2.92)	30.36 (26.96–33.04)
Middle SDI	49.25 (38.69–61.10)	365.42 (287.67–451.19)	1333.13 (1192.54–1482.76)	5148.53 (4616.29–5699.6)	359.87 (299.34–438.93)	1280.51 (1069.55–1551.67)	0.97 (0.84–1.14)	3.72 (3.24–4.35)	0.67 (0.61–0.73)	2.46 (2.25–2.69)	2.28 (1.95–2.59)	19.43 (16.51–22.07)
Low-middle SDI	27.05 (20.94–34.05)	377.12 (292.19–470.61)	1360.44 (1200.04–1527.46)	7233.73 (6407.8–8105.63)	203.44 (168.22–249.49)	1131.51 (939.52–1386.06)	0.36 (0.32–0.48)	2.64 (2.32–3.48)	0.20 (0.19–0.22)	1.33 (1.22–1.45)	0.96 (0.8–1.11)	15.90 (13.29–18.51)
Low SDI	8.10 (6.23–10.24)	316.96 (244.62–400.47)	494.78 (432.09–557.27)	5247.32 (4622.31–5932.82)	69.40 (55.80–86.11)	837.27 (688.09–1034.42)	0.14 (0.12–0.16)	2.95 (2.56–3.42)	0.08 (0.06–0.10)	1.15 (0.87–1.4)	0.36 (0.23–0.44)	18.14 (11.84–22.34)
Andean Latin America	0.96 (0.73–1.23)	338.46 (255.41–429.17)	83.21 (70.93–94.73)	12,329.29 (10,545.34–13,972.53)	11.49 (9.61–13.98)	1728.54 (1442.18–2095.12)	0.01 (0.01–0.02)	2.49 (1.99–3.1)	0.02 (0.02–0.03)	3.56 (2.85–4.41)	0.11 (0.08–0.15)	42.16 (30.78–57.66)
Australasia	0.59 (0.44–0.77)	238.76 (181.61–306.07)	29.30 (26.14–33.11)	9059.46 (7987.57–10,236.89)	5.11 (4.15–6.32)	1279.62 (1039.29–1590.43)	0.04 (0.04–0.04)	7.38 (6.69–7.99)	0.04 (0.04–0.05)	8.55 (7.57–9.55)	0.25 (0.21–0.3)	97.88 (82.7–115.15)
Caribbean	0.93 (0.71–1.18)	361.16 (277.09–456.08)	38.38 (34.24–43.87)	7847.49 (6982.63–8893.56)	6.78 (5.61–8.30)	1289.99 (1071.71–1568.69)	0.03 (0.02–0.03)	5.16 (4.5–5.72)	0.02 (0.01–0.02)	3.06 (2.69–3.44)	0.23 (0.2–0.27)	93.77 (80.45–108.03)
Central Asia	1.26 (0.96–1.62)	329.97 (254.99–415.88)	63.69 (55.24–73.7)	6556.46 (5724.47–7604.7)	17.93 (15.16–21.46)	1869.97 (1590.87–2214.08)	0.03 (0.03–0.03)	3.62 (3.19–4.03)	0.03 (0.03–0.04)	3.90 (3.39–4.38)	0.05 (0.04–0.05)	14.90 (13.51–16.48)
Central Europe	3.71 (3.02–4.42)	376.63 (310.49–445.07)	54.71 (49.97–59.73)	4502.79 (4166.66–4911.73)	15.77 (13.09–18.95)	1039.50 (867.21–1240.02)	0.28 (0.25–0.30)	12.20 (11.17–13.11)	0.18 (0.17–0.20)	8.63 (7.91–9.37)	0.48 (0.43–0.53)	48.90 (44.4–53.65)
Central Latin America	6.22 (4.95–7.65)	522.07 (416.84–639.17)	199.81 (182.10–220.42)	7906.47 (7228.09–8718.27)	31.42 (26.04–37.89)	1177.50 (977.50–1417.98)	0.06 (0.05–0.07)	2.42 (2.14–2.68)	0.11 (0.1–0.12)	4.27 (3.82–4.76)	0.72 (0.62–0.83)	65.04 (56.1–74.53)
Central Sub-Saharan Africa	0.66 (0.49–0.88)	246.34 (187.34–322.96)	42.39 (36.95–48.43)	3717.58 (3249.29–4174.09)	6.46 (5.21–8.01)	667.25 (543.24–830.03)	0.02 (0.01–0.02)	3.36 (2.61–4.28)	0.01 (0–0.01)	0.95 (0.58–1.48)	0.05 (0.03–0.06)	28.26 (17.17–39.13)
East Asia	33.96 (26.04–42.46)	303.48 (236.84–380.16)	217.31 (191.75–243.82)	1188.92 (1062.4–1335.12)	201.05 (164.81–248.23)	978.49 (812.71–1190.62)	1.11 (0.88–1.42)	5.20 (4.17–6.61)	0.70 (0.58–0.84)	3.41 (2.83–4.05)	0.97 (0.71–1.30)	9.90 (7.39–13.06)
Eastern Europe	9.59 (7.52–11.75)	661.12 (527.06–792.25)	187.00 (168.25–210.13)	9043.32 (8203.32–10,051.3)	102.01 (86.17–120.05)	3557.08 (2986.04–4230.09)	0.23 (0.21–0.25)	6.52 (5.88–7.11)	0.33 (0.30–0.35)	9.61 (8.82–10.4)	0.76 (0.68–0.83)	56.28 (50.56–61.65)
Eastern Sub-Saharan Africa	2.29 (1.75–2.94)	275.75 (209.05–352.62)	123.00 (106.64–140.20)	3548.27 (3116.81–4004.23)	21.92 (17.65–27.23)	708.93 (579.56–885.91)	0.05 (0.04–0.06)	3.41 (2.83–4.19)	0.04 (0.03–0.05)	1.72 (1.20–2.18)	0.15 (0.09–0.19)	22.55 (14.67–28.24)
High-income Asia Pacific	2.53 (1.94–3.31)	138.23 (106.16–179.65)	112.66 (100.90–123.86)	6206.60 (5676.77–6828.78)	36.98 (30.03–45.47)	1381.01 (1128.98–1699.03)	0.35 (0.30–0.38)	6.95 (6.24–7.51)	0.19 (0.17–0.21)	4.58 (4.20–4.86)	0.66 (0.58–0.73)	28.74 (25.29–31.7)
High-income North America	6.47 (5.44–7.58)	216.18 (183.87–251.08)	296.05 (271.49–324.57)	6556.42 (6030.20–7161.38)	47.88 (40.48–56.53)	965.22 (824.31–1129.38)	0.93 (0.86–0.98)	13.98 (12.96–14.61)	0.71 (0.66–0.74)	11.54 (10.82–11.96)	3.16 (2.98–3.30)	101.92 (95.89–106.4)
North Africa and Middle East	6.04 (4.63–7.76)	250.22 (191.45–319.89)	258.15 (226.27–292.11)	4033.36 (3553.69–4548.7)	53.04 (42.16–68.01)	851.40 (686.65–1075.18)	0.36 (0.30–0.43)	8.22 (6.95–9.95)	0.14 (0.13–0.16)	2.82 (2.51–3.16)	0.56 (0.39–0.68)	27.39 (19.27–33.39)
Oceania	0.17 (0.13–0.21)	437.75 (333.86–548.82)	2.44 (2.08–2.85)	2014.02 (1725.21–2346.91)	1.06 (0.86–1.34)	934.04 (762.55–1162.92)	0.00 (0. 00–0.00)	2.13 (1.37–2.86)	0.00 (0. 00–0.00)	0.64 (0.41–0.92)	0.01 (0.00–0.01)	24.56 (15.95–34.27)
South Asia	31.26 (24.08–39.42)	412.90 (319.88–516.15)	1666.39 (1466.27–1873.36)	8997.24 (7932.64–10,080.58)	228.13 (185.92–281.74)	1246.52 (1023.87–1534.23)	0.31 (0.27–0.37)	2.19 (1.91–2.68)	0.16 (0.14–0.17)	1.03 (0.94–1.13)	0.48 (0.39–0.66)	7.60 (6.26–10.42)
Southeast Asia	15.40 (12.11–19.21)	467.42 (367.61–577.87)	215.07 (189.75–242.12)	2876.54 (2546.21–3220.06)	123.13 (102.42–147.09)	1628.43 (1364.12–1936.29)	0.16 (0.14–0.20)	2.55 (2.17–3.19)	0.14 (0.12–0.15)	1.96 (1.74–2.20)	0.43 (0.28–0.53)	16.72 (10.97–20.71)
Southern Latin America	0.65 (0.49–0.83)	164.39 (124.74–211.33)	52.26 (45.84–58.79)	7160.84 (6263.39–8082.91)	15.04 (12.58–18.21)	1965.12 (1646.29–2386.85)	0.05 (0.05–0.06)	5.85 (5.41–6.26)	0.11 (0.10–0.12)	13.44 (12.27–14.73)	0.17 (0.14–0.19)	44.06 (37.83–50.95)
Southern Sub-Saharan Africa	0.89 (0.68–1.16)	342.67 (265.2–433.79)	39.21 (33.66–45.25)	4806.93 (4149.53–5523.07)	5.34 (4.28–6.69)	680.49 (547.08–849.18)	0.02 (0.02–0.03)	4.28 (3.82–4.76)	0.01 (0.01–0.01)	2.13 (1.92–2.33)	0.11 (0.08–0.13)	55.12 (40.52–64.65)
Tropical Latin America	2.69 (2.22–3.33)	222.87 (183.01–273.81)	313.99 (282.61–348.70)	13,021.38 (11,715.27–14,448.99)	26.84 (22.63–31.94)	1032.28 (875.09–1223.82)	0.10 (0.09–0.11)	3.97 (3.66–4.20)	0.09 (0.08–0.09)	3.41 (3.19–3.59)	0.45 (0.42–0.48)	41.32 (38.45–43.85)
Western Europe	9.18 (7.38–11.21)	241.55 (197.00–291.87)	320.81 (285.71–360.95)	7291.66 (6397.07–8293.60)	81.39 (67.13–99.55)	1366.17 (1132.05–1664.34)	1.22 (1.11–1.30)	12.59 (11.57–13.40)	0.82 (0.76–0.87)	9.74 (9.11–10.36)	3.15 (2.86–3.39)	73.05 (66.40–78.43)
Western Sub-Saharan Africa	2.42 (1.85–3.10)	250.90 (191.94–320.18)	175.20 (153.18–198.01)	4307.05 (3786.14–4865.97)	21.07 (16.85–26.27)	605.63 (492.84–755.04)	0.04 (0.04–0.05)	2.36 (1.99–2.84)	0.03 (0.02–0.04)	0.98 (0.78–1.20)	0.30 (0.16–0.40)	41.46 (21.70–55.16)

*BPH* benign prostatic hyperplasia, *UTI* urinary tract infections, *ASIR* age-standardized incidence rate, *UI* uncertainty interval

In 2021, Western Europe exhibited the highest incidence (1.22 × 10^5^, 95% UI 1.11–1.30), prevalence (7.09 × 10^5^, 95% UI 6.56–7.53) and mortality (0.48 × 10^5^, 95% UI 0.42–0.51) of bladder cancer, while East Asia had the highest DALYs (9.70 × 10^5^, 95% UI 7.75–12.24). For kidney cancer, Western Europe recorded the highest incidence (0.82 × 10^5^, 95% UI 0.76–0.87) and mortality (0.33 × 10^5^, 95% UI 0.29–0.35), whereas high-income North America had the highest prevalence (4.12 × 10^5^, 95% UI 3.89–4.27) and East Asia had the highest DALYs (7.00 × 10^5^, 95% UI 5.77–8.36). Regarding prostate cancer, high-income North America showed the highest incidence (3.16 × 10^5^, 95% UI 2.98–3.30) and prevalence (28.29 × 10^5^, 95% UI 26.98–29.47), while Western Europe had the highest mortality (0.86 × 10^5^, 95% UI 0.76–0.93) and DALYs (14.43 × 10^5^, 95% UI 12.92–15.69) (Table 2; Additional file 1: Tables S3–S5). It was noteworthy that from 1990 to 2021, kidney cancer demonstrated increasing trends in ASIR and ASPR across all GBD regions, and mortality, as well as DALY rates for kidney cancer continued to rise over time in most regions. Notably, East Asia exhibited rapid increases in both ASIR and ASPR for kidney cancer, while Southern Sub-Saharan Africa showed significant rises for both ASMR as well as ASDR (Additional file 1: Figs. S4–S7). From 1990 to 2021, there was a notable rise in ASIR and ASPR for bladder cancer across most regions, particularly pronounced in Central Europe. Conversely, a decline was observed for ASMR and ASDR, with the largest reductions occurring in East Asia. The patterns of prostate cancer closely resembled those of bladder cancer, with the most rapid increases in ASIR and ASPR observed in East Europe, North Africa, and the Middle East. Conversely, the most significant decreases in ASMR and ASDR were seen in Australasia (Fig. 1; Additional file 1: Figs. S1–S3).

### National incidence, prevalence, mortality, and DALYs

In 2021, Lithuania exhibited the highest burden of BPH, with ASIR, ASPR, and ASDR reaching 691.36, 6719.37, and 132.83 per 100,000 persons respectively (Additional file 1: Table S6). Austria demonstrated the most rapid increases in ASIR, ASPR, and ASDR for BPH from 1990 to 2021, with EAPCs were 0.72 (95% CI 0.61–0.83), 0.57 (95% CI 0.45–0.69) and 0.58 (95% CI 0.46–0.70) respectively (Additional file 1: Tables S7–S10). Ecuador exhibited the highest ASIR and ASPR of UTI, with ASIR at 15,136.70 and ASPR at 288.01, while Barbados had the highest ASMR at 12.96 and Turkmenistan had the highest ASDR at 298.01 for UTI, respectively (Additional file 1: Table S6). From 1990 to 2021, Mexico showed the fastest increases in ASIR and ASPR of UTI (EAPC = 1.16, 95% CI 0.79–1.52; EAPC = 1.16, 95% CI 0.80–1.53, respectively), while Argentina demonstrated the fastest increases in ASMR and ASDR (EAPC = 7.57, 95% CI 6.52–8.62; EAPC = 6.69, 95% CI 5.82–7.58, respectively) (Additional file 1: Tables S7–S10). The highest ASIR and ASPR of urolithiasis were observed in Ukraine (ASIR: 3766.92, ASPR: 142.62), while the highest ASMR and ASDR were recorded in Kazakhstan (ASMR: 1.29, ASDR: 33.34) in 2021 (Additional file 1: Table S6). From 1990 to 2021, the most rapid increases in ASIR and ASPR occurred in Trinidad and Tobago (EAPC = 2.85, 95% CI 2.61–3.10; EAPC = 2.86, 95% CI 2.61–3.11, respectively), whereas the fastest increases in ASMR and ASDR were observed in Kuwait with an EAPC of 22.50 (95% CI 19.37–25.71), as well as Brazil with an EAPC of 2.91 (95% CI 2.66–3.17) (Additional file 1: Tables S7–S10).

In 2021, Lebanon recorded the highest ASIR of bladder cancer at 21.66 and the highest ASPR at 130.22 among three urologic cancers. Mali had the highest ASMR for bladder cancer at 8.99, while Malawi had the highest ASDR at 179.92 (Additional file 1: Table S6). The Republic of Cabo Verde exhibited the most rapid increases in ASIR, ASPR, ASMR and ASDR for bladder cancer, with EAPC of 6.19 (95% CI 4.76–7.65), 6.39 (95% CI 5.16–7.62), 5.40 (95% CI 3.94–6.88) and 5.51 (95% CI 4.05–6.99), respectively (Additional file 1: Tables S7–S10). In 2021, Argentina exhibited the highest ASIR at 15.60 and ASPR at 89.01 for kidney cancer, while Uruguay recorded the highest ASMR at 6.47 and ASDR at 170.16 (Additional file 1: Table S6). Furthermore, from 1990 to 2021, the most rapid increases in kidney cancer burden were observed in the Republic of Cabo Verde (Additional file 1: Tables S7–S10). The highest ASIR and ASPR for prostate cancer in 2021 were observed in Bermuda (ASIR: 196.12, ASPR: 1527.67), while the highest ASMR and ASDR were in Grenada (ASMR: 93.90, ASDR: 1542.79) (Additional file 1: Table S6). From 1990 to 2021, the most rapid increases in ASIR and ASPR for prostate cancer occurred in the Republic of Korea, and the fastest increases in ASMR and ASDR were seen in Georgia (Additional file 1: Tables S7–S10).

### Burden of 6 urologic diseases by SDI

**Fig. 2:**
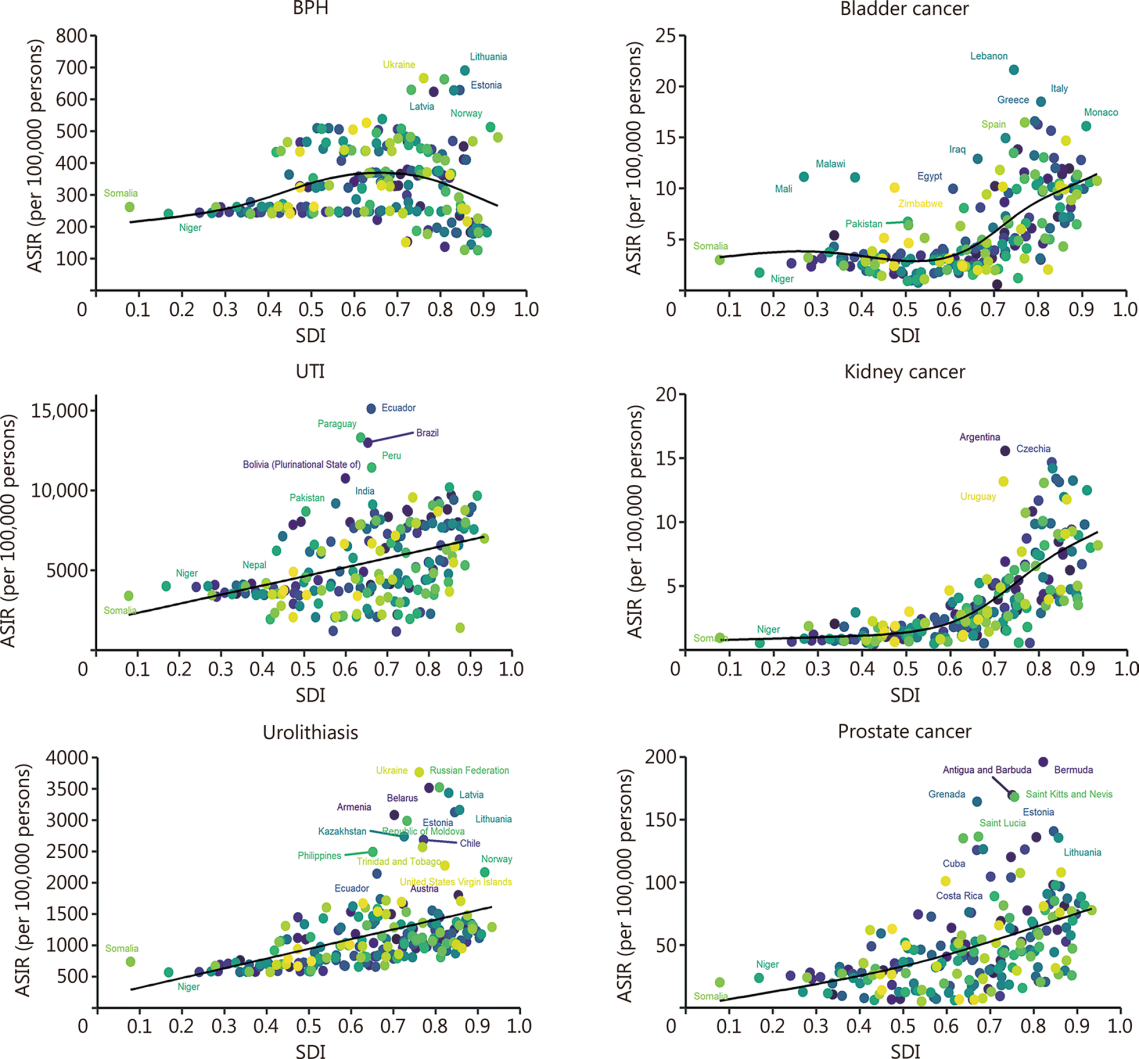
ASIR of 6 urologic diseases for 204 countries and territories by SDI. ASIR age-standardized incidence rate, SDI sociodemographic index, BPH benign prostatic hyperplasia, UTI urinary tract infections

In 2021, the middle and low-middle SDI quintile levels exhibited higher incidence and prevalence, mortality, and DALYs of BPH, UTI, and urolithiasis. Conversely, the high and high-middle SDI quintile levels showed higher rates for bladder, kidney, and prostate cancer (Table 2; Additional file 1: Tables S3–S5). Nationally, similar associations were found between ASIR and ASPR for 6 urologic diseases with SDI across 204 countries and territories in 2021. Specifically, generally positive relationships were observed between ASIR and ASPR for 3 urologic cancers with SDI. This trend was also evident in the associations between ASIR and ASPR of UTI as well as urolithiasis with SDI. Furthermore, there were positive correlations between ASMR and ASDR of kidney cancer with SDI, while the ASDR of UTI showed slight negative correlations with SDI. No significant associations among ASMR, ASDR, and SDI were found for BPH, urolithiasis, bladder cancer, and prostate cancer (Fig. 2; Additional file 1: Figs. S8–S10).

### Burden of 6 urologic diseases by age and sex

**Fig. 3:**
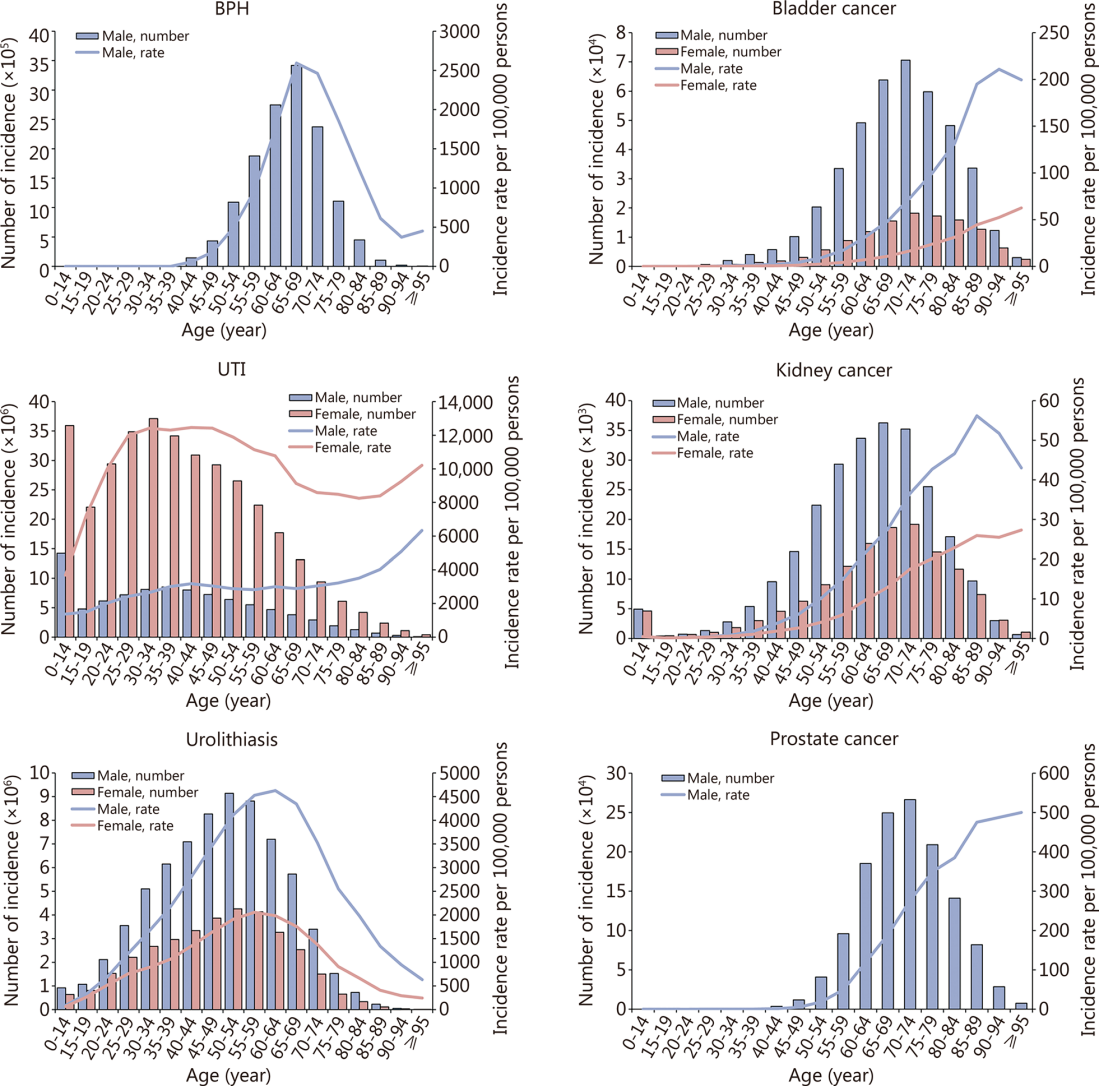
Global incidence of 6 urologic diseases by age and sex in 2021. BPH benign prostatic hyperplasia, UTI urinary tract infections

The age distribution patterns of 6 urologic diseases were observed in 2021. Specifically, the number of incidence and prevalence as well as DALYs of BPH were predominantly concentrated in the 65–69 age group, with the highest incidence rates also occurring in the 65–69 age group, while the rates of prevalence and DALY were highest in the 75–79 age group (Fig. 3; Additional file 1: Figs. S11–S13). Both incidence and prevalence cases of UTI mainly focused on groups aged 25–34 and 0–14 years, while the deaths occurred primarily in those aged over 80 years, and DALYs were observed across both the youngest and oldest age groups. The incidence and prevalence rates of UTI were highest among individuals aged between 25 and 34 years, whereas mortality and DALY rates increased with advancing age. Urolithiasis incidence and prevalence, along with their corresponding rates, mainly affected individuals aged between 50 and 65 years, while deaths and DALYs as well as their respective rates were most prevalent among those aged over 80 years (Fig. 3; Additional file 1: Additional file 1: Figs. S11–S13). The incidence, prevalence, and DALYs of bladder, kidney, and prostate cancer were predominantly concentrated in the 70–74, 65–69, and 70–74 age groups. Conversely, deaths occurred most frequently in the 80–84, 70–74, and 80–84 age groups. Additionally, the age-specific rates for all three urologic cancers increased with age, reaching their highest values in older age groups. Notably observed was a significant decrease in the incidence and prevalence rates of kidney cancer among the elderly population (Fig. 3; Additional file 1: Figs. S11–S13).

In 2021, males constituted the primary demographic affected by urolithiasis with significantly higher incidence, prevalence, mortality, and DALYs, as well as their corresponding rates across all age groups compared to females. Conversely, females represented a more susceptible population for UTI, exhibiting substantially greater incidence and prevalence as well as higher rates compared to males. However, the mortality and DALYs associated with UTI showed a balanced between men and women (Fig. 3; Additional file 1: Figs. S11–S13). Moreover, there were distinct sex-specific patterns in the cancer burden, with higher incidence, prevalence, mortality, and DALYs of bladder and kidney cancers observed in men compared to women across all age groups globally in 2021 (Fig. 3; Additional file 1: Figs. S11–S13).

### Attributable burden of urologic cancers caused by risk factors

In 2021, smoking was responsible for 26.48% (95% UI 22.78–30.40) of global deaths and 28.15% (95% UI 24.42–31.95) of DALYs related to bladder cancer, with the highest burden observed in East Asia (Table 3). Moreover, Additionally, high FPG contributed to 7.91% (95% UI − 0.99 to 18.02) of global deaths and 7.36% (95% UI − 0.93 to 16.74) of DALYs from bladder cancer in the year (Table 3), particularly impacting regions with high SDI quintiles such as high-income North America (Additional file 1: Tables S11–S12). For kidney cancer, 20.07% (95% UI 7.96–31.73) of global deaths and 19.46% (95% UI 7.76–31.03) of DALYs were attributable to high BMI, while smoking contributed to 10.06% (95% UI 6.05–14.35) of mortality and 9.53% (95% UI 5.92–13.42) of DALYs, and occupational exposure to trichloroethylene caused only a small proportion of mortality at 0.05% (95% UI 0.01–0.09) and DALYs at 0.06% (95% UI 0.01–0.12) (Table 3). In 2021, there was an increase in the prevalence of high BMI and occupational exposure to trichloroethylene compared to 1990, while the prevalence of smoking decreased (Table 3). High-BMI and smoking were associated with a higher burden of attributable mortality and DALYs for kidney cancer in regions with high SDI and high-middle SDI quintiles. Specifically, the highest burden attributable to high BMI was observed in high-income North America, while East Asia had the highest burden attributable to smoking. In contrast, occupational exposure to trichloroethylene contributed to the highest mortality and DALYs in middle SDI and low-middle SDI quintiles, such as Andean Latin America and Central Latin America (Additional file 1: Tables S11–S12). In terms of prostate cancer, smoking emerged as its primary risk factor, contributing to 3.00% (95% UI 1.42–4.92) of global deaths and 3.46% (95% UI 1.65–5.56) of DALYs in 2021 (Table 3), with the highest attributable burden observed in high-middle SDI quintiles (Additional file 1: Tables S11–S12).

**Table 3:** Percentage of urologic cancer deaths and DALYs attributed to risk factors in 1990 and 2021

Cancer	1990			2021		
	Leading risk	Percentage of death (95% UI)	Percentage of DALYs (95% UI)	Leading risk	Percentage of death (95% UI)	Percentage of DALYs (95% UI)
Bladder cancer	Smoking	33.38 (29.28–37.42)	34.58 (30.53–38.67)	Smoking	26.48 (22.78–30.40)	28.15 (24.42–31.95)
	High FPG	4.95 (− 0.64 to 11.18)	4.50 (− 0.58 to 10.15)	High FPG	7.91 (− 0.99 to 18.02)	7.36 (− 0.93 to 16.74)
Kidney cancer	High BMI	15.65 (6.12–25.23)	13.88 (5.46–22.22)	High BMI	20.07 (7.96–31.73)	19.46 (7.76–31.03)
	Smoking	12.49 (7.86–17.39)	10.97 (6.95–15.22)	Smoking	10.06 (6.05–14.35)	9.53 (5.92–13.42)
	Occupational exposure to trichloroethylene	0.03 (0.01–0.06)	0.04 (0.01–0.07)	Occupational exposure to trichloroethylene	0.05 (0.01–0.09)	0.06 (0.01–0.12)
Prostate cancer	Smoking	4.69 (2.23–7.42)	5.27 (2.50–8.30)	Smoking	3.00 (1.42–4.92)	3.46 (1.65–5.56)

*BMI* body mass index, *DALYs* disability-adjusted life-years, *FPG* fasting plasma glucose, *UI* uncertainty interval

## Discussion

The global community has become increasingly aware of the significant threat to global health posed by urologic diseases. The prevalence of these diseases has been on the rise, presenting growing challenges to public health policies and healthcare systems worldwide. Their impact is widespread, necessitating immediate attention and action. This study offers comprehensive estimates of urologic diseases at the global, regional, and national levels by SDI, age, and sex group from 1990 to 2021. In 2021, among various urologic diseases, UTI exhibited the highest ASIR, while BPH recorded the highest ASPR. Additionally, prostate cancer emerged as having the most significant ASMR and ASDR globally. From 1990 to 2021, BPH, UTI, and urolithiasis showed increasing trends in over half of GBD regions. In terms of bladder cancer, kidney cancer, and prostate cancer, the majority of regions demonstrated rising ASIR and ASPR, along with declining ASMR and ASDR. At the national level, areas with middle and low-middle SDI quintile levels experienced higher incidences and prevalences as well as mortality and DALYs related to BPH, UTI, and urolithiasis. Conversely, high and high-middle SDI quintile levels had higher incidences of bladder, kidney, and prostate cancers in 2021. Accurate data on the epidemiology and impact of urologic diseases is essential for the fair allocation of limited resources at global, regional, and national levels. This study builds upon previous research efforts to provide a comprehensive assessment of the global burden of urologic diseases, laying a solid foundation for healthcare services planning and intervention programs.

This study demonstrated that the SDI was correlated with the incidence, prevalence, mortality, and DALYs of 6 urologic diseases. Positive associations were found between ASIR and ASPR of these diseases and SDI across 204 countries and territories in 2021. Previous studies indicated that countries in higher SDI quintiles exhibited higher incidences of urologic diseases [4, 5, 16]. Generally, countries with higher SDI levels tend to have more advanced healthcare systems and increased patient engagement. Furthermore, transitioning from a traditional diet to an industrialized one, heightened exposure to environmental pollutants, and lifestyle changes such as reduced physical activity, can all contribute to an elevated risk of chronic diseases [17–19]. The incidence of BPH and urologic cancers tends to increase with advancing age. In countries with a higher SDI, there is a tendency for a larger proportion of the elderly population, which may subsequently lead to an increased incidence of BPH and urologic cancers. The SDI exhibited positive correlations with the ASMR and ASDR of kidney cancer, whereas a slight negative correlation was observed between the ASDR of UTI and SDI. Furthermore, other disease burden analyses of kidney cancer indicated that regions with the highest SDI levels continue to bear higher cancer burdens compared to regions with the lowest SDI levels [16, 20, 21]. Therefore, regions with higher SDIs should prioritize efforts in reducing kidney cancer-specific risk factors and improving early detection and treatment.

The burden of 6 urologic diseases exhibited distinct age and sex distribution patterns. The incidence and prevalence of BPH and urologic cancers increased with age, particularly after the age of 40. The rise in urologic conditions with advancing age is often attributed to the accumulation of genetic mutations, hormonal changes, and lifestyle factors over time. Moreover, researchers project that life expectancy will continue to rise from 73.6 years in 2022 to 78.2 years in 2050 [22, 23]. Global demographic and aging trends will exacerbate the burden of these diseases. This trend has significant implications for societies and economies worldwide, necessitating adaptive policies and strategies to ensure the well-being of older populations. The burden of BPH and urologic cancers is predominantly concentrated in males. However, this sex gap is narrowing in UTI and urolithiasis. Previous evidence suggests that smoking, alcohol consumption, and obesity are risk factors for urologic cancers, with men being more susceptible due to prolonged exposure to these risk factors [24]. Additionally, industries with higher male participation may be more likely to expose individuals to occupational hazards associated with urologic cancers [25]. Meanwhile, previous studies have indicated that men have a threefold greater risk of developing bladder cancer than women, but women diagnosed with bladder cancer are often at a more advanced stage than men [26–29]. Current evidence suggests that men are more frequently diagnosed with urologic cancers than women. Sex steroid hormones and their receptors could play a role in the development and progression of urologic cancers [30–32]. Therefore, sex-specific preventive measures as well as therapeutic approaches are crucial for promoting gender health equality.

In 2021, high BMI, smoking, and high FPG emerged as primary drivers behind the burden of urologic cancers. Notably for kidney cancer, approximately 20.07% of global deaths and 19.46% of DALYs could be attributed to high BMI (Additional file 1: Tables S11, S12). Research from a European Perspective Investigation into Cancer and Nutrition revealed a significant association between high BMI and a relative risk for kidney cancer at 2.25 [33]. Furthermore, obesity and weight gain exhibit positive correlations with increased risks for kidney cancer. Conversely, regular physical activity has been linked to reduced risks [34–36]. Compelling evidence underscores that maintaining a healthy weight through regular exercise along with a balanced diet not only aids in preventing excessive weight gain but also mitigates risks associated with kidney cancer [37]. Consequently, it is imperative that preventive strategies prioritize these modifiable risk factors. Moreover, there have been observed declining trends from 1990 to 2021 regarding global deaths as well as DALYs attributed to smoking-related contributions toward urological cancer burdens. Heightening awareness about these specific risk factors related to urologic cancers within both developed nations as well as those still developing may serve to reduce disparities while simultaneously improving prognoses.

Focusing on 6 urologic diseases, we examine the disease burden of these conditions with global prospects for prevention and control. The GBD for these six urologic diseases reveals 2 distinct patterns, one related to cancer and the other to non-malignant diseases. The ASIR and ASPR of bladder cancer, kidney cancer, and prostate cancer have shown upward trends, while the ASMR and ASDR have exhibited downward trends in most countries and territories, particularly in those with higher SDI quintile levels. This pattern demonstrates the practical impact of different SDI regions on disease prevention and management strategies. Long-term cancer control outcomes in Northern America, and Oceania, as well as Northern and Western Europe, underscore universal access to screening and improved treatment outcomes as key factors in reducing the burden of disease [38–40]. Epidemiological evidence regarding the impact of prostate cancer in Germany indicates a rise in incidence alongside a decline in mortality over recent decades, which is consistent with our research [41, 42]. This pattern can be attributed partly to an increased adoption of prostate-specific antigen screening. While this widespread use has significantly enhanced early detection and subsequently lowered mortality [43, 44], it has also contributed to overtreatment and compounded the economic strain associated with this disease. A long-term population-based study conducted in Germany revealed that advancements in early detection and treatment have improved 10-year survival rates for those with prostate cancer; however, this shift has primarily placed greater financial responsibility on payers and providers [45]. Similarly, cross-section imaging screening for kidney cancer as well as endoscopic resection for bladder cancer have diminished mortality within highly developed healthcare environments [46, 47]. Furthermore, it is important to note that the observed increase in incidence may not necessarily indicate a genuine rise in malignant diseases, but could be attributed (at least partially) to the growing prevalence of screening and early detection. Our findings revealed an increase in ASIR and a decrease in ASMR and ASDR for prostate cancer in Germany during the study period, illustrating the combined impact of enhanced outcomes from cancer screening and treatment. Therefore, conducting long-term follow-up studies on cancer survival might play a more significant role in disease prevention and control. Non-malignant disease pattern shows higher ASIR and ASPR compared to cancer patterns in the middle and low-middle SDI quintile levels. Population growth, dietary changes, and increases in antimicrobial resistance in these areas may partly account for this trend [48–50]. Conversely, unequal distribution of medical resources in underdeveloped areas could exacerbate this phenomenon [5]. Moreover, there has been a global upward trend in ASIR, ASPR, ASMR, and ASDR for UTI from 1990 to 2021, contrasting with a downward trend for urolithiasis. Given the pressing issue of global bacterial antimicrobial resistance in UTIs, it is imperative for the international community to strengthen surveillance and promote the rational use of antibiotics [51]. Additionally, the utilization of ureteroscopy for urolithiasis could be expanded to more developing countries due to its lower recurrence rates and costs [48]. BPH is considered a non-fatal disease with stable ASIR, ASPR, and ASDR trends during the study period. However, BPH remains a prevalent diagnosis among aging males with increasing prevalence [52]. With the accelerated pace of global aging, early identification of men at high risk of BPH is critical for timely intervention to delay disease onset and progression.

This study presents the global burden of 6 common urologic diseases based on updated estimates from the GBD 2021, but several limitations remain. Firstly, there are general limitations shared by all GBD studies, which have been described elsewhere [7–9, 14]. The scarcity of reliable epidemiological data in low-income and middle-income countries, as well as diagnostic and other biases in the original research, also affect the estimated results of the GBD. Secondly, the COVID-19 pandemic has introduced significant uncertainty into the estimation of mortality rates for all diseases, particularly in areas most severely affected by the pandemic. Thirdly, relying solely on GBD data is insufficient for disentangling the intricate impacts of sex on health outcomes. Fourth, due to the limitation of the urologic disease definition provided by GBD, the burden of diseases may be underestimated. Fifth, there are other influential factors in shaping the burden of disease, such as the prevalence of prostate-specific antigen-based prostate cancer screening, which could introduce bias into estimates because accurate information for each country was not available. Finally, GBD conducts risk factor analyses based on literature review and may not encompass all risk factors for each disease.

## Conclusions

The burden of urologic diseases represents a significant public health concern on a global scale. In national efforts to reduce this burden, policymakers must take into account the increasing number of individuals affected by urologic diseases and the impact of aging populations. It is essential for worldwide collaboration to make progress in improving the health status of those with urologic diseases, including the development of effective diagnostic screening tools and the implementation of high-quality prevention and treatment strategies [53].

## Supplementary Information

**Additional** **file 1: Table S1** EAPC of the ASIR, ASPR, ASMR and ASDR of 3 urologic cancers in global and 21 regions. **Table S2** EAPC of the ASIR, ASPR, ASMR and ASDR of 3 benign urologic diseases in global and 21 regions. **Table S3** Regional prevalence cases and ASPR of the 6 urologic diseases in 2021. **Table S4** Regional death cases and ASMR of the 6 urologic diseases in 2021. **Table S5** Regional DALYs and ASDR of the 6 urologic diseases in 2021. **Table S6** Age-standardized incidence, prevalence, mortality, and DALY rates of the 6 urologic diseases among the top 3 and bottom 3 countries in 2021. **Table S7** EAPC of ASIR for the 6 urologic diseases in 204 countries and territories from 1990 to 2021. **Table S8** EAPC of ASPR for the 6 urologic diseases in 204 countries and territories from 1990 to 2021. **Table S9** EAPC of ASMR for the 6 urologic diseases in 204 countries and territories from 1990 to 2021. **Table S10** EAPC of ASDR for the 6 urologic diseases in 204 countries and territories from 1990 to 2021. **Table S11** Percentage of urologic cancers deaths attributable to risk factors in 2021. **Table S12** Percentage of urologic cancers DALYs attributable to risk factors in 2021. **Fig. S1** The EAPC of ASPR for 6 urologic diseases in global and 21 regions. **Fig. S2** The EAPC of ASMR for 5 urologic diseases in global and 21 regions. **Fig. S3** The EAPC of ASDR for 6 urologic diseases in global and 21 regions. **Fig. S4** The ASIR for 6 urologic diseases in 21 regions from 1990 to 2021. **Fig. S5** The ASPR for 6 urologic diseases in 21 regions from 1990 to 2021. **Fig. S6** The ASMR for 5 urologic diseases in 21 regions from 1990 to 2021. **Fig. S7** The ASDR for 6 urologic diseases in 21 regions from 1990 to 2021.** Fig. S8** ASPR of 6 urologic diseases for 204 countries and territories by SDI. **Fig. S9** ASMR of 5 urologic diseases for 204 countries and territories by SDI. **Fig. S10** ASDR of 6 urologic diseases for 204 countries and territories by SDI. **Fig. S11** Global prevalence of 6 urologic diseases by age and sex in 2021. **Fig. S12** Global deaths of 5 urologic diseases by age and sex in 2021. **Fig. S13** Global DALYs of 6 urologic diseases by age and sex in 2021.

## Data Availability

The datasets generated during the current study are available in the Global Health Data Exchange query tool (https://vizhub.healthdata.org/gbd-results).

## Abbreviations

ASDR: Age-standardized DALYs rate
ASIR: Age-standardized incidence rate
ASMR: Age-standardized mortality rate
ASPR: Age-standardized prevalence rate
ASR: Age-standardized rate
BMI: Body mass index
BPH: Benign prostatic hyperplasia
CI: Confidence interval
DALYs: Disability-adjusted life-years
EAPC: Estimated annual percentage changes
FPG: Fasting plasma glucose
GBD: Global Burden of Disease
ICD: International Classification of Diseases
PAFs: Population attributable fractions
SDI: Socio-demographic index
UI: Uncertainty interval
UTI: Urinary tract infections
YLD: Years lived with disability

## References

[1] Miller DC, Saigal CS, Litwin MS. The demographic burden of urologic diseases in America. Urol Clin North Am. 2009;36(1):11–27. 19038632 10.1016/j.ucl.2008.08.004 PMC2614213

[2] Okeke CJ, Jeje EA, Obi AO, Ojewola RW, Ogbobe UU. The burden of urologic diseases in a tertiary hospital in South-Eastern Nigeria: a three-year review. J West Afr Coll Surg. 2023;13(4):78–82. 38449546 10.4103/jwas.jwas_59_23 PMC10914105

[3] Choi SY, Yoon CG. Urologic diseases in Korean military population: a 6-year epidemiological review of medical records. J Korean Med Sci. 2017;32(1):135–42. 27914143 10.3346/jkms.2017.32.1.135 PMC5143286

[4] Zi H, He SH, Leng XY, Xu XF, Huang Q, Weng H, et al. Global, regional, and national burden of kidney, bladder, and prostate cancers and their attributable risk factors, 1990–2019. Mil Med Res. 2021;8(1):60. 34819142 10.1186/s40779-021-00354-z PMC8611255

[5] Zhu C, Wang DQ, Zi H, Huang Q, Gu JM, Li LY, et al. Epidemiological trends of urinary tract infections, urolithiasis and benign prostatic hyperplasia in 203 countries and territories from 1990 to 2019. Mil Med Res. 2021;8(1):64. 34879880 10.1186/s40779-021-00359-8 PMC8656041

[6] Bray F, Laversanne M, Sung H, Ferlay J, Siegel RL, Soerjomataram I, et al. Global cancer statistics 2022: GLOBOCAN estimates of incidence and mortality worldwide for 36 cancers in 185 countries. CA Cancer J Clin. 2024;74(3):229–63. 38572751 10.3322/caac.21834

[7] GBD 2021 Diseases and Injuries Collaborators. Global incidence, prevalence, years lived with disability (YLDs), disability-adjusted life-years (DALYs), and healthy life expectancy (HALE) for 371 diseases and injuries in 204 countries and territories and 811 subnational locations, 1990-2021: a systematic analysis for the Global Burden of Disease Study 2021. Lancet. 2024;403(10440):2133–61. 38642570 10.1016/S0140-6736(24)00757-8 PMC11122111

[8] GBD 2021 Causes of Death Collaborators. Global burden of 288 causes of death and life expectancy decomposition in 204 countries and territories and 811 subnational locations, 1990–2021: a systematic analysis for the Global Burden of Disease Study 2021. Lancet. 2024;403(10440):2100–32. 38582094 10.1016/S0140-6736(24)00367-2 PMC11126520

[9] GBD 2021 Demographics Collaborators. Global age-sex-specific mortality, life expectancy, and population estimates in 204 countries and territories and 811 subnational locations, 1950-2021, and the impact of the COVID-19 pandemic: a comprehensive demographic analysis for the Global Burden of Disease Study 2021. Lancet. 2024;403(10440):1989–2056. 38484753 10.1016/S0140-6736(24)00476-8 PMC11126395

[10] Kim YE, Jung YS, Ock M, Yoon SJ. DALY estimation approaches: understanding and using the incidence-based approach and the prevalence-based approach. J Prev Med Public Health. 2022;55(1):10–8. 35135044 10.3961/jpmph.21.597 PMC8841194

[11] GBD 2019 Risk Factors Collaborators. Global burden of 87 risk factors in 204 countries and territories, 1990–2019: a systematic analysis for the Global Burden of Disease Study 2019. Lancet. 2020;396(10258):1223–49. 33069327 10.1016/S0140-6736(20)30752-2 PMC7566194

[12] GBD 2019 Diseases and Injuries Collaborators. Global burden of 369 diseases and injuries in 204 countries and territories, 1990–2019: a systematic analysis for the Global Burden of Disease Study 2019. Lancet. 2020;396(10258):1204–22. 33069326 10.1016/S0140-6736(20)30925-9 PMC7567026

[13] Liu Y, Wen H, Bai J, Sun J, Chen J, Yu C. Disease burden and prediction analysis of tracheal, bronchus, and lung cancer attributable to residential radon, solid fuels, and particulate matter pollution under different sociodemographic transitions from 1990 to 2030. Chest. 2024;165(2):446–60. 37806491 10.1016/j.chest.2023.09.028

[14] GBD 2021 Risk Factors Collaborators. Global burden and strength of evidence for 88 risk factors in 204 countries and 811 subnational locations, 1990–2021: a systematic analysis for the Global Burden of Disease Study 2021. Lancet. 2024;403(10440):2162–203. 38762324 10.1016/S0140-6736(24)00933-4 PMC11120204

[15] Hankey BF, Ries LA, Kosary CL, Feuer EJ, Merrill RM, Clegg LX, et al. Partitioning linear trends in age-adjusted rates. Cancer Causes Control. 2000;11(1):31–5. 10680727 10.1023/a:1008953201688

[16] Huang Q, Yang J, Liu GX, Zi H, Tang SD, Jia HC, et al. Changes in disease burden and global inequalities in bladder, kidney and prostate cancers from 1990 to 2019: a comparative analysis based on the global burden of disease study 2019. BMC Public Health. 2024;24(1):891. 38528465 10.1186/s12889-024-18353-9 PMC10962085

[17] GBD 2021 Diabetes Collaborators. Global, regional, and national burden of diabetes from 1990 to 2021, with projections of prevalence to 2050: a systematic analysis for the Global Burden of Disease Study 2021. Lancet. 2023;402(10397):203–34. 37356446 10.1016/S0140-6736(23)01301-6 PMC10364581

[18] Dai X, Gakidou E, Lopez AD. Evolution of the global smoking epidemic over the past half century: strengthening the evidence base for policy action. Tob Control. 2022;31(2):129–37. 35241576 10.1136/tobaccocontrol-2021-056535

[19] GBD 2015 Obesity Collaborators, Afshin A, Forouzanfar MH, Reitsma MB, Sur P, Estep K, Lee A, et al. Health effects of overweight and obesity in 195 countries over 25 years. N Engl J Med. 2017;377(1):13–27. 28604169 10.1056/NEJMoa1614362 PMC5477817

[20] Khadembashiri MM, Ghasemi E, Khadembashiri MA, Azadnajafabad S, Moghaddam SS, Eslami M, et al. The global, regional, and national burden and quality of care index of kidney cancer; a global burden of disease systematic analysis 1990–2019. Int J Qual Health Care. 2024;36(1):mzad113. 38183265 10.1093/intqhc/mzad113

[21] Cirillo L, Innocenti S, Becherucci F. Global epidemiology of kidney cancer. Nephrol Dial Transpl. 2024;39(6):920–8. 10.1093/ndt/gfae036 38341277

[22] GBD 2021 Forecasting Collaborators. Burden of disease scenarios for 204 countries and territories, 2022–2050: a forecasting analysis for the Global Burden of Disease Study 2021. Lancet. 2024;403(10440):2204–56. 38762325 10.1016/S0140-6736(24)00685-8 PMC11121021

[23] Wise J. Global life expectancy to increase by almost five years by 2050, study predicts. BMJ. 2024;385:q1126. 38768972 10.1136/bmj.q1126

[24] Cepeda-Benito A, Doogan NJ, Redner R, Roberts ME, Kurti AN, Villanti AC, et al. Trend differences in men and women in rural and urban U.S. settings. Prev Med. 2018;117:69–75. 29627511 10.1016/j.ypmed.2018.04.008 PMC6173654

[25] Lucca I, Klatte T, Fajkovic H, de Martino M, Shariat SF. Gender differences in incidence and outcomes of urothelial and kidney cancer. Nat Rev Urol. 2015;12(10):585–92. 26436686 10.1038/nrurol.2015.232

[26] Horstmann M, Witthuhn R, Falk M, Stenzl A. Gender-specific differences in bladder cancer: a retrospective analysis. Gend Med. 2008;5(4):385–94. 19108811 10.1016/j.genm.2008.11.002

[27] Kluth LA, Fajkovic H, Xylinas E, Crivelli JJ, Passoni N, Rouprêt M, et al. Female gender is associated with higher risk of disease recurrence in patients with primary T1 high-grade urothelial carcinoma of the bladder. World J Urol. 2013;31(5):1029–36. 23196773 10.1007/s00345-012-0996-9

[28] Soave A, Dahlem R, Hansen J, Weisbach L, Minner S, Engel O, et al. Gender-specific outcomes of bladder cancer patients: a stage-specific analysis in a contemporary, homogenous radical cystectomy cohort. Eur J Surg Oncol. 2015;41(3):368–77. 24674298 10.1016/j.ejso.2014.03.003

[29] Messer JC, Shariat SF, Dinney CP, Novara G, Fradet Y, Kassouf W, et al. Female gender is associated with a worse survival after radical cystectomy for urothelial carcinoma of the bladder: a competing risk analysis. Urology. 2014;83(4):863–7. 24485993 10.1016/j.urology.2013.10.060

[30] Boorjian S, Ugras S, Mongan NP, Gudas LJ, You X, Tickoo SK, et al. Androgen receptor expression is inversely correlated with pathologic tumor stage in bladder cancer. Urology. 2004;64(2):383–8. 15302512 10.1016/j.urology.2004.03.025

[31] Sottnik JL, Vanderlinden L, Joshi M, Chauca-Diaz A, Owens C, Hansel DE, et al. Androgen receptor regulates CD44 expression in bladder cancer. Cancer Res. 2021;81(11):2833–46. 33687952 10.1158/0008-5472.CAN-20-3095 PMC8782536

[32] Shortliffe LM, Ye Y, Behr B, Wang B. Testosterone changes bladder and kidney structure in juvenile male rats. J Urol. 2014;191(6):1913–9. 24518779 10.1016/j.juro.2014.01.012

[33] Pischon T, Lahmann PH, Boeing H, Tjønneland A, Halkjaer J, Overvad K, et al. Body size and risk of renal cell carcinoma in the European Prospective Investigation into Cancer and Nutrition (EPIC). Int J Cancer. 2006;118(3):728–38. 16094628 10.1002/ijc.21398

[34] Callahan CL, Hofmann JN, Corley DA, Zhao WK, Shuch B, Chow WH, et al. Obesity and renal cell carcinoma risk by histologic subtype: a nested case-control study and meta-analysis. Cancer Epidemiol. 2018;56:31–7. 30029068 10.1016/j.canep.2018.07.002 PMC6151870

[35] MacLeod LC, Hotaling JM, Wright JL, Davenport MT, Gore JL, Harper J, et al. Risk factors for renal cell carcinoma in the VITAL study. J Urol. 2013;190(5):1657–61. 23665301 10.1016/j.juro.2013.04.130 PMC4420476

[36] Keimling M, Behrens G, Schmid D, Jochem C, Leitzmann MF. The association between physical activity and bladder cancer: systematic review and meta-analysis. Br J Cancer. 2014;110(7):1862–70. 24594995 10.1038/bjc.2014.77 PMC3974090

[37] Padala SA, Barsouk A, Thandra KC, Saginala K, Mohammed A, Vakiti A, et al. Epidemiology of renal cell carcinoma. World J Oncol. 2020;11(3):79–87. 32494314 10.14740/wjon1279 PMC7239575

[38] Center MM, Jemal A, Lortet-Tieulent J, Ward E, Ferlay J, Brawley O, et al. International variation in prostate cancer incidence and mortality rates. Eur Urol. 2012;61(6):1079–92. 22424666 10.1016/j.eururo.2012.02.054

[39] Bray F, Piñeros M. Cancer patterns, trends and projections in Latin America and the Caribbean: a global context. Salud Publica Mex. 2016;58(2):104–17. 27557369 10.21149/spm.v58i2.7779

[40] Wong MCS, Goggins WB, Wang HHX, Fung FD, Leung C, Wong SY, et al. Global incidence and mortality for prostate cancer: analysis of temporal patterns and trends in 36 countries. Eur Urol. 2016;70(5):862–74. 27289567 10.1016/j.eururo.2016.05.043

[41] Smith-Palmer J, Takizawa C, Valentine W. Literature review of the burden of prostate cancer in Germany, France, the United Kingdom and Canada. BMC Urol. 2019;19(1):19. 30885200 10.1186/s12894-019-0448-6 PMC6421711

[42] Winter A, Sirri E, Jansen L, Wawroschek F, Kieschke J, Castro FA, et al. Comparison of prostate cancer survival in Germany and the USA: Can differences be attributed to differences in stage distributions?. BJU Int. 2017;119(4):550–9. 27208546 10.1111/bju.13537

[43] Tsodikov A, Gulati R, Heijnsdijk EAM, Pinsky PF, Moss SM, Qiu S, et al. Reconciling the effects of screening on prostate cancer mortality in the ERSPC and PLCO trials. Ann Intern Med. 2017;167(7):449–55. 28869989 10.7326/M16-2586 PMC5734053

[44] Etzioni R, Tsodikov A, Mariotto A, Szabo A, Falcon S, Wegelin J, et al. Quantifying the role of PSA screening in the US prostate cancer mortality decline. Cancer Causes Control. 2008;19(2):175–81. 18027095 10.1007/s10552-007-9083-8 PMC3064270

[45] Michaeli T, Michaeli D. Prostate cancer follow-up costs in Germany from 2000 to 2015. J Cancer Surviv. 2022;16(1):86–94. 33646503 10.1007/s11764-021-01006-w PMC8881276

[46] Hock LM, Lynch J, Balaji KC. Increasing incidence of all stages of kidney cancer in the last 2 decades in the United States: an analysis of surveillance, epidemiology and end results program data. J Urol. 2002;167(1):57–60. 11743275

[47] Babjuk M, Burger M, Zigeuner R, Shariat SF, van Rhijn BW, Compérat E, et al. EAU guidelines on non-muscle-invasive urothelial carcinoma of the bladder: update 2013. Eur Urol. 2013;64(4):639–53. 23827737 10.1016/j.eururo.2013.06.003

[48] Raheem OA, Khandwala YS, Sur RL, Ghani KR, Denstedt JD. Burden of urolithiasis: trends in prevalence, treatments, and costs. Eur Urol Focus. 2017;3(1):18–26. 28720363 10.1016/j.euf.2017.04.001

[49] Speakman M, Kirby R, Doyle S, Ioannou C. Burden of male lower urinary tract symptoms (LUTS) suggestive of benign prostatic hyperplasia (BPH)—focus on the UK. BJU Int. 2015;115(4):508–19. 24656222 10.1111/bju.12745

[50] Lu PL, Liu YC, Toh HS, Lee YL, Liu YM, Ho CM, et al. Epidemiology and antimicrobial susceptibility profiles of Gram-negative bacteria causing urinary tract infections in the Asia-Pacific region: 2009–2010 results from the Study for Monitoring Antimicrobial Resistance Trends (SMART). Int J Antimicrob Agents. 2012;40(Suppl):S37-43. 22749057 10.1016/S0924-8579(12)70008-0

[51] Li X, Fan H, Zi H, Hu H, Li B, Huang J, et al. Global and regional burden of bacterial antimicrobial resistance in urinary tract infections in 2019. J Clin Med. 2022;11(10):2817. 35628941 10.3390/jcm11102817 PMC9147874

[52] Chughtai B, Forde JC, Thomas DD, Laor L, Hossack T, Woo HH, et al. Benign prostatic hyperplasia. Nat Rev Dis Primers. 2016;2:16031. 27147135 10.1038/nrdp.2016.31

[53] Jin YH, Wang YP, Xie YL, Tian GH, Zhang XY, Shi NN, et al. Research on the development methodology for clinical practice guidelines for organic integration of traditional Chinese and Western medicine. Mil Med Res. 2023;10(1):45. 37752599 10.1186/s40779-023-00481-9 PMC10523673

