# Supplementary material for: Global burden of benign prostatic hyperplasia, urinary tract infections, urolithiasis, bladder cancer, kidney cancer, and prostate cancer from 1990 to 2021
Source: Mil Med Res. 2024 Sep 18;11:64. doi: 10.1186/s40779-024-00569-w (PMC11409598; doi:10.1186/s40779-024-00569-w)
Supplement: Supplementary file 1 — Additional file 1: Table S1 EAPC of the ASIR, ASPR, ASMR and ASDR of 3 urologic cancers in global and 21 regions. Table S2 EAPC of the ASIR, ASPR, ASMR and ASDR of 3 benign urologic diseases in global and 21 regions. Table S3 Regional prevalence cases and ASPR of the 6 urologic diseases in 2021. Table S4 Regional death cases and ASMR of the 6 urologic diseases in 2021. Table S5 Regional DALYs and ASDR of the 6 urologic diseases in 2021. Table S6 Age-standardized incidence, prevalence, mortality, and DALY rates of the 6 urologic diseases among the top 3 and bottom 3 countries in 2021. Table S7 EAPC of ASIR for the 6 urologic diseases in 204 countries and territories from 1990 to 2021. Table S8 EAPC of ASPR for the 6 urologic diseases in 204 countries and territories from 1990 to 2021. Table S9 EAPC of ASMR for the 6 urologic diseases in 204 countries and territories from 1990 to 2021. Table S10 EAPC of ASDR for the 6 urologic diseases in 204 countries and territories from 1990 to 2021. Table S11 Percentage of urologic cancers deaths attributable to risk factors in 2021. Table S12 Percentage of urologic cancers DALYs attributable to risk factors in 2021. Fig. S1 The EAPC of ASPR for 6 urologic diseases in global and 21 regions. Fig. S2 The EAPC of ASMR for 5 urologic diseases in global and 21 regions. Fig. S3 The EAPC of ASDR for 6 urologic diseases in global and 21 regions. Fig. S4 The ASIR for 6 urologic diseases in 21 regions from 1990 to 2021. Fig. S5 The ASPR for 6 urologic diseases in 21 regions from 1990 to 2021. Fig. S6 The ASMR for 5 urologic diseases in 21 regions from 1990 to 2021. Fig. S7 The ASDR for 6 urologic diseases in 21 regions from 1990 to 2021. Fig. S8 ASPR of 6 urologic diseases for 204 countries and territories by SDI. Fig. S9 ASMR of 5 urologic diseases for 204 countries and territories by SDI. Fig. S10 ASDR of 6 urologic diseases for 204 countries and territories by SDI. Fig. S11 Global prevalence of 6 urologic diseases by age and sex in 2021. [file 40779_2024_569_MOESM1_ESM.pdf]

Table S1 EAPC of the ASIR, ASPR, ASMR and ASDR of 3 benign urologic diseases in global and 21 regions

Location	EAPC of ASIR (95% CI)			EAPC of ASPR (95% CI)			EAPC of ASMR (95% CI)			EAPC of ASDR (95% CI)		
	**BPH**	**UTI**	**Urolithiasis**	**BPH**	**UTI**	**Urolithiasis**	**BPH**	**UTI**	**Urolithiasis**	**BPH**	**UTI**	**Urolithiasis**
Global	0.03 (-0.02 to 0.08)	0.15 (0.10 − 0.20)	-0.87 (-0.91 to -0.84)	-0.01 (-0.06 to 0.04)	0.15 (0.10 − 0.19)	-0.87 (-0.90 to -0.84)	–	1.02 (0.95 − 1.10)	-1.02 (-1.24 to -0.80)	0.00 (-0.05 to 0.05)	0.42 (0.35 − 0.49)	-1.15 (-1.28 to -1.02)
Andean Latin America	0.18 (0.15 − 0.22)	0.34 (0.29 − 0.38)	0.35 (0.26 − 0.43)	0.15 (0.11 − 0.18)	0.33 (0.29 − 0.38)	0.34 (0.26 − 0.43)	–	1.60 (1.29 − 1.92)	1.01 (0.72 − 1.31)	0.14 (0.10 − 0.17)	0.87 (0.61 − 1.13)	0.41 (0.32 − 0.50)
Australasia	0.10 (0.00 − 0.20)	0.08 (0.07 − 0.09)	-0.46 (-0.57 to -0.35)	0.03 (-0.01 to 0.07)	0.08 (0.07 − 0.09)	-0.45 (-0.56 to -0.34)	–	1.34 (0.89 − 1.79)	-0.77 (-1.41 to -0.12)	0.03 (0 − 0.07)	0.71 (0.36 − 1.07)	-0.65 (-0.98 to -0.33)
Caribbean	0.21 (0.19 − 0.24)	-0.09 (-0.1 to -0.08)	0.83 (0.81 − 0.85)	0.19 (0.18 − 0.21)	-0.09 (-0.10 to -0.08)	0.83 (0.81 − 0.86)	–	2.74 (2.23 − 3.26)	2.29 (1.82 − 2.76)	0.17 (0.15 − 0.19)	2.31 (1.94 − 2.67)	1.60 (1.35 − 1.84)
Central Asia	0.02 (-0.01 − 0.05)	0.21 (0.17 − 0.24)	0.25 (0.23 − 0.27)	0.05 (0.03 − 0.07)	0.21 (0.17 − 0.24)	0.25 (0.24 − 0.27)	–	1.68 (1.13 − 2.24)	1.53 (1.19 − 1.87)	0.05 (0.03 − 0.07)	0.95 (0.48 − 1.43)	0.49 (0.30 − 0.69)
Central Europe	-0.22 (-0.28 to -0.16)	-0.39 (-0.54 to -0.24)	-1.18 (-1.45 to -0.92)	-0.15 (-0.25 to -0.04)	-0.39 (-0.55 to -0.24)	-1.18 (-1.45 to -0.92)	–	0.41 (-0.93 to 1.76)	-5.60 (-6.51 to -4.68)	-0.13 (-0.23 to -0.02)	-0.22 (-1.45 to 1.04)	-3.36 (-3.73 to -2.99)
Central Latin America	0.15 (0.12 − 0.18)	0.63 (0.45 − 0.82)	0.42 (0.05 − 0.79)	0.12 (0.09 − 0.15)	0.64 (0.45 − 0.83)	0.42 (0.05 − 0.79)	–	2.77 (2.35 − 3.20)	-0.54 (-0.63 to -0.44)	0.13 (0.10 to 0.16)	2.60 (2.14 − 3.06)	-0.13 (-0.28 to 0.02)
Central Sub-Saharan Africa	0.03 (0.02 − 0.05)	0.10 (0.09 − 0.11)	0.28 (0.25 − 0.30)	0.03 (0.02 − 0.04)	0.10 (0.09 − 0.11)	0.28 (0.25 − 0.30)	–	-0.54 (-0.64 to -0.45)	-0.42 (-0.51 to -0.33)	0.06 (0.05 to 0.07)	-0.73 (-0.8 to -0.66)	-0.12 (-0.16 to -0.07)
East Asia	-0.21 (-0.35 to -0.06)	-0.23 (-0.3 to -0.17)	-2.26 (-2.44 to -2.09)	-0.23 (-0.39 to -0.08)	-0.24 (-0.30 to -0.17)	-2.26 (-2.44 to -2.09)	–	-1.43 (-1.66 to -1.19)	-4.26 (-4.5 to -4.02)	-0.25 (-0.41 to -0.09)	-2.25 (-2.51 to -2.00)	-3.64 (-3.86 to -3.43)
Eastern Europe	-0.02 (-0.05 to 0.01)	0.23 (0.10 − 0.35)	-0.71 (-0.76 to -0.67)	0 (-0.02 to 0.01)	0.22 (0.10 − 0.35)	-0.71 (-0.76 to -0.67)	–	0.42 (-0.45 to 1.30)	0.14 (-0.39 to 0.67)	0.02 (0.00 − 0.04)	-0.31 (-0.96 to 0.34)	-0.61 (-0.85 to -0.37)
Eastern Sub-Saharan Africa	0.02 (0.00 − 0.03)	0.10 (0.08 − 0.13)	-0.12 (-0.15 to -0.08)	0.01 (0 − 0.03)	0.10 (0.08 − 0.13)	-0.12 (-0.16 to -0.08)	–	-0.65 (-0.76 to -0.54)	-1.12 (-1.18 to -1.06)	0.04 (0.02 − 0.05)	-1.05 (-1.16 to -0.94)	-1.08 (-1.13 to -1.02)
High-income Asia Pacific	-0.08 (-0.18 to 0.01)	0.03 (-0.03 to 0.08)	-0.23 (-0.28 to -0.19)	-0.15 (-0.22 to -0.08)	0.03 (-0.03 to 0.08)	-0.24 (-0.28 to -0.19)	–	2.29 (1.90 − 2.68)	3.37 (3.19 − 3.56)	-0.13 (-0.21 to -0.06)	1.66 (1.37 − 1.95)	0.71 (0.63 − 0.78)
High-income North America	0.47 (0.38 − 0.56)	-0.32 (-0.37 to -0.27)	-1.94 (-2.26 to -1.61)	0.24 (0.17 to 0.31)	-0.34 (-0.39 to -0.29)	-1.94 (-2.26 to -1.62)	–	0.15 (-0.14 to 0.43)	2.19 (1.83 to 2.54)	0.2 (0.13 to 0.27)	0.28 (0.06 − 0.50)	-0.22 (-0.52 to 0.09)

Location	EAPC of ASIR (95% CI)			EAPC of ASPR (95% CI)			EAPC of ASMR (95% CI)			EAPC of ASDR (95% CI)		
	BPH	UTI	Urolithiasis	BPH	UTI	Urolithiasis	BPH	UTI	Urolithiasis	BPH	UTI	Urolithiasis
North Africa and Middle East	0.13 (0.12 − 0.14)	0.10 (0.06 − 0.14)	0.25 (0.20 − 0.29)	0.13 (0.13 to 0.14)	0.09 (0.05 − 0.13)	0.25 (0.20 − 0.29)	–	0.08 (-0.05 to 0.22)	1.03 (0.85 to 1.22)	0.12 (0.11 − 0.13)	-0.36 (-0.45 to -0.27)	0.28 (0.18 − 0.38)
Oceania	0.12 (0.10 − 0.13)	0.06 (0.05 − 0.07)	-0.05 (-0.12 − 0.03)	0.14 (0.13 to 0.15)	0.06 (0.05 − 0.06)	-0.05 (-0.12 to 0.03)	–	-0.45 (-0.51 to -0.39)	-1.68 (-1.83 to -1.52)	0.14 (0.13 − 0.16)	-0.42 (-0.46 to -0.37)	-0.21 (-0.26 to -0.16)
South Asia	0.21 (0.16 − 0.25)	0.32 (0.25 − 0.38)	0.14 (0.02 − 0.26)	0.22 (0.19 to 0.25)	0.32 (0.25 − 0.38)	0.14 (0.02 to 0.26)	–	0.73 (0.62 to 0.83)	-0.95 (-1.16 to -0.75)	0.24 (0.21 to 0.27)	0.18 (0.11 − 0.24)	-0.72 (-0.85 to -0.58)
Southeast Asia	-0.10 (-0.18 to -0.03)	-0.13 (-0.18 to -0.08)	-0.53 (-0.59 to -0.47)	-0.13 (-0.22 to -0.03)	-0.13 (-0.17 to -0.08)	-0.54 (-0.60 to -0.48)	–	1.03 (0.97 to 1.09)	0.06 (-0.01 to 0.12)	-0.12 (-0.21 to -0.02)	0.36 (0.32 − 0.40)	-0.41 (-0.50 to -0.33)
Southern Latin America	0.38 (0.28 − 0.48)	0.09 (0.00 − 0.18)	0.24 (0.13 − 0.35)	0.33 (0.29 − 0.38)	0.09 (0.00 − 0.18)	0.24 (0.13 − 0.34)	–	5.37 (4.71 − 6.04)	1.51 (0.86 − 2.16)	0.33 (0.28 − 0.37)	4.82 (4.24 − 5.41)	0.41 (0.28 − 0.54)
Southern Sub-Saharan Africa	0.11 (0.09 − 0.13)	-0.03 (-0.06 to 0.01)	-0.10 (-0.17 to -0.02)	0.12 (0.10 − 0.14)	-0.03 (-0.06 to 0.01)	-0.10 (-0.17 to -0.02)	–	0.25 (-0.25 to 0.75)	0.95 (0.34 to 1.55)	0.1 (0.08 to 0.12)	0.23 (-0.32 to 0.78)	0.21 (-0.14 to 0.57)
Tropical Latin America	-0.25 (-0.36 to -0.14)	-0.01 (-0.09 to 0.08)	1.57 (1.12 to 2.02)	-0.32 (-0.43 to -0.20)	0.00 (-0.09 to 0.08)	1.58 (1.12 − 2.03)	–	4.41 (4.02 − 4.80)	3.70 (3.48 to 3.92)	-0.31 (-0.43 to -0.2)	3.31 (2.98 − 3.64)	2.90 (2.65 − 3.16)
Western Europe	0.38 (0.25 − 0.50)	-0.20 (-0.31 to -0.10)	0.16 (0.00 − 0.32)	0.20 (0.17 − 0.23)	-0.20 (-0.31 to -0.10)	0.16 (0.00 − 0.32)	–	2.59 (2.18 − 3.00)	-0.60 (-0.91 to -0.29)	0.21 (0.18 to 0.24)	2.00 (1.64 − 2.35)	-0.13 (-0.23 to -0.03)
Western Sub-Saharan Africa	0.00 (-0.01 to 0.01)	0.20 (0.17 to 0.23)	-0.02 (-0.07 to 0.03)	0.01 (0.00 − 0.02)	0.20 (0.17 − 0.23)	-0.02 (-0.07 to 0.03)	–	-0.38 (-0.51 to -0.26)	-0.46 (-0.54 to -0.38)	0.04 (0.03 to 0.05)	-0.73 (-0.85 to -0.61)	-0.60 (-0.65 to -0.54)

BPH benign prostatic hyperplasia, *UTI* urinary tract infections, *EAPC* estimated annual percentage change, *ASIR* age-standardized incidence rate, *ASPR* age-standardized prevalence rate, *ASMR* age-standardized mortality rate, *ASDR* age-standardized DALYs rate, *CI* confidence interval

Table S2 EAPC of the ASIR, ASPR, ASMR and ASDR of 3 urologic cancers in global and 21 regions

Location	EAPC of ASIR (95% CI)			EAPC of ASPR (95% CI)			EAPC of ASMR (95% CI)			EAPC of ASDR (95% CI)		
	**Bladder cancer**	**Kidney cancer**	**Prostate cancer**	**Bladder cancer**	**Kidney cancer**	**Prostate cancer**	**Bladder cancer**	**Kidney cancer**	**Prostate cancer**	**Bladder cancer**	**Kidney cancer**	**Prostate cancer**
Global	-0.36 (-0.41 to -0.30)	0.53 (0.40 − 0.66)	-0.06 (-0.20 to 0.08)	0.10 (0.01 − 0.19)	0.98 (0.80 − 1.16)	0.42 (0.27 − 0.58)	-0.98 (-1.03 to -0.94)	-0.14 (-0.21 to -0.07)	-1.05 (-1.14 to -0.95)	-1.19 (-1.24 to -1.13)	-0.37 (-0.43 to -0.30)	-0.96 (-1.05 to -0.88)
Andean Latin America	0.47 (0.31 − 0.64)	1.18 (1.00 − 1.36)	1.47 (1.33 − 1.62)	1.59 (1.45 − 1.73)	2.20 (2.03 − 2.37)	2.76 (2.54 − 2.99)	-0.35 (-0.51 to -0.20)	0.27 (0.11 to 0.42)	-0.11 (-0.25 to 0.04)	-0.46 (-0.61 to -0.30)	-0.10 (-0.28 to 0.07)	-0.11 (-0.24 to 0.03)
Australasia	-1.03 (-1.14 to -0.92)	0.92 (0.70 − 1.14)	-0.83 (-1.47 to -0.19)	-0.85 (-0.94 to -0.76)	1.78 (1.45 − 2.11)	-0.10 (-0.75 to 0.56)	-1.15 (-1.24 to -1.05)	-0.51 (-0.59 to -0.43)	-2.74 (-3.06 to -2.43)	-1.50 (-1.59 to -1.40)	-0.64 (-0.73 to -0.55)	-2.67 (-3.05 to -2.29)
Caribbean	0.62 (0.50 − 0.75)	1.01 (0.94 − 1.08)	1.30 (1.11 − 1.50)	1.25 (1.11 − 1.40)	1.60 (1.51 − 1.68)	1.77 (1.57 − 1.98)	0.06 (-0.07 to 0.19)	0.40 (0.31 − 0.49)	0.21 (0.05 − 0.38)	0.14 (0.02 − 0.26)	0.26 (0.17 − 0.35)	0.36 (0.21 − 0.51)
Central Asia	-0.27 (-0.43 to -0.12)	1.02 (0.84 − 1.20)	1.76 (1.43 − 2.09)	-0.11 (-0.26 to 0.04)	1.33 (1.17 − 1.50)	2.10 (1.77 − 2.43)	-0.47 (-0.64 to -0.30)	0.68 (0.47 − 0.89)	1.03 (0.75 − 1.31)	-0.87 (-1.07 to -0.66)	0.43 (0.21 − 0.64)	0.79 (0.55 − 1.02)
Central Europe	1.25 (1.12 − 1.38)	1.33 (1.08 − 1.59)	2.27 (2.04 − 2.49)	1.92 (1.74 − 2.09)	2.06 (1.72 − 2.40)	3.27 (3.00 − 3.54)	0.40 (0.32 − 0.49)	0.52 (0.35 − 0.69)	0.45 (0.25 − 0.64)	0.24 (0.15 − 0.33)	0.23 (0.03 − 0.43)	0.49 (0.31 − 0.67)
Central Latin America	0.01 (-0.09 to 0.12)	1.67 (1.62 − 1.71)	1.43 (1.10 − 1.76)	0.85 (0.71 − 1.00)	2.59 (2.53 − 2.65)	1.94 (1.58 − 2.31)	-0.67 (-0.77 to -0.57)	0.85 (0.77 − 0.92)	-0.31 (-0.54 to -0.09)	-0.63 (-0.74 to -0.52)	0.72 (0.65 − 0.80)	-0.22 (-0.43 to 0)
Central Sub-Saharan Africa	-0.05 (-0.25 to 0.15)	0.74 (0.50 − 0.97)	0.93 (0.77 − 1.09)	0.60 (0.34 − 0.86)	1.52 (1.24 − 1.80)	1.79 (1.56 − 2.02)	-0.33 (-0.48 to -0.17)	0.36 (0.15 − 0.57)	0.64 (0.51 − 0.77)	-0.31 (-0.46 to -0.15)	0.30 (0.11 − 0.49)	0.57 (0.45 − 0.70)
East Asia	0.12 (0.04 − 0.21)	2.40 (2.22 − 2.59)	1.93 (1.78 − 2.09)	1.55 (1.50 − 1.59)	3.42 (3.20 − 3.64)	3.75 (3.58 − 3.91)	-1.56 (-1.69 to -1.42)	0.49 (0.33 − 0.64)	-0.24 (-0.46 to -0.01)	-1.70 (-1.84 to -1.57)	0.06 (-0.08 to 0.20)	-0.20 (-0.39 to -0.01)
Eastern Europe	0.13 (-0.09 to 0.34)	0.99 (0.79 − 1.18)	3.27 (3.08 − 3.47)	0.62 (0.47 − 0.77)	1.22 (1.02 − 1.42)	3.73 (3.45 − 4.00)	-0.61 (-0.90 to -0.31)	0.37 (0.16 − 0.57)	1.48 (1.39 to 1.57)	-0.82 (-1.14 to -0.50)	-0.09 (-0.32 to 0.14)	1.51 (1.41 − 1.60)
Eastern Sub-Saharan Africa	-0.24 (-0.32 to -0.17)	0.90 (0.77 − 1.03)	0.59 (0.54 − 0.65)	0.37 (0.27 − 0.47)	1.57 (1.37 − 1.76)	1.68 (1.59 − 1.78)	-0.47 (-0.53 to -0.41)	0.52 (0.42 − 0.63)	0.15 (0.12 to 0.19)	-0.62 (-0.69 to -0.56)	0.22 (0.09 − 0.35)	0.18 (0.15 − 0.21)
High-income Asia Pacific	0.29 (0.22 − 0.37)	1.28 (0.98 − 1.58)	2.35 (1.83 − 2.87)	0.67 (0.58 − 0.76)	1.97 (1.57 − 2.38)	3.02 (2.48 − 3.56)	-0.46 (-0.53 to -0.40)	0.33 (0.14 − 0.52)	-0.09 (-0.37 to 0.20)	-0.63 (-0.69 to -0.57)	-0.10 (-0.30 to 0.10)	0 (-0.28 to 0.28)
High-income North America	0.03 (-0.15 to 0.20)	0 (-0.22 − 0.22)	-1.25 (-1.38 to -1.12)	0.16 (-0.05 to 0.36)	0.19 (-0.07 to 0.46)	-0.86 (-0.97 to -0.74)	-0.17 (-0.25 to -0.08)	-0.51 (-0.64 to -0.38)	-2.40 (-2.55 to -2.26)	-0.40 (-0.51 to -0.30)	-0.81 (-0.93 to -0.68)	-2.25 (-2.40 to -2.10)
North Africa and Middle East	0.40 (0.29 − 0.52)	1.89 (1.81 − 1.97)	2.81 (2.72 − 2.91)	1.16 (1.04 − 1.29)	2.44 (2.34 − 2.53)	3.86 (3.73 − 3.99)	-0.90 (-1.03 to -0.78)	0.77 (0.71 to 0.84)	0.38 (0.30 to 0.46)	-1.23 (-1.38 to -1.08)	0.45 (0.39 − 0.51)	0.51 (0.44 − 0.58)

Location	EAPC of ASIR (95% CI)			EAPC of ASPR (95% CI)			EAPC of ASMR (95% CI)			EAPC of ASDR (95% CI)		
	**Bladder cancer**	**Kidney cancer**	**Prostate cancer**	**Bladder cancer**	**Kidney cancer**	**Prostate cancer**	**Bladder cancer**	**Kidney cancer**	**Prostate cancer**	**Bladder cancer**	**Kidney cancer**	**Prostate cancer**
Oceania	0.85 (0.80 − 0.89)	0.32 (0.25 − 0.40)	0.76 (0.72 − 0.80)	1.20 (1.16 − 1.24)	0.34 (0.26 − 0.43)	1.21 (1.17 − 1.24)	0.54 (0.48 − 0.60)	0.31 (0.25 − 0.37)	0.50 (0.44 − 0.55)	0.56 (0.50 − 0.63)	0.33 (0.29 − 0.37)	0.53 (0.48 − 0.58)
South Asia	0.04 (-0.10 to 0.19)	1.63 (1.54 − 1.72)	0.97 (0.83 − 1.12)	0.82 (0.65 − 0.99)	2.61 (2.52 − 2.69)	2.31 (2.16 − 2.45)	-0.49 (-0.62 to -0.36)	0.95 (0.86 − 1.04)	0.21 (0.07 − 0.35)	-0.53 (-0.65 to -0.41)	0.78 (0.70 − 0.86)	0.08 (-0.06 to 0.22)
Southeast Asia	0.69 (0.64 − 0.74)	1.00 (0.85 − 1.15)	1.87 (1.81 − 1.93)	1.58 (1.49 − 1.67)	1.37 (1.19 − 1.54)	2.92 (2.86 − 2.98)	-0.21 (-0.26 to -0.17)	0.43 (0.29 − 0.57)	0.79 (0.71 − 0.88)	-0.29 (-0.34 to -0.25)	0.36 (0.23 − 0.48)	0.81 (0.74 − 0.88)
Southern Latin America	-0.66 (-0.76 to -0.57)	1.40 (1.24 − 1.56)	0.86 (0.48 − 1.24)	-0.06 (-0.17 to 0.06)	1.75 (1.59 − 1.92)	1.83 (1.43 − 2.22)	-1.27 (-1.36 to -1.19)	0.68 (0.56 − 0.81)	-0.45 (-0.78 to -0.13)	-1.35 (-1.43 to -1.27)	0.44 (0.35 − 0.53)	-0.55 (-0.87 to -0.22)
Southern Sub-Saharan Africa	0.39 (0.21 − 0.58)	1.80 (1.66 − 1.93)	1.41 (1.27 − 1.55)	0.53 (0.47 − 0.59)	2.17 (2.04 − 2.29)	1.99 (1.92 − 2.06)	0.19 (-0.05 to 0.42)	1.41 (1.23 − 1.58)	0.89 (0.69 − 1.09)	0.25 (0.02 − 0.49)	1.28 (1.13 − 1.44)	0.94 (0.73 − 1.14)
Tropical Latin America	0.29 (0.22 − 0.37)	1.87 (1.75 − 1.99)	0.95 (0.60 − 1.31)	0.94 (0.85 − 1.03)	2.55 (2.39 − 2.7)	1.71 (1.32 − 2.11)	-0.20 (-0.25 to -0.14)	1.23 (1.13 − 1.32)	-0.03 (-0.36 to 0.30)	-0.33 (-0.39 to -0.27)	0.86 (0.75 − 0.98)	-0.11 (-0.44 to 0.22)
Western Europe	-0.46 (-0.54 to -0.38)	0.86 (0.69 − 1.03)	0.41 (0.08 − 0.75)	-0.01 (-0.14 to 0.13)	1.48 (1.22 − 1.73)	1.13 (0.75 − 1.52)	-1.13 (-1.19 to -1.08)	-0.27 (-0.33 to -0.21)	-1.85 (-1.99 to -1.72)	-1.34 (-1.38 to -1.30)	-0.58 (-0.64 to -0.51)	-1.68 (-1.82 to -1.55)
Western Sub-Saharan Africa	0.20 (0.15 − 0.25)	1.68 (1.58 − 1.78)	1.25 (1.20 − 1.31)	0.58 (0.52 − 0.64)	2.14 (2.00 − 2.29)	2.13 (2.03 − 2.22)	-0.02 (-0.07 to 0.03)	1.29 (1.20 − 1.38)	0.87 (0.83 − 0.92)	-0.10 (-0.16 to -0.04)	1.03 (0.92 − 1.13)	0.8 (0.76 − 0.84)

EAPC estimated annual percentage change, *ASIR* age-standardized incidence rate, *ASPR* age-standardized prevalence rate, *ASMR* age-standardized mortality rate, *ASDR* age-standardized DALYs rate, *CI* confidence interval

Table S3 Regional prevalence and ASPR of the 6 urologic diseases in 2021

Location	BPH		UTI		Urolithiasis		Bladder cancer		Kidney cancer		Prostate cancer	
	**Prevalence ($\times 10^5$, 95% UI)**	**ASPR (1/100,000, 95% UI)**	**Prevalence ($\times 10^5$, 95% UI)**	**ASPR (1/100,000, 95% UI)**	**Prevalence ($\times 10^5$, 95% UI)**	**ASPR (1/100,000, 95% UI)**	**Prevalences ($\times 10^5$, 95% UI)**	**ASPR (1/100,000, 95% UI)**	**Prevalence ($\times 10^5$, 95% UI)**	**ASPR (1/100,000, 95% UI)**	**Prevalence ($\times 10^5$, 95% UI)**	**ASPR (1/100,000, 95% UI)**
Global	1125.02 (881.32 − 1426.34)	2782.59 (2191.58 − 3508.04)	85.61 (76.74 − 94.97)	105.35 (94.44 − 116.98)	40.21 (33.64 − 48.16)	47.10 (39.41 − 56.31)	30.26 (28.23 − 32.24)	34.91 (32.54 − 37.19)	19.61 (18.62 − 20.52)	22.70 (21.54 − 23.76)	103.88 (97.06 − 109.04)	260.05 (243.39 − 272.68)
High SDI	187.79 (155.40 − 227.86)	1927.97 (1605.03 − 2322.83)	14.16 (12.90 − 15.50)	116.26 (105.24 − 127.96)	6.64 (5.48 − 8.02)	44.31 (36.92 − 52.96)	13.70 (12.79 − 14.31)	66.81 (62.85 − 69.58)	9.08 (8.65 − 9.42)	50.66 (48.52 − 52.45)	59.88 (56.61 − 62.45)	612.57 (579.53 − 638.50)
High-middle SDI	262.59 (205.04 − 330.79)	2881.35 (2275.34 − 3621.68)	10.69 (9.69 − 11.83)	76.98 (69.53 − 85.00)	9.59 (8.00 − 11.40)	54.65 (45.88 − 64.93)	9.42 (8.70 − 10.38)	47.22 (43.56 − 52.05)	6.11 (5.70 − 6.55)	33.03 (30.88 − 35.32)	20.60 (18.50 − 22.29)	225.91 (202.96 − 244.08)
Middle SDI	386.37 (298.30 − 493.36)	3097.50 (2433.52 − 3911.51)	25.44 (22.78 − 28.31)	98.14 (87.93 − 108.87)	13.64 (11.34 − 16.45)	48.48 (40.54 − 57.82)	4.99 (4.31 − 5.86)	18.00 (15.60 − 21.03)	3.19 (2.89 − 3.51)	11.56 (10.5 − 12.70)	15.91 (13.75 − 17.87)	125.63 (108.85 − 140.62)
Low-middle SDI	222.60 (166.71 − 289.73)	3388.59 (2569.07 − 4372.07)	25.88 (22.86 − 29.04)	137.82 (122.29 − 154.65)	7.70 (6.33 − 9.40)	42.94 (35.53 − 52.24)	1.59 (1.39 − 2.10)	10.56 (9.19 − 13.93)	0.87 (0.77 − 0.97)	5.19 (4.65 − 5.79)	5.71 (4.79 − 6.56)	85.84 (72.80 − 98.2)
Low SDI	64.57 (48.50 − 85.10)	2837.28 (2142.20 − 3707.99)	9.37 (8.23 − 10.57)	99.99 (88.67 − 113.27)	2.62 (2.10 − 3.21)	31.80 (26.06 − 38.91)	0.51 (0.44 − 0.60)	9.39 (8.09 − 10.87)	0.34 (0.23 − 0.45)	3.89 (2.81 − 4.91)	1.64 (1.09 − 2.02)	70.35 (47.20 − 86.16)
Andean Latin America	7.91 (5.82 − 10.37)	2877.23 (2127.17 − 3752.32)	1.58 (1.35 − 1.79)	234.98 (201.52 − 263.81)	0.44 (0.36 − 0.53)	65.56 (54.57 − 79.42)	0.06 (0.05 − 0.08)	10.47 (8.40 − 13.10)	0.09 (0.07 − 0.12)	14.83 (11.79 − 18.48)	0.76 (0.55 − 1.04)	275.22 (201.7 − 378.08)
Australasia	5.21 (3.85 − 6.85)	1979.78 (1482.19 − 2581.41)	0.56 (0.50 − 0.64)	172.96 (153.3 − 196.24)	0.19 (0.16 − 0.24)	48.50 (39.37 − 59.73)	0.23 (0.21 − 0.25)	43.51 (39.94 − 46.72)	0.23 (0.21 − 0.26)	50.38 (44.57 − 56.04)	2.35 (2.04 − 2.71)	906.45 (787.27 − 1045.55)
Caribbean	7.69 (5.65 − 10.02)	3060.81 (2253.52 − 3991.28)	0.73 (0.65 − 0.83)	149.97 (133.1 − 170.12)	0.26 (0.21 − 0.31)	48.95 (40.64 − 58.76)	0.13 (0.12 − 0.15)	24.30 (21.43 − 27.20)	0.07 (0.06 − 0.08)	13.77 (12.07 − 15.49)	1.64 (1.38 − 1.89)	653.29 (550.36 − 754.63)
Central Asia	9.43 (7.09 − 12.44)	2826.87 (2151.52 − 3627.82)	1.22 (1.07 − 1.41)	125.69 (110.11 − 145.8)	0.68 (0.57 − 0.81)	70.93 (59.81 − 83.70)	0.15 (0.13 − 0.17)	17.06 (15.08 − 18.99)	0.15 (0.13 − 0.17)	15.98 (13.85 − 17.99)	0.31 (0.28 − 0.35)	89.92 (81.23 − 99.58)
Central Europe	31.64 (26.22 − 38.35)	3222.04 (2698.56 − 3877.95)	1.05 (0.96 − 1.14)	86.64 (80.10 − 94.11)	0.60 (0.50 − 0.72)	39.43 (32.90 − 47.06)	1.42 (1.31 − 1.53)	65.57 (60.19 − 70.42)	0.76 (0.69 − 0.82)	38.70 (35.29 − 42.12)	3.38 (3.07 − 3.70)	338.01 (306.97 − 369.88)
Central Latin America	53.74 (42.42 − 66.93)	4775.05 (3770.46 − 5939.43)	3.82 (3.48 − 4.21)	151.14 (137.75 − 166.41)	1.19 (0.99 − 1.42)	44.71 (37.27 − 53.30)	0.28 (0.25 − 0.31)	11.03 (9.94 − 12.28)	0.47 (0.42 − 0.52)	17.88 (15.92 − 20.03)	5.58 (4.80 − 6.40)	492.55 (424.5 − 563.85)
Central Sub-Saharan Africa	4.76 (3.5 − 6.53)	2143.08 (1598.98 − 2845.61)	0.80 (0.70 − 0.92)	70.82 (61.61 − 79.49)	0.24 (0.19 − 0.30)	25.31 (20.5 − 31.26)	0.06 (0.05 − 0.08)	10.58 (8.25 − 13.42)	0.02 (0.02 − 0.04)	2.82 (1.76 − 4.32)	0.21 (0.13 − 0.28)	95.7 (60.18 − 128.31)

Location	BPH		UTI		Urolithiasis		Bladder cancer		Kidney cancer		Prostate cancer	
	**Prevalence (× 10^5, 95% UI)**	**ASPR (1/100,000, 95% UI)**	**Prevalence (× 10^5, 95% UI)**	**ASPR (1/100,000, 95% UI)**	**Prevalence (× 10^5, 95% UI)**	**ASPR (1/100,000, 95% UI)**	**Prevalences (× 10^5, 95% UI)**	**ASPR (1/100,000, 95% UI)**	**Prevalence (× 10^5, 95% UI)**	**ASPR (1/100,000, 95% UI)**	**Prevalence (× 10^5, 95% UI)**	**ASPR (1/100,000, 95% UI)**
East Asia	243.04 (189.22 − 316.11)	2361.87 (1862.45 − 2997.80)	4.16 (3.68 − 4.68)	22.61 (20.16 − 25.51)	7.63 (6.23 − 9.34)	36.97 (30.51 − 44.56)	5.99 (4.80 − 7.65)	27.02 (21.71 − 34.35)	3.70 (3.06 − 4.44)	18.36 (15.36 − 21.76)	6.92 (5.13 − 9.16)	64.31 (47.85 − 84.41)
Eastern Europe	84.97 (64.48 − 107.99)	6262.23 (4821.08 − 7834.28)	3.60 (3.26 − 4.02)	173.88 (158.02 − 192.39)	3.88 (3.26 − 4.54)	134.55 (113.03 − 158.80)	1.22 (1.11 − 1.33)	34.91 (31.69 − 38.02)	1.54 (1.42 − 1.68)	47.22 (43.34 − 51.24)	5.71 (5.12 − 6.26)	411.07 (370.69 − 448.37)
Eastern Sub-Saharan Africa	17.55 (12.95 − 23.75)	2421.32 (1784.74 − 3225.77)	2.32 (2.01 − 2.66)	67.52 (59.45 − 75.95)	0.82 (0.66 − 1.01)	26.93 (21.97 − 33.35)	0.19 (0.16 − 0.24)	10.46 (8.60 − 13.02)	0.17 (0.10 − 0.23)	5.44 (3.64 − 7.06)	0.70 (0.46 − 0.90)	93.37 (62.31 − 118.20)
High-income Asia Pacific	24.20 (18.70 − 31.35)	1167.78 (902.09 − 1524.86)	2.16 (1.95 − 2.39)	119.87 (109.07 − 131.54)	1.41 (1.15 − 1.74)	52.35 (42.98 − 64.12)	2.01 (1.81 − 2.16)	44.53 (40.70 − 48.00)	0.85 (0.78 − 0.91)	23.86 (22.23 − 25.12)	5.34 (4.75 − 5.87)	237.31 (211.16 − 260.35)
High-income North America	55.53 (48.53 − 63.47)	1818.90 (1599.15 − 2062.68)	5.60 (5.13 − 6.13)	124.16 (113.72 − 135.59)	1.82 (1.53 − 2.14)	36.53 (31.07 − 42.83)	6.19 (5.81 − 6.44)	94.8 (89.41 − 98.40)	4.12 (3.89 − 4.27)	70.07 (66.69 − 72.40)	28.29 (26.98 − 29.47)	910.62 (867.85 − 948.35)
North Africa and Middle East	47.06 (34.71 − 63.39)	2168.64 (1598.24 − 2888.35)	4.94 (4.32 − 5.58)	77.21 (68.09 − 87.05)	2.01 (1.60 − 2.56)	32.34 (26.00 − 40.91)	2.23 (1.89 − 2.70)	47.9 (40.79 − 57.51)	0.90 (0.79 − 1.01)	16.38 (14.5 − 18.44)	4.32 (3.10 − 5.25)	200.42 (144.79 − 242.38)
Oceania	1.17 (0.89 − 1.53)	3632.80 (2754.12 − 4576.18)	0.05 (0.04 − 0.05)	38.29 (33.00 − 44.65)	0.04 (0.03 − 0.05)	35.44 (28.90 − 43.77)	0.01 (0.01 − 0.01)	8.82 (5.79 − 11.63)	0.00 (0.00 − 0.00)	2.51 (1.65 − 3.54)	0.03 (0.02 − 0.05)	101.39 (67.82 − 137.57)
South Asia	261.16 (192.77 − 339.67)	3749.01 (2817.43 − 4827.64)	31.69 (27.91 − 35.66)	171.33 (151.92 − 192.60)	8.64 (7.01 − 10.65)	47.31 (38.57 − 58.14)	1.19 (1.03 − 1.43)	7.73 (6.70 − 9.33)	0.58 (0.53 − 0.64)	3.55 (3.21 − 3.93)	2.63 (2.14 − 3.51)	36.99 (30.23 − 49.02)
Southeast Asia	122.24 (93.20 − 158.12)	4189.58 (3228.55 − 5327.26)	4.09 (3.61 − 4.62)	54.67 (48.43 − 61.33)	4.65 (3.89 − 5.51)	61.62 (51.95 − 72.50)	0.83 (0.71 − 1.00)	12.01 (10.29 − 14.47)	0.69 (0.61 − 0.78)	9.53 (8.39 − 10.79)	2.74 (1.83 − 3.37)	95.91 (64.65 − 117.10)
Southern Latin America	5.47 (4.02 − 7.25)	1404.11 (1037.67 − 1852.78)	1.00 (0.87 − 1.12)	136.35 (118.97 − 153.82)	0.57 (0.48 − 0.69)	74.53 (62.15 − 90.59)	0.25 (0.23 − 0.27)	28.96 (27.09 − 31.09)	0.59 (0.53 − 0.64)	73.93 (67.56 − 81.28)	1.19 (1.02 − 1.36)	303.87 (261.43 − 348.22)
Southern Sub-Saharan Africa	7.32 (5.45 − 9.98)	3166.50 (2375.95 − 4202.84)	0.75 (0.65 − 0.87)	91.92 (79.74 − 105.68)	0.20 (0.16 − 0.25)	25.85 (20.91 − 32.17)	0.10 (0.09 − 0.12)	16.17 (14.36 − 18.12)	0.05 (0.05 − 0.06)	7.44 (6.60 − 8.21)	0.59 (0.45 − 0.70)	251.77 (193.09 − 298.10)
Tropical Latin America	21.71 (18.13 − 26.56)	1892.15 (1570.65 − 2306.74)	6.00 (5.39 − 6.67)	248.35 (223.35 − 276.03)	1.02 (0.86 − 1.20)	39.15 (33.12 − 46.05)	0.45 (0.42 − 0.47)	17.14 (16.09 − 17.96)	0.36 (0.34 − 0.38)	13.99 (13.22 − 14.70)	3.11 (2.93 − 3.29)	270.67 (254.1 − 286.45)

Location	BPH		UTI		Urolithiasis		Bladder cancer		Kidney cancer		Prostate cancer	
	**Prevalence (× 10^5, 95% UI)**	**ASPR (1/100,000, 95% UI)**	**Prevalence (× 10^5, 95% UI)**	**ASPR (1/100,000, 95% UI)**	**Prevalence (× 10^5, 95% UI)**	**ASPR (1/100,000, 95% UI)**	**Prevalences (× 10^5, 95% UI)**	**ASPR (1/100,000, 95% UI)**	**Prevalence (× 10^5, 95% UI)**	**ASPR (1/100,000, 95% UI)**	**Prevalence (× 10^5, 95% UI)**	**ASPR (1/100,000, 95% UI)**
Western Europe	94.36 (77.31 − 115.9)	2269.19 (1864.75 − 2771.65)	6.16 (5.49 − 6.92)	139.14 (122.22 − 158.31)	3.11 (2.56 − 3.77)	51.77 (43.09 − 62.45)	7.09 (6.56 − 7.53)	78.49 (73.31 − 83.13)	4.11 (3.85 − 4.36)	54.33 (51.15 − 57.62)	26.70 (24.6 − 28.49)	625.03 (577.47 − 667.95)
Western Sub-Saharan Africa	18.89 (13.94 − 25.54)	2210.79 (1638.32 − 2941.62)	3.32 (2.91 − 3.77)	82.07 (72.23 − 92.29)	0.79 (0.63 − 0.98)	23.01 (18.72 − 28.50)	0.16 (0.13 − 0.19)	7.34 (6.17 − 8.81)	0.16 (0.10 − 0.21)	3.40 (2.46 − 4.30)	1.35 (0.71 − 1.85)	159.95 (86.08 − 215.29)

BPH benign prostatic hyperplasia, *UTI* urinary tract infections, *ASPR* age-standardized prevalence rate, *UI* uncertainty interval, *SDI* sociodemographic index

Table S4 Regional mortality and ASMR of the 6 urologic diseases in 2021

Location	BPH		UTI		Urolithiasis		Bladder cancer		Kidney cancer		Prostate cancer	
	**Mortality (× 10^5, 95% UI)**	**ASMR (1/100,000, 95% UI)**	**Mortality (× 10^5, 95% UI)**	**ASMR (1/100,000, 95% UI)**	**Mortality (× 10^5, 95% UI)**	**ASMR (1/100,000, 95% UI)**	**Mortality (× 10^5, 95% UI)**	**ASMR (1/100,000, 95% UI)**	**Mortality (× 105, 95% UI)**	**ASMR (1/100,000, 95% UI)**	**Mortality (× 10^5, 95% UI)**	**ASMR (1/100,000, 95% UI)**
Global	–	–	3.00 (2.68 − 3.24)	3.71 (3.31 − 4.01)	0.18 (0.14 − 0.21)	0.21 (0.17 − 0.25)	2.22 (2.01 − 2.42)	2.68 (2.42 − 2.93)	1.61 (1.50 − 1.69)	1.91 (1.78 − 2.01)	4.32 (3.82 − 4.64)	12.63 (11.16 − 13.55)
High SDI	–	–	0.72 (0.60 − 0.80)	2.85 (2.42 − 3.11)	0.03 (0.03 − 0.04)	0.14 (0.12 − 0.16)	0.81 (0.72 − 0.87)	3.40 (3.03 − 3.61)	0.65 (0.59 − 0.69)	2.98 (2.74 − 3.12)	1.54 (1.39 − 1.64)	15.35 (13.80 − 16.27)
High-middle SDI	–	–	0.49 (0.44 − 0.53)	2.60 (2.32 − 2.81)	0.04 (0.03 − 0.05)	0.20 (0.18 − 0.24)	0.64 (0.58 − 0.71)	3.25 (2.94 − 3.60)	0.47 (0.44 − 0.50)	2.39 (2.23 − 2.55)	0.90 (0.80 − 0.99)	11.73 (10.33 − 12.86)
Middle SDI	–	–	0.81 (0.73 − 0.89)	3.57 (3.15 − 3.90)	0.05 (0.04 − 0.07)	0.22 (0.15 − 0.27)	0.46 (0.40 − 0.53)	1.89 (1.65 − 2.20)	0.32 (0.29 − 0.35)	1.22 (1.12 − 1.32)	1.00 (0.83 − 1.16)	10.03 (8.30 − 11.60)
Low-middle SDI	–	–	0.73 (0.61 − 0.82)	5.68 (4.76 − 6.38)	0.04 (0.03 − 0.05)	0.29 (0.18 − 0.40)	0.21 (0.19 − 0.28)	1.68 (1.48 − 2.21)	0.12 (0.11 − 0.13)	0.86 (0.79 − 0.93)	0.58 (0.47 − 0.68)	10.92 (9.02 − 12.93)
Low SDI	–	–	0.25 (0.21 − 0.29)	5.17 (4.38 − 5.97)	0.01 (0.01 − 0.02)	0.21 (0.12 − 0.42)	0.09 (0.08 − 0.11)	2.26 (1.97 − 2.63)	0.05 (0.04 − 0.06)	0.85 (0.65 − 1.04)	0.29 (0.19 − 0.36)	16.80 (10.86 − 20.59)
Andean Latin America	–	–	0.03 (0.02 − 0.03)	4.77 (3.47 − 5.98)	0.00 (0.00 − 0.00)	0.07 (0.04 − 0.10)	0.01 (0.01 − 0.01)	1.52 (1.23 − 1.87)	0.01 (0.01 − 0.02)	2.08 (1.67 − 2.56)	0.06 (0.04 − 0.07)	22.26 (16.58 − 29.52)
Australasia	–	–	0.02 (0.01 − 0.02)	2.47 (2.03 − 2.76)	0.00 (0.00 − 0.00)	0.14 (0.12 − 0.16)	0.02 (0.01 − 0.02)	2.78 (2.46 − 3.04)	0.02 (0.01 − 0.02)	2.87 (2.51 − 3.22)	0.05 (0.04 − 0.06)	19.76 (16.71 − 22.70)
Caribbean	–	–	0.02 (0.01 − 0.02)	3.14 (2.67 − 3.68)	0.00 (0.00 − 0.00)	0.21 (0.18 − 0.25)	0.02 (0.01 − 0.02)	2.81 (2.48 − 3.13)	0.01 (0.01 − 0.01)	1.67 (1.46 − 1.87)	0.09 (0.08 − 0.11)	38.35 (32.98 − 44.63)
Central Asia	–	–	0.03 (0.02 − 0.03)	3.16 (2.80 − 3.50)	0.00 (0.00 − 0.00)	0.39 (0.31 − 0.48)	0.01 (0.01 − 0.02)	2.01 (1.79 − 2.23)	0.02 (0.02 − 0.02)	2.25 (1.95 − 2.52)	0.02 (0.02 − 0.03)	9.12 (8.32 − 10.01)
Central Europe	–	–	0.07 (0.06 − 0.07)	2.88 (2.56 − 3.24)	0.00 (0.00 − 0.00)	0.07 (0.06 − 0.09)	0.13 (0.12 − 0.14)	5.40 (4.97 − 5.79)	0.09 (0.09 − 0.10)	4.22 (3.87 − 4.56)	0.20 (0.18 − 0.21)	21.49 (19.56 − 23.19)
Central Latin America	–	–	0.15 (0.13 − 0.17)	6.25 (5.60 − 7.03)	0.01 (0.00 − 0.01)	0.22 (0.19 − 0.25)	0.03 (0.03 − 0.04)	1.38 (1.22 − 1.52)	0.06 (0.06 − 0.07)	2.46 (2.21 − 2.73)	0.20 (0.17 − 0.23)	19.52 (17.03 − 21.95)
Central Sub-Saharan Africa	–	–	0.01 (0.01 − 0.02)	2.28 (1.28 − 3.95)	0.00 (0.00 − 0.00)	0.09 (0.04 − 0.20)	0.01 (0.01 − 0.01)	2.64 (2.04 − 3.41)	0.00 (0.00 − 0.01)	0.74 (0.45 − 1.18)	0.04 (0.02 − 0.05)	27.70 (16.75 − 38.72)

Location	BPH		UTI		Urolithiasis		Bladder cancer		Kidney cancer		Prostate cancer	
	**Mortality (× 10^5, 95% UI)**	**ASMR (1/100,000, 95% UI)**	**Mortality (× 10^5, 95% UI)**	**ASMR (1/100,000, 95% UI)**	**Mortality (× 10^5, 95% UI)**	**ASMR (1/100,000, 95% UI)**	**Mortality (× 10^5, 95% UI)**	**ASMR (1/100,000, 95% UI)**	**Mortality (× 105, 95% UI)**	**ASMR (1/100,000, 95% UI)**	**Mortality (× 10^5, 95% UI)**	**ASMR (1/100,000, 95% UI)**
East Asia	−	−	0.12 (0.10 − 0.17)	0.70 (0.57 − 0.94)	0.03 (0.02 − 0.04)	0.15 (0.10 − 0.21)	0.47 (0.38 − 0.59)	2.35 (1.91 − 2.93)	0.26 (0.22 − 0.31)	1.27 (1.06 − 1.50)	0.40 (0.30 − 0.53)	5.05 (3.80 − 6.62)
Eastern Europe	−	−	0.17 (0.16 − 0.18)	4.89 (4.49 − 5.28)	0.02 (0.02 − 0.03)	0.62 (0.56 − 0.72)	0.10 (0.09 − 0.11)	2.86 (2.60 − 3.12)	0.14 (0.13 − 0.15)	3.97 (3.64 − 4.31)	0.22 (0.20 − 0.25)	18.39 (16.54 − 20.34)
Eastern Sub-Saharan Africa	−	−	0.13 (0.09 − 0.17)	7.82 (5.67 − 10.36)	0.00 (0.00 − 0.01)	0.25 (0.09 − 0.62)	0.04 (0.03 − 0.04)	2.68 (2.24 − 3.26)	0.03 (0.02 − 0.03)	1.31 (0.93 − 1.65)	0.11 (0.07 − 0.15)	20.00 (13.03 − 25.16)
High-income Asia Pacific	−	−	0.13 (0.10 − 0.15)	1.76 (1.41 − 2.01)	0.01 (0.01 − 0.01)	0.16 (0.13 − 0.18)	0.14 (0.11 − 0.15)	2.18 (1.88 − 2.39)	0.10 (0.09 − 0.11)	1.89 (1.68 − 2.02)	0.19 (0.17 − 0.20)	7.65 (6.80 − 8.23)
High-income North America	−	−	0.24 (0.20 − 0.26)	3.35 (2.87 − 3.61)	0.01 (0.01 − 0.01)	0.15 (0.13 − 0.16)	0.24 (0.21 − 0.26)	3.41 (3.04 − 3.62)	0.20 (0.18 − 0.21)	3.04 (2.78 − 3.17)	0.50 (0.45 − 0.52)	16.43 (14.7 − 17.43)
North Africa and Middle East	−	−	0.08 (0.07 − 0.09)	2.28 (1.94 − 2.71)	0.00 (0.00 − 0.01)	0.10 (0.05 − 0.13)	0.12 (0.10 − 0.15)	3.24 (2.75 − 3.91)	0.05 (0.05 − 0.06)	1.25 (1.11 − 1.43)	0.17 (0.12 − 0.20)	10.05 (7.09 − 12.18)
Oceania	−	−	0.00 (0.00 − 0.00)	2.3 (1.65 − 4.43)	0.00 (0.00 − 0.00)	0.01 (0 − 0.01)	0.00 (0.00 − 0.00)	1.38 (0.91 − 1.86)	0.00 (0.00 − 0.00)	0.41 (0.25 − 0.60)	0.01 (0.00 − 0.01)	21.76 (13.86 − 30.59)
South Asia	−	−	0.80 (0.66 − 0.90)	6.01 (5.00 − 6.77)	0.04 (0.02 − 0.06)	0.28 (0.16 − 0.43)	0.2 (0.17 − 0.24)	1.51 (1.31 − 1.88)	0.10 (0.09 − 0.11)	0.71 (0.64 − 0.78)	0.34 (0.28 − 0.46)	6.03 (4.98 − 8.26)
Southeast Asia	−	−	0.24 (0.18 − 0.28)	4.47 (3.3 − 5.27)	0.02 (0.01 − 0.03)	0.37 (0.16 − 0.47)	0.08 (0.07 − 0.10)	1.4 (1.19 − 1.80)	0.06 (0.06 − 0.07)	0.97 (0.86 − 1.08)	0.24 (0.16 − 0.30)	10.86 (7.10− 13.36)
Southern Latin America	−	−	0.08 (0.07 − 0.09)	8.75 (7.79 − 9.53)	0.00 (0.00 − 0.00)	0.05 (0.04 − 0.06)	0.03 (0.02 − 0.03)	2.94 (2.72 − 3.15)	0.04 (0.04 − 0.04)	4.55 (4.12 − 4.97)	0.08 (0.07 − 0.10)	23.26 (20.04 − 26.33)
Southern Sub-Saharan Africa	−	−	0.01 (0.01 − 0.01)	1.31 (1.06 − 1.79)	0.00 (0.00 − 0.00)	0.06 (0.04 − 0.09)	0.01 (0.01 − 0.02)	2.86 (2.55 − 3.18)	0.01 (0.01 − 0.01)	1.45 (1.31 − 1.56)	0.07 (0.05 − 0.09)	44.25 (31.48 − 51.79)
Tropical Latin America	−	−	0.28 (0.25 − 0.31)	11.74 (10.16 − 12.66)	0.01 (0.01 − 0.01)	0.27 (0.25 − 0.29)	0.06 (0.05 − 0.06)	2.38 (2.15 − 2.52)	0.05 (0.05 − 0.05)	2.02 (1.87 − 2.13)	0.22 (0.20 − 0.23)	21.62 (19.72 − 23.06)
Western Europe	−	−	0.37 (0.31 − 0.41)	2.96 (2.50 − 3.26)	0.01 (0.01 − 0.01)	0.10 (0.09 − 0.12)	0.48 (0.42 − 0.51)	4.29 (3.87 − 4.58)	0.33 (0.29 − 0.35)	3.28 (3.01 − 3.50)	0.86 (0.76 − 0.93)	18.36 (16.26 − 19.81)
Western Sub-Saharan Africa	−	−	0.04 (0.03 − 0.05)	1.66 (1.35 − 2.19)	0.00 (0.00 − 0.01)	0.18 (0.08 − 0.40)	0.03 (0.02 − 0.03)	1.83 (1.54 − 2.22)	0.02 (0.01 − 0.02)	0.72 (0.59 − 0.86)	0.25 (0.13 − 0.33)	38.56 (20.37 − 50.45)

BPH benign prostatic hyperplasia, *UTI* urinary tract infections, *ASMR* age-standardized mortality rate, *ASMR* age-standardized mortality rate

Table S5 Regional DALYs and ASDR of the 6 urologic diseases in 2021

Location	BPH		UTI		Urolithiasis		Bladder cancer		Kidney cancer		Prostate cancer	
	**DALYs ($\times 10^5$, 95% UI)**	**ASDR (1/100,000, 95% UI)**	**DALYs ($\times 10^5$, 95% UI)**	**ASDR (1/100,000, 95% UI)**	**DALYs ($\times 10^5$, 95% UI)**	**ASDR (1/100,000, 95% UI)**	**DALYs ($\times 10^5$, 95% UI)**	**ASDR (1/100,000, 95% UI)**	**DALYs ($\times 10^5$, 95% UI)**	**ASDR (1/100,000, 95% UI)**	**DALYs ($\times 10^5$, 95% UI)**	**ASDR (1/100,000, 95% UI)**
Global	22.36 (13.46 − 34.03)	55.12 (33.21 − 83.48)	68.48 (61.75 − 73.69)	83.74 (75.54 − 90.22)	6.93 (5.68 − 8.50)	8.15 (6.68 − 9.99)	43.97 (40.64 − 48.14)	51.58 (47.56 − 56.42)	40.16 (38.07 − 42.47)	47.33 (44.76 − 50.07)	81.42 (71.77 − 88.09)	217.83 (192.65 − 235.53)
High SDI	3.72 (2.34 − 5.59)	38.22 (24.15 − 57.34)	10.45 (9.20 − 11.28)	48.97 (44.46 − 52.63)	1.00 (0.84 − 1.20)	5.76 (4.69 − 7.10)	14.15 (12.92 − 15.00)	65.07 (60.13 − 68.71)	13.45 (12.60 − 14.08)	69.99 (66.59 − 73.07)	27.88 (25.62 − 29.85)	280.72 (257.96 − 300.71)
High-middle SDI	5.22 (3.12 − 7.92)	57.15 (34.03 − 86.02)	8.91 (8.25 − 9.53)	49.11 (45.46 − 52.54)	1.49 (1.26 − 1.83)	8.15 (6.78 − 10.11)	12.65 (11.57 − 14.02)	63.84 (58.43 − 70.77)	11.56 (10.79 − 12.35)	61.16 (57.09 − 65.23)	16.91 (15.01 − 18.56)	201.01 (179.17 − 220.19)
Middle SDI	7.70 (4.6 − 11.79)	61.38 (36.73 − 92.45)	18.85 (17.31 − 20.42)	75.4 (69.40 − 81.79)	2.30 (1.82 − 2.83)	8.45 (6.67 − 10.38)	9.88 (8.67 − 11.50)	37.63 (33.12 − 43.70)	9.02 (8.32 − 9.76)	33.47 (30.84 − 36.15)	19.07 (15.76 − 21.99)	168.77 (139.74 − 195.09)
Low-middle SDI	4.42 (2.64 − 6.85)	66.81 (39.95 − 102.81)	21.29 (17.58 − 23.92)	136.89 (113.75 − 153.21)	1.62 (1.18 − 2.14)	10.01 (7.24 − 13.33)	4.97 (4.38 − 6.40)	35.18 (30.99 − 45.55)	3.90 (3.49 − 4.35)	24.46 (22.12 − 27.02)	11.35 (9.10 − 13.42)	189.22 (153.60 − 223.29)
Low SDI	1.28 (0.77 − 1.99)	55.97 (33.22 − 87.23)	8.94 (7.64 − 10.42)	126.27 (108.07 − 145.81)	0.51 (0.35 − 0.78)	7.52 (5.04 − 12.32)	2.26 (1.95 − 2.64)	45.95 (39.78 − 53.52)	2.18 (1.50 − 2.87)	26.00 (18.96 − 32.50)	6.06 (3.86 − 7.55)	294.26 (189.52 − 363.32)
Andean Latin America	0.16 (0.09 − 0.25)	57.26 (33.77 − 90.69)	0.55 (0.41 − 0.69)	93.46 (69.59 − 115.80)	0.04 (0.03 − 0.06)	6.57 (4.91 − 8.96)	0.17 (0.14 − 0.21)	29.34 (23.63 − 36.18)	0.34 (0.27 − 0.42)	55.46 (44.65 − 68.78)	0.98 (0.72 − 1.32)	372.49 (275.16 − 500.95)
Australasia	0.10 (0.06 − 0.16)	39.12 (23.57 − 60.97)	0.21 (0.18 − 0.24)	39.04 (33.86 − 43.25)	0.03 (0.02 − 0.03)	6.15 (4.82 − 7.97)	0.28 (0.25 − 0.30)	49.59 (45.19 − 53.91)	0.33 (0.30 − 0.37)	67.67 (60.29 − 75.54)	0.94 (0.79 − 1.09)	354.41 (299.70 − 411.98)
Caribbean	0.15 (0.09 − 0.24)	60.73 (36.75 − 95.66)	0.36 (0.30 − 0.43)	69.67 (58.37 − 82.93)	0.05 (0.04 − 0.06)	8.92 (7.4 − 10.82)	0.30 (0.27 − 0.34)	55.98 (49.43 − 62.58)	0.25 (0.22 − 0.28)	48.57 (42.24 − 55.71)	1.68 (1.43 − 1.97)	680.84 (582.10 − 799.04)
Central Asia	0.19 (0.11 − 0.29)	56.48 (33.31 − 86.52)	0.88 (0.78 − 1.01)	97.55 (86.54 − 110.02)	0.11 (0.09 − 0.14)	12.85 (10.54 − 16.06)	0.36 (0.32 − 0.40)	44.37 (39.02 − 49.71)	0.58 (0.50 − 0.65)	63.85 (55.23 − 71.86)	0.54 (0.49 − 0.60)	174.31 (158.35 − 191.95)
Central Europe	0.62 (0.40 − 0.95)	63.6 (40.46 − 95.41)	1.17 (1.04 − 1.34)	56.66 (50.68 − 65.49)	0.08 (0.06 − 0.10)	4.42 (3.40 − 5.85)	2.53 (2.33 − 2.73)	112 (103.07 − 121.02)	2.12 (1.96 − 2.29)	102.79 (94.92 − 111.10)	3.65 (3.31 − 3.96)	380.69 (345.46 − 412.55)
Central Latin America	1.07 (0.67 − 1.62)	94.46 (58.99 − 143.91)	3.42 (3.04 − 3.94)	137.47 (122.27 − 158.39)	0.24 (0.20 − 0.28)	8.99 (7.76 − 10.78)	0.70 (0.63 − 0.78)	28.24 (25.22 − 31.21)	1.72 (1.54 − 1.93)	67.13 (59.97 − 75.34)	3.87 (3.34 − 4.43)	357.67 (310.25 − 408.84)
Central Sub-Saharan Africa	0.10 (0.06 − 0.15)	42.28 (25.35 − 66.35)	0.37 (0.24 − 0.59)	51.51 (30.57 − 85.97)	0.03 (0.02 − 0.05)	4.04 (2.49 − 6.81)	0.29 (0.22 − 0.37)	54.88 (42.29 − 70.31)	0.16 (0.10 − 0.24)	20.48 (12.55 − 31.70)	0.80 (0.49 − 1.10)	462.63 (277.39 − 637.06)
East Asia	4.84 (2.85 − 7.37)	46.78 (27.91 − 70.91)	2.62 (2.12 − 3.54)	14.07 (11.52 − 18.71)	1.17 (0.90 − 1.49)	5.72 (4.42 − 7.31)	9.70 (7.75 − 12.24)	45.62 (36.68 − 57.34)	7.00 (5.77 − 8.36)	34.77 (29.00 − 41.18)	7.29 (5.53 − 9.84)	79.68 (60.09 − 105.85)

Location	BPH		UTI		Urolithiasis		Bladder cancer		Kidney cancer		Prostate cancer	
	**DALYs (× 10^5, 95% UI)**	**ASDR (1/100,000, 95% UI)**	**DALYs (× 10^5, 95% UI)**	**ASDR (1/100,000, 95% UI)**	**DALYs (× 10^5, 95% UI)**	**ASDR (1/100,000, 95% UI)**	**DALYs (× 10^5, 95% UI)**	**ASDR (1/100,000, 95% UI)**	**DALYs (× 10^5, 95% UI)**	**ASDR (1/100,000, 95% UI)**	**DALYs (× 10^5, 95% UI)**	**ASDR (1/100,000, 95% UI)**
Eastern Europe	1.68 (1.01 − 2.54)	123.56 (75.40 − 185.08)	3.65 (3.33 − 3.98)	113.65 (104.13 − 123.67)	0.72 (0.62 − 0.88)	22.82 (19.42 − 28.00)	2.27 (2.04 − 2.48)	63.86 (57.69 − 70.02)	3.55 (3.24 − 3.88)	105.76 (96.57 − 115.64)	4.82 (4.29 − 5.40)	370.67 (332.05 − 413.25)
Eastern Sub-Saharan Africa	0.35 (0.21 − 0.55)	48.07 (28.63 − 75.84)	4.50 (3.39 − 5.65)	187.5 (138.51 − 244.83)	0.19 (0.10 − 0.37)	8.20 (4.01 − 17.03)	0.89 (0.73 − 1.12)	54.45 (44.88 − 67.50)	1.05 (0.65 − 1.44)	37.82 (25.44 − 49.07)	2.50 (1.60 − 3.24)	371.75 (238.72 − 471.99)
High-income Asia Pacific	0.48 (0.29 − 0.76)	23.39 (14.17 − 37.35)	1.53 (1.23 − 1.73)	28.94 (24.86 − 32.25)	0.24 (0.19 − 0.29)	6.29 (4.96 − 7.98)	2.03 (1.78 − 2.22)	39.27 (35.51 − 42.42)	1.74 (1.56 − 1.86)	41.24 (38.15 − 43.59)	3.00 (2.68 − 3.25)	128.79 (115.29 − 139.46)
High-income North America	1.08 (0.68 − 1.58)	35.53 (22.46 − 51.92)	3.84 (3.41 − 4.08)	59.54 (53.96 − 63.26)	0.32 (0.27 − 0.37)	5.67 (4.80 − 6.72)	4.52 (4.14 − 4.81)	67.31 (62.04 − 71.53)	4.53 (4.25 − 4.74)	74.46 (70.22 − 77.61)	10.05 (9.22 − 10.87)	327.02 (300.14 − 353.70)
North Africa and Middle East	0.94 (0.56 − 1.46)	42.84 (25.50 − 67.53)	1.79 (1.56 − 2.03)	41.34 (36.12 − 47.20)	0.25 (0.18 − 0.34)	4.55 (3.18 − 5.98)	2.88 (2.44 − 3.53)	66.27 (56.58 − 80.57)	1.65 (1.47 − 1.86)	32.81 (29.06 − 36.93)	3.28 (2.31 − 3.92)	173.58 (123.07 − 209.38)
Oceania	0.02 (0.01 − 0.04)	71.31 (43.08 − 107.33)	0.05 (0.03 − 0.08)	51.4 (36.69 − 96.56)	0.00 (0.00 − 0.00)	2.81 (1.91 − 4.04)	0.02 (0.02 − 0.03)	31.41 (20.31 − 43.08)	0.01 (0.01 − 0.02)	11.5 (7.14 − 16.86)	0.10 (0.07 − 0.15)	361.58 (229.77 − 512.13)
South Asia	5.17 (3.11 − 7.94)	73.7 (44.30 − 113.02)	23.97 (20.00 − 26.87)	152.64 (127.55 − 171.11)	1.66 (1.14 − 2.31)	10.08 (6.83 − 14.11)	4.44 (3.85 − 5.47)	30.84 (26.73 − 38.03)	3.05 (2.75 − 3.36)	19.46 (17.60 − 21.45)	6.65 (5.4 − 9.12)	104.26 (85.19 − 143.01)
Southeast Asia	2.46 (1.48 − 3.77)	83.47 (50.21 − 127.59)	6.04 (4.46 − 6.97)	94.51 (69.7 − 109.30)	0.89 (0.54 − 1.12)	12.67 (7.66 − 15.82)	1.82 (1.55 − 2.32)	28.60 (24.36 − 36.71)	1.95 (1.72 − 2.17)	28.03 (24.72 − 31.24)	4.92 (3.25 − 6.01)	190.74 (125.11 − 231.98)
Southern Latin America	0.11 (0.07 − 0.17)	27.98 (16.88 − 43.59)	1.26 (1.16 − 1.36)	143.61 (132.95 − 154.22)	0.05 (0.04 − 0.07)	6.56 (4.68 − 9.07)	0.52 (0.48 − 0.56)	58.96 (54.88 − 63.13)	1.00 (0.91 − 1.09)	120.33 (109.84 − 130.97)	1.49 (1.28 − 1.70)	394.12 (340.04 − 452.15)
Southern Sub-Saharan Africa	0.15 (0.09 − 0.22)	62.47 (37.46 − 96.99)	0.25 (0.21 − 0.32)	36.51 (29.99 − 47.17)	0.03 (0.02 − 0.03)	3.54 (2.71 − 4.73)	0.37 (0.32 − 0.42)	63.23 (55.53 − 71.44)	0.26 (0.24 − 0.29)	40.36 (36.28 − 44.41)	1.55 (1.15 − 1.85)	774.40 (563.55 − 905.89)
Tropical Latin America	0.43 (0.27 − 0.65)	37.17 (23.56 − 56.27)	5.33 (4.88 − 5.64)	217.07 (198.1 − 229.94)	0.27 (0.24 − 0.30)	10.38 (9.35 − 11.69)	1.20 (1.12 − 1.27)	47.22 (43.72 − 49.70)	1.38 (1.31 − 1.45)	54.17 (51.10 − 56.95)	4.06 (3.79 − 4.31)	376.24 (349.02 − 400.47)
Western Europe	1.89 (1.20 − 2.86)	45.51 (28.90 − 68.63)	4.74 (4.11 − 5.17)	46.06 (41.32 − 50.38)	0.40 (0.32 − 0.49)	5.63 (4.37 − 7.25)	7.96 (7.23 − 8.51)	81.34 (74.89 − 86.68)	6.39 (5.94 − 6.79)	75.82 (71.42 − 80.46)	14.43 (12.92 − 15.69)	319.95 (287.64 − 347.77)
Western Sub-Saharan Africa	0.38 (0.22 − 0.59)	44.09 (25.96 − 69.48)	1.94 (1.58 − 2.35)	49.46 (40.69 − 60.05)	0.18 (0.11 − 0.33)	6.14 (3.61 − 11.53)	0.71 (0.59 − 0.86)	36.83 (30.85 − 44.15)	1.08 (0.73 − 1.41)	24.32 (18.31 − 30.31)	4.81 (2.52 − 6.51)	646.45 (343.4 − 860.10)

BPH benign prostatic hyperplasia, *UTI* urinary tract infections, *DALYs* disability-adjusted life-years, *ASDR* age-standardized DALYs rate

Table S6 Age-standardized incidence, prevalence, mortality, and DALYs rates of the 6 urologic diseases among the top 3 and bottom 3 countries in 2021

**Measure**	**Type**	**Sex**	**Top 3 countries**			**Bottom 3 countries**		
ASIR (1/100,000)	BPH	Male	Lithuania (691.36)	Ukraine (666.49)	Russian Federation (663.09)	Brunei Darussalam (137.05)	Singapore (127.13)	Republic of Korea (126.90)
	UTI	Both	Ecuador (15,136.70)	Paraguay (13,326.42)	Brazil (13,014.71)	Taiwan (Province of China) (1423.67)	Democratic People's Republic of Korea (1214.56)	China (1184.13)
		Male	Ukraine (7187.51)	Paraguay (6049.19)	Russian Federation (5652.30)	Taiwan (Province of China) (423.35)	Democratic People's Republic of Korea (371.20)	China (322.16)
		Female	Ecuador (26,078.77)	Brazil (21,070.28)	Paraguay (20,810.84)	Taiwan (Province of China) (2415.84)	China (2084.82)	Democratic People's Republic of Korea (2075.34)
	Urolithiasis	Both	Ukraine (3766.92)	Russian Federation (3525.62)	Belarus (3516.81)	Chad (576.03)	Guinea (574.74)	Niger (573.14)
		Male	Ukraine (5821.66)	Belarus (5411.22)	Latvia (5371.32)	South Sudan (687.52)	Eritrea (684.54)	Somalia (681.47)
		Female	Russian Federation (2157.21)	Ukraine (2075.01)	Chile (2011.66)	Democratic Republic of the Congo (410.00)	Angola (405.23)	Central African Republic (404.74)
	Bladder cancer	Both	Lebanon (21.66)	Italy (18.52)	Greece (16.60)	Nigeria (0.95)	Kiribati (0.78)	Albania (0.58)
		Male	Lebanon (39.87)	Italy (33.31)	Spain (31.48)	Nigeria (1.15)	Albania (0.87)	Kiribati (0.68)

Measure	Type	Sex	Top 3 countries			Bottom 3 countries		
		Female	Malawi (8.68)	United Arab Emirates (8.29)	Monaco (8.27)	Albania (0.32)	Palau (0.13)	Samoa (0.07)
	Kidney cancer	Both	Argentina (15.60)	Czechia (14.69)	Latvia (14.23)	Morocco (0.52)	Papua New Guinea (0.51)	Republic of Côte d'Ivoire (0.47)
		Male	Argentina (23.79)	Latvia (21.37)	Estonia (21.30)	Gambia (0.66)	Republic of Côte d'Ivoire (0.65)	Morocco (0.54)
		Female	Iceland (10.59)	Monaco (10.54)	United Arab Emirates (10.21)	Palau (0.10)	Sao Tome and Principe (0.09)	Kiribati (0.06)
	Prostate cancer	Male	Bermuda (196.12)	Antigua and Barbuda (169.71)	Saint Kitts and Nevis (168.23)	Nepal (6.15)	Mongolia (6.05)	Tajikistan (5.03)
ASPR (1/100,000)	BPH	Male	Lithuania (6719.37)	Ukraine (6314.19)	Russian Federation (6279.89)	Brunei Darussalam (1118.07)	Singapore (1027.45)	Republic of Korea (1025.00)
	UTI	Both	Ecuador (288.01)	Paraguay (253.94)	Brazil (248.24)	Taiwan (Province of China) (27.11)	Democratic People's Republic of Korea (23.11)	China (22.52)
		Male	Ukraine (137.68)	Paraguay (114.75)	Russian Federation (108.39)	Taiwan (Province of China) (8.04)	Democratic People's Republic of Korea (7.03)	China (6.10)
		Female	Ecuador (496.28)	Brazil (402.36)	Paraguay (397.01)	Taiwan (Province of China) (45.99)	China (39.65)	Democratic People's Republic of Korea (39.49)
	Urolithiasis	Both	Ukraine (142.62)	Russian Federation (133.33)	Belarus (132.93)	Chad (21.88)	Guinea (21.83)	Niger (21.77)

Measure	Type	Sex	Top 3 countries			Bottom 3 countries		
		Male	Ukraine (220.64)	Belarus (204.68)	Latvia (202.96)	South Sudan (26.14)	Eritrea (26.02)	Somalia (25.91)
		Female	Russian Federation (81.71)	Ukraine (78.73)	Chile (76.15)	Democratic Republic of the Congo (15.58)	Angola (15.40)	Central African Republic (15.37)
	Bladder cancer	Both	Lebanon (130.22)	Italy (125.66)	Spain (108.68)	Albania (3.34)	Kiribati (2.85)	Nigeria (2.58)
		Male	Lebanon (246.10)	Italy (219.62)	Spain (203.04)	Honduras (4.63)	Nigeria (3.16)	Kiribati (2.66)
		Female	Czechia (52.21)	United States of America (49.65)	Monaco (49.40)	Albania (1.95)	Palau (0.61)	Samoa (0.38)
	Kidney cancer	Both	Argentina (89.01)	France (77.21)	Czechia (75.69)	Papua New Guinea (1.87)	Niger (1.85)	Republic of Côte d'Ivoire (1.62)
		Male	Argentina (140.07)	France (105.07)	Estonia (104.63)	Niger (2.46)	Gambia (2.14)	Republic of Côte d'Ivoire (2.12)
		Female	Iceland (60.71)	Monaco (60.17)	Czechia (53.92)	Palau (0.43)	Sao Tome and Principe (0.33)	Kiribati (0.21)
	Prostate cancer	Male	Bermuda (1527.67)	Antigua and Barbuda (1115.17)	Estonia (1101.36)	Bhutan (32.06)	Tajikistan (29.24)	Nepal (27.04)
ASMR (1/100,000)	BPH	Male	–	–	–	–	–	–
	UTI	Both	Barbados (12.96)	Brazil (11.96)	Uruguay (10.75)	North Macedonia (0.22)	Cook Islands (0.19)	Montenegro (0.16)

Measure	Type	Sex	Top 3 countries			Bottom 3 countries		
		Male	Saint Kitts and Nevis (18.16)	Barbados (17.09)	Honduras (12.71)	North Macedonia (0.25)	Montenegro (0.22)	Cook Islands (0.16)
		Female	Ethiopia (12.94)	Brazil (11.94)	Pakistan (10.41)	Cook Islands (0.20)	North Macedonia (0.20)	Montenegro (0.13)
	Urolithiasis	Both	Kazakhstan (1.29)	Trinidad and Tobago (0.91)	Russian Federation (0.78)	Northern Mariana Islands (0)	Romania (0)	Uzbekistan (0)
		Male	Trinidad and Tobago (1.47)	Kazakhstan (1.43)	Philippines (0.99)	Uzbekistan (0)	Montenegro (0)	United Arab Emirates (0)
		Female	Kazakhstan (1.20)	Russian Federation (0.72)	Belarus (0.63)	Maldives (0)	Romania (0)	Uzbekistan (0)
	Bladder cancer	Both	Mali (8.99)	Malawi (8.60)	Zimbabwe (7.84)	Kiribati (0.58)	Palau (0.58)	Albania (0.30)
		Male	Poland (13.57)	Lebanon (13.34)	Mali (12.91)	Nicaragua (0.81)	Kiribati (0.48)	Albania (0.48)
		Female	Malawi (6.87)	Zimbabwe (6.46)	United Arab Emirates (5.40)	Albania (0.16)	Palau (0.08)	Samoa (0.04)
	Kidney cancer	Both	Uruguay (6.47)	Czechia (5.95)	Lithuania (5.25)	Palau (0.30)	Morocco (0.27)	Cook Islands (0.24)
		Male	Uruguay (10.27)	Lithuania (8.86)	Czechia (8.85)	Republic of Côte d'Ivoire (0.48)	Cook Islands (0.44)	Morocco (0.37)
		Female	United Arab Emirates (4.80)	Czechia (3.72)	Uruguay (3.66)	Cook Islands (0.05)	Palau (0.05)	Kiribati (0.04)

Measure	Type	Sex	Top 3 countries			Bottom 3 countries		
	Prostate cancer	Male	Grenada (93.90)	Saint Kitts and Nevis (93.85)	Saint Vincent and the Grenadines (82.13)	Tajikistan (3.77)	Viet Nam (3.72)	Algeria (2.88)
ASDR (1/100,000)	BPH	Male	Lithuania (132.83)	Ukraine (124.78)	Russian Federation (123.78)	Brunei Darussalam (22.07)	Singapore (20.66)	Republic of Korea (20.36)
	UTI	Both	Turkmenistan (298.01)	Barbados (239.18)	Ethiopia (226.24)	North Macedonia (6.66)	Montenegro (5.75)	Cook Islands (4.83)
		Male	Turkmenistan (333.44)	Saint Kitts and Nevis (318.74)	Barbados (295.62)	North Macedonia (5.42)	Montenegro (4.85)	Cook Islands (3.54)
		Female	Ethiopia (319.45)	Turkmenistan (275.77)	Pakistan (272.47)	North Macedonia (8.08)	Montenegro (6.91)	Cook Islands (5.94)
	Urolithiasis	Both	Kazakhstan (33.34)	Trinidad and Tobago (30.14)	Russian Federation (25.57)	Guam (2.77)	Saudi Arabia (2.73)	Papua New Guinea (2.73)
		Male	Trinidad and Tobago (45.15)	Kazakhstan (41.00)	Philippines (34.59)	Romania (3.25)	Costa Rica (3.20)	Montenegro (3.16)
		Female	Kazakhstan (27.60)	Russian Federation (20.90)	Belarus (20.10)	Kuwait (1.73)	Saudi Arabia (1.61)	Palestine (1.50)
	Bladder cancer	Both	Malawi (179.92)	Mali (176.81)	Zimbabwe (169.21)	Kiribati (13.40)	Nicaragua (12.78)	Albania (5.75)
		Male	Poland (255.26)	Lebanon (252.37)	Mali (249.42)	Nicaragua (16.48)	Kiribati (11.09)	Albania (8.75)
		Female	Malawi (142.83)	Zimbabwe (139.87)	Mali (99.33)	Albania (3.15)	Palau (1.65)	Samoa (1.00)

Measure	Type	Sex	Top 3 countries			Bottom 3 countries		
	Kidney cancer	Both	Uruguay (170.16)	Czechia (139.52)	Lithuania (135.35)	Palau (8.71)	Morocco (7.10)	Cook Islands (6.31)
		Male	Uruguay (261.61)	Lithuania (223.93)	Latvia (210.07)	Samoa (13.17)	Cook Islands (11.54)	Morocco (9.36)
		Female	Uruguay (95.87)	United Arab Emirates (93.62)	Monaco (86.31)	Cook Islands (1.40)	Palau (1.33)	Kiribati (1.20)
	Prostate cancer	Male	Grenada (1542.79)	Saint Kitts and Nevis (1517.58)	Saint Vincent and the Grenadines (1325.19)	China (76.97)	Viet Nam (64.20)	Algeria (46.25)

BPH benign prostatic hyperplasia, *UTI* urinary tract infections, *ASIR* age-standardized incidence rate, *ASPR* age-standardized prevalence rate, *ASMR* age-standardized mortality rate, *ASDR* age-standardized DALYs rate

Table S7 EAPC of ASIR for the 6 urologic diseases in 204 countries and territories from 1990 to 2021 (95% CI)

Location	BPH	UTI	Urolithiasis	Bladder cancer	Kidney cancer	Prostate cancer
Afghanistan	0.14 (0.13 − 0.15)	-0.03 (-0.06 to -0.01)	0.19 (0.16 − 0.23)	-0.02 (-0.07 to 0.02)	1.46 (1.16 − 1.76)	1.25 (1.14 − 1.36)
Albania	0.03 (-0.04 to 0.09)	0.06 (0.04 − 0.08)	-0.19 (-0.25 to -0.13)	1.00 (0.82 − 1.19)	2.65 (2.33 − 2.98)	1.31 (1.16 − 1.45)
Algeria	0.12 (0.11 − 0.14)	0.09 (0.07 − 0.10)	0.22 (0.17 − 0.27)	0.73 (0.56 to 0.89)	2.55 (2.42 − 2.68)	1.87 (1.78 − 1.96)
American Samoa	0.19 (0.16 − 0.21)	0.14 (0.12 − 0.16)	-0.30 (-0.35 to -0.25)	2.44 (2.08 − 2.81)	1.24 (0.96 − 1.52)	0.91 (0.73 − 1.09)
Andorra	0.43 (0.30 − 0.56)	0.17 (0.12 − 0.21)	-0.15 (-0.16 to -0.13)	-0.65 (-0.90 to -0.40)	0.40 (0.19 − 0.62)	1.07 (0.76 − 1.37)
Angola	0.04 (0.03 − 0.06)	0.22 (0.21 − 0.23)	-0.01 (-0.03 to 0.01)	0.15 (0.02 − 0.28)	1.20 (1.07 − 1.34)	1.16 (1.06 − 1.26)
Antigua and Barbuda	0.23 (0.21 − 0.26)	0.00 (-0.04 to 0.03)	0.42 (0.36 − 0.47)	0.69 (0.44 − 0.94)	1.01 (0.83 − 1.18)	0.65 (0.26 − 1.04)
Argentina	0.30 (0.20 − 0.41)	0.19 (0.17 − 0.20)	-0.13 (-0.19 to -0.06)	-0.88 (-1.01 to -0.76)	1.45 (1.25 − 1.65)	0.47 (0.05 − 0.90)
Armenia	0.04 (0.00 − 0.07)	0.42 (0.32 − 0.52)	1.29 (1.19 to 1.39)	0.73 (0.48 − 0.97)	1.97 (1.65 − 2.29)	2.84 (2.59 − 3.08)
Australia	0.19 (0.10 − 0.27)	0.01 (0.00 − 0.02)	-0.40 (-0.47 to -0.34)	-1.02 (-1.11 to -0.93)	0.95 (0.71 to 1.20)	-0.82 (-1.55 to -0.09)
Austria	0.72 (0.61 − 0.83)	0.46 (0.29 − 0.63)	-1.91 (-2.33 to -1.49)	-0.21 (-0.31 to -0.11)	-0.14 (-0.30 to 0.02)	-0.04 (-0.42 to 0.34)
Azerbaijan	0.05 (0.02 − 0.07)	0.11 (0.08 − 0.14)	0.08 (0.02 − 0.14)	0.07 (-0.09 − 0.22)	1.25 (1.05 − 1.45)	0.88 (0.65 − 1.11)
Bahrain	0.20 (0.18 − 0.21)	-0.08 (-0.10 to -0.05)	0.42 (0.38 − 0.47)	-0.61 (-0.85 to -0.37)	0.55 (0.34 − 0.76)	1.91 (1.76 − 2.07)
Bangladesh	0.12 (0.11 − 0.12)	0.23 (0.20 − 0.26)	0.27 (0.21 − 0.32)	-0.48 (-0.66 to -0.31)	0.75 (0.68 − 0.81)	0.41 (0.28 − 0.55)
Barbados	0.20 (0.18 − 0.23)	0.11 (0.10 − 0.12)	0.69 (0.65 − 0.73)	0.76 (0.60 − 0.93)	1.29 (1.13 − 1.46)	0.74 (0.57 − 0.91)

Location	BPH	UTI	Urolithiasis	Bladder cancer	Kidney cancer	Prostate cancer
Belarus	-0.03 (-0.05 to 0.00)	0.00 (-0.01 to 0.02)	0.08 (0.05 − 0.11)	-0.66 (-0.99 to -0.32)	3.52 (2.76 − 4.29)	3.37 (3.06 − 3.68)
Belgium	0.44 (0.29 − 0.58)	-0.03 (-0.11 to 0.05)	0.72 (-0.34 − 1.8)	-0.70 (-0.93 to -0.47)	0.37 (0.07 − 0.67)	-0.79 (-1.17 to -0.4)
Belize	0.24 (0.21 − 0.27)	0.20 (0.19 − 0.21)	0.62 (0.56 − 0.69)	1.21 (0.83 − 1.58)	1.55 (1.21 − 1.90)	1.62 (0.91 − 2.33)
Benin	0.05 (0.03 − 0.06)	0.03 (0.01 − 0.05)	0.10 (0.05 − 0.14)	-0.88 (-1.01 to -0.75)	2.33 (2.21 − 2.45)	2.07 (1.96 − 2.17)
Bermuda	0.19 (0.17 − 0.21)	0.02 (0.00 − 0.04)	0.40 (0.32 − 0.47)	0.20 (-0.01 − 0.41)	0.31 (0.15 − 0.48)	1.38 (1.09 − 1.68)
Bhutan	0.13 (0.12 − 0.14)	0.16 (0.14 − 0.18)	0.30 (0.25 − 0.35)	0.28 (0.17 − 0.40)	1.66 (1.62 − 1.70)	0.93 (0.83 − 1.03)
Bolivarian Republic of Venezuela	0.18 (0.17 − 0.20)	-0.01 (-0.03 to 0.01)	0.37 (0.30 − 0.43)	0.31 (0.17 − 0.45)	1.17 (0.91 − 1.43)	1.93 (1.45 − 2.41)
Bosnia and Herzegovina	0.12 (0.05 − 0.18)	-0.03 (-0.05 to -0.02)	-0.14 (-0.22 to -0.05)	2.04 (1.81 − 2.27)	3.58 (3.19 − 3.98)	2.88 (2.59 − 3.16)
Botswana	0.09 (0.08 − 0.11)	0.54 (-0.14 − 1.22)	0.25 (0.14 − 0.36)	-0.52 (-0.65 to -0.39)	1.89 (1.75 − 2.04)	0.78 (0.57 − 1.00)
Brazil	-0.26 (-0.37 to -0.14)	0.00 (-0.09 to 0.08)	1.61 (1.15 − 2.08)	0.27 (0.19 − 0.35)	1.85 (1.73 − 1.97)	0.92 (0.56 − 1.28)
Brunei Darussalam	0.16 (0.10 to 0.21)	0.09 (0.06 − 0.11)	-0.22 (-0.27 to -0.18)	0.07 (-0.13 to 0.27)	1.26 (1.09 − 1.42)	1.60 (1.41 − 1.78)
Bulgaria	0.04 (-0.03 to 0.11)	0.02 (-0.14 − 0.18)	-1.67 (-1.9 to -1.44)	1.83 (1.72 − 1.94)	4.71 (4.28 − 5.13)	2.60 (2.45 − 2.75)
Burkina Faso	0.04 (0.03 − 0.04)	0.07 (0.06 − 0.09)	0.12 (0.08 − 0.16)	-0.80 (-0.93 to -0.67)	2.38 (2.28 − 2.48)	2.17 (2.06 − 2.28)
Burundi	-0.03 (-0.04 to -0.01)	-0.06 (-0.09 to -0.04)	-0.09 (-0.13 to -0.05)	-1.21 (-1.37 to -1.06)	-0.10 (-0.18 to -0.01)	-0.19 (-0.28 to -0.1)
Cambodia	0.07 (0.05 − 0.09)	0.07 (0.06 − 0.09)	0.18 (0.17 − 0.20)	0.53 (0.33 − 0.74)	1.5 (1.35 − 1.66)	1.97 (1.86 − 2.08)

Location	BPH	UTI	Urolithiasis	Bladder cancer	Kidney cancer	Prostate cancer
Cameroon	0.04 (0.04 − 0.05)	0.00 (-0.02 to 0.02)	0.05 (0.01 − 0.08)	-0.41 (-0.53 to -0.30)	2.26 (2.15 − 2.38)	2.08 (2.01 − 2.16)
Canada	0.31 (0.18 − 0.43)	-0.05 (-0.07 to -0.03)	0.22 (0.20 − 0.23)	-1.01 (-1.21 to -0.81)	0.95 (0.64 − 1.26)	-2.22 (-2.56 to -1.87)
Central African Republic	0.03 (0.02 − 0.05)	0.09 (0.06 to 0.12)	0.35 (0.33 − 0.37)	-0.47 (-0.52 to -0.42)	0.23 (0.19 − 0.26)	0.45 (0.42 − 0.47)
Chad	0.03 (0.01 − 0.04)	-0.05 (-0.08 to -0.02)	0.08 (0.03 − 0.13)	0.16 (0.01 − 0.32)	2.85 (2.72 − 2.98)	2.45 (2.30 − 2.61)
Chile	0.33 (0.23 − 0.43)	-0.15 (-0.43 to 0.13)	0.93 (0.52 − 1.35)	0.37 (0.24 − 0.50)	1.45 (1.32 − 1.58)	1.76 (1.40 − 2.12)
China	-0.21 (-0.36 to -0.06)	-0.24 (-0.31 to -0.18)	-2.37 (-2.55 to -2.18)	0.12 (0.02 − 0.22)	2.39 (2.19 − 2.59)	1.83 (1.71 − 1.96)
Colombia	0.17 (0.15 − 0.18)	0.08 (0.07 − 0.09)	-0.02 (-0.05 to 0.01)	-0.59 (-0.76 to -0.42)	1.48 (1.38 − 1.57)	1.04 (0.70 − 1.39)
Commonwealth of the Bahamas	0.20 (0.18 − 0.23)	0.14 (0.01 − 0.17)	0.38 (0.31 − 0.46)	0.91 (0.76 − 1.05)	1.14 (0.99 − 1.29)	0.97 (0.68 − 1.27)
Comoros	-0.02 (-0.03 to 0.00)	0.07 (0.05 − 0.08)	0.08 (0.05 − 0.12)	-0.22 (-0.32 to -0.12)	0.90 (0.80 − 0.99)	0.66 (0.62 − 0.70)
Congo	0.02 (0.01 − 0.04)	0.05 (0.04 − 0.06)	0.22 (0.19 − 0.25)	-0.25 (-0.43 to -0.06)	1.17 (1.02 − 1.31)	0.61 (0.49 − 0.73)
Cook Islands	0.13 (0.11 − 0.15)	0.28 (0.27 − 0.30)	0.06 (0.00 − 0.11)	1.05 (0.96 − 1.14)	-0.16 (-0.38 to 0.06)	1.16 (1.03 − 1.29)
Costa Rica	0.21 (0.19 − 0.22)	0.04 (0.03 − 0.05)	-0.22 (-0.28 to -0.16)	-0.21 (-0.35 to -0.07)	2.77 (2.61 − 2.92)	2.38 (2.01 − 2.75)
Croatia	-0.04 (-0.09 to 0.00)	0.45 (0.21 − 0.69)	-0.04 (-0.46 to 0.38)	1.34 (1.19 − 1.49)	1.64 (1.33 − 1.96)	1.39 (1.14 − 1.64)
Cuba	0.19 (0.17 − 0.22)	-0.02 (-0.03 to -0.01)	1.20 (1.14 − 1.26)	1.05 (0.91 − 1.20)	1.18 (1.07 − 1.28)	2.07 (1.95 − 2.18)
Cyprus	0.25 (0.14 − 0.35)	-0.30 (-0.69 to 0.10)	-0.74 (-1.32 to -0.16)	0.28 (0.09 − 0.47)	2.15 (1.80 − 2.49)	1.41 (1.08 − 1.74)

Location	BPH	UTI	Urolithiasis	Bladder cancer	Kidney cancer	Prostate cancer
Czech Republic	0.14 (0.09 − 0.20)	0.74 (0.54 − 0.94)	-0.91 (-1.16 to -0.66)	0.63 (0.51 − 0.74)	0.83 (0.33 − 1.34)	1.37 (0.84 − 1.90)
Democratic People's Republic of Korea	0.08 (0.07 − 0.09)	-0.21 (-0.23 to -0.19)	0.22 (0.19 − 0.25)	0.58 (0.49 − 0.67)	0.86 (0.74 − 0.99)	1.49 (1.37 − 1.61)
Democratic Republic of the Congo	0.03 (0.01 − 0.04)	0.07 (0.06 − 0.08)	0.35 (0.32 − 0.38)	-0.05 (-0.28 to 0.19)	0.43 (0.12 − 0.73)	0.89 (0.69 − 1.08)
Denmark	0.47 (0.31 − 0.63)	0.05 (0.01 − 0.08)	0.13 (-0.01 − 0.27)	-0.64 (-1.21 to -0.07)	1.16 (0.61 − 1.71)	1.72 (1.15 − 2.28)
Djibouti	0.00 (-0.01 to 0.02)	0.07 (0.05 − 0.10)	0.13 (0.11 − 0.16)	0.25 (0.21 − 0.29)	1.54 (1.47 − 1.61)	0.70 (0.66 − 0.74)
Dominica	0.28 (0.25 − 0.31)	-0.11 (-0.12 to -0.09)	0.34 (0.23 − 0.45)	0.33 (0.27 − 0.39)	1.35 (1.28 − 1.42)	0.36 (-0.01 − 0.72)
Dominican Republic	0.22 (0.19 − 0.25)	-0.08 (-0.09 to -0.06)	0.11 (-0.01 − 0.22)	0.96 (0.78 − 1.15)	1.78 (1.67 − 1.89)	1.07 (0.61 − 1.54)
Ecuador	0.08 (0.00 − 0.17)	0.69 (0.53 − 0.85)	1.19 (1.02 − 1.36)	1.10 (0.83 − 1.37)	1.48 (1.20 − 1.76)	1.21 (0.79 − 1.63)
Egypt	0.12 (0.11 − 0.14)	0.07 (0.06 − 0.08)	0.24 (0.19 − 0.29)	-1.79 (-2.12 to -1.45)	2.77 (2.54 − 3.00)	4.07 (3.82 − 4.33)
El Salvador	0.23 (0.21 − 0.25)	0.20 (0.18 − 0.21)	-0.19 (-0.25 to -0.12)	1.15 (1.01 − 1.29)	2.15 (2.03 − 2.26)	2.88 (2.44 − 3.31)
Equatorial Guinea	0.04 (0.03 − 0.06)	0.04 (0.01 − 0.08)	0.32 (0.27 − 0.37)	0.78 (0.55 − 1.00)	3.28 (3.02 − 3.54)	1.86 (1.77 − 1.95)
Eritrea	0.00 (-0.02 to 0.01)	0.08 (0.06 − 0.10)	0.13 (0.10 − 0.17)	0.01 (-0.05 − 0.08)	1.04 (0.96 − 1.11)	0.62 (0.49 − 0.74)
Estonia	0.00 (-0.03 to 0.03)	-0.14 (-0.16 to -0.13)	-0.47 (-0.51 to -0.42)	0.25 (0.04 − 0.46)	2.15 (1.68 − 2.61)	4.14 (3.43 − 4.85)
Ethiopia	-0.03 (-0.05 to -0.01)	0.01 (-0.03 − 0.05)	-0.54 (-0.61 to -0.47)	-0.71 (-0.88 to -0.53)	0.11 (-0.13 to 0.35)	0.53 (0.37 − 0.70)
Federated States of Micronesia	0.17 (0.15 − 0.20)	0.19 (0.16 − 0.21)	0.05 (0.02 − 0.08)	1.04 (0.99 − 1.09)	0.81 (0.78 − 0.84)	1.16 (1.11 − 1.21)

Location	BPH	UTI	Urolithiasis	Bladder cancer	Kidney cancer	Prostate cancer
Fiji	0.17 (0.16 − 0.18)	0.03 (0.02 − 0.04)	0.04 (-0.04 − 0.12)	1.38 (1.21 − 1.54)	-0.29 (-0.54 to -0.04)	0.62 (0.48 − 0.76)
Finland	0.34 (0.23 − 0.45)	0.10 (0.01 − 0.20)	0.02 (-0.67 − 0.72)	-0.96 (-1.07 to -0.84)	0.28 (0.09 − 0.46)	0.87 (0.34 − 1.39)
France	0.40 (0.27 − 0.53)	0.06 (0.04 − 0.08)	-0.12 (-0.16 to -0.09)	-0.07 (-0.19 to 0.05)	2.09 (1.90 − 2.27)	0.73 (0.49 − 0.98)
Gabon	0.06 (0.04 − 0.07)	0.19 (0.16 − 0.22)	0.42 (0.41 − 0.44)	0.00 (-0.08 to 0.08)	1.92 (1.85 − 1.98)	0.98 (0.94 − 1.01)
Georgia	0.12 (0.06 − 0.18)	0.23 (0.14 − 0.31)	-0.64 (-0.76 to -0.52)	0.89 (0.29 − 1.49)	2.53 (2.17 − 2.89)	4.30 (3.58 − 5.03)
Germany	0.47 (0.34 − 0.61)	0.09 (0.01 − 0.17)	0.59 (0.10 − 1.07)	-0.70 (-0.87 to -0.54)	-0.14 (-0.29 to 0.00)	0.52 (0.12 − 0.92)
Ghana	0.05 (0.04 − 0.06)	0.15 (0.14 − 0.17)	0.75 (0.63 − 0.87)	-0.16 (-0.29 to -0.03)	3.56 (3.43 − 3.68)	0.52 (0.35 − 0.69)
Greece	0.38 (0.24 − 0.53)	0.01 (-0.01 − 0.03)	1.05 (0.70 − 1.41)	-0.53 (-0.65 to -0.41)	0.77 (0.59 − 0.95)	-0.33 (-0.67 to 0.02)
Greenland	0.35 (0.23 − 0.47)	0.04 (0.01 − 0.06)	-0.11 (-0.19 to -0.03)	-0.70 (-0.88 to -0.53)	0.95 (0.81 − 1.09)	0.05 (-0.03 − 0.13)
Grenada	0.24 (0.22 − 0.27)	0.05 (0.04 − 0.06)	1.75 (1.71 − 1.79)	0.85 (0.51 − 1.20)	1.85 (1.63 − 2.08)	2.08 (0.67 − 3.51)
Guam	0.08 (0.06 − 0.10)	0.16 (0.13 − 0.20)	-0.31 (-0.34 to -0.28)	1.86 (1.49 − 2.23)	0.84 (0.52 − 1.16)	0.55 (0.25 − 0.85)
Guatemala	0.24 (0.22 − 0.27)	0.14 (0.13 − 0.15)	-0.12 (-0.19 to -0.06)	-0.66 (-0.9 to -0.43)	1.03 (0.69 − 1.37)	2.22 (1.43 − 3.03)
Guinea	0.03 (0.02 − 0.05)	0.10 (0.08 − 0.12)	0.06 (0.02 − 0.10)	0.69 (0.63 − 0.74)	0.89 (0.83 − 0.96)	1.24 (1.18 − 1.31)
Guinea-Bissau	0.05 (0.04 − 0.06)	0.14 (0.12 − 0.15)	-0.03 (-0.06 to 0.01)	-0.62 (-0.70 to -0.54)	1.79 (1.66 − 1.92)	2.12 (2.01 − 2.23)
Guyana	0.26 (0.23 − 0.29)	0.17 (0.15 − 0.18)	0.86 (0.79 − 0.93)	0.81 (0.62 − 0.99)	1.41 (1.22 − 1.59)	1.02 (0.75 − 1.29)

Location	BPH	UTI	Urolithiasis	Bladder cancer	Kidney cancer	Prostate cancer
Haiti	0.24 (0.22 − 0.26)	0.09 (0.08 − 0.10)	0.41 (0.36 − 0.47)	0.20 (0.14 − 0.25)	0.27 (0.21 − 0.32)	0.71 (0.67 − 0.75)
Honduras	0.21 (0.19 − 0.23)	0.06 (0.05 − 0.07)	0.00 (-0.01 to 0.01)	1.65 (1.52 − 1.78)	1.90 (1.75 − 2.04)	2.08 (2.01 − 2.16)
Hungary	0.06 (-0.01 − 0.13)	-0.04 (-0.06 to -0.03)	-1.14 (-1.31 to -0.96)	0.85 (0.53 − 1.18)	0.66 (0.31 − 1.00)	0.68 (0.39 − 0.98)
Iceland	0.48 (0.33 − 0.63)	-0.23 (-0.36 to -0.10)	-0.31 (-0.51 to -0.11)	-0.73 (-0.96 to -0.50)	0.98 (0.65 − 1.31)	-0.13 (-0.46 to 0.19)
India	0.22 (0.17 − 0.27)	0.26 (0.18 − 0.34)	0.17 (0.03 − 0.31)	0.35 (0.16 − 0.55)	1.77 (1.66 − 1.88)	1.21 (1.04 − 1.39)
Indonesia	-0.36 (-0.57 to -0.15)	-0.61 (-0.73 to -0.5)	-1.41 (-1.64 to -1.18)	0.69 (0.65 − 0.72)	1.84 (1.80 − 1.89)	2.16 (2.12 − 2.21)
Iraq	0.09 (0.07 − 0.11)	0.08 (0.06 − 0.10)	0.07 (0.02 − 0.12)	1.61 (1.42 − 1.81)	1.94 (1.73 − 2.14)	3.11 (2.80 − 3.44)
Ireland	0.46 (0.33 − 0.60)	0.08 (0.06 − 0.11)	-0.15 (-0.18 to -0.12)	-0.03 (-0.17 to 0.11)	1.37 (1.07 − 1.68)	0.26 (-0.23 − 0.75)
Islamic Republic of Iran	0.11 (0.10 − 0.13)	0.02 (-0.03 − 0.07)	0.04 (-0.03 − 0.11)	1.3 (1.20 − 1.39)	2.06 (1.93 − 2.19)	2.90 (2.72 − 3.08)
Israel	0.44 (0.31 − 0.57)	0.00 (-0.02 to 0.03)	0.08 (0.03 − 0.13)	0.03 (-0.30 − 0.37)	0.58 (0.17 − 0.99)	-0.29 (-0.85 to 0.27)
Italy	0.07 (-0.05 − 0.19)	-2.36 (-2.91 to -1.81)	-0.64 (-0.72 to -0.57)	-0.55 (-0.69 to -0.42)	0.59 (0.35 − 0.83)	0.22 (-0.24 − 0.67)
Jamaica	0.26 (0.24 − 0.29)	-0.05 (-0.06 to -0.03)	0.47 (0.40 − 0.54)	0.8 (0.48 − 1.12)	1.25 (0.90 − 1.60)	2.21 (1.44 − 2.97)
Japan	-0.03 (-0.14 to 0.08)	0.02 (-0.07 − 0.11)	-0.27 (-0.34 to -0.21)	0.38 (0.30 − 0.47)	1.15 (0.87 − 1.44)	2.28 (1.75 − 2.80)
Jordan	0.21 (0.13 − 0.30)	0.80 (0.68 − 0.92)	1.24 (1.06 − 1.42)	0.61 (0.43 − 0.78)	2.15 (1.93 − 2.37)	2.80 (2.53 − 3.07)
Kazakhstan	0.04 (0.01 − 0.07)	0.13 (0.11 − 0.16)	0.68 (0.56 − 0.80)	-0.68 (-1.05 to -0.31)	0.11 (-0.31 − 0.54)	1.77 (1.38 − 2.16)

Location	BPH	UTI	Urolithiasis	Bladder cancer	Kidney cancer	Prostate cancer
Kenya	-0.01 (-0.02 to 0.00)	0.35 (0.28 − 0.42)	0.03 (-0.02 − 0.09)	1.19 (1.09 − 1.29)	2.22 (2.04 − 2.40)	2.23 (2.05 − 2.41)
Kingdom of Eswatini	0.10 (0.08 − 0.11)	-0.03 (-0.05 to -0.01)	0.12 (0.09 − 0.15)	0.56 (0.24 − 0.87)	1.73 (1.40 − 2.05)	1.20 (1.03 − 1.37)
Kiribati	0.14 (0.12 − 0.15)	0.12 (0.11 − 0.13)	-0.15 (-0.20 to -0.10)	0.74 (0.63 − 0.85)	0.95 (0.73 − 1.18)	0.70 (0.67 − 0.72)
Kuwait	0.11 (0.08 − 0.13)	0.34 (0.27 − 0.42)	-0.03 (-0.12 to 0.06)	0.68 (0.14 − 1.22)	1.06 (0.37 − 1.75)	3.74 (3.11 − 4.38)
Kyrgyzstan	-0.02 (-0.06 to 0.02)	0.21 (0.18 − 0.23)	-0.26 (-0.40 to -0.11)	-0.92 (-1.31 to -0.52)	2.70 (2.04 − 3.36)	0.46 (-0.01 to 0.93)
Lao People's Democratic Republic	0.08 (0.06 − 0.09)	0.05 (0.02 − 0.09)	-0.4 (-0.48 to -0.33)	-0.12 (-0.24 to 0.01)	1.00 (0.92 − 1.09)	1.20 (1.17 − 1.24)
Latvia	0.01 (-0.02 to 0.03)	0.20 (-0.31 − 0.71)	-0.64 (-0.72 to -0.56)	1.04 (0.84 − 1.23)	3.66 (3.10 − 4.24)	3.48 (3.21 − 3.75)
Lebanon	0.11 (0.10 − 0.12)	0.07 (0.04 − 0.09)	0.22 (0.19 − 0.26)	1.09 (0.83 − 1.34)	2.78 (2.48 − 3.07)	3.86 (3.53 − 4.19)
Lesotho	0.09 (0.08 − 0.11)	-0.07 (-0.10 to -0.03)	0.25 (0.22 − 0.27)	1.49 (1.24 − 1.73)	3.16 (2.90 − 3.41)	1.31 (1.17 − 1.44)
Liberia	0.06 (0.05 − 0.07)	0.05 (0.03 − 0.07)	0.11 (0.07 − 0.15)	-0.43 (-0.56 to -0.31)	2.45 (2.01 − 2.88)	2.23 (2.12 − 2.35)
Libya	0.16 (0.15 − 0.17)	0.10 (0.08 − 0.12)	0.09 (0.06 − 0.13)	1.63 (1.36 − 1.90)	1.97 (1.71 − 2.23)	2.14 (1.92 − 2.37)
Lithuania	0.00 (-0.03 to 0.03)	-0.27 (-0.67 to 0.15)	-1.21 (-1.29 to -1.14)	-0.59 (-1.26 to 0.09)	2.67 (2.11 − 3.22)	3.52 (3.00 − 4.04)
Luxembourg	0.60 (0.45 − 0.75)	0.14 (-0.09 − 0.37)	-0.51 (-1.23 to 0.21)	-0.3 (-0.48 to -0.11)	0.69 (0.38 − 1.00)	-0.14 (-0.40 to 0.12)
Madagascar	-0.01 (-0.03 to 0.00)	0.08 (0.07 − 0.10)	0.06 (0.03 − 0.08)	-0.50 (-0.62 to -0.39)	0.41 (0.25 − 0.57)	-0.2 (-0.33 to -0.07)
Malawi	0.01 (0.00 − 0.03)	0.12 (0.11 − 0.13)	0.01 (-0.01 − 0.04)	0.60 (0.45 − 0.75)	0.78 (0.72 − 0.84)	1.54 (1.42 − 1.66)

Location	BPH	UTI	Urolithiasis	Bladder cancer	Kidney cancer	Prostate cancer
Malaysia	0.04 (0.03 − 0.06)	0.09 (0.07 − 0.11)	0.01 (-0.08 − 0.09)	0.87 (0.74 − 1.00)	1.03 (0.86 − 1.20)	1.61 (1.44 − 1.79)
Maldives	0.03 (0.02 − 0.04)	-0.26 (-0.37 to -0.16)	0.21 (0.17 − 0.25)	-0.63 (-0.77 to -0.49)	0.43 (0.33 − 0.53)	1.27 (1.10 − 1.44)
Mali	0.02 (0.01 − 0.03)	0.01 (0.00 − 0.02)	0.02 (-0.02 − 0.05)	0.24 (0.18 − 0.31)	0.78 (0.70 − 0.86)	1.00 (0.91 − 1.09)
Malta	0.34 (0.22 − 0.46)	-0.40 (-0.82 to 0.03)	0.47 (-0.21 − 1.14)	-0.75 (-0.88 to -0.62)	1.29 (1.08 − 1.49)	0.11 (-0.15 − 0.38)
Marshall Islands	0.21 (0.19 − 0.23)	0.15 (0.13 − 0.17)	0.02 (-0.02 − 0.07)	1.32 (1.19 − 1.45)	1.15 (1.11 − 1.19)	1.15 (1.08 − 1.23)
Mauritania	0.00 (-0.01 to 0.02)	0.09 (0.08 − 0.11)	0.05 (0.02 − 0.08)	-0.55 (-0.72 to -0.38)	2.57 (2.51 − 2.63)	2.46 (2.37 − 2.56)
Mauritius	0.15 (0.13 − 0.17)	0.07 (0.05 − 0.09)	-0.05 (-0.13 to 0.03)	-0.85 (-1.54 to -0.14)	-1.46 (-3.15 to 0.26)	1.17 (0.84 − 1.51)
Mexico	0.11 (0.07 − 0.16)	1.16 (0.79 − 1.52)	0.67 (0.00 − 1.34)	0.11 (-0.04 − 0.26)	1.80 (1.68 − 1.91)	1.12 (0.84 − 1.41)
Mongolia	0.01 (-0.02 − 0.05)	-0.04 (-0.07 to -0.01)	-0.04 (-0.12 to 0.04)	-2.07 (-2.52 to -1.61)	5.36 (4.89 − 5.82)	2.49 (2.34 − 2.63)
Montenegro	0.06 (-0.01 − 0.13)	-0.02 (-0.03 to 0.00)	-0.17 (-0.23 to -0.12)	1.08 (0.95 − 1.21)	1.52 (1.32 − 1.71)	1.84 (1.46 − 2.21)
Morocco	0.13 (0.12 − 0.14)	0.08 (0.07 − 0.09)	0.19 (0.15 − 0.23)	1.42 (1.23 − 1.61)	2.22 (2.10 − 2.33)	2.38 (2.15 − 2.62)
Mozambique	0.02 (0.00 − 0.03)	0.11 (0.09 − 0.13)	0.12 (0.09 − 0.14)	0.77 (0.68 − 0.86)	1.50 (1.39 − 1.61)	1.23 (1.16 − 1.29)
Myanmar	0.05 (0.04 − 0.06)	0.18 (0.16 − 0.20)	-0.29 (-0.36 to -0.21)	-0.38 (-0.50 to -0.26)	0.97 (0.90 − 1.04)	1.43 (1.39 − 1.46)
Namibia	0.06 (0.04 − 0.07)	0.08 (0.06 − 0.09)	0.03 (0.01 − 0.05)	0.53 (0.33 − 0.72)	1.56 (1.46 − 1.67)	2.95 (2.73 − 3.17)
Nepal	0.04 (-0.03 − 0.11)	-0.35 (-0.55 to -0.15)	0.25 (0.20 − 0.30)	0.30 (0.05 − 0.54)	1.68 (1.50 − 1.85)	0.95 (0.78 − 1.11)

Location	BPH	UTI	Urolithiasis	Bladder cancer	Kidney cancer	Prostate cancer
Netherlands	0.39 (0.25 − 0.52)	0.05 (0.04 − 0.06)	-0.44 (-0.51 to -0.37)	-0.35 (-0.56 to -0.14)	0.76 (0.54 − 0.99)	0.20 (-0.15 to 0.54)
New Zealand	-0.27 (-0.42 to -0.11)	0.42 (0.38 − 0.47)	-0.69 (-1.00 to -0.38)	-1.09 (-1.43 to -0.75)	0.74 (0.60 − 0.88)	-0.97 (-1.21 to -0.74)
Nicaragua	0.21 (0.19 − 0.23)	0.02 (0.00 − 0.04)	-0.12 (-0.16 to -0.08)	0.93 (0.69 − 1.18)	1.94 (1.74 − 2.14)	1.90 (1.62 − 2.19)
Niger	0.02 (0.01 − 0.03)	0.15 (0.14 − 0.16)	-0.02 (-0.06 to 0.02)	-0.68 (-0.76 to -0.59)	1.45 (1.35 − 1.54)	2.22 (2.08 − 2.36)
Nigeria	-0.01 (-0.03 to 0.00)	0.33 (0.27 − 0.39)	-0.27 (-0.37 to -0.18)	0.54 (0.48 − 0.60)	1.43 (1.31 − 1.55)	1.10 (1.01 − 1.19)
Northern Mariana Islands	0.11 (0.10 − 0.12)	0.18 (0.09 − 0.27)	-0.14 (-0.19 to -0.08)	4.07 (3.50 − 4.64)	0.93 (0.64 − 1.21)	1.58 (1.33 − 1.83)
Norway	0.21 (0.12 − 0.30)	-0.07 (-0.13 to 0.00)	0.06 (-0.31 − 0.42)	-0.63 (-0.9 to -0.37)	0.53 (0.10 − 0.96)	0.36 (-0.16 to 0.88)
Oman	0.10 (0.07 − 0.12)	0.15 (0.09 − 0.22)	0.25 (0.23 − 0.26)	0.96 (0.72 − 1.20)	2.64 (2.35 − 2.93)	2.73 (2.51 − 2.94)
Pakistan	0.15 (0.15 − 0.16)	0.66 (0.57 − 0.75)	-0.09 (-0.22 to 0.03)	0.43 (0.29 − 0.56)	1.41 (1.28 − 1.54)	1.16 (1.00 − 1.31)
Palestine	0.13 (0.12 − 0.14)	0.06 (0.03 − 0.08)	0.31 (0.26 − 0.37)	0.31 (0.13 − 0.49)	0.81 (0.74 − 0.89)	1.43 (1.27 − 1.59)
Panama	0.20 (0.19 − 0.22)	-0.02 (-0.04 to -0.01)	-0.20 (-0.26 to -0.14)	0.45 (0.24 − 0.66)	1.73 (1.58 − 1.87)	1.74 (1.19 − 2.30)
Papua New Guinea	0.11 (0.09 − 0.12)	0.08 (0.06 − 0.09)	-0.04 (-0.12 to 0.04)	0.70 (0.66 − 0.73)	0.43 (0.35 − 0.51)	1.02 (0.98 − 1.05)
Paraguay	0.14 (0.12 − 0.16)	-0.08 (-0.10 to -0.06)	-0.04 (-0.10 to 0.02)	1.55 (1.47 − 1.63)	2.46 (2.32 − 2.61)	2.33 (1.99 − 2.68)
Peru	0.22 (0.19 − 0.24)	0.17 (0.14 − 0.19)	-0.03 (-0.09 to 0.02)	0.33 (0.10 − 0.57)	1.06 (0.79 − 1.33)	1.77 (1.63 − 1.91)
Philippines	0.02 (0.00 − 0.04)	0.51 (0.41 − 0.61)	-0.13 (-0.22 to -0.05)	0.61 (0.56 − 0.67)	1.02 (0.96 − 1.08)	1.06 (1.02 − 1.11)

Location	BPH	UTI	Urolithiasis	Bladder cancer	Kidney cancer	Prostate cancer
Plurinational State of Bolivia	0.21 (0.19 − 0.24)	0.14 (0.10 − 0.18)	0.00 (-0.07 to 0.07)	0.20 (0.16 − 0.25)	1.36 (1.32 − 1.40)	1.01 (0.97 − 1.06)
Poland	-0.79 (-1.16 to -0.42)	-1.82 (-2.23 to -1.42)	-2.93 (-3.48 to -2.38)	1.43 (1.23 − 1.63)	0.36 (0.15 − 0.56)	2.67 (2.33 − 3.02)
Portugal	0.46 (0.32 − 0.60)	-0.63 (-0.76 to -0.49)	0.31 (0.25 − 0.36)	0.91 (0.78 − 1.04)	1.75 (1.43 − 2.07)	0.67 (0.35 − 0.99)
Principality of Monaco	0.44 (0.31 − 0.58)	0.05 (0.04 − 0.07)	-0.15 (-0.21 to -0.09)	0.67 (0.59 − 0.75)	2.29 (1.96 − 2.62)	1.17 (1.02 − 1.32)
Puerto Rico	0.23 (0.20 − 0.27)	0.06 (0.05 − 0.08)	0.19 (0.11 − 0.26)	0.25 (0.05 − 0.45)	1.43 (1.27 − 1.58)	0.10 (-0.16 to 0.36)
Qatar	0.25 (0.22 − 0.28)	-0.69 (-1.20 to -0.18)	0.39 (0.32 − 0.46)	0.07 (-0.35 to 0.49)	0.74 (0.52 − 0.96)	2.68 (2.33 − 3.03)
Republic of Cabo Verde	0.07 (0.06 − 0.09)	-0.13 (-0.13 to -0.12)	0.25 (0.21 − 0.29)	6.19 (4.76 − 7.65)	9.41 (7.46 − 11.41)	3.27 (2.88 − 3.66)
Republic of Côte d’Ivoire	0.04 (0.03 − 0.05)	0.06 (0.05 − 0.08)	0.09 (0.04 − 0.13)	-0.06 (-0.15 to 0.02)	1.01 (0.91 − 1.10)	0.65 (0.59 − 0.71)
Republic of Korea	0.13 (0.08 − 0.19)	0.02 (0.00 − 0.04)	-0.01 (-0.04 to 0.03)	0.30 (0.14 − 0.47)	2.59 (2.10 − 3.08)	4.35 (3.76 − 4.94)
Republic of Moldova	-0.02 (-0.05 to 0.01)	0.04 (0.02 − 0.06)	0.08 (-0.06 to 0.22)	0.31 (0.05 − 0.57)	1.59 (1.26 − 1.92)	3.87 (3.19 − 4.55)
Republic of Nauru	0.12 (0.11 − 0.14)	0.27 (0.25 − 0.30)	-0.17 (-0.2 to -0.14)	0.52 (0.48 − 0.56)	-0.13 (-0.4 to 0.14)	1.00 (0.96 − 1.04)
Republic of Niue	0.19 (0.17 − 0.20)	0.05 (0.03 − 0.07)	0.08 (0.04 − 0.13)	1.21 (1.10 − 1.32)	1.48 (1.35 − 1.60)	1.29 (1.23 − 1.34)
Republic of Palau	0.16 (0.15 − 0.17)	-0.17 (-0.24 to -0.10)	0.17 (0.11 − 0.23)	0.46 (0.34 − 0.57)	0.61 (0.47 − 0.75)	0.03 (-0.02 to 0.08)
Republic of San Marino	0.43 (0.30 − 0.56)	0.07 (0.01 − 0.12)	0.01 (-0.02 to 0.04)	-0.77 (-1.18 to -0.37)	0.38 (-0.05 to 0.81)	-0.18 (-0.63 to 0.28)
Republic of the Gambia	0.04 (0.02 − 0.05)	0.21 (0.20 − 0.22)	0.05 (0.01 − 0.08)	0.30 (0.18 − 0.42)	1.12 (0.95 − 1.29)	1.14 (1.06 − 1.23)

Location	BPH	UTI	Urolithiasis	Bladder cancer	Kidney cancer	Prostate cancer
Romania	0.05 (-0.01 − 0.10)	0.49 (0.24 − 0.74)	0.24 (-0.10 to 0.57)	1.73 (1.57 − 1.88)	2.90 (2.64 − 3.16)	3.56 (3.38 − 3.75)
Russian Federation	-0.02 (-0.05 to 0.00)	0.31 (0.20 − 0.42)	-0.82 (-0.87 to -0.77)	0.08 (-0.14 to 0.30)	0.82 (0.62 − 1.01)	4.02 (3.76 − 4.27)
Rwanda	-0.01 (-0.02 to 0.00)	0.10 (0.08 − 0.13)	-0.05 (-0.08 to -0.02)	-1.35 (-1.58 to -1.11)	0.27 (0.05 − 0.48)	0.23 (0.12 − 0.35)
Saint Kitts and Nevis	0.19 (0.17 − 0.21)	0.05 (0.04 − 0.06)	0.47 (0.39 − 0.54)	0.84 (0.61 − 1.07)	1.08 (0.89 − 1.28)	2.01 (1.70 − 2.32)
Saint Lucia	0.23 (0.21 − 0.25)	-0.02 (-0.04 to -0.01)	0.54 (0.47 − 0.61)	-0.46 (-0.65 to -0.27)	0.75 (0.54 − 0.97)	0.16 (-0.14 to 0.45)
Saint Vincent and the Grenadines	0.23 (0.21 − 0.25)	0.04 (0.03 − 0.06)	0.87 (0.84 − 0.90)	0.65 (0.41 − 0.88)	0.75 (0.56 − 0.93)	1.09 (0.83 − 1.36)
Samoa	0.10 (0.08 − 0.13)	0.10 (0.09 − 0.10)	-0.01 (-0.06 to 0.04)	0.25 (0.18 − 0.31)	0.20 (0.12 − 0.29)	0.37 (0.34 − 0.39)
Sao Tome and Principe	0.04 (0.03 − 0.05)	0.18 (0.16 − 0.20)	0.16 (0.14 − 0.19)	1.26 (1.18 − 1.34)	1.06 (0.70 − 1.41)	1.74 (1.55 − 1.92)
Saudi Arabia	0.15 (0.15 − 0.16)	0.17 (0.13 − 0.20)	0.22 (0.18 − 0.25)	1.06 (0.96 − 1.16)	3.72 (3.50 − 3.94)	2.71 (2.51 − 2.91)
Senegal	0.07 (0.06 − 0.08)	0.08 (0.07 − 0.09)	0.04 (0.00 − 0.08)	-0.44 (-0.54 to -0.34)	2.16 (2.01 − 2.30)	2.52 (2.40 − 2.64)
Serbia	0.10 (0.05 − 0.15)	-0.09 (-0.11 to -0.07)	0.39 (-0.09 − 0.86)	0.83 (0.73 − 0.92)	1.19 (1.09 − 1.28)	1.80 (1.58 − 2.02)
Seychelles	0.14 (0.12 − 0.17)	0.00 (-0.03 − 0.03)	0.50 (0.43 − 0.56)	0.30 (0.11 − 0.49)	-0.20 (-0.87 to 0.47)	1.91 (1.47 − 2.35)
Sierra Leone	0.06 (0.05 − 0.07)	0.04 (0.01 − 0.07)	0.09 (0.05 − 0.12)	-0.55 (-0.62 to -0.47)	2.04 (1.90 − 2.18)	2.11 (1.99 − 2.22)
Singapore	0.09 (0.02 − 0.15)	0.06 (0.04 − 0.08)	-0.21 (-0.25 to -0.16)	-0.39 (-0.6 to -0.18)	1.35 (0.92 − 1.78)	2.32 (2.03 − 2.61)
Slovakia	0.04 (0.00 − 0.09)	-0.07 (-0.43 to 0.29)	-0.54 (-1.52 to 0.45)	0.46 (0.38 − 0.55)	1.93 (1.64 − 2.23)	2.14 (1.99 − 2.28)

Location	BPH	UTI	Urolithiasis	Bladder cancer	Kidney cancer	Prostate cancer
Slovenia	0.04 (-0.01 − 0.10)	0.65 (0.40 − 0.90)	0.13 (-0.49 − 0.76)	0.77 (0.60 − 0.94)	2.17 (1.77 − 2.57)	2.58 (2.12 − 3.04)
Socialist Republic of Viet Nam	0.14 (0.09 − 0.19)	-0.20 (-0.24 to -0.16)	0.99 (0.79 − 1.19)	1.66 (1.61 − 1.71)	3.25 (3.06 − 3.44)	2.44 (2.32 − 2.57)
Solomon Islands	0.15 (0.13 − 0.17)	0.27 (0.25 − 0.29)	0.01 (-0.03 − 0.05)	0.90 (0.84 − 0.97)	0.62 (0.54 − 0.70)	0.99 (0.95 − 1.03)
Somalia	0.01 (0.00 − 0.02)	0.09 (0.06 − 0.11)	0.14 (0.12 − 0.16)	-0.24 (-0.28 to -0.20)	0.03 (-0.05 − 0.11)	0.01 (-0.04 to 0.07)
South Africa	0.09 (0.07 − 0.11)	-0.06 (-0.08 to -0.04)	-0.14 (-0.23 to -0.06)	0.64 (0.50 − 0.79)	1.60 (1.50 − 1.70)	1.56 (1.43 − 1.68)
South Sudan	-0.01 (-0.03 to 0.01)	0.21 (0.18 − 0.24)	-0.01 (-0.04 to 0.02)	-0.05 (-0.19 to 0.09)	1.09 (0.92 − 1.27)	0.22 (0.16 − 0.29)
Spain	0.63 (0.49 − 0.77)	0.02 (-0.01 − 0.05)	0.95 (0.76 − 1.14)	-0.29 (-0.49 to -0.08)	1.79 (1.58 − 2.01)	0.33 (-0.09 to 0.76)
Sri Lanka	0.12 (0.10 − 0.14)	0.38 (0.37 − 0.39)	-0.25 (-0.31 to -0.19)	1.07 (0.86 − 1.29)	-5.74 (-6.85 to -4.62)	1.98 (1.85 − 2.10)
Sudan	0.11 (0.09 − 0.13)	0.10 (0.09 − 0.10)	0.18 (0.11 − 0.24)	0.42 (0.32 − 0.53)	2.02 (1.91 − 2.13)	1.98 (1.86 − 2.11)
Suriname	0.29 (0.26 − 0.32)	0.16 (0.14 − 0.18)	1.47 (1.38 − 1.56)	0.54 (0.35 − 0.73)	1.08 (0.95 − 1.22)	1.40 (1.18 − 1.63)
Sweden	0.01 (-0.14 to 0.17)	-0.04 (-0.10 to 0.01)	-0.21 (-0.45 to 0.04)	0.31 (-0.03 to 0.64)	-1.02 (-1.26 to -0.78)	-0.30 (-0.77 to 0.18)
Switzerland	0.26 (0.16 − 0.36)	0.23 (-0.16 − 0.62)	-0.53 (-1.13 to 0.08)	0.97 (0.61 − 1.34)	1.09 (0.59 − 1.58)	-0.96 (-1.21 to -0.71)
Syrian Arab Republic	0.09 (0.08 − 0.11)	0.28 (0.21 − 0.34)	0.09 (0.01 − 0.17)	1.30 (1.13 − 1.47)	1.56 (1.44 − 1.69)	2.54 (2.37 − 2.71)
Taiwan (Province of China)	0.00 (-0.02 to 0.02)	0.25 (0.24 − 0.27)	0.92 (0.73 − 1.11)	-0.11 (-0.44 to 0.23)	3.25 (2.54 − 3.97)	3.73 (3.14 − 4.32)
Tajikistan	0.06 (0.03 − 0.09)	0.16 (0.12 − 0.20)	0.26 (0.22 − 0.29)	-0.88 (-1.11 to -0.65)	-0.72 (-0.84 to -0.61)	-1.27 (-1.44 to -1.11)

Location	BPH	UTI	Urolithiasis	Bladder cancer	Kidney cancer	Prostate cancer
Thailand	0.01 (-0.02 to 0.03)	-0.03 (-0.05 to -0.01)	-0.79 (-0.97 to -0.61)	0.04 (-0.12 − 0.21)	2.12 (2.04 − 2.19)	1.85 (1.7 − 2.00)
The former Yugoslav Republic of Macedonia	0.09 (0.02 − 0.16)	0.03 (0.02 − 0.05)	-0.15 (-0.21 to -0.09)	0.90 (0.58 − 1.23)	2.67 (2.22 − 3.11)	2.22 (1.85 − 2.59)
Timor-Leste	0.05 (0.03 − 0.07)	0.15 (0.12 − 0.18)	0.00 (0.00 − 0.00)	0.59 (0.40 − 0.77)	0.85 (0.63 − 1.07)	1.66 (1.58 − 1.74)
Togo	0.02 (0.01 − 0.03)	0.06 (0.05 − 0.07)	0.03 (0.00 − 0.06)	-0.48 (-0.61 to -0.35)	2.15 (2.10 − 2.19)	2.45 (2.33 − 2.56)
Tokelau	0.13 (0.11 − 0.15)	0.12 (0.09 − 0.14)	0.13 (0.07 − 0.18)	0.91 (0.85 − 0.97)	1.67 (1.34 − 2.00)	1.15 (1.11 − 1.19)
Tonga	0.10 (0.09 − 0.12)	0.04 (0.01 − 0.06)	0.11 (0.05 − 0.18)	1.07 (0.91 − 1.23)	0.97 (0.85 − 1.10)	0.75 (0.65 − 0.86)
Trinidad and Tobago	0.19 (0.16 − 0.21)	0.07 (0.05 − 0.08)	2.85 (2.61 − 3.10)	0.36 (0.18 − 0.53)	1.01 (0.89 − 1.13)	0.48 (0.28 − 0.67)
Tunisia	0.13 (0.11 − 0.14)	0.12 (0.11 − 0.12)	0.17 (0.13 − 0.21)	0.94 (0.85 − 1.04)	1.64 (1.61 − 1.68)	2.30 (2.25 − 2.35)
Turkey	0.11 (0.09 − 0.13)	0.41 (0.23 − 0.59)	0.36 (0.33 − 0.39)	1.09 (0.88 − 1.29)	1.25 (1.13 − 1.38)	2.28 (2.07 − 2.48)
Turkmenistan	0.04 (0.01 − 0.07)	0.26 (0.23 − 0.29)	0.11 (0.07 − 0.14)	-1.05 (-1.41 to -0.69)	4.14 (3.62 − 4.65)	1.51 (1.16 − 1.85)
Tuvalu	0.15 (0.13 − 0.17)	-0.20 (-0.21 to -0.18)	0.12 (0.09 − 0.16)	1.25 (1.16 − 1.35)	1.43 (1.37 − 1.48)	1.27 (1.18 − 1.35)
Uganda	-0.01 (-0.03 to 0.00)	0.13 (0.12 − 0.13)	0.06 (0.02 − 0.09)	-0.23 (-0.37 to -0.08)	1.81 (1.68 − 1.94)	0.80 (0.67 − 0.93)
Ukraine	-0.03 (-0.05 to 0.00)	0.14 (-0.17 to 0.46)	-0.54 (-0.60 to -0.48)	0.34 (0.08 − 0.60)	0.68 (0.41 − 0.95)	0.63 (0.46 − 0.81)
United Arab Emirates	0.14 (0.12 − 0.16)	-0.01 (-0.09 to 0.07)	0.32 (0.27 − 0.37)	1.22 (0.73 − 1.71)	3.04 (2.59 − 3.50)	2.59 (2.09 − 3.10)
United Kingdom of Great Britain and Northern Ireland	0.53 (0.39 − 0.67)	0.64 (-0.05 − 1.33)	0.75 (0.58 − 0.93)	-1.07 (-1.20 to -0.94)	1.33 (1.13 − 1.52)	0.72 (0.46 − 0.97)

Location	BPH	UTI	Urolithiasis	Bladder cancer	Kidney cancer	Prostate cancer
United Republic of Tanzania	0.01 (0.00 − 0.02)	0.08 (0.06 − 0.10)	0.09 (0.06 − 0.11)	-0.62 (-0.70 to -0.54)	1.07 (0.96 − 1.18)	-0.02 (-0.11 to 0.07)
United States of America	0.49 (0.40 − 0.58)	-0.35 (-0.4 to -0.29)	-2.15 (-2.50 to -1.80)	0.12 (-0.06 to 0.30)	-0.07 (-0.29 to 0.16)	-1.16 (-1.28 to -1.04)
United States Virgin Islands	0.26 (0.23 − 0.29)	0.05 (0.01 − 0.08)	2.24 (2.14 − 2.33)	-0.77 (-1.00 to -0.54)	0.52 (0.30 − 0.74)	-1.04 (-1.45 to -0.62)
Uruguay	0.32 (0.21 − 0.44)	0.21 (0.19 − 0.22)	-0.04 (-0.10 to 0.02)	-0.27 (-0.40 to -0.13)	1.82 (1.67 − 1.96)	0.76 (0.38 − 1.14)
Uzbekistan	0.06 (0.03 − 0.09)	0.39 (0.33 − 0.46)	0.04 (-0.01 to 0.09)	0.65 (0.46 − 0.84)	1.49 (1.35 − 1.64)	0.82 (-0.03 − 1.67)
Vanuatu	0.13 (0.11 − 0.16)	0.16 (0.14 − 0.18)	-0.05 (-0.11 to 0.00)	0.65 (0.61 − 0.69)	0.47 (0.39 − 0.54)	0.95 (0.91 − 1.00)
Yemen	0.07 (0.06 − 0.08)	0.11 (0.10 − 0.13)	0.11 (0.04 − 0.17)	0.63 (0.57 − 0.68)	1.14 (1.00 − 1.29)	1.91 (1.80 − 2.01)
Zambia	0.01 (0.00 − 0.02)	0.10 (0.08 − 0.11)	0.08 (0.05 − 0.11)	0.75 (0.60 − 0.91)	2.82 (2.39 − 3.25)	2.99 (2.61 − 3.37)
Zimbabwe	0.11 (0.09 − 0.12)	0.01 (-0.01 to 0.03)	-0.06 (-0.09 to -0.03)	0.56 (0.25 − 0.88)	2.73 (1.96 − 3.52)	0.98 (0.67 − 1.30)

BPH benign prostatic hyperplasia, *UTI* urinary tract infections, *EAPC* estimated annual percentage change, *ASIR* age-standardized incidence rate

Table S8 EAPC of ASPR for the 6 urologic diseases in 204 countries and territories from 1990 to 2021 (95% CI)

Location	BPH	UTI	Urolithiasis	Bladder cancer	Kidney cancer	Prostate cancer
Afghanistan	0.15 (0.14 − 0.16)	-0.03 (-0.06 to -0.01)	0.18 (0.15 − 0.22)	0.61 (0.50 − 0.72)	2.06 (1.68 − 2.45)	2.17 (1.92 − 2.42)
Albania	0.10 (0.08 − 0.13)	0.06 (0.04 − 0.08)	-0.19 (-0.25 to -0.13)	1.37 (1.21 − 1.54)	3.81 (3.31 − 4.31)	2.72 (2.46 − 2.99)
Algeria	0.14 (0.13 − 0.15)	0.09 (0.07 − 0.10)	0.22 (0.17 − 0.27)	1.40 (1.30 − 1.50)	3.09 (2.94 − 3.24)	2.59 (2.48 − 2.70)
American Samoa	0.24 (0.22 − 0.26)	0.14 (0.12 − 0.16)	-0.30 (-0.35 to -0.25)	2.65 (2.33 − 2.98)	1.30 (0.99 − 1.61)	1.45 (1.29 − 1.62)
Andorra	0.29 (0.25 − 0.32)	0.17 (0.13 − 0.21)	-0.14 (-0.16 to -0.13)	-0.26 (-0.49 to -0.02)	1.16 (0.88 −1.43)	1.49 (1.17 − 1.81)
Angola	0.04 (0.03 − 0.05)	0.22 (0.21 − 0.23)	-0.01 (-0.03 to 0.01)	0.91 (0.71 − 1.11)	2.05 (1.87 − 2.23)	2.26 (2.09 − 2.42)
Antigua and Barbuda	0.22 (0.20 − 0.24)	0.00 (-0.04 to 0.03)	0.42 (0.36 − 0.47)	0.92 (0.70 − 1.14)	1.63 (1.47 − 1.79)	0.87 (0.49 − 1.25)
Argentina	0.25 (0.20 − 0.30)	0.18 (0.17 − 0.20)	-0.13 (-0.19 to -0.06)	-0.36 (-0.52 to -0.21)	1.66 (1.46 − 1.85)	1.35 (0.90 − 1.80)
Armenia	0.07 (0.04 − 0.09)	0.41 (0.32 − 0.51)	1.29 (1.20 − 1.39)	1.30 (1.05 − 1.54)	2.59 (2.20 − 2.97)	3.41 (3.19 − 3.64)
Australia	0.12 (0.10 − 0.14)	0.01 (0.00 − 0.02)	-0.40 (-0.47 to -0.33)	-0.83 (-0.9 to -0.75)	1.90 (1.53 − 2.28)	-0.03 (-0.78 to 0.72)
Austria	0.57 (0.45 − 0.69)	0.45 (0.28 − 0.62)	-1.89 (-2.31 to -1.47)	0.26 (0.12 − 0.41)	0.67 (0.40 − 0.93)	0.60 (0.18 − 1.02)
Azerbaijan	0.07 (0.05 − 0.09)	0.11 (0.08 − 0.14)	0.08 (0.02 − 0.14)	0.37 (0.20 − 0.55)	1.72 (1.44 − 2.01)	1.71 (1.48 − 1.93)
Bahrain	0.23 (0.21 − 0.24)	-0.08 (-0.11 to -0.06)	0.42 (0.37 − 0.46)	0.41 (0.23 − 0.59)	1.43 (1.22 − 1.63)	3.25 (3.16 − 3.33)
Bangladesh	0.12 (0.12 − 0.13)	0.23 (0.20 − 0.26)	0.26 (0.21 − 0.32)	0.72 (0.62 − 0.82)	1.98 (1.92 − 2.04)	2.21 (2.12 − 2.29)
Barbados	0.18 (0.16 − 0.20)	0.11 (0.10 − 0.12)	0.69 (0.65 − 0.73)	1.14 (0.99 − 1.30)	1.88 (1.67 − 2.10)	1.16 (0.98 − 1.33)

Location	BPH	UTI	Urolithiasis	Bladder cancer	Kidney cancer	Prostate cancer
Belarus	0.00 (-0.02 to 0.01)	0.00 (-0.01 to 0.02)	0.08 (0.04 − 0.11)	0.02 (-0.24 to 0.29)	3.94 (3.26 − 4.62)	3.65 (3.30 – 4.00)
Belgium	0.31 (0.26 − 0.35)	-0.03 (-0.11 to 0.05)	0.72 (-0.34 to 1.79)	-0.27 (-0.50 to -0.04)	1.10 (0.71 − 1.49)	0.08 (-0.38 to 0.54)
Belize	0.22 (0.20 − 0.24)	0.20 (0.19 − 0.21)	0.63 (0.56 − 0.69)	1.48 (1.19 − 1.78)	1.97 (1.66 − 2.27)	2.00 (1.45 − 2.55)
Benin	0.05 (0.04 − 0.07)	0.03 (0.01 − 0.05)	0.09 (0.05 − 0.13)	-0.31 (-0.44 to -0.18)	2.80 (2.65 − 2.94)	2.77 (2.68 − 2.86)
Bermuda	0.17 (0.15 − 0.19)	0.02 (0.00 − 0.04)	0.39 (0.32 − 0.47)	1.06 (0.86 − 1.27)	1.43 (1.26 − 1.60)	1.99 (1.63 − 2.35)
Bhutan	0.15 (0.14 − 0.16)	0.16 (0.14 − 0.17)	0.30 (0.25 − 0.35)	1.21 (1.10 − 1.32)	2.73 (2.65 − 2.80)	2.59 (2.51 − 2.67)
Bolivarian Republic of Venezuela	0.17 (0.16 − 0.19)	-0.01 (-0.03 to 0.01)	0.37 (0.30 − 0.43)	0.95 (0.74 − 1.17)	1.96 (1.67 − 2.25)	2.25 (1.74 − 2.76)
Bosnia and Herzegovina	0.21 (0.18 − 0.24)	-0.03 (-0.05 to -0.02)	-0.14 (-0.22 to -0.05)	2.72 (2.42 − 3.01)	4.37 (3.89 − 4.87)	3.96 (3.56 to 4.37)
Botswana	0.10 (0.09 − 0.12)	0.53 (-0.14 to 1.20)	0.25 (0.14 − 0.36)	-0.17 (-0.29 to -0.05)	2.75 (2.45 − 3.04)	1.76 (1.66 − 1.86)
Brazil	-0.33 (-0.44 to -0.21)	0.00 (-0.08 to 0.09)	1.62 (1.15 − 2.08)	0.92 (0.83 − 1.02)	2.54 (2.38 − 2.69)	1.69 (1.29 − 2.09)
Brunei Darussalam	0.12 (0.11 − 0.13)	0.08 (0.06 − 0.11)	-0.23 (-0.27 to -0.19)	0.34 (0.23 − 0.44)	2.26 (2.09 − 2.43)	2.32 (2.13 − 2.52)
Bulgaria	0.12 (0.09 − 0.15)	0.03 (-0.13 to 0.18)	-1.67 (-1.90 to -1.44)	2.13 (2.03 − 2.24)	5.07 (4.69 − 5.46)	3.24 (3.09 − 3.39)
Burkina Faso	0.04 (0.04 − 0.05)	0.07 (0.06 − 0.09)	0.12 (0.08 − 0.16)	-0.27 (-0.38 to -0.16)	2.87 (2.73 − 3.00)	2.68 (2.56 − 2.80)
Burundi	-0.03 (-0.04 to -0.01)	-0.06 (-0.09 to -0.04)	-0.09 (-0.13 to -0.05)	-0.67 (-0.80 to -0.54)	0.43 (0.29 − 0.58)	0.83 (0.75 − 0.91)
Cambodia	0.1 (0.09 − 0.12)	0.08 (0.06 − 0.09)	0.18 (0.16 − 0.19)	1.57 (1.39 − 1.74)	2.07 (1.86 − 2.27)	3.23 (3.09 − 3.36)

Location	BPH	UTI	Urolithiasis	Bladder cancer	Kidney cancer	Prostate cancer
Cameroon	0.06 (0.05 − 0.07)	0.00 (-0.02 to 0.02)	0.04 (0.01 − 0.08)	0.10 (-0.04 − 0.24)	2.74 (2.56 − 2.93)	2.76 (2.63 − 2.90)
Canada	0.24 (0.19 − 0.28)	-0.05 (-0.07 to -0.03)	0.22 (0.20 − 0.23)	-0.72 (-0.97 to -0.47)	1.23 (0.88 − 1.60)	-1.60 (-1.97 to -1.23)
Central African Republic	0.04 (0.03 − 0.05)	0.09 (0.06 − 0.12)	0.35 (0.33 − 0.37)	-0.28 (-0.37 to -0.20)	0.62 (0.55 − 0.69)	0.78 (0.73 − 0.84)
Chad	0.04 (0.03 − 0.05)	-0.05 (-0.08 to -0.02)	0.08 (0.03 − 0.13)	0.49 (0.38 − 0.60)	3.13 (3.00 − 3.27)	2.85 (2.73 − 2.97)
Chile	0.28 (0.24 − 0.32)	-0.15 (-0.43 to 0.13)	0.92 (0.51 − 1.33)	1.25 (1.08 − 1.41)	2.62 (2.48 − 2.77)	2.85 (2.51 − 3.20)
China	-0.24 (-0.41 to -0.07)	-0.25 (-0.31 to -0.18)	-2.37 (-2.55 to -2.18)	1.59 (1.54 − 1.64)	3.47 (3.23 − 3.72)	3.77 (3.66 − 3.88)
Colombia	0.14 (0.13 − 0.15)	0.08 (0.07 − 0.09)	-0.02 (-0.05 to 0.01)	0.49 (0.32 − 0.65)	2.71 (2.61 − 2.81)	1.76 (1.39 − 2.13)
Commonwealth of the Bahamas	0.18 (0.16 − 0.20)	0.14 (0.10 − 0.17)	0.38 (0.31 − 0.46)	1.19 (1.02 − 1.36)	1.77 (1.62 − 1.92)	1.34 (1.02 − 1.66)
Comoros	-0.01 (-0.02 to 0.00)	0.07 (0.06 − 0.08)	0.08 (0.05 − 0.12)	0.10 (-0.02 to 0.22)	1.37 (1.23 − 1.51)	1.53 (1.44 − 1.62)
Congo	0.03 (0.01 − 0.04)	0.05 (0.04 − 0.06)	0.22 (0.19 − 0.25)	0.39 (0.18 − 0.61)	2.01 (1.84 − 2.17)	1.89 (1.72 −2.07)
Cook Islands	0.18 (0.17 − 0.19)	0.28 (0.27 − 0.30)	0.06 (0.00 − 0.11)	1.96 (1.86 − 2.06)	0.37 (0.16 − 0.57)	2.32 (2.11 − 2.52)
Costa Rica	0.18 (0.17 − 0.19)	0.04 (0.03 − 0.05)	-0.22 (-0.28 to -0.16)	0.33 (0.20 − 0.46)	3.67 (3.51 − 3.83)	2.72 (2.37 − 3.08)
Croatia	0.01 (-0.07 to 0.09)	0.44 (0.20 − 0.68)	-0.04 (-0.46 to 0.38)	1.95 (1.81 − 2.08)	2.16 (1.82 − 2.49)	2.38 (2.13 − 2.63)
Cuba	0.17 (0.15 − 0.19)	-0.02 (-0.03 to -0.01)	1.2 (1.14 − 1.25)	1.65 (1.48 − 1.82)	1.70 (1.59 − 1.81)	2.44 (2.30 − 2.58)
Cyprus	0.11 (0.10 − 0.12)	-0.29 (-0.68 to 0.10)	-0.74 (-1.31 to -0.16)	1.68 (1.38 − 1.98)	4.00 (3.52 − 4.49)	2.87 (2.52 − 3.21)

Location	BPH	UTI	Urolithiasis	Bladder cancer	Kidney cancer	Prostate cancer
Czech Republic	0.23 (0.21 − 0.25)	0.72 (0.53 − 0.92)	-0.91 (-1.15 to -0.66)	1.18 (1.00 − 1.36)	1.45 (0.86 − 2.04)	2.38 (1.78 − 2.98)
Democratic People's Republic of Korea	0.10 (0.09 − 0.10)	-0.21 (-0.22 to -0.19)	0.22 (0.20 − 0.25)	1.46 (1.36 − 1.57)	1.13 (0.93 − 1.32)	2.45 (2.24 − 2.66)
Democratic Republic of the Congo	0.03 (0.01 − 0.04)	0.07 (0.05 − 0.08)	0.35 (0.32 − 0.38)	0.58 (0.28 − 0.88)	1.15 (0.80 − 1.51)	1.61 (1.34 − 1.88)
Denmark	0.32 (0.27 to 0.37)	0.05 (0.01 to 0.08)	0.12 (-0.02 to 0.27)	-0.22 (-0.76 to 0.33)	2.21 (1.57 to 2.85)	2.80 (2.11 − 3.49)
Djibouti	0.01 (-0.01 to 0.02)	0.07 (0.05 − 0.10)	0.13 (0.10 − 0.16)	0.59 (0.51 − 0.67)	1.78 (1.67 − 1.90)	1.53 (1.46 − 1.61)
Dominica	0.26 (0.24 − 0.28)	-0.11 (-0.12 to -0.10)	0.34 (0.23 − 0.44)	0.62 (0.54 − 0.70)	2.06 (1.94 − 2.18)	0.58 (0.18 − 0.99)
Dominican Republic	0.2 (0.17 − 0.22)	-0.07 (-0.09 to -0.06)	0.1 (-0.02 − 0.22)	1.59 (1.44 − 1.73)	2.37 (2.24 − 2.50)	1.56 (1.06 − 2.06)
Ecuador	0.01 (-0.09 to 0.12)	0.69 (0.52 − 0.85)	1.17 (1.00 − 1.35)	1.87 (1.61 − 2.13)	2.37 (2.07 − 2.67)	2.15 (1.63 − 2.66)
Egypt	0.13 (0.11 − 0.14)	0.07 (0.06 − 0.08)	0.24 (0.19 − 0.29)	-1.35 (-1.73 to -0.96)	2.82 (2.65 − 2.98)	4.45 (4.26 − 4.65)
El Salvador	0.23 (0.22 − 0.24)	0.20 (0.18 − 0.21)	-0.19 (-0.25 to -0.12)	2.33 (2.12 − 2.55)	3.35 (3.15 − 3.55)	3.62 (3.11 − 4.13)
Equatorial Guinea	0.05 (0.04 − 0.06)	0.05 (0.01 − 0.08)	0.32 (0.27 − 0.37)	2.15 (1.90 − 2.40)	4.80 (4.49 − 5.10)	4.10 (3.96 − 4.23)
Eritrea	0.00 (-0.02 to 0.01)	0.08 (0.06 − 0.10)	0.13 (0.10 − 0.17)	0.34 (0.25 − 0.43)	1.48 (1.41 − 1.56)	1.48 (1.34 to 1.62)
Estonia	0.03 (0.01 − 0.05)	-0.14 (-0.16 to -0.12)	-0.46 (-0.50 to -0.41)	0.86 (0.63 − 1.09)	2.57 (2.07 − 3.06)	4.77 (4.09 − 5.47)
Ethiopia	-0.04 (-0.06 to -0.01)	0.01 (-0.03 to 0.06)	-0.55 (-0.62 to -0.47)	0.25 (0.08 − 0.43)	1.12 (0.84 −1.41)	2.08 (1.87 − 2.29)
Federated States of Micronesia	0.21 (0.19 − 0.24)	0.19 (0.16 − 0.21)	0.05 (0.02 − 0.08)	1.75 (1.69 − 1.81)	1.16 (1.12 − 1.20)	2.08 (2.00 − 2.15)

Location	BPH	UTI	Urolithiasis	Bladder cancer	Kidney cancer	Prostate cancer
Fiji	0.22 (0.21 − 0.22)	0.03 (0.02 − 0.04)	0.04 (-0.05 to 0.12)	1.50 (1.29 − 1.71)	-0.08 (-0.34 to 0.19)	1.04 (0.93 − 1.15)
Finland	0.21 (0.19 − 0.22)	0.10 (0.01 − 0.20)	0.03 (-0.67 to 0.73)	-0.36 (-0.51 to -0.20)	1.01 (0.73 − 1.28)	1.67 (1.08 − 2.26)
France	0.25 (0.22 − 0.29)	0.06 (0.04 − 0.08)	-0.12 (-0.16 to -0.08)	0.51 (0.33 − 0.68)	2.95 (2.72 − 3.19)	1.57 (1.28 − 1.87)
Gabon	0.05 (0.04 − 0.07)	0.19 (0.16 − 0.22)	0.42 (0.41 − 0.43)	0.62 (0.46 − 0.78)	2.72 (2.65 − 2.79)	2.14 (2.05 − 2.23)
Georgia	0.12 (0.05 − 0.19)	0.22 (0.14 − 0.31)	-0.64 (-0.76 to -0.52)	0.78 (0.27 − 1.29)	2.43 (2.05 − 2.80)	4.14 (3.60 − 4.69)
Germany	0.34 (0.30 − 0.37)	0.09 (0.01 − 0.16)	0.58 (0.10 − 1.07)	-0.10 (-0.20 to 0.00)	0.31 (0.09 − 0.52)	1.28 (0.81 − 1.74)
Ghana	0.06 (0.06 − 0.07)	0.15 (0.14 − 0.17)	0.75 (0.63 − 0.87)	0.29 (0.16 − 0.42)	3.93 (3.81 − 4.05)	1.34 (1.14 − 1.54)
Greece	0.19 (0.10 − 0.29)	0.01 (-0.01 to 0.03)	1.05 (0.70 − 1.40)	-0.18 (-0.27 to -0.09)	1.46 (1.23 − 1.69)	0.11 (-0.22 to 0.43)
Greenland	0.28 (0.24 − 0.33)	0.04 (0.02 − 0.06)	-0.11 (-0.19 to -0.03)	-0.03 (-0.21 to 0.14)	2.13 (1.93 − 2.32)	1.02 (0.94 − 1.10)
Grenada	0.23 (0.22 − 0.25)	0.05 (0.04 − 0.06)	1.75 (1.70 − 1.79)	1.09 (0.92 − 1.25)	2.36 (2.20 − 2.51)	2.06 (1.01 − 3.12)
Guam	0.1 (0.08 − 0.11)	0.16 (0.13 − 0.20)	-0.31 (-0.35 to -0.28)	2.44 (2.07 − 2.81)	1.16 (0.87 − 1.46)	1.19 (0.83 − 1.55)
Guatemala	0.25 (0.24 − 0.26)	0.14 (0.13 − 0.15)	-0.12 (-0.19 to -0.06)	0.69 (0.43 − 0.96)	2.02 (1.70 − 2.33)	3.55 (2.71 − 4.40)
Guinea	0.04 (0.03 − 0.06)	0.1 (0.07 − 0.12)	0.06 (0.01 − 0.10)	0.99 (0.95 − 1.02)	0.96 (0.86 − 1.05)	1.78 (1.74 − 1.82)
Guinea-Bissau	0.05 (0.04 − 0.06)	0.14 (0.12 − 0.15)	-0.03 (-0.07 to 0.01)	-0.02 (-0.09 to 0.05)	2.04 (1.85 − 2.24)	2.75 (2.66 − 2.85)
Guyana	0.25 (0.23 − 0.27)	0.17 (0.15 − 0.18)	0.86 (0.79 − 0.93)	1.30 (1.14 − 1.47)	2.16 (1.93 − 2.39)	1.61 (1.33 − 1.89)

Location	BPH	UTI	Urolithiasis	Bladder cancer	Kidney cancer	Prostate cancer
Haiti	0.22 (0.21 − 0.23)	0.09 (0.08 − 0.10)	0.41 (0.35 − 0.46)	0.58 (0.46 − 0.70)	0.82 (0.70 − 0.93)	1.26 (1.10 − 1.42)
Honduras	0.19 (0.18 − 0.20)	0.06 (0.05 − 0.07)	0.00 (-0.01 to 0.01)	1.89 (1.79 − 1.99)	2.25 (2.09 − 2.42)	2.47 (2.40 − 2.54)
Hungary	0.14 (0.11 − 0.17)	-0.04 (-0.06 to -0.02)	-1.14 (-1.31 to -0.96)	1.71 (1.33 − 2.08)	1.45 (1.02 − 1.89)	1.81 (1.48 − 2.15)
Iceland	0.33 (0.28 − 0.38)	-0.23 (-0.36 to -0.10)	-0.31 (-0.51 to -0.11)	-0.40 (-0.65 to -0.16)	1.71 (1.30 − 2.12)	0.23 (-0.11 to 0.57)
India	0.22 (0.18 − 0.26)	0.27 (0.19 − 0.34)	0.18 (0.03 − 0.32)	1.01 (0.80 − 1.22)	2.68 (2.57 − 2.79)	2.48 (2.31 − 2.65)
Indonesia	-0.44 (-0.70 to -0.17)	-0.61 (-0.73 to -0.50)	-1.41 (-1.64 to -1.18)	1.19 (1.12 − 1.27)	2.05 (1.95 − 2.15)	2.78 (2.74 − 2.82)
Iraq	0.10 (0.08 − 0.12)	0.08 (0.06 − 0.10)	0.07 (0.01 − 0.12)	2.22 (2.01 − 2.43)	2.54 (2.30 − 2.78)	3.73 (3.38 − 4.08)
Ireland	0.33 (0.29 − 0.36)	0.08 (0.06 − 0.11)	-0.15 (-0.18 to -0.12)	0.6 (0.48 − 0.72)	2.46 (2.02 − 2.90)	1.18 (0.65 − 1.71)
Islamic Republic of Iran	0.12 (0.11 − 0.14)	0.02 (-0.03 to 0.08)	0.04 (-0.04 to 0.11)	1.95 (1.86 − 2.04)	2.38 (2.23 − 2.54)	3.69 (3.53 − 3.86)
Israel	0.29 (0.26 − 0.32)	0.00 (-0.02 to 0.02)	0.07 (0.03 − 0.12)	0.69 (0.31 − 1.07)	1.59 (1.06 − 2.12)	0.6 (0.02 − 1.19)
Italy	0.03 (-0.01 to 0.07)	-2.33 (-2.87 to -1.79)	-0.64 (-0.72 to -0.57)	-0.15 (-0.35 to 0.05)	1.10 (0.76 − 1.44)	0.67 (0.22 − 1.13)
Jamaica	0.25 (0.24 − 0.27)	-0.05 (-0.06 to -0.03)	0.47 (0.39 − 0.54)	1.23 (0.89 − 1.58)	1.73 (1.35 − 2.11)	2.37 (1.62 − 3.14)
Japan	-0.11 (-0.19 to -0.02)	0.02 (-0.07 to 0.11)	-0.27 (-0.34 to -0.21)	0.70 (0.62 − 0.79)	1.75 (1.37 − 2.14)	2.91 (2.37 − 3.46)
Jordan	0.24 (0.14 − 0.34)	0.79 (0.67 − 0.90)	1.23 (1.05 − 1.41)	1.44 (1.21 − 1.67)	2.89 (2.63 − 3.16)	3.56 (3.26 − 3.85)
Kazakhstan	0.07 (0.05 − 0.09)	0.13 (0.11 − 0.16)	0.67 (0.55 − 0.79)	-0.15 (-0.44 to 0.13)	0.73 (0.38 − 1.07)	2.52 (2.05 – 3.00)

Location	BPH	UTI	Urolithiasis	Bladder cancer	Kidney cancer	Prostate cancer
Kenya	-0.01 (-0.02 to 0.00)	0.35 (0.28 − 0.42)	0.03 (-0.03 to 0.09)	1.43 (1.34 − 1.52)	2.73 (2.48 − 2.99)	2.42 (2.32 − 2.52)
Kingdom of Eswatini	0.10 (0.08 − 0.12)	-0.03 (-0.05 to -0.01)	0.12 (0.09 − 0.15)	0.59 (0.49 − 0.69)	2.05 (1.95 − 2.16)	1.59 (1.48 − 1.70)
Kiribati	0.18 (0.17 − 0.18)	0.12 (0.11 − 0.13)	-0.15 (-0.20 to -0.11)	1.13 (1.01 − 1.25)	1.14 (0.94 − 1.35)	1.14 (1.11 − 1.18)
Kuwait	0.12 (0.10 − 0.14)	0.35 (0.27 − 0.43)	-0.03 (-0.12 to 0.06)	1.21 (0.66 − 1.76)	1.36 (0.62 − 2.11)	4.03 (3.44 − 4.62)
Kyrgyzstan	-0.01 (-0.03 to 0.02)	0.20 (0.18 − 0.23)	-0.26 (-0.41 to -0.11)	-0.33 (-0.76 to 0.10)	3.13 (2.41 − 3.86)	1.15 (0.74 − 1.56)
Lao People's Democratic Republic	0.09 (0.08 − 0.11)	0.05 (0.02 − 0.09)	-0.41 (-0.48 to -0.33)	0.93 (0.82 − 1.03)	1.62 (1.49 − 1.76)	2.52 (2.43 − 2.61)
Latvia	0.04 (0.02 − 0.06)	0.19 (-0.31 to 0.70)	-0.64 (-0.72 to -0.56)	1.4 (1.21 − 1.58)	3.9 (3.34 − 4.46)	3.81 (3.55 − 4.08)
Lebanon	0.12 (0.11 − 0.13)	0.07 (0.05 − 0.10)	0.22 (0.18 − 0.25)	1.89 (1.64 − 2.15)	3.36 (3.09 − 3.64)	4.63 (4.30 − 4.95)
Lesotho	0.09 (0.08 − 0.10)	-0.07 (-0.10 to -0.04)	0.24 (0.22 − 0.27)	1.29 (1.21 − 1.38)	3.36 (3.20 − 3.51)	1.26 (1.17 − 1.35)
Liberia	0.07 (0.06 − 0.08)	0.05 (0.03 − 0.07)	0.11 (0.07 − 0.15)	0.65 (0.56 − 0.74)	3.06 (2.46 − 3.67)	3.44 (3.28 − 3.59)
Libya	0.18 (0.17 − 0.19)	0.10 (0.09 − 0.12)	0.09 (0.06 − 0.12)	2.04 (1.78 − 2.31)	2.35 (2.06 − 2.64)	2.69 (2.41 − 2.97)
Lithuania	0.05 (0.03 − 0.06)	-0.26 (-0.67 to 0.14)	-1.22 (-1.29 to -1.14)	-0.26 (-0.79 to 0.27)	2.80 (2.28 − 3.32)	3.76 (3.30 − 4.23)
Luxembourg	0.47 (0.42 − 0.52)	0.14 (-0.09 to 0.36)	-0.51 (-1.22 to 0.21)	0.30 (0.05 − 0.55)	1.61 (1.18 − 2.05)	0.70 (0.38 − 1.02)
Madagascar	-0.01 (-0.02 to 0.00)	0.08 (0.07 − 0.10)	0.05 (0.03 − 0.08)	-0.15 (-0.28 to -0.01)	0.78 (0.60 − 0.96)	0.43 (0.30 − 0.56)
Malawi	0.02 (0.01 − 0.03)	0.12 (0.11 − 0.13)	0.01 (-0.01 to 0.04)	1.31 (1.25 − 1.37)	1.18 (1.06 − 1.29)	2.50 (2.43 − 2.57)

Location	BPH	UTI	Urolithiasis	Bladder cancer	Kidney cancer	Prostate cancer
Malaysia	0.06 (0.05 − 0.08)	0.09 (0.07 − 0.11)	0.01 (-0.08 to 0.09)	1.81 (1.69 − 1.93)	1.55 (1.38 − 1.72)	2.71 (2.57 − 2.85)
Maldives	0.05 (0.04 − 0.06)	-0.26 (-0.36 to -0.15)	0.20 (0.16 − 0.24)	0.73 (0.49 − 0.98)	1.67 (1.56 − 1.77)	3.01 (2.71 − 3.30)
Mali	0.03 (0.02 − 0.04)	0.01 (0.00 − 0.02)	0.01 (-0.02 to 0.05)	0.65 (0.60 − 0.70)	0.90 (0.80 – 1.00)	1.69 (1.62 − 1.75)
Malta	0.18 (0.17 − 0.20)	-0.39 (-0.81 to 0.03)	0.47 (-0.20 to 1.14)	-0.09 (-0.20 to 0.03)	2.44 (2.18 − 2.69)	0.74 (0.48 − 1.00)
Marshall Islands	0.26 (0.24 − 0.27)	0.15 (0.13 − 0.17)	0.02 (-0.02 to 0.07)	1.69 (1.60 − 1.77)	1.17 (1.09 − 1.25)	1.77 (1.68 − 1.85)
Mauritania	0.01 (0.00 − 0.03)	0.09 (0.08 − 0.11)	0.05 (0.02 − 0.08)	0.29 (0.12 − 0.46)	3.33 (3.24 − 3.42)	3.75 (3.67 − 3.84)
Mauritius	0.20 (0.18 − 0.21)	0.07 (0.05 − 0.09)	-0.05 (-0.13 to 0.03)	-0.17 (-0.77 to 0.43)	-1.23 (-2.92 to 0.49)	2.04 (1.64 − 2.45)
Mexico	0.08 (0.02 − 0.13)	1.16 (0.80 − 1.53)	0.67 (0.01 − 1.34)	0.93 (0.74 − 1.11)	2.69 (2.55 − 2.84)	1.57 (1.23 − 1.92)
Mongolia	0.03 (0.01 − 0.05)	-0.04 (-0.07 to -0.01)	-0.04 (-0.12 to 0.03)	-1.03 (-1.42 to -0.64)	6.47 (5.97 − 6.97)	3.68 (3.32 − 4.03)
Montenegro	0.14 (0.12 − 0.17)	-0.02 (-0.03 to 0.00)	-0.17 (-0.23 to -0.12)	1.13 (1.02 − 1.23)	1.73 (1.52 − 1.94)	2.06 (1.70 − 2.42)
Morocco	0.13 (0.13 − 0.14)	0.08 (0.07 − 0.09)	0.19 (0.15 − 0.23)	2.06 (1.84 − 2.28)	2.75 (2.64 to 2.86)	3.24 (2.98 to 3.5)
Mozambique	0.02 (0.01 − 0.03)	0.11 (0.09 − 0.13)	0.12 (0.09 − 0.14)	1.09 (0.99 − 1.18)	1.72 (1.58 − 1.86)	1.51 (1.47 − 1.55)
Myanmar	0.07 (0.07 − 0.08)	0.18 (0.16 − 0.20)	-0.29 (-0.37 to -0.21)	0.82 (0.68 − 0.96)	1.62 (1.47 − 1.76)	2.93 (2.81 − 3.05)
Namibia	0.05 (0.03 − 0.07)	0.08 (0.06 − 0.09)	0.03 (0.00 − 0.05)	1.09 (0.96 − 1.22)	2.52 (2.45 − 2.60)	3.84 (3.58 − 4.10)
Nepal	0.06 (-0.01 to 0.13)	-0.34 (-0.54 to -0.14)	0.25 (0.20 − 0.30)	1.22 (1.01 − 1.42)	2.6 (2.43 − 2.76)	2.40 (2.31 − 2.49)

Location	BPH	UTI	Urolithiasis	Bladder cancer	Kidney cancer	Prostate cancer
Netherlands	0.24 (0.21 − 0.27)	0.05 (0.04 − 0.06)	-0.44 (-0.50 to -0.37)	0.13 (-0.14 to 0.40)	1.55 (1.22 − 1.88)	1.07 (0.66 − 1.47)
New Zealand	-0.35 (-0.47 to -0.23)	0.42 (0.37 − 0.47)	-0.68 (-0.99 to -0.37)	-0.94 (-1.28 to -0.60)	1.22 (1.05 − 1.39)	-0.54 (-0.76 to -0.32)
Nicaragua	0.20 (0.19 − 0.21)	0.02 (0.00 − 0.05)	-0.12 (-0.16 to -0.08)	1.71 (1.49 − 1.94)	2.6 (2.41 − 2.79)	2.63 (2.35 − 2.91)
Niger	0.03 (0.02 − 0.05)	0.15 (0.14 − 0.16)	-0.02 (-0.07 to 0.02)	-0.30 (-0.37 to -0.24)	1.42 (1.29 − 1.55)	2.84 (2.67 – 3.00)
Nigeria	0.00 (-0.01 to 0.02)	0.34 (0.27 − 0.40)	-0.27 (-0.37 to -0.18)	0.83 (0.70 − 0.95)	1.89 (1.70 − 2.08)	2.14 (1.99 − 2.29)
Northern Mariana Islands	0.13 (0.12 − 0.14)	0.19 (0.10 − 0.27)	-0.14 (-0.19 to -0.09)	3.99 (3.49 − 4.49)	0.83 (0.52 − 1.15)	1.80 (1.52 − 2.08)
Norway	0.05 (0.04 − 0.06)	-0.07 (-0.13 to -0.01)	0.07 (-0.30 to 0.44)	-0.2 (-0.53 to 0.13)	1.36 (0.81 − 1.91)	1.06 (0.49 − 1.64)
Oman	0.10 (0.09 − 0.12)	0.15 (0.09 − 0.22)	0.24 (0.23 − 0.26)	1.52 (1.24 − 1.79)	3.31 (3.02 − 3.60)	3.25 (2.99 − 3.50)
Pakistan	0.17 (0.16 − 0.18)	0.66 (0.57 − 0.75)	-0.1 (-0.22 to 0.03)	0.77 (0.66 − 0.88)	2.2 (2.11 − 2.29)	2.00 (1.93 − 2.07)
Palestine	0.13 (0.12 − 0.14)	0.06 (0.03 − 0.08)	0.31 (0.26 − 0.37)	1.15 (1.06 − 1.25)	1.29 (1.19 − 1.39)	2.32 (2.21 − 2.43)
Panama	0.20 (0.18 − 0.21)	-0.02 (-0.04 to -0.01)	-0.20 (-0.26 to -0.14)	1.12 (0.92 − 1.32)	2.58 (2.45 − 2.72)	2.13 (1.58 − 2.69)
Papua New Guinea	0.12 (0.11 − 0.14)	0.08 (0.06 − 0.09)	-0.04 (-0.12 to 0.04)	0.99 (0.94 − 1.04)	0.42 (0.30 − 0.55)	1.29 (1.24 − 1.35)
Paraguay	0.12 (0.11 − 0.14)	-0.08 (-0.10 to -0.06)	-0.04 (-0.09 to 0.02)	1.92 (1.82 − 2.02)	2.95 (2.82 − 3.08)	2.69 (2.35 − 3.03)
Peru	0.19 (0.17 − 0.21)	0.17 (0.14 − 0.19)	-0.03 (-0.09 to 0.02)	1.65 (1.47 − 1.84)	2.17 (1.92 − 2.43)	3.16 (2.99 − 3.33)
Philippines	0.04 (0.01 − 0.06)	0.51 (0.41 − 0.61)	-0.13 (-0.22 to -0.05)	0.94 (0.84 − 1.03)	0.95 (0.84 − 1.07)	1.42 (1.34 − 1.50)

Location	BPH	UTI	Urolithiasis	Bladder cancer	Kidney cancer	Prostate cancer
Plurinational State of Bolivia	0.19 (0.17 − 0.21)	0.14 (0.10 − 0.18)	0.00 (-0.07 to 0.07)	1.04 (1.00 − 1.07)	2.2 (2.17 − 2.24)	2.23 (2.15 − 2.31)
Poland	-0.82 (-1.25 to -0.39)	-1.83 (-2.24 to -1.43)	-2.93 (-3.48 to -2.37)	2.22 (1.97 − 2.48)	1.06 (0.75 − 1.37)	3.9 (3.53 − 4.27)
Portugal	0.32 (0.28 − 0.36)	-0.62 (-0.75 to -0.49)	0.31 (0.25 − 0.36)	1.77 (1.59 − 1.96)	2.73 (2.33 − 3.13)	1.66 (1.31 − 2.01)
Principality of Monaco	0.3 (0.26 − 0.34)	0.05 (0.04 − 0.07)	-0.15 (-0.21 to -0.09)	0.79 (0.69 − 0.88)	2.87 (2.49 − 3.26)	1.49 (1.33 − 1.65)
Puerto Rico	0.22 (0.19 − 0.24)	0.06 (0.05 − 0.08)	0.19 (0.11 − 0.26)	1.16 (0.94 − 1.38)	2.29 (2.15 − 2.44)	0.84 (0.57 − 1.12)
Qatar	0.29 (0.25 − 0.32)	-0.68 (-1.18 to -0.18)	0.39 (0.31 − 0.46)	1.12 (0.76 − 1.48)	1.59 (1.44 − 1.73)	3.95 (3.59 − 4.30)
Republic of Cabo Verde	0.09 (0.08 − 0.10)	-0.13 (-0.13 to -0.12)	0.24 (0.20 − 0.28)	6.39 (5.16 − 7.62)	9.44 (7.69 − 11.22)	4.36 (4.04 − 4.69)
Republic of Côte d’Ivoire	0.04 (0.03 − 0.05)	0.06 (0.05 − 0.08)	0.08 (0.04 − 0.13)	0.39 (0.32 − 0.47)	1.64 (1.46 − 1.82)	1.69 (1.62 − 1.77)
Republic of Korea	0.08 (0.07 − 0.09)	0.02 (0.00 − 0.04)	-0.01 (-0.04 to 0.03)	1.36 (1.09 − 1.64)	4.07 (3.39 − 4.75)	5.89 (5.19 − 6.58)
Republic of Moldova	0.01 (-0.01 to 0.02)	0.04 (0.02 − 0.05)	0.08 (-0.06 to 0.22)	1.14 (0.85 − 1.42)	2.13 (1.76 − 2.50)	4.32 (3.67 − 4.97)
Republic of Nauru	0.16 (0.14 − 0.17)	0.28 (0.25 − 0.30)	-0.17 (-0.20 to -0.14)	1.13 (1.06 − 1.20)	0.17 (-0.25 to 0.59)	1.55 (1.34 − 1.77)
Republic of Niue	0.24 (0.23 − 0.24)	0.05 (0.03 − 0.07)	0.09 (0.04 −0.13)	1.65 (1.54 − 1.76)	1.79 (1.59 − 1.99)	2.02 (1.94 − 2.10)
Republic of Palau	0.20 (0.19 − 0.21)	-0.16 (-0.23 to -0.09)	0.17 (0.11 − 0.23)	0.99 (0.86 − 1.12)	0.8 (0.67 − 0.93)	0.84 (0.74 − 0.93)
Republic of San Marino	0.29 (0.25 − 0.32)	0.07 (0.02 − 0.13)	0.01 (-0.02 to 0.04)	-0.42 (-0.82 to -0.02)	1.04 (0.56 − 1.53)	0.27 (-0.16 to 0.69)
Republic of the Gambia	0.05 (0.04 − 0.06)	0.21 (0.20 − 0.22)	0.04 (0.01 − 0.08)	0.44 (0.27 − 0.61)	1.21 (0.99 − 1.42)	1.71 (1.60 − 1.81)

Location	BPH	UTI	Urolithiasis	Bladder cancer	Kidney cancer	Prostate cancer
Romania	0.12 (0.11 − 0.13)	0.48 (0.24 − 0.73)	0.24 (-0.10 to 0.57)	2.45 (2.27 − 2.63)	3.63 (3.31 − 3.94)	4.45 (4.25 − 4.65)
Russian Federation	0.00 (-0.02 to 0.01)	0.30 (0.19 − 0.41)	-0.82 (-0.87 to -0.77)	0.59 (0.43 − 0.74)	1.04 (0.83 − 1.25)	4.38 (4.03 − 4.72)
Rwanda	-0.02 (-0.03 to 0.00)	0.11 (0.08 − 0.13)	-0.05 (-0.08 to -0.02)	-0.28 (-0.55 to -0.02)	1.22 (0.96 − 1.48)	2.08 (1.82 − 2.33)
Saint Kitts and Nevis	0.17 (0.16 − 0.19)	0.05 (0.04 − 0.06)	0.46 (0.39 − 0.54)	1.62 (1.36 − 1.88)	1.75 (1.58 − 1.91)	3.29 (2.79 − 3.79)
Saint Lucia	0.21 (0.20 − 0.23)	-0.02 (-0.04 to -0.01)	0.54 (0.47 − 0.61)	0.40 (0.31 − 0.49)	1.64 (1.49 − 1.79)	1.13 (0.79 − 1.47)
Saint Vincent and the Grenadines	0.21 (0.20 − 0.23)	0.04 (0.03 − 0.06)	0.87 (0.84 − 0.90)	0.94 (0.77 − 1.11)	1.31 (1.15 − 1.47)	1.33 (1.09 − 1.57)
Samoa	0.13 (0.12 − 0.15)	0.09 (0.09 to 0.1)	-0.01 (-0.06 to 0.04)	0.85 (0.80 − 0.91)	0.49 (0.40 − 0.59)	1.18 (1.13 − 1.24)
Sao Tome and Principe	0.06 (0.05 − 0.07)	0.18 (0.16 to 0.2)	0.16 (0.14 to 0.19)	1.71 (1.63 − 1.78)	1.12 (0.55 − 1.69)	2.80 (2.66 − 2.93)
Saudi Arabia	0.17 (0.16 − 0.17)	0.17 (0.14 to 0.2)	0.21 (0.18 to 0.25)	1.85 (1.72 − 1.98)	4.4 (4.21 − 4.59)	3.53 (3.36 − 3.70)
Senegal	0.08 (0.06 − 0.09)	0.08 (0.07 − 0.10)	0.04 (0.00 − 0.08)	0.10 (-0.01 to 0.20)	2.38 (2.19 − 2.57)	3.28 (3.14 − 3.42)
Serbia	0.18 (0.17 − 0.19)	-0.08 (-0.11 to -0.06)	0.38 (-0.09 to 0.85)	1.8 (1.66 − 1.93)	2.13 (1.99 − 2.27)	2.9 (2.65 − 3.15)
Seychelles	0.18 (0.16 − 0.20)	0.00 (-0.03 to 0.03)	0.49 (0.42 − 0.56)	0.85 (0.70 − 0.99)	-0.03 (-0.70 to 0.65)	2.83 (2.33 − 3.33)
Sierra Leone	0.06 (0.05 − 0.07)	0.04 (0.01 − 0.07)	0.08 (0.05 − 0.12)	0.09 (-0.01 to 0.19)	2.38 (2.17 − 2.58)	2.82 (2.68 − 2.96)
Singapore	0.03 (0.01 − 0.05)	0.06 (0.04 − 0.08)	-0.20 (-0.25 to -0.16)	0.26 (0.06 − 0.46)	2.55 (2.09 − 3.01)	3.34 (2.98 − 3.69)
Slovakia	0.10 (0.10 − 0.11)	-0.07 (-0.43 to 0.29)	-0.54 (-1.51 to 0.44)	1.05 (0.98 − 1.12)	2.52 (2.19 − 2.85)	2.82 (2.66 − 2.98)

Location	BPH	UTI	Urolithiasis	Bladder cancer	Kidney cancer	Prostate cancer
Slovenia	0.09 (0.07 − 0.11)	0.64 (0.40 − 0.89)	0.13 (-0.49 to 0.75)	1.37 (1.15 − 1.59)	2.83 (2.35 − 3.30)	3.51 (2.98 − 4.04)
Socialist Republic of Viet Nam	0.18 (0.12 − 0.24)	-0.20 (-0.24 to -0.16)	0.98 (0.78 − 1.18)	2.72 (2.67 − 2.77)	3.86 (3.65 − 4.06)	3.46 (3.34 − 3.58)
Solomon Islands	0.19 (0.17 − 0.20)	0.27 (0.25 − 0.29)	0.01 (-0.03 to 0.05)	1.49 (1.39 − 1.58)	0.93 (0.78 − 1.08)	1.61 (1.50 − 1.72)
Somalia	0.00 (-0.01 to 0.02)	0.09 (0.06 − 0.11)	0.14 (0.12 − 0.16)	-0.05 (-0.09 to -0.02)	0.21 (0.08 − 0.35)	0.47 (0.43 − 0.52)
South Africa	0.09 (0.07 − 0.12)	-0.06 (-0.08 to -0.04)	-0.14 (-0.23 to -0.06)	0.76 (0.69 − 0.83)	1.92 (1.74 − 2.11)	2.17 (2.05 − 2.29)
South Sudan	-0.01 (-0.03 to 0.01)	0.21 (0.18 − 0.25)	-0.01 (-0.05 to 0.02)	0.21 (0.02 − 0.40)	1.52 (1.24 − 1.8)	0.91 (0.86 − 0.97)
Spain	0.52 (0.38 − 0.67)	0.02 (-0.01 to 0.05)	0.95 (0.76 − 1.14)	0.10 (-0.15 to 0.34)	2.35 (2.06 to 2.64)	1.23 (0.75 − 1.71)
Sri Lanka	0.16 (0.15 − 0.17)	0.38 (0.37 − 0.39)	-0.25 (-0.31 to -0.20)	2.02 (1.79 − 2.25)	-5.26 (-6.40 to -4.10)	3.25 (3.12 − 3.38)
Sudan	0.13 (0.11 − 0.14)	0.10 (0.09 − 0.10)	0.18 (0.11 − 0.24)	1.35 (1.21 − 1.48)	2.54 (2.41 − 2.67)	3.06 (2.91 − 3.21)
Suriname	0.28 (0.26 − 0.31)	0.16 (0.14 − 0.18)	1.47 (1.38 − 1.56)	0.99 (0.82 − 1.16)	1.74 (1.60 − 1.89)	1.81 (1.59 − 2.02)
Sweden	-0.12 (-0.22 to -0.03)	-0.04 (-0.10 to 0.01)	-0.21 (-0.45 to 0.04)	0.57 (0.17 − 0.96)	-0.38 (-0.72 to -0.04)	0.19 (-0.31 to 0.69)
Switzerland	0.12 (0.11 to 0.13)	0.23 (-0.15 to 0.61)	-0.53 (-1.13 to 0.08)	1.25 (0.89 to 1.62)	1.54 (1.02 − 2.08)	-0.40 (-0.72 to -0.07)
Syrian Arab Republic	0.10 (0.09 − 0.12)	0.28 (0.21 − 0.34)	0.09 (0.01 − 0.17)	1.89 (1.67 − 2.10)	2.06 (1.90 − 2.22)	3.21 (2.98 − 3.44)
Taiwan (Province of China)	0.09 (0.09 − 0.10)	0.26 (0.24 − 0.27)	0.91 (0.72 − 1.10)	0.50 (0.10 − 0.90)	3.48 (2.79 − 4.18)	4.27 (3.65 − 4.90)
Tajikistan	0.09 (0.07 − 0.11)	0.16 (0.12 − 0.20)	0.25 (0.22 − 0.29)	-0.71 (-0.86 to -0.56)	-0.33 (-0.52 to -0.14)	-0.52 (-0.62 to -0.41)

Location	BPH	UTI	Urolithiasis	Bladder cancer	Kidney cancer	Prostate cancer
Thailand	0.02 (-0.01 to 0.04)	-0.03 (-0.05 to -0.01)	-0.80 (-0.97 to -0.62)	1.29 (1.06 − 1.53)	2.83 (2.74 − 2.91)	3.20 (3.00 − 3.39)
The former Yugoslav Republic of Macedonia	0.18 (0.15 − 0.21)	0.03 (0.02 − 0.05)	-0.15 (-0.21 to -0.09)	1.62 (1.31 − 1.93)	3.47 (2.97 − 3.98)	3.16 (2.86 − 3.46)
Timor-Leste	0.08 (0.07 − 0.10)	0.15 (0.12 − 0.18)	0.05 (-0.01 to 0.10)	1.45 (1.24 − 1.67)	1.17 (0.85 − 1.49)	2.60 (2.46 − 2.74)
Togo	0.03 (0.02 − 0.04)	0.06 (0.05 − 0.08)	0.03 (-0.01 to 0.06)	0.05 (-0.08 to 0.19)	2.48 (2.38 − 2.57)	3.03 (2.95 − 3.10)
Tokelau	0.16 (0.15 − 0.18)	0.11 (0.09 − 0.14)	0.13 (0.07 − 0.18)	1.74 (1.69 − 1.79)	2.38 (1.86 − 2.90)	2.27 (2.23 − 2.32)
Tonga	0.14 (0.12 − 0.15)	0.04 (0.01 − 0.06)	0.11 (0.05 − 0.18)	1.53 (1.39 − 1.66)	1.16 (1.08 − 1.24)	1.32 (1.25 − 1.39)
Trinidad and Tobago	0.16 (0.14 − 0.18)	0.07 (0.05 − 0.08)	2.86 (2.61 − 3.11)	1.21 (1.05 − 1.36)	1.96 (1.83 − 2.10)	1.26 (1.10 − 1.42)
Tunisia	0.14 (0.13 − 0.15)	0.12 (0.11 − 0.12)	0.17 (0.13 − 0.21)	1.69 (1.59 − 1.79)	2.12 (2.08 − 2.16)	2.99 (2.94 − 3.04)
Turkey	0.11 (0.09 − 0.13)	0.40 (0.23 − 0.58)	0.36 (0.33 − 0.39)	2.19 (2.06 − 2.32)	2.01 (1.90 − 2.12)	3.77 (3.51 − 4.03)
Turkmenistan	0.07 (0.05 − 0.09)	0.26 (0.23 − 0.29)	0.11 (0.07 − 0.14)	-0.46 (-0.77 to -0.14)	4.07 (3.60 − 4.55)	2.26 (1.91 − 2.62)
Tuvalu	0.18 (0.16 − 0.19)	-0.20 (-0.21 to -0.18)	0.12 (0.08 − 0.16)	2.06 (1.95 − 2.18)	1.91 (1.87 − 1.94)	2.38 (2.25 − 2.52)
Uganda	-0.01 (-0.02 to 0.01)	0.13 (0.12 − 0.13)	0.05 (0.02 − 0.09)	0.28 (0.16 − 0.40)	2.46 (2.37 − 2.56)	1.92 (1.84 – 2.00)
Ukraine	0.00 (-0.02 to 0.02)	0.14 (-0.17 to 0.45)	-0.54 (-0.60 to -0.48)	0.76 (0.54 − 0.99)	0.85 (0.55 − 1.14)	1.13 (0.93 − 1.33)
United Arab Emirates	0.15 (0.13 − 0.17)	-0.01 (-0.09 to 0.07)	0.32 (0.27 − 0.37)	1.14 (0.70 − 1.57)	2.82 (2.46 − 3.18)	2.88 (2.44 − 3.32)
United Kingdom of Great Britain and Northern Ireland	0.31 (0.25 − 0.37)	0.64 (-0.05 to 1.33)	0.76 (0.59 − 0.93)	-0.92 (-1.01 to -0.82)	1.95 (1.67 − 2.24)	1.4 (1.08 − 1.72)

Location	BPH	UTI	Urolithiasis	Bladder cancer	Kidney cancer	Prostate cancer
United Republic of Tanzania	0.01 (0.00 − 0.03)	0.08 (0.06 − 0.10)	0.09 (0.06 − 0.11)	-0.16 (-0.26 to -0.07)	1.66 (1.47 − 1.85)	0.91 (0.84 − 0.98)
United States of America	0.24 (0.17 − 0.32)	-0.37 (-0.42 to -0.31)	-2.16 (-2.51 to -1.81)	0.24 (0.03 − 0.45)	0.12 (-0.14 to 0.38)	-0.78 (-0.89 to -0.67)
United States Virgin Islands	0.24 (0.22 − 0.27)	0.05 (0.01 − 0.08)	2.24 (2.14 − 2.34)	-0.46 (-0.66 to -0.26)	1.59 (1.37 − 1.80)	-1.18 (-1.67 to -0.69)
Uruguay	0.27 (0.21 − 0.33)	0.20 (0.19 − 0.22)	-0.04 (-0.10 to 0.02)	0.12 (-0.01 to 0.25)	2.64 (2.46 − 2.82)	1.56 (1.12 − 1.99)
Uzbekistan	0.09 (0.07 − 0.11)	0.39 (0.32 − 0.45)	0.04 (-0.01 to 0.09)	0.63 (0.47 − 0.80)	1.53 (1.34 − 1.73)	0.86 (0.04 − 1.69)
Vanuatu	0.16 (0.14 − 0.18)	0.16 (0.15 − 0.18)	-0.05 (-0.11 to 0.00)	0.99 (0.91 − 1.07)	0.55 (0.40 − 0.71)	1.29 (1.24 − 1.33)
Yemen	0.07 (0.07 − 0.08)	0.11 (0.10 − 0.13)	0.10 (0.04 − 0.17)	1.22 (1.13 − 1.32)	1.59 (1.39 − 1.79)	2.73 (2.57 − 2.9)
Zambia	0.00 (-0.01 to 0.01)	0.10 (0.08 − 0.11)	0.08 (0.05 − 0.11)	1.55 (1.25 − 1.85)	3.26 (2.69 − 3.84)	4.36 (3.77 − 4.95)
Zimbabwe	0.11 (0.10 − 0.13)	0.01 (-0.01 to 0.03)	-0.06 (-0.09 to -0.03)	0.39 (0.16 − 0.61)	3.20 (2.49 − 3.92)	0.84 (0.56 − 1.12)

BPH benign prostatic hyperplasia, *UTI* urinary tract infections, *EAPC* estimated annual percentage change, *ASPR* age-standardized prevalence rate

Table S9 EAPC of ASMR for the 6 urologic diseases in 204 countries and territories from 1990 to 2021 (95% CI)

Location	BPH	UTI	Urolithiasis	Bladder cancer	Kidney cancer	Prostate cancer
Afghanistan	–	0.09 (-0.05 to 0.24)	4.77 (4.17 − 5.37)	-0.47 (-0.52 to -0.43)	0.71 (0.49 − 0.92)	0.58 (0.55 − 0.61)
Albania	–	-1.82 (-2.37 to -1.28)	-3.70 (-4.54 to -2.86)	-0.01 (-0.16 to 0.15)	1.64 (1.42 − 1.85)	-0.48 (-0.62 to -0.34)
Algeria	–	0.54 (0.44 − 0.64)	5.28 (4.64 − 5.93)	-0.23 (-0.45 to 0.00)	1.58 (1.47 − 1.69)	0.04 (-0.02 to 0.11)
American Samoa	–	-0.30 (-0.53 to -0.06)	-2.08 (-3.39 to -0.76)	2.17 (1.77 − 2.56)	1.20 (0.92 − 1.48)	0.54 (0.35 − 0.73)
Andorra	–	-1.11 (-1.31 to -0.92)	-0.45 (-0.77 to -0.13)	-1.34 (-1.60 to -1.08)	-0.67 (-0.85 to -0.50)	-0.62 (-0.84 to -0.41)
Angola	–	-0.45 (-0.55 to -0.35)	-0.80 (-0.89 to -0.70)	-0.16 (-0.24 to -0.07)	0.80 (0.70 − 0.90)	0.80 (0.73 − 0.87)
Antigua and Barbuda	–	4.52 (3.90 − 5.14)	3.87 (2.98 − 4.77)	0.43 (0.17 − 0.69)	0.37 (0.16 − 0.59)	0.29 (-0.11 to 0.69)
Argentina	–	7.57 (6.52 − 8.62)	2.3 (1.39 − 3.21)	-1.38 (-1.48 to -1.27)	0.89 (0.72 − 1.07)	-0.58 (-0.92 to -0.23)
Armenia	–	3.76 (2.04 − 5.51)	0.84 (-0.17 to 1.85)	0.17 (-0.06 to 0.41)	1.37 (1.09 − 1.66)	1.68 (1.42 − 1.93)
Australia	–	1.57 (0.94 − 2.19)	-0.96 (-1.68 to -0.23)	-1.15 (-1.23 to -1.07)	-0.59 (-0.67 to -0.51)	-2.9 (-3.26 to -2.53)
Austria	–	1.69 (1.01 − 2.37)	-0.76 (-1.39 to -0.12)	-1.12 (-1.23 to -1.01)	-1.16 (-1.27 to -1.06)	-1.93 (-2.06 to -1.79)
Azerbaijan	–	0.96 (0.54 − 1.38)	1.83 (1.39 − 2.27)	-0.29 (-0.43 to -0.14)	0.83 (0.68 − 0.98)	-0.25 (-0.46 to -0.04)
Bahrain	–	-0.72 (-1.56 to 0.11)	5.17 (4.40 − 5.94)	-1.94 (-2.22 to -1.66)	-0.7 (-0.92 to -0.47)	-0.74 (-1.01 to -0.47)
Bangladesh	–	-0.95 (-1.19 to -0.72)	-1.97 (-2.18 to -1.76)	-1.11 (-1.35 to -0.88)	-0.04 (-0.14 to 0.05)	-0.51 (-0.69 to -0.33)
Barbados	–	4.33 (3.36 − 5.31)	3.11 (2.41 − 3.81)	0.44 (0.27 − 0.61)	0.68 (0.55 − 0.81)	-0.05 (-0.19 to 0.09)

Location	BPH	UTI	Urolithiasis	Bladder cancer	Kidney cancer	Prostate cancer
Belarus	–	0.03 (-0.60 to 0.67)	0.10 (-0.08 to 0.28)	-1.68 (-2.09 to -1.27)	2.62 (1.77 − 3.48)	1.25 (0.99 − 1.50)
Belgium	–	4.66 (4.03 − 5.30)	-0.10 (-0.52 to 0.31)	-1.31 (-1.54 to -1.09)	-0.56 (-0.76 to -0.36)	-2.75 (-2.9 to -2.59)
Belize	–	4.56 (3.40 − 5.73)	3.77 (2.67 − 4.87)	0.83 (0.40 − 1.25)	0.96 (0.56 − 1.37)	0.95 (0.12 − 1.78)
Benin	–	-0.62 (-0.70 to -0.54)	-0.83 (-0.94 to -0.72)	-1.14 (-1.27 to -1.00)	1.93 (1.81 − 2.05)	1.77 (1.66 − 1.89)
Bermuda	–	2.81 (2.26 − 3.37)	1.80 (1.07 − 2.54)	-0.82 (-1.08 to -0.56)	-1.10 (-1.31 to -0.89)	-0.75 (-0.91 to -0.60)
Bhutan	–	0.47 (0.42 − 0.52)	-0.56 (-0.71 to -0.41)	-0.21 (-0.30 to -0.12)	0.96 (0.91 – 1.00)	0.10 (0.04 − 0.17)
Bolivarian Republic of Venezuela	–	3.54 (2.90 − 4.19)	0.98 (0.30 − 1.68)	-0.21 (-0.34 to -0.09)	0.45 (0.18 − 0.71)	0.53 (0.15 − 0.91)
Bosnia and Herzegovina	–	-2.48 (-3.19 to -1.76)	-3.75 (-4.36 to -3.14)	1.10 (0.96 − 1.25)	2.76 (2.47 − 3.06)	1.05 (0.90 − 1.20)
Botswana	–	0.31 (0.06 − 0.55)	-0.60 (-0.94 to -0.26)	-0.79 (-0.96 to -0.62)	1.39 (1.26 − 1.52)	0.33 (0.06 − 0.61)
Brazil	–	4.4 (4.01 − 4.80)	3.67 (3.45 − 3.89)	-0.22 (-0.28 to -0.17)	1.21 (1.12 − 1.30)	-0.08 (-0.40 to 0.25)
Brunei Darussalam	–	1.8 (1.27 − 2.32)	0.78 (0.34 − 1.24)	-0.15 (-0.43 to 0.13)	0.60 (0.41 − 0.79)	0.98 (0.67 − 1.29)
Bulgaria	–	-2.73 (-4.16 to -1.28)	-5.86 (-6.80 to -4.91)	1.25 (1.12 − 1.38)	4.14 (3.67 − 4.62)	1.28 (1.11 − 1.45)
Burkina Faso	–	-0.66 (-0.79 to -0.52)	-0.53 (-0.65 to -0.42)	-0.98 (-1.12 to -0.84)	2.06 (1.97 − 2.15)	1.95 (1.84 − 2.05)
Burundi	–	-1.22 (-1.37 to -1.06)	-1.20 (-1.39 to -1.02)	-1.30 (-1.44 to -1.15)	-0.32 (-0.41 to -0.24)	-0.4 (-0.49 to -0.32)
Cambodia	–	0.05 (-0.03 to 0.12)	-0.12 (-0.19 to -0.04)	-0.24 (-0.44 to -0.05)	0.87 (0.77 − 0.96)	1.24 (1.15 − 1.32)

Location	BPH	UTI	Urolithiasis	Bladder cancer	Kidney cancer	Prostate cancer
Cameroon	–	-1.24 (-1.34 to -1.15)	-0.83 (-1.00 to -0.66)	-0.73 (-0.87 to -0.59)	1.86 (1.76 − 1.96)	1.75 (1.65 − 1.85)
Canada	–	1.59 (1.18 − 1.99)	2.02 (1.62 − 2.41)	-1.33 (-1.46 to -1.20)	0.37 (0.14 − 0.59)	-3.15 (-3.36 to -2.93)
Central African Republic	–	-0.35 (-0.40 to -0.29)	-0.25 (-0.32 to -0.17)	-0.48 (-0.52 to -0.44)	0.10 (0.07 − 0.13)	0.37 (0.34 − 0.40)
Chad	–	-0.53 (-0.64 to -0.43)	0.21 (-0.04 to 0.45)	0.01 (-0.16 to 0.18)	2.62 (2.47 − 2.76)	2.28 (2.11 − 2.45)
Chile	–	1.18 (0.94 − 1.43)	-0.05 (-0.43 to 0.33)	-0.66 (-0.78 to -0.53)	0.15 (0.03 − 0.28)	-0.09 (-0.45 to 0.28)
China	–	-2.93 (-3.35 to -2.51)	-4.40 (-4.66 to -4.15)	-1.60 (-1.73 to -1.46)	0.41 (0.25 − 0.57)	-0.34 (-0.56 to -0.12)
Colombia	–	3.03 (2.35 − 3.72)	-1.04 (-1.29 to -0.78)	-1.45 (-1.64 to -1.25)	0.33 (0.19 − 0.46)	-1.43 (-1.70 to -1.16)
Commonwealth of the Bahamas	–	4.53 (3.83 − 5.24)	2.42 (1.84 − 3.01)	0.66 (0.53 − 0.79)	0.53 (0.35 − 0.70)	0.39 (0.12 − 0.65)
Comoros	–	-0.35 (-0.49 to -0.21)	-0.47 (-0.62 to -0.32)	-0.38 (-0.48 to -0.29)	0.55 (0.47 − 0.63)	0.32 (0.27 − 0.36)
Congo	–	-0.77 (-0.91 to -0.64)	-0.84 (-1.00 to -0.67)	-0.56 (-0.71 to -0.41)	0.73 (0.60 − 0.85)	0.20 (0.11 − 0.29)
Cook Islands	–	-1.62 (-1.87 to -1.36)	-3.37 (-3.72 to -3.02)	-0.16 (-0.27 to -0.06)	-1.01 (-1.25 to -0.78)	-0.40 (-0.47 to -0.33)
Costa Rica	–	3.40 (3.00 − 3.80)	-0.12 (-0.41 to 0.18)	-0.8 (-0.96 to -0.64)	1.80 (1.67 − 1.93)	0.63 (0.18 − 1.08)
Croatia	–	1.89 (1.04 − 2.75)	-5.23 (-6.22 to -4.22)	0.25 (0.07 − 0.43)	0.81 (0.51 − 1.11)	-0.53 (-0.74 to -0.33)
Cuba	–	4.82 (4.28 − 5.35)	2.54 (1.99 − 3.09)	0.45 (0.28 − 0.62)	0.53 (0.39 − 0.67)	0.73 (0.64 − 0.82)
Cyprus	–	-1.94 (-2.30 to -1.58)	-2.97 (-3.49 to -2.45)	-1.84 (-1.96 to -1.72)	0.11 (-0.13 to 0.35)	-1.85 (-2.23 to -1.47)

Location	BPH	UTI	Urolithiasis	Bladder cancer	Kidney cancer	Prostate cancer
Czech Republic	–	-1.09 (-2.16 to -0.01)	-5.99 (-6.89 to -5.07)	-0.38 (-0.49 to -0.26)	-0.03 (-0.42 to 0.36)	-0.85 (-1.19 to -0.51)
Democratic People's Republic of Korea	–	-0.80 (-0.89 to -0.72)	-1.03 (-1.10 to -0.95)	-0.45 (-0.52 to -0.38)	0.25 (0.17 – 0.34)	0.42 (0.32 – 0.52)
Democratic Republic of the Congo	–	-0.57 (-0.69 to -0.46)	-0.31 (-0.42 to -0.20)	-0.31 (-0.50 to -0.12)	0.09 (-0.19 to 0.36)	0.64 (0.48 – 0.80)
Denmark	–	2.85 (2.11 – 3.60)	-0.23 (-0.45 to -0.01)	-1.33 (-1.90 to -0.77)	0.07 (-0.43 to 0.57)	-0.72 (-0.89 to -0.54)
Djibouti	–	0.61 (0.43 – 0.79)	0.47 (0.29 – 0.65)	0.06 (-0.01 to 0.12)	1.27 (1.20 – 1.34)	0.32 (0.25 – 0.39)
Dominica	–	2.65 (2.23 – 3.08)	2.64 (2.39 – 2.89)	0.12 (0.06 – 0.19)	0.83 (0.77 – 0.90)	0.12 (-0.18 to 0.43)
Dominican Republic	–	1.08 (0.73 – 1.43)	1.26 (0.97 – 1.55)	0.47 (0.28 – 0.66)	1.19 (1.04 – 1.33)	0.25 (-0.14 to 0.64)
Ecuador	–	3.78 (3.16 – 4.39)	1.94 (1.23 – 2.66)	0.53 (0.24 – 0.81)	0.75 (0.44 – 1.06)	0.17 (-0.22 to 0.57)
Egypt	–	4.57 (3.97 – 5.19)	4.49 (3.82 – 5.16)	-2.49 (-2.75 to -2.23)	2.31 (2.02 – 2.60)	2.58 (2.25 – 2.92)
El Salvador	–	0.67 (0.49 – 0.85)	-0.29 (-0.49 to -0.09)	0.23 (0.13 – 0.33)	1.08 (1.00 – 1.16)	0.47 (0.21 – 0.72)
Equatorial Guinea	–	0.68 (0.37 – 0.99)	0.27 (-0.13 to 0.68)	-0.01 (-0.22 to 0.20)	2.37 (2.13 – 2.61)	0.90 (0.84 – 0.97)
Eritrea	–	0.26 (0.18 – 0.35)	-0.44 (-0.52 to -0.35)	-0.07 (-0.13 to 0.00)	0.8 (0.73 – 0.87)	0.42 (0.30 – 0.54)
Estonia	–	-2.05 (-2.97 to -1.11)	-0.93 (-1.51 to -0.34)	-0.78 (-1.00 to -0.56)	1.16 (0.70 – 1.63)	1.57 (0.87 – 2.26)
Ethiopia	–	-2.02 (-2.26 to -1.78)	-2.64 (-2.79 to -2.49)	-1.06 (-1.20 to -0.92)	-0.37 (-0.57 to -0.17)	0.02 (-0.10 to 0.14)
Federated States of Micronesia	–	-0.32 (-0.42 to -0.21)	-1.82 (-1.99 to -1.64)	0.40 (0.36 – 0.44)	0.44 (0.38 – 0.50)	0.66 (0.62 – 0.71)

Location	BPH	UTI	Urolithiasis	Bladder cancer	Kidney cancer	Prostate cancer
Fiji	–	1.60 (1.40 − 1.79)	6.02 (4.96 − 7.09)	1.1 (0.95 − 1.26)	-0.42 (-0.66 to -0.18)	0.32 (0.15 − 0.49)
Finland	–	-2.99 (-3.63 to -2.35)	0.31 (0.06 − 0.56)	-1.89 (-2.01 to -1.76)	-0.80 (-0.89 to -0.70)	-1.82 (-2.00 to -1.63)
France	–	0.13 (-0.13 to 0.39)	-1.07 (-1.28 to -0.86)	-0.90 (-0.94 to -0.87)	-0.08 (-0.17 to 0.01)	-2.69 (-2.89 to -2.49)
Gabon	–	0.32 (0.13 − 0.51)	0.45 (0.22 − 0.69)	-0.43 (-0.49 to -0.37)	1.37 (1.28 − 1.46)	0.44 (0.41 − 0.46)
Georgia	–	4.2 (3.32 − 5.09)	3.50 (2.70 − 4.30)	1.13 (0.51 − 1.75)	2.76 (2.36 − 3.16)	4.07 (3.21 − 4.93)
Germany	–	4.66 (4.21 − 5.11)	0.73 (0.17 − 1.29)	-1.52 (-1.73 to -1.31)	-0.8 (-0.94 to -0.66)	-1.83 (-2.10 to -1.57)
Ghana	–	2.12 (1.93 − 2.30)	0.35 (0.23 − 0.47)	-0.45 (-0.56 to -0.33)	3.19 (3.06 − 3.33)	0.07 (-0.09 to 0.23)
Greece	–	2.29 (0.90 − 3.69)	-0.81 (-1.64 to 0.04)	-1.01 (-1.20 to -0.83)	-0.08 (-0.24 to 0.08)	-1.39 (-1.69 to -1.09)
Greenland	–	-0.87 (-0.96 to -0.79)	0.22 (0.00 − 0.44)	-1.21 (-1.39 to -1.03)	-0.09 (-0.21 to 0.03)	-1.06 (-1.13 to -0.98)
Grenada	–	3.35 (2.76 − 3.94)	1.81 (1.42 − 2.21)	0.53 (0.14 − 0.92)	1.31 (1.05 − 1.58)	2.11 (0.57 − 3.68)
Guam	–	-2.62 (-3.24 to -2.00)	-9.74 (-11.01 to -8.46)	0.80 (0.44 − 1.17)	0.24 (-0.17 to 0.65)	-0.41 (-0.70 to -0.12)
Guatemala	–	3.08 (2.59 − 3.58)	-1.21 (-1.53 to -0.89)	-1.41 (-1.64 to -1.18)	0.25 (-0.11 to 0.61)	0.62 (-0.12 to 1.37)
Guinea	–	-0.48 (-0.52 to -0.43)	0.13 (0.04 − 0.23)	0.48 (0.42 − 0.54)	0.69 (0.63 − 0.75)	1.00 (0.93 − 1.06)
Guinea-Bissau	–	-1.03 (-1.07 to -0.99)	-1.01 (-1.05 to -0.96)	-0.84 (-0.93 to -0.75)	1.53 (1.41 − 1.65)	1.91 (1.79 − 2.03)
Guyana	–	4.08 (3.29 − 4.88)	3.45 (2.51 − 4.40)	0.52 (0.33 − 0.72)	0.90 (0.73 − 1.06)	0.59 (0.29 − 0.88)

Location	BPH	UTI	Urolithiasis	Bladder cancer	Kidney cancer	Prostate cancer
Haiti	–	0.97 (0.83 − 1.10)	0.69 (0.53 − 0.84)	0.04 (-0.02 to 0.10)	-0.04 (-0.10 to 0.02)	0.44 (0.39 − 0.48)
Honduras	–	1.71 (1.52 − 1.90)	1.17 (0.97 − 1.36)	1.39 (1.25 − 1.54)	1.54 (1.40 − 1.68)	1.04 (0.96 − 1.13)
Hungary	–	-0.79 (-1.71 to 0.14)	-4.63 (-5.37 to -3.89)	-0.15 (-0.39 to 0.09)	-0.13 (-0.38 to 0.13)	-1.18 (-1.41 to -0.94)
Iceland	–	3.72 (2.99 − 4.46)	1.32 (1.13 − 1.50)	-1.27 (-1.42 to -1.12)	-0.08 (-0.30 to 0.14)	-1.17 (-1.35 to -0.99)
India	–	0.94 (0.79 − 1.09)	-0.95 (-1.22 to -0.68)	-0.15 (-0.32 to 0.01)	1.10 (0.99 − 1.21)	0.43 (0.26 − 0.60)
Indonesia	–	1.25 (1.13 − 1.37)	0.66 (0.57 − 0.75)	0.14 (0.12 − 0.17)	1.46 (1.42 − 1.50)	1.57 (1.48 − 1.65)
Iraq	–	-0.41 (-0.63 to -0.19)	-0.23 (-0.40 to -0.05)	0.43 (0.27 − 0.60)	0.70 (0.54 − 0.86)	1.02 (0.82 − 1.21)
Ireland	–	0.72 (0.47 − 0.97)	-1.55 (-1.98 to -1.11)	-1.11 (-1.3 0to -0.93)	-0.11 (-0.33 to 0.10)	-2.15 (-2.46 to -1.83)
Islamic Republic of Iran	–	-0.81 (-0.90 to -0.72)	-1.75 (-1.86 to -1.65)	-0.03 (-0.12 to 0.06)	1.05 (0.93 − 1.17)	0.85 (0.68 − 1.01)
Israel	–	2.5 (1.40 − 3.61)	0.62 (0.00 − 1.24)	-0.95 (-1.20 to -0.69)	-0.60 (-0.90 to -0.29)	-2.37 (-2.75 to -2.00)
Italy	–	3.42 (2.71 − 4.14)	-2.85 (-3.48 to -2.22)	-1.27 (-1.33 to -1.20)	-0.23 (-0.33 to -0.13)	-1.31 (-1.55 to -1.08)
Jamaica	–	4.28 (3.32 − 5.25)	4.66 (3.57 − 5.76)	0.41 (0.12 − 0.70)	0.72 (0.40 − 1.04)	1.59 (0.93 − 2.27)
Japan	–	2.66 (2.22 − 3.11)	3.88 (3.69 − 4.08)	-0.25 (-0.32 to -0.18)	0.34 (0.17 − 0.52)	-0.13 (-0.42 to 0.16)
Jordan	–	0.26 (0.09 − 0.42)	3.34 (2.84 − 3.84)	-0.99 (-1.12 to -0.86)	0.75 (0.62 − 0.88)	0.07 (-0.07 to 0.21)
Kazakhstan	–	0.69 (0.19 − 1.21)	2.26 (1.73 − 2.79)	-1.3 (-1.78 to -0.83)	-0.60 (-1.14 to -0.06)	0.56 (0.27 − 0.85)

Location	BPH	UTI	Urolithiasis	Bladder cancer	Kidney cancer	Prostate cancer
Kenya	–	1.53 (1.42 – 1.64)	1.20 (1.01 – 1.38)	1.09 (0.97 – 1.22)	1.98 (1.80 – 2.15)	2.02 (1.79 – 2.25)
Kingdom of Eswatini	–	0.55 (-0.01 to 1.11)	1.25 (0.43 – 2.08)	0.36 (0.01 – 0.72)	1.40 (1.04 – 1.77)	0.90 (0.67 – 1.13)
Kiribati	–	-0.25 (-0.40 to -0.09)	-1.41 (-1.62 to -1.20)	0.49 (0.41 – 0.57)	0.83 (0.60 – 1.05)	0.51 (0.47 – 0.54)
Kuwait	–	7.00 (5.20 – 8.84)	22.50 (19.37 – 25.71)	-0.43 (-0.91 to 0.06)	0.41 (-0.16 to 0.98)	1.68 (1.05 – 2.31)
Kyrgyzstan	–	1.46 (0.34 – 2.58)	0.72 (0.11 – 1.33)	-1.48 (-1.86 to -1.09)	2.24 (1.61 – 2.88)	-0.34 (-0.80 to 0.12)
Lao People's Democratic Republic	–	-0.84 (-0.94 to -0.73)	-1.29 (-1.39 to -1.19)	-0.74 (-0.85 to -0.64)	0.44 (0.41 – 0.46)	0.63 (0.60 – 0.67)
Latvia	–	-1.31 (-2.09 to -0.52)	-0.46 (-0.86 to -0.06)	0.54 (0.33 – 0.74)	3.21 (2.60 – 3.82)	2.36 (2.07 – 2.64)
Lebanon	–	-0.31 (-0.43 to -0.19)	-1.00 (-1.23 to -0.77)	-0.36 (-0.58 to -0.14)	1.68 (1.37 – 1.99)	1.23 (0.93 – 1.52)
Lesotho	–	2.80 (2.30 – 3.30)	3.88 (3.31 – 4.45)	1.41 (1.15 – 1.68)	2.96 (2.70 – 3.23)	1.19 (1.01 – 1.37)
Liberia	–	-0.86 (-1.28 to -0.44)	-0.52 (-0.80 to -0.24)	-0.92 (-1.05 to -0.79)	1.92 (1.54 – 2.30)	1.71 (1.61 – 1.81)
Libya	–	1.80 (1.50 – 2.10)	7.55 (6.68 – 8.43)	0.66 (0.48 – 0.84)	0.98 (0.85 – 1.12)	0.49 (0.40 – 0.58)
Lithuania	–	-0.14 (-1.05 to 0.79)	-0.53 (-1.04 to -0.02)	-0.83 (-1.55 to -0.11)	2.39 (1.83 – 2.96)	2.19 (1.69 – 2.69)
Luxembourg	–	3.13 (2.70 – 3.56)	-0.44 (-0.70 to -0.19)	-1.17 (-1.30 to -1.03)	-0.47 (-0.66 to -0.27)	-2.22 (-2.34 to -2.10)
Madagascar	–	-0.58 (-0.68 to -0.48)	-0.44 (-0.51 to -0.37)	-0.63 (-0.74 to -0.53)	0.16 (0.00 – 0.32)	-0.43 (-0.56 to -0.31)
Malawi	–	-0.24 (-0.41 to -0.08)	-0.43 (-0.63 to -0.24)	0.32 (0.16 – 0.47)	0.46 (0.39 – 0.52)	1.12 (0.99 – 1.26)

Location	BPH	UTI	Urolithiasis	Bladder cancer	Kidney cancer	Prostate cancer
Malaysia	–	1.00 (0.80 – 1.19)	0.99 (0.79 – 1.20)	-0.06 (-0.20 to 0.08)	0.31 (0.12 – 0.50)	0.26 (0.03 – 0.49)
Maldives	–	-1.74 (-1.90 to -1.59)	-2.49 (-2.59 to -2.39)	-1.88 (-1.98 to -1.78)	-1.05 (-1.19 to -0.92)	-0.62 (-0.74 to -0.51)
Mali	–	-1.03 (-1.23 to -0.82)	-0.93 (-1.06 to -0.80)	0.07 (0.01 – 0.14)	0.54 (0.46 – 0.62)	0.68 (0.58 – 0.77)
Malta	–	2.24 (1.89 – 2.59)	-1.18 (-1.42 to -0.94)	-1.73 (-1.87 to -1.59)	-0.15 (-0.31 to 0.01)	-2.06 (-2.24 to -1.87)
Marshall Islands	–	0.26 (0.16 – 0.35)	-0.85 (-1.08 to -0.63)	0.98 (0.85 – 1.12)	1.08 (1.01 – 1.16)	0.88 (0.81 – 0.95)
Mauritania	–	-1.31 (-1.48 to -1.15)	-1.36 (-1.57 to -1.15)	-0.98 (-1.12 to -0.84)	1.98 (1.92 – 2.04)	1.80 (1.71 – 1.90)
Mauritius	–	6.21 (5.77 – 6.64)	2.58 (0.58 – 4.62)	-1.53 (-2.32 to -0.73)	-1.82 (-3.47 to -0.15)	0.25 (-0.01 to 0.50)
Mexico	–	2.78 (1.98 – 3.57)	-0.82 (-0.92 to -0.73)	-0.57 (-0.73 to -0.42)	1.02 (0.94 – 1.10)	-0.39 (-0.54 to -0.23)
Mongolia	–	-1.59 (-1.87 to -1.30)	-1.79 (-2.16 to -1.42)	-2.87 (-3.34 to -2.40)	4.53 (4.13 – 4.94)	1.36 (1.29 – 1.44)
Montenegro	–	0.56 (0.24 – 0.89)	-0.23 (-0.52 to 0.05)	0.81 (0.62 – 1.00)	1.21 (1.04 – 1.39)	0.79 (0.37 – 1.22)
Morocco	–	1.11 (1.05 – 1.16)	6.35 (5.68 – 7.03)	0.42 (0.28 – 0.56)	1.35 (1.22 – 1.49)	0.73 (0.58 – 0.87)
Mozambique	–	1.11 (0.92 – 1.30)	1.10 (0.88 – 1.32)	0.6 (0.49 – 0.70)	1.31 (1.19 – 1.44)	1.02 (0.94 – 1.10)
Myanmar	–	-0.54 (-0.69 to -0.39)	-0.91 (-1.05 to -0.78)	-1.10 (-1.20 to -1.00)	0.34 (0.31 – 0.36)	0.70 (0.67 – 0.72)
Namibia	–	0.06 (-0.26 to 0.38)	-0.14 (-0.56 to 0.29)	0.11 (-0.11 to 0.33)	1.00 (0.86 – 1.14)	2.28 (2.05 – 2.51)
Nepal	–	1.19 (0.79 – 1.59)	0.01 (-0.49 to 0.52)	-0.15 (-0.39 to 0.09)	1.11 (0.94 – 1.29)	0.26 (0.10 – 0.42)

Location	BPH	UTI	Urolithiasis	Bladder cancer	Kidney cancer	Prostate cancer
Netherlands	–	0.26 (-0.28 to 0.81)	-1.05 (-1.39 to -0.70)	-0.97 (-1.11 to -0.83)	-0.29 (-0.41 to -0.17)	-2.06 (-2.24 to -1.88)
New Zealand	–	0.63 (-0.40 to 1.67)	-0.14 (-0.70 to 0.43)	-1.13 (-1.43 to -0.84)	-0.12 (-0.24 to 0.00)	-1.89 (-2.12 to -1.67)
Nicaragua	–	1.86 (1.61 − 2.10)	0.46 (0.28 − 0.64)	0.22 (-0.03 to 0.48)	1.18 (0.98v1.38)	-0.22 (-0.53 to 0.10)
Niger	–	-1.46 (-1.63 to -1.28)	-1.43 (-1.55 to -1.31)	-0.83 (-0.93 to -0.73)	1.25 (1.18 − 1.33)	1.97 (1.85 − 2.10)
Nigeria	–	-0.69 (-0.83 to -0.54)	-0.66 (-0.76 to -0.57)	0.29 (0.24 − 0.34)	1.03 (0.93 − 1.13)	0.65 (0.58 − 0.72)
Northern Mariana Islands	–	-0.65 (-1.01 to -0.28)	-8.25 (-9.56 to -6.93)	4.00 (3.38 − 4.63)	1.02 (0.78 − 1.26)	1.15 (0.91 − 1.40)
Norway	–	1.36 (0.94 − 1.78)	-0.80 (-1.33 to -0.25)	-1.32 (-1.44 to -1.20)	-0.70 (-0.97 to -0.42)	-1.54 (-1.74 to -1.34)
Oman	–	1.5 (1.11 − 1.89)	5.03 (4.39 − 5.68)	-0.20 (-0.42 to 0.01)	1.49 (1.22 − 1.76)	0.36 (0.14 − 0.58)
Pakistan	–	0.98 (0.72 − 1.24)	-0.13 (-0.34 to 0.07)	0.18 (0.02 − 0.35)	0.95 (0.78 − 1.12)	0.76 (0.57 − 0.95)
Palestine	–	-0.29 (-0.41 to -0.16)	-1.2 (-1.51 to -0.90)	-0.93 (-1.17 to -0.69)	-0.04 (-0.14 to 0.06)	-0.22 (-0.50 to 0.06)
Panama	–	3.55 (3.22 − 3.87)	-0.01 (-0.26 to 0.23)	-0.11 (-0.32 to 0.10)	0.93 (0.76 to 1.10)	0.01 (-0.52 to 0.55)
Papua New Guinea	–	-0.29 (-0.34 to -0.24)	-2.13 (-2.24 to -2.02)	0.51 (0.47 − 0.56)	0.48 (0.43 − 0.53)	0.88 (0.84 − 0.92)
Paraguay	–	1.65 (1.39 − 1.90)	5.48 (5.15 − 5.80)	1.14 (1.04 − 1.23)	1.91 (1.77 − 2.06)	1.6 (1.25 − 1.94)
Peru	–	1.31 (0.94 − 1.69)	0.91 (0.61 − 1.20)	-0.75 (-0.97 to -0.52)	-0.03 (-0.27 to 0.20)	-0.27 (-0.40 to -0.15)
Philippines	–	0.85 (0.70 − 0.99)	0.68 (0.58 − 0.79)	0.21 (0.16 − 0.26)	0.98 (0.92 − 1.04)	0.62 (0.54 − 0.70)

Location	BPH	UTI	Urolithiasis	Bladder cancer	Kidney cancer	Prostate cancer
Plurinational State of Bolivia	–	1.05 (0.96 − 1.15)	0.23 (0.17 − 0.29)	-0.25 (-0.30 to -0.21)	0.78 (0.74 − 0.82)	0.19 (0.15 − 0.24)
Poland	–	3.27 (0.78 − 5.82)	-7.46 (-9.12 to -5.76)	0.63 (0.48 − 0.78)	-0.29 (-0.41 to -0.17)	0.99 (0.63 − 1.36)
Portugal	–	7.05 (6.27 − 7.84)	-1.1 (-1.46 to -0.73)	-0.72 (-0.84 to -0.60)	0.02 (-0.22 to 0.26)	-2.11 (-2.35 to -1.87)
Principality of Monaco	–	1.82 (1.42 − 2.22)	1.47 (1.07 − 1.88)	0.34 (0.31 − 0.38)	1.33 (1.07 − 1.60)	-0.23 (-0.28 to -0.18)
Puerto Rico	–	2.26 (1.29 − 3.24)	4.78 (3.63 − 5.95)	-0.85 (-1.03 to -0.66)	0.20 (0.03 to 0.37)	-2.21 (-2.43 to -1.99)
Qatar	–	-4.69 (-5.54 to -3.84)	-2.39 (-3.47 to -1.29)	-1.85 (-2.36 to -1.34)	-0.78 (-1.11 to -0.45)	-0.89 (-1.35 to -0.43)
Republic of Cabo Verde	–	0.58 (0.37 − 0.79)	1.1 (0.56 − 1.65)	5.40 (3.94 to 6.88)	8.97 (6.90 − 11.08)	2.24 (1.80 − 2.68)
Republic of Côte d'Ivoire	–	-0.59 (-0.67 to -0.50)	-0.75 (-0.95 to -0.55)	-0.32 (-0.44 to -0.21)	0.57 (0.50 − 0.64)	0.25 (0.16 − 0.35)
Republic of Korea	–	0.87 (0.38 − 1.36)	-0.32 (-0.52 to -0.12)	-1.49 (-1.63 to -1.36)	0.65 (0.32 − 0.98)	0.57 (0.22 − 0.92)
Republic of Moldova	–	-1.04 (-1.58 to -0.49)	0.08 (-0.23 to 0.40)	-0.62 (-0.84 to -0.39)	0.83 (0.51 − 1.15)	1.9 (1.31 − 2.49)
Republic of Nauru	–	-0.20 (-0.30 to -0.11)	-2.31 (-2.53 to -2.10)	0.00 (-0.10 to 0.10)	-0.41 (-0.56 to -0.26)	0.66 (0.60 − 0.72)
Republic of Niue	–	-0.15 (-0.23 to -0.06)	-1.35 (-1.46 to -1.23)	0.65 (0.57 to 0.73)	1.06 (0.97 − 1.15)	0.59 (0.55 − 0.62)
Republic of Palau	–	-0.09 (-0.14 to -0.03)	-1.45 (-1.54 to -1.36)	-0.16 (-0.24 to -0.08)	0.38 (0.25 − 0.50)	-0.62 (-0.69 to -0.55)
Republic of San Marino	–	0.59 (0.11 − 1.07)	-1.57 (-1.82 to -1.32)	-1.18 (-1.60 to -0.77)	-0.47 (-0.87 to -0.07)	-1.43 (-1.90 to -0.96)
Republic of the Gambia	–	-0.11 (-0.26 to 0.04)	-0.05 (-0.21 to 0.11)	0.13 (0.04 − 0.23)	0.85 (0.7 − 1.00)	0.80 (0.72 − 0.87)

Location	BPH	UTI	Urolithiasis	Bladder cancer	Kidney cancer	Prostate cancer
Romania	–	-1.14 (-1.98 to -0.29)	-3.41 (-3.84 to -2.98)	0.74 (0.62 – 0.86)	2.07 (1.86 – 2.29)	1.26 (1.10 – 1.42)
Russian Federation	–	0.61 (-0.45 to 1.69)	0.37 (-0.27 to 1.01)	-0.74 (-1.07 to -0.41)	0.12 (-0.07 to 0.31)	2.27 (2.15 – 2.39)
Rwanda	–	-2.22 (-2.55 to -1.89)	-2.52 (-2.87 to -2.16)	-1.68 (-1.91 to -1.45)	-0.23 (-0.44 to -0.03)	-0.28 (-0.40 to -0.17)
Saint Kitts and Nevis	–	4.06 (3.35 – 4.77)	2.57 (2.03 – 3.12)	0.27 (0.05 – 0.49)	0.45 (0.23 – 0.68)	0.46 (0.29 – 0.63)
Saint Lucia	–	3.01 (2.42 – 3.59)	2.18 (1.47 – 2.90)	-1.11 (-1.39 to -0.84)	-0.07 (-0.38 to 0.24)	-0.91 (-1.23 to -0.58)
Saint Vincent and the Grenadines	–	3.43 (3.20 – 3.67)	0.88 (0.28 – 1.47)	0.38 (0.12 – 0.63)	0.18 (-0.03 to 0.39)	0.80 (0.51 – 1.09)
Samoa	–	-0.77 (-0.87 to -0.66)	-1.49 (-1.67 to -1.32)	-0.22 (-0.25 to -0.18)	-0.06 (-0.13 to 0.01)	-0.20 (-0.26 to -0.14)
Sao Tome and Principe	–	0.03 (-0.08 to 0.13)	0.12 (-0.01 to 0.26)	0.98 (0.87 – 1.09)	0.70 (0.43 – 0.96)	1.11 (0.91 – 1.31)
Saudi Arabia	–	-0.03 (-0.33 to 0.28)	7.69 (6.57 – 8.82)	-0.84 (-1.00 to -0.69)	2.19 (2.00 – 2.39)	-0.65 (-0.96 to -0.35)
Senegal	–	-0.6 (-0.73 to -0.46)	-0.59 (-0.73 to -0.45)	-0.67 (-0.76 to -0.57)	1.88 (1.74 – 2.01)	2.20 (2.08 to 2.31)
Serbia	–	-1.37 (-1.70 to -1.03)	-1.95 (-2.16 to -1.73)	-0.42 (-0.49 to -0.36)	0.23 (0.16 – 0.30)	-0.17 (-0.31 to -0.02)
Seychelles	–	2.96 (2.48 – 3.44)	1.90 (1.34 – 2.46)	-0.3 (-0.52 to -0.07)	-0.62 (-1.29 to 0.05)	0.98 (0.59 – 1.38)
Sierra Leone	–	-0.90 (-0.94 to -0.86)	-0.55 (-0.62 to -0.49)	-0.8 (-0.88 to -0.71)	1.73 (1.60 – 1.85)	1.8 (1.68 – 1.93)
Singapore	–	-0.44 (-0.90 to 0.02)	1.38 (1.19 – 1.56)	-1.54 (-1.78 to -1.30)	-0.13 (-0.55 to 0.28)	-0.62 (-0.85 to -0.38)
Slovakia	–	-0.75 (-1.07 to -0.43)	-3.49 (-3.82 to -3.15)	-0.36 (-0.43 to -0.28)	1.13 (0.87 –1.40)	0.48 (0.39 – 0.58)

Location	BPH	UTI	Urolithiasis	Bladder cancer	Kidney cancer	Prostate cancer
Slovenia	–	-0.20 (-1.67 to 1.29)	-5.04 (-6.04 to -4.04)	-0.04 (-0.21 to 0.12)	1.17 (0.83 − 1.50)	0.48 (0.14 − 0.83)
Socialist Republic of Viet Nam	–	0.37 (0.32 − 0.42)	0.31 (0.21 − 0.41)	0.40 (0.35 to 0.46)	2.32 (2.12 − 2.51)	0.85 (0.71 − 0.99)
Solomon Islands	–	-0.15 (-0.18 to -0.11)	-1.68 (-1.79 to -1.57)	0.46 (0.41 − 0.50)	0.40 (0.35 − 0.45)	0.72 (0.69 − 0.75)
Somalia	–	0.07 (-0.05 to 0.19)	-0.25 (-0.33 to -0.16)	-0.24 (-0.29 to -0.20)	-0.07 (-0.14 to 0.01)	-0.09 (-0.15 to -0.03)
South Africa	–	-0.03 (-0.57 to 0.52)	0.68 (0.07 − 1.29)	0.31 (0.09 − 0.53)	1.17 (1.05 − 1.30)	0.92 (0.73 − 1.11)
South Sudan	–	0.20 (-0.01 to 0.42)	-0.08 (-0.18 to 0.01)	-0.18 (-0.29 to -0.07)	0.80 (0.66 − 0.94)	-0.04 (-0.09 to 0.01)
Spain	–	3.17 (2.85 − 3.50)	-1.03 (-1.41 to -0.65)	-0.98 (-1.11 to -0.86)	0.59 (0.51 − 0.67)	-2.15 (-2.30 to -2.00)
Sri Lanka	–	1.86 (1.44 − 2.29)	1.64 (1.20 − 2.09)	-0.19 (-0.39 to 0.02)	-6.62 (-7.70 to -5.53)	0.05 (-0.08 to 0.18)
Sudan	–	0.20 (0.12 − 0.27)	5.07 (4.50 − 5.64)	-0.48 (-0.55 to -0.40)	1.25 (1.14 − 1.37)	0.67 (0.61 − 0.74)
Suriname	–	2.49 (2.07 − 2.91)	1.94 (1.45 − 2.43)	0.22 (0.04 − 0.40)	0.55 (0.42 − 0.68)	0.87 (0.65 − 1.10)
Sweden	–	-1.53 (-2.03 to -1.03)	0.45 (-0.29 to 1.20)	-0.15 (-0.36 to 0.05)	-1.49 (-1.65 to -1.34)	-1.52 (-1.79 to -1.24)
Switzerland	–	3.03 (2.17 − 3.89)	-0.87 (-1.11 to -0.62)	0.25 (-0.04 to 0.53)	0.40 (0.01 − 0.79)	-2.51 (-2.72 to -2.29)
Syrian Arab Republic	–	-1.41 (-1.76 to -1.06)	-0.70 (-1.02 to -0.38)	0.00 (-0.12 to 0.13)	0.53 (0.43 − 0.63)	0.34 (0.25 − 0.44)
Taiwan (Province of China)	–	4.58 (3.59 − 5.59)	1.68 (0.56 − 2.80)	-1.06 (-1.31 to -0.80)	2.52 (1.79 − 3.26)	1.64 (1.28 − 2.00)
Tajikistan	–	-0.48 (-0.76 to -0.20)	-0.71 (-1.03 to -0.39)	-0.96 (-1.24 to -0.67)	-0.92 (-1.08 to -0.76)	-1.85 (-2.06 to -1.63)

Location	BPH	UTI	Urolithiasis	Bladder cancer	Kidney cancer	Prostate cancer
Thailand	–	0.52 (0.13 − 0.90)	-1.77 (-1.98 to -1.57)	-1.38 (-1.53 to -1.22)	0.89 (0.79 − 0.99)	-0.27 (-0.40 to -0.14)
The former Yugoslav Republic of Macedonia	–	-0.85 (-1.03 to -0.67)	-2.69 (-2.95 to -2.44)	0.14 (-0.19 to 0.47)	1.95 (1.53 − 2.37)	0.83 (0.43 − 1.23)
Timor-Leste	–	0.29 (0.14 − 0.44)	0.21 (0.06 − 0.36)	-0.02 (-0.19 to 0.16)	0.43 (0.27 − 0.59)	1.06 (1.01 − 1.11)
Togo	–	-0.15 (-0.21 to -0.10)	-0.16 (-0.30 to -0.03)	-0.79 (-0.94 to -0.64)	1.80 (1.74 − 1.86)	2.13 (1.99 − 2.27)
Tokelau	–	-0.35 (-0.41 to -0.29)	-2.3 (-2.57 to -2.03)	0.09 (0.02 − 0.16)	0.93 (0.75 − 1.11)	0.31 (0.26 − 0.37)
Tonga	–	0.09 (-0.14 to 0.32)	-1.15 (-1.33 to -0.96)	0.57 (0.42 − 0.73)	0.74 (0.58 − 0.89)	0.27 (0.16 − 0.37)
Trinidad and Tobago	–	3.04 (2.39 − 3.70)	2.03 (1.47 − 2.60)	-0.36 (-0.54 to -0.19)	0.09 (-0.04 to 0.21)	-0.61 (-0.86 to -0.37)
Tunisia	–	0.25 (0.05 − 0.44)	5.02 (4.29 − 5.76)	-0.44 (-0.55 to -0.33)	0.6 (0.57 − 0.62)	0.13 (0.02 − 0.24)
Turkey	–	0.15 (-0.30 to 0.60)	-0.31 (-0.78 to 0.16)	-0.82 (-1.07 to -0.56)	-0.24 (-0.40 to -0.08)	-1.01 (-1.30 to -0.72)
Turkmenistan	–	5.16 (4.85 − 5.47)	1.48 (1.18 − 1.79)	-1.58 (-1.94 to -1.22)	3.93 (3.38 − 4.48)	0.59 (0.30 − 0.88)
Tuvalu	–	-0.32 (-0.37 to -0.28)	-2.08 (-2.20 to -1.96)	0.61 (0.53 − 0.69)	1.03 (0.96 − 1.09)	0.70 (0.63 − 0.78)
Uganda	–	-0.05 (-0.24 to 0.15)	-0.40 (-0.60 to -0.20)	-0.43 (-0.59 to -0.27)	1.43 (1.27 − 1.59)	0.39 (0.25 − 0.54)
Ukraine	–	-0.46 (-0.74 to -0.19)	-2.28 (-2.61 to -1.94)	-0.09 (-0.35 to 0.17)	0.39 (0.16 − 0.61)	-0.19 (-0.42 to 0.03)
United Arab Emirates	–	2.39 (1.55 − 3.24)	2.74 (2.02 − 3.47)	0.94 (0.39 − 1.48)	2.81 (2.29 to 3.34)	1.39 (0.84 − 1.95)
United Kingdom of Great Britain and Northern Ireland	–	3.76 (2.50 − 5.03)	0.83 (0.61 − 1.06)	-1.18 (-1.33 to -1.04)	0.43 (0.33 to 0.53)	-1.16 (-1.26 to -1.05)

Location	BPH	UTI	Urolithiasis	Bladder cancer	Kidney cancer	Prostate cancer
United Republic of Tanzania	–	-0.54 (-0.61 to -0.47)	-1.00 (-1.08 to -0.93)	-0.79 (-0.86 to -0.72)	0.71 (0.61 − 0.80)	-0.38 (-0.46 to -0.29)
United States of America	–	0.07 (-0.22 to 0.36)	2.20 (1.84 − 2.55)	-0.03 (-0.11 to 0.06)	-0.60 (-0.73 to -0.47)	-2.32 (-2.47 to -2.18)
United States Virgin Islands	–	-0.11 (-0.46 to 0.25)	-0.34 (-0.89 to 0.21)	-1.08 (-1.30 to -0.85)	-0.50 (-0.73 to -0.26)	-1.19 (-1.52 to -0.87)
Uruguay	–	7.24 (6.21 − 8.27)	3.06 (2.09 − 4.03)	-0.65 (-0.77 to -0.54)	0.97 (0.85 − 1.08)	-0.33 (-0.59 to -0.07)
Uzbekistan	–	1.43 (0.54 − 2.32)	1.30 (0.76 − 1.84)	0.39 (0.20 − 0.58)	1.31 (1.19 − 1.43)	0.28 (-0.49 to 1.06)
Vanuatu	–	-0.07 (-0.12 to -0.01)	-1.33 (-1.42 to -1.23)	0.37 (0.33 to 0.41)	0.39 (0.34 − 0.44)	0.79 (0.73 − 0.85)
Yemen	–	0.37 (0.30 to 0.44)	5.44 (4.83 − 6.06)	0.00 (-0.05 to 0.04)	0.51 (0.44 − 0.58)	0.90 (0.85 − 0.95)
Zambia	–	0.14 (-0.03 to 0.31)	-0.57 (-0.81 to -0.32)	0.38 (0.26 − 0.49)	2.40 (2.03 − 2.76)	2.33 (2.03 − 2.63)
Zimbabwe	–	1.45 (1.10 − 1.80)	2.27 (1.52 − 3.03)	0.79 (0.50 − 1.08)	2.64 (1.90 − 3.39)	1.05 (0.77 − 1.34)

BPH benign prostatic hyperplasia, *UTI* urinary tract infections, *EAPC* estimated annual percentage change, *ASMR* age-standardized mortality rate

Table S10 EAPC of ASDR for the 6 urologic diseases in 204 countries and territories from 1990 to 2021 (95% CI)

**Location**	**BPH**	**UTI**	**Urolithiasis**	**Bladder cancer**	**Kidney cancer**	**Prostate cancer**
Afghanistan	0.10 (0.09 − 0.11)	-0.07 (-0.22 to 0.09)	2.47 (2.16 − 2.77)	-0.58 (-0.64 to -0.53)	0.63 (0.41 − 0.85)	0.55 (0.53 − 0.58)
Albania	0.13 (0.11 − 0.15)	-1.80 (-2.22 to -1.38)	-1.45 (-1.70 to -1.20)	-0.16 (-0.31 to -0.01)	1.37 (1.12 − 1.62)	-0.38 (-0.51 to -0.25)
Algeria	0.12 (0.11 − 0.14)	0.08 (0.03 − 0.14)	1.46 (1.26 − 1.66)	-0.44 (-0.59 to -0.28)	1.19 (1.09 − 1.29)	0.01 (-0.04 to 0.07)
American Samoa	0.22 (0.20 − 0.24)	-0.29 (-0.53 to -0.06)	-0.74 (-0.98 to -0.51)	2.17 (1.77 − 2.56)	1.18 (0.89 − 1.48)	0.53 (0.34 − 0.73)
Andorra	0.28 (0.25 − 0.31)	-1.13 (-1.31 to -0.95)	-0.40 (-0.51 to -0.29)	-1.38 (-1.63 to -1.13)	-0.78 (-0.94 to -0.61)	-0.48 (-0.69 to -0.27)
Angola	0.05 (0.04 − 0.06)	-0.82 (-0.91 to -0.73)	-0.59 (-0.65 to -0.53)	-0.28 (-0.38 to -0.18)	0.59 (0.49 − 0.69)	0.70 (0.62 − 0.77)
Antigua and Barbuda	0.19 (0.18 − 0.21)	3.83 (3.30 − 4.37)	1.58 (1.28 − 1.88)	0.23 (-0.02 to 0.47)	0.28 (0.10 − 0.47)	-0.07 (-0.44 to 0.31)
Argentina	0.24 (0.20 − 0.29)	6.69 (5.82 − 7.58)	0.10 (-0.03 to 0.24)	-1.42 (-1.53 to -1.31)	0.64 (0.50 − 0.77)	-0.63 (-0.99 to -0.28)
Armenia	0.09 (0.06 − 0.11)	2.46 (1.06 − 3.88)	0.58 (-0.03 to 1.20)	-0.03 (-0.28 to 0.22)	1.21 (0.90 − 1.52)	1.51 (1.28 − 1.73)
Australia	0.12 (0.10 − 0.14)	0.88 (0.39 − 1.36)	-0.70 (-1.02 to -0.38)	-1.51 (-1.59 to -1.44)	-0.73 (-0.83 to -0.63)	-2.8 (-3.24 to -2.36)
Austria	0.58 (0.46 − 0.70)	0.94 (0.50 − 1.38)	-1.52 (-1.73 to -1.30)	-1.22 (-1.32 to -1.11)	-1.44 (-1.54 to -1.33)	-1.74 (-1.88 to -1.61)
Azerbaijan	0.07 (0.05 − 0.09)	0.13 (-0.23 to 0.49)	0.30 (0.14 − 0.47)	-0.70 (-0.84 to -0.56)	0.51 (0.38 − 0.64)	-0.15 (-0.32 to 0.02)
Bahrain	0.20 (0.18 − 0.21)	-1.14 (-1.93 to -0.34)	2.00 (1.72 to 2.28)	-2.15 (-2.39 to -1.90)	-0.84 (-1.07 to -0.62)	-0.6 (-0.83 to -0.37)
Bangladesh	0.14 (0.13 − 0.14)	-1.47 (-1.60 to -1.34)	-1.33 (-1.43 to -1.23)	-1.30 (-1.46 to -1.14)	-0.29 (-0.35 to -0.24)	-0.66 (-0.80 to -0.52)
Barbados	0.16 (0.14 − 0.18)	3.83 (2.96 − 4.70)	1.94 (1.54 − 2.35)	0.36 (0.21 − 0.51)	0.50 (0.37 − 0.63)	-0.05 (-0.18 to 0.08)

Location	BPH	UTI	Urolithiasis	Bladder cancer	Kidney cancer	Prostate cancer
Belarus	0.00 (-0.02 to 0.02)	-0.56 (-1.15 to 0.04)	-0.04 (-0.17 to 0.10)	-1.74 (-2.14 to -1.33)	2.28 (1.43 − 3.13)	1.39 (1.10 − 1.67)
Belgium	0.30 (0.26 − 0.35)	3.7 (3.19 − 4.22)	0.46 (-0.31 to 1.23)	-1.50 (-1.72 to -1.28)	-0.83 (-1.06 to -0.60)	-2.5 (-2.73 to -2.28)
Belize	0.19 (0.17 − 0.21)	4.18 (3.17 − 5.20)	2.29 (1.72 − 2.86)	0.85 (0.45 − 1.26)	0.74 (0.40 − 1.08)	0.93 (0.13 −1.73)
Benin	0.07 (0.06 − 0.08)	-1.04 (-1.15 to -0.93)	-0.75 (-0.84 to -0.66)	-1.11 (-1.25 to -0.97)	1.73 (1.60 − 1.86)	1.68 (1.57 − 1.78)
Bermuda	0.15 (0.13 − 0.16)	2.13 (1.73 − 2.53)	0.83 (0.55 − 1.11)	-0.94 (-1.21 to -0.67)	-1.09 (-1.31 to -0.87)	-0.68 (-0.84 to -0.53)
Bhutan	0.15 (0.14 − 0.16)	-0.30 (-0.37 to -0.24)	-0.72 (-0.83 to -0.61)	-0.53 (-0.63 to -0.43)	0.62 (0.54 − 0.71)	0.00 (-0.06 to 0.07)
Bolivarian Republic of Venezuela	0.16 (0.15 − 0.18)	2.95 (2.47 − 3.43)	0.75 (0.31 − 1.18)	-0.30 (-0.43 to -0.18)	0.35 (0.05 − 0.64)	0.46 (0.10 − 0.83)
Bosnia and Herzegovina	0.22 (0.19 − 0.25)	-2.46 (-3.06 to -1.85)	-2.07 (-2.32 to -1.81)	0.97 (0.80 − 1.14)	2.53 (2.22 − 2.83)	1.19 (1.04 − 1.35)
Botswana	0.08 (0.07 − 0.10)	-0.01 (-0.28 to 0.26)	-0.26 (-0.49 to -0.04)	-0.88 (-1.08 to -0.68)	1.46 (1.33 − 1.60)	0.26 (-0.02 to 0.55)
Brazil	-0.32 (-0.44 to -0.20)	3.31 (2.98 − 3.65)	2.91 (2.66 − 3.17)	-0.35 (-0.41 to -0.29)	0.84 (0.73 − 0.95)	-0.15 (-0.48 to 0.18)
Brunei Darussalam	0.12 (0.10 − 0.14)	1.24 (0.79 − 1.70)	-0.02 (-0.23 to 0.20)	-0.51 (-0.73 to -0.29)	0.44 (0.27 − 0.61)	0.52 (0.31 − 0.74)
Bulgaria	0.14 (0.11 − 0.17)	-2.80 (-4.13 to -1.45)	-4.15 (-4.84 to -3.46)	1.12 (1.01 − 1.23)	3.99 (3.53 − 4.44)	1.24 (1.07 − 1.41)
Burkina Faso	0.08 (0.07 − 0.09)	-0.84 (-0.99 to -0.68)	-0.51 (-0.60 to -0.41)	-0.95 (-1.09 to -0.80)	1.93 (1.80 − 2.05)	1.87 (1.77 − 1.96)
Burundi	-0.01 (-0.03 to 0.01)	-1.63 (-1.78 to -1.49)	-1.14 (-1.29 to -0.98)	-1.51 (-1.67 to -1.36)	-0.59 (-0.69 to -0.50)	-0.57 (-0.67 to -0.47)
Cambodia	0.11 (0.10 − 0.12)	-0.46 (-0.53 to -0.38)	-0.29 (-0.35 to -0.24)	-0.39 (-0.58 to -0.20)	0.63 (0.55 to 0.72)	1.11 (1.03 − 1.18)

Location	BPH	UTI	Urolithiasis	Bladder cancer	Kidney cancer	Prostate cancer
Cameroon	0.08 (0.07 − 0.09)	-1.31 (-1.41 to -1.22)	-0.77 (-0.91 to -0.64)	-0.61 (-0.76 to -0.46)	1.79 (1.65 to 1.92)	1.72 (1.62 − 1.83)
Canada	0.22 (0.18 − 0.26)	1.04 (0.72 − 1.35)	0.86 (0.71 − 1.02)	-1.73 (-1.88 to -1.58)	0.06 (-0.17 to 0.29)	-3.27 (-3.54 to -3.01)
Central African Republic	0.06 (0.05 − 0.07)	-0.45 (-0.50 to -0.39)	-0.09 (-0.15 to -0.04)	-0.53 (-0.58 to -0.49)	0.06 (0.02 to 0.09)	0.32 (0.29 − 0.34)
Chad	0.05 (0.04 − 0.06)	-0.63 (-0.70 to -0.55)	0.08 (-0.09 to 0.26)	0.07 (-0.12 to 0.26)	2.42 (2.28 to 2.56)	2.25 (2.08 − 2.43)
Chile	0.27 (0.24 − 0.31)	1.06 (0.84 − 1.28)	0.70 (0.39 − 1.01)	-0.77 (-0.89 to -0.65)	-0.08 (-0.23 to 0.07)	-0.24 (-0.54 to 0.06)
China	-0.26 (-0.43 to -0.09)	-3.21 (-3.60 to -2.82)	-3.78 (-4.00 to -3.55)	-1.74 (-1.88 to -1.60)	-0.01 (-0.15 to 0.14)	-0.3 (-0.48 to -0.11)
Colombia	0.16 (0.15 − 0.17)	2.30 (1.82 − 2.78)	-0.51 (-0.63 to -0.40)	-1.54 (-1.72 to -1.35)	0.23 (0.13 − 0.34)	-1.25 (-1.52 to -0.98)
Commonwealth of the Bahamas	0.16 (0.14 − 0.17)	3.98 (3.35 − 4.61)	1.34 (1.04 − 1.65)	0.47 (0.33 − 0.60)	0.41 (0.23 − 0.60)	0.23 (0.02 − 0.44)
Comoros	0.00 (-0.01 to 0.01)	-0.68 (-0.88 to -0.47)	-0.49 (-0.64 to -0.33)	-0.58 (-0.71 to -0.46)	0.28 (0.17 − 0.40)	0.15 (0.09 − 0.21)
Congo	0.03 (0.02 − 0.05)	-1.05 (-1.21 to -0.90)	-0.64 (-0.77 to -0.51)	-0.71 (-0.87 to -0.55)	0.57 (0.45 − 0.69)	0.11 (0.01 − 0.21)
Cook Islands	0.16 (0.15 − 0.18)	-1.29 (-1.51 to -1.08)	-0.22 (-0.25 to -0.20)	-0.09 (-0.22 to 0.03)	-0.96 (-1.19 to -0.73)	-0.30 (-0.37 to -0.24)
Costa Rica	0.16 (0.15 − 0.17)	2.97 (2.61 − 3.33)	-0.21 (-0.27 to -0.15)	-0.79 (-0.93 to -0.64)	1.88 (1.74 − 2.03)	0.88 (0.50 − 1.27)
Croatia	0.02 (-0.06 to 0.10)	0.86 (0.04 − 1.69)	-1.86 (-2.02 to -1.69)	0.26 (0.12 − 0.40)	0.57 (0.27 − 0.87)	-0.30 (-0.52 to -0.09)
Cuba	0.15 (0.13 − 0.17)	3.77 (3.38 − 4.16)	1.94 (1.63 − 2.24)	0.53 (0.37 − 0.69)	0.29 (0.16 − 0.43)	0.94 (0.86 − 1.03)
Cyprus	0.12 (0.11 − 0.14)	-2.19 (-2.47 to -1.90)	-2.10 (-2.53 to -1.67)	-1.38 (-1.46 to -1.30)	0.48 (0.27 − 0.69)	-1.37 (-1.63 to -1.11)

Location	BPH	UTI	Urolithiasis	Bladder cancer	Kidney cancer	Prostate cancer
Czech Republic	0.23 (0.21 − 0.26)	-1.60 (-2.61 to -0.57)	-3.76 (-4.35 to -3.16)	-0.58 (-0.67 to -0.48)	-0.4 (-0.77 to -0.03)	-0.66 (-0.97 to -0.34)
Democratic People's Republic of Korea	0.10 (0.09 to 0.11)	-0.75 (-0.85 to -0.65)	-0.57 (-0.63 to -0.52)	-0.29 (-0.35 to -0.24)	0.16 (0.08 − 0.24)	0.49 (0.39 − 0.59)
Democratic Republic of the Congo	0.06 (0.05 − 0.07)	-0.73 (-0.82 to -0.64)	0.06 (0.01 − 0.11)	-0.23 (-0.42 to -0.04)	0.08 (-0.18 to 0.34)	0.58 (0.42 − 0.74)
Denmark	0.34 (0.29 − 0.38)	1.89 (1.28 − 2.50)	-0.31 (-0.44 to -0.19)	-1.67 (-2.21 to -1.13)	-0.33 (-0.83 to 0.17)	-0.88 (-1.12 to -0.64)
Djibouti	0.01 (-0.01 to 0.02)	0.32 (0.09 − 0.54)	0.28 (0.11 − 0.45)	-0.06 (-0.14 to 0.03)	0.8 (0.67 − 0.93)	0.24 (0.16 − 0.31)
Dominica	0.23 (0.21 − 0.25)	2.47 (2.16 − 2.77)	0.87 (0.76 − 0.98)	0.18 (0.09 − 0.26)	1.11 (1.03 − 1.18)	0.06 (-0.25 to 0.38)
Dominican Republic	0.17 (0.14 − 0.19)	0.71 (0.53 − 0.90)	0.48 (0.34 − 0.62)	0.61 (0.48 −0.74)	0.86 (0.71 − 1.01)	0.39 (0.03 − 0.75)
Ecuador	-0.01 (-0.12 to 0.09)	3.08 (2.49 − 3.67)	1.35 (1.13 − 1.57)	0.38 (0.11 − 0.64)	0.50 (0.19 − 0.81)	0.09 (-0.32 to 0.49)
Egypt	0.11 (0.10 − 0.12)	3.33 (2.90 − 3.76)	1.97 (1.68 − 2.27)	-3.06 (-3.4 to -2.73)	1.50 (1.28 − 1.72)	2.47 (2.19 − 2.76)
El Salvador	0.22 (0.22 − 0.23)	0.32 (0.13 − 0.51)	-0.16 (-0.23 to -0.09)	0.25 (0.14 − 0.36)	0.85 (0.77 − 0.94)	0.66 (0.41 − 0.91)
Equatorial Guinea	0.09 (0.08 − 0.10)	0.07 (-0.22 to 0.36)	0.02 (-0.23 to 0.28)	-0.32 (-0.56 to -0.09)	2.02 (1.80 − 2.25)	0.75 (0.66 − 0.83)
Eritrea	-0.01 (-0.02 to 0.01)	-0.22 (-0.30 to -0.15)	-0.48 (-0.56 to -0.40)	-0.29 (-0.36 to -0.23)	0.50 (0.42 − 0.58)	0.22 (0.10 − 0.35)
Estonia	0.06 (0.03 − 0.08)	-2.26 (-3.14 to -1.37)	-0.88 (-1.15 to -0.60)	-1.10 (-1.35 to -0.84)	0.60 (0.10 to 1.11)	1.66 (0.98 − 2.34)
Ethiopia	0.00 (-0.02 to 0.02)	-2.48 (-2.74 to -2.23)	-2.7 (-2.86 to -2.54)	-1.39 (-1.54 to -1.24)	-0.73 (-0.97 to -0.49)	-0.17 (-0.30 to -0.05)
Federated States of Micronesia	0.21 (0.18 − 0.23)	-0.37 (-0.46 to -0.27)	-0.37 (-0.42 to -0.31)	0.46 (0.42 − 0.49)	0.45 (0.39 to 0.50)	0.64 (0.60 to 0.69)

Location	BPH	UTI	Urolithiasis	Bladder cancer	Kidney cancer	Prostate cancer
Fiji	0.20 (0.19 − 0.21)	1.47 (1.28 − 1.66)	0.38 (0.28 − 0.48)	1.04 (0.84 − 1.25)	-0.25 (-0.47 to -0.03)	0.36 (0.24 − 0.47)
Finland	0.23 (0.21 − 0.24)	-1.88 (-2.34 to -1.42)	0.00 (-0.46 to 0.47)	-2.07 (-2.18 to -1.96)	-1.06 (-1.16 to -0.96)	-1.60 (-1.82 to -1.39)
France	0.27 (0.23 − 0.30)	0.27 (0.01 − 0.52)	-0.41 (-0.46 to -0.36)	-1.05 (-1.11 to -1.00)	-0.19 (-0.28 to -0.10)	-2.24 (-2.42 to -2.06)
Gabon	0.05 (0.03 − 0.06)	0.14 (-0.07 to 0.35)	0.34 (0.18 − 0.50)	-0.46 (-0.51 to -0.41)	1.30 (1.20 − 1.39)	0.38 (0.36 to 0.40)
Georgia	0.10 (0.03 − 0.17)	2.71 (1.96 − 3.47)	0.78 (0.46 − 1.10)	0.73 (0.18 − 1.30)	2.46 (2.09 − 2.84)	3.89 (3.13 − 4.64)
Germany	0.33 (0.30 − 0.37)	3.17 (2.83 − 3.50)	0.5 (0.22 − 0.78)	-1.57 (-1.76 to -1.37)	-1.23 (-1.35 to -1.11)	-1.49 (-1.71 to -1.28)
Ghana	0.08 (0.07 − 0.08)	1.41 (1.27 − 1.56)	0.35 (0.26 − 0.43)	-0.41 (-0.53 to -0.29)	2.84 (2.69 − 2.99)	0.13 (-0.02 to 0.28)
Greece	0.20 (0.10 − 0.29)	1.82 (0.91 − 2.75)	0.94 (0.62 − 1.25)	-0.92 (-1.10 to -0.75)	0.00 (-0.11 to 0.11)	-1.34 (-1.60 to -1.07)
Greenland	0.29 (0.25 − 0.33)	-1.1 (-1.17 to -1.02)	-0.10 (-0.15 to -0.05)	-1.27 (-1.42 to -1.13)	-0.26 (-0.37 to -0.14)	-1.07 (-1.14 to -0.99)
Grenada	0.20 (0.18 − 0.22)	2.87 (2.41 − 3.34)	1.80 (1.62 − 1.98)	0.45 (0.17 − 0.74)	1.20 (1.01 − 1.39)	1.51 (0.14 − 2.90)
Guam	0.09 (0.08 − 0.11)	-1.45 (-1.98 to -0.92)	-1.06 (-1.22 to -0.89)	1.37 (1.01 − 1.73)	0.82 (0.45 − 1.19)	-0.10 (-0.35 to 0.15)
Guatemala	0.23 (0.22 − 0.24)	2.87 (2.36 − 3.39)	-0.81 (-1.04 to -0.57)	-1.16 (-1.40 to -0.92)	0.10 (-0.25 to 0.47)	0.79 (0.09 − 1.49)
Guinea	0.06 (0.04 − 0.07)	-0.97 (-1.04 to -0.90)	-0.18 (-0.24 to -0.12)	0.55 (0.48 − 0.63)	0.26 (0.16 − 0.35)	1.00 (0.93 − 1.08)
Guinea-Bissau	0.07 (0.06 − 0.08)	-1.53 (-1.57 to -1.48)	-1.10 (-1.15 to -1.06)	-0.80 (-0.88 to -0.72)	1.11 (0.91 − 1.30)	1.81 (1.69 − 1.93)
Guyana	0.23 (0.21 − 0.25)	3.91 (3.22 − 4.61)	2.68 (2.07 − 3.29)	0.60 (0.41 to 0.80)	1.01 (0.81 − 1.21)	0.61 (0.35 − 0.87)

Location	BPH	UTI	Urolithiasis	Bladder cancer	Kidney cancer	Prostate cancer
Haiti	0.22 (0.21 − 0.23)	0.70 (0.59 − 0.81)	0.43 (0.32 − 0.55)	-0.08 (-0.15 to 0.00)	-0.21 (-0.29 to -0.12)	0.38 (0.34 − 0.41)
Honduras	0.17 (0.16 − 0.18)	1.00 (0.87 − 1.13)	0.42 (0.28 − 0.55)	1.20 (1.08 − 1.32)	1.06 (0.96 − 1.17)	1.14 (1.05 − 1.23)
Hungary	0.18 (0.16 − 0.21)	-0.93 (-1.82 to -0.03)	-3.84 (-4.48 to -3.19)	-0.05 (-0.30 to 0.20)	-0.2 (-0.48 to 0.07)	-0.90 (-1.13 to -0.68)
Iceland	0.34 (0.29 − 0.38)	2.74 (2.21 − 3.28)	0.23 (0.06 − 0.40)	-1.50 (-1.64 to -1.35)	-0.27 (-0.48 to -0.07)	-1.32 (-1.51 to -1.14)
India	0.24 (0.21 − 0.28)	0.22 (0.14 − 0.30)	-0.73 (-0.91 to -0.55)	-0.37 (-0.53 to -0.21)	0.79 (0.69 − 0.90)	0.22 (0.06 − 0.38)
Indonesia	-0.43 (-0.70 to -0.17)	0.79 (0.68 − 0.90)	-0.31 (-0.39 to -0.23)	-0.03 (-0.05 to -0.01)	1.21 (1.16 − 1.27)	1.41 (1.34 − 1.49)
Iraq	0.06 (0.04 − 0.08)	-0.79 (-0.92 to -0.66)	-0.30 (-0.36 to -0.24)	0.22 (0.10 − 0.35)	0.54 (0.42 − 0.66)	1.03 (0.85 − 1.22)
Ireland	0.33 (0.29 − 0.37)	0.45 (0.21 − 0.69)	-0.67 (-0.78 to -0.56)	-1.32 (-1.48 to -1.15)	-0.33 (-0.56 to -0.10)	-2.09 (-2.43 to -1.75)
Islamic Republic of Iran	0.11 (0.10 − 0.13)	-1.39 (-1.48 to -1.29)	-1.44 (-1.54 to -1.34)	-0.1 (-0.18 to -0.02)	0.60 (0.48 − 0.73)	0.94 (0.78 − 1.11)
Israel	0.29 (0.26 − 0.32)	2.03 (1.15 − 2.92)	0.09 (-0.17 to 0.35)	-0.99 (-1.26 to -0.72)	-0.76 (-1.09 to -0.43)	-2.25 (-2.66 to -1.83)
Italy	0.06 (0.02 − 0.10)	1.56 (0.88 − 2.25)	-1.37 (-1.60 to -1.14)	-1.55 (-1.61 to -1.49)	-0.67 (-0.77 to -0.57)	-1.41 (-1.62 to -1.19)
Jamaica	0.23 (0.21 − 0.24)	4.00 (3.09 − 4.91)	2.80 (2.18 − 3.42)	0.53 (0.21 − 0.84)	0.54 (0.21 − 0.87)	1.60 (0.90 − 2.31)
Japan	-0.08 (-0.17 to 0.00)	2.06 (1.74 − 2.39)	0.93 (0.83 − 1.02)	-0.33 (-0.39 to -0.26)	-0.06 (-0.26 to 0.13)	-0.03 (-0.31 to 0.25)
Jordan	0.21 (0.11 − 0.31)	-0.16 (-0.29 to -0.02)	1.31 (1.12 − 1.50)	-1.06 (-1.20 to -0.91)	0.68 (0.56 − 0.81)	0.19 (0.08 − 0.30)
Kazakhstan	0.07 (0.05 − 0.09)	0.08 (-0.32 to 0.47)	1.23 (0.85 − 1.61)	-1.59 (-2.11 to -1.07)	-0.77 (-1.33 to -0.21)	0.36 (0.09 − 0.64)

Location	BPH	UTI	Urolithiasis	Bladder cancer	Kidney cancer	Prostate cancer
Kenya	0.00 (0.00 − 0.01)	1.38 (1.24 − 1.53)	1.02 (0.82 − 1.21)	1.10 (0.94 − 1.25)	1.81 (1.60 − 2.02)	1.91 (1.68 to 2.14)
Kingdom of Eswatini	0.04 (0.03 − 0.06)	0.81 (0.18v1.45)	0.94 (0.31 − 1.56)	0.49 (0.09 − 0.90)	1.45 (1.10 − 1.79)	0.97 (0.75 − 1.20)
Kiribati	0.18 (0.18 − 0.19)	-0.47 (-0.61 to -0.32)	-0.46 (-0.54 to -0.39)	0.41 (0.33 −0.48)	0.84 (0.59 − 1.08)	0.44 (0.40 − 0.48)
Kuwait	0.08 (0.05 − 0.10)	5.39 (3.96 − 6.85)	1.01 (0.84 − 1.18)	-0.65 (-1.16 to -0.13)	0.12 (-0.46 to 0.71)	1.71 (1.08 − 2.35)
Kyrgyzstan	0.01 (-0.01 to 0.04)	1.01 (-0.09 to 2.12)	-0.07 (-0.41 to 0.26)	-1.55 (-2.01 to -1.10)	2.01 (1.33 − 2.68)	-0.51 (-0.85 to -0.17)
Lao People's Democratic Republic	0.11 (0.09 − 0.12)	-1.27 (-1.39 to -1.15)	-1.27 (-1.36 to -1.19)	-0.95 (-1.05 to -0.84)	0.28 (0.26 − 0.29)	0.49 (0.45 − 0.52)
Latvia	0.08 (0.06 − 0.10)	-1.59 (-2.31 to -0.87)	-0.72 (-0.98 to -0.45)	0.25 (0.02 − 0.47)	2.75 (2.14 − 3.36)	2.26 (1.96 − 2.57)
Lebanon	0.09 (0.08 − 0.10)	-0.88 (-0.97 to -0.78)	-0.57 (-0.68 to -0.46)	-0.56 (-0.76 to -0.36)	1.37 (1.10 − 1.63)	1.28 (1.00 − 1.56)
Lesotho	0.03 (0.02 − 0.04)	2.96 (2.43 − 3.50)	2.46 (2.11 − 2.81)	1.65 (1.35 − 1.94)	3.05 (2.78 − 3.33)	1.28 (1.13 − 1.43)
Liberia	0.08 (0.06 − 0.09)	-1.61 (-2.16 to -1.05)	-0.57 (-0.85 to -0.29)	-0.81 (-0.95 to -0.68)	1.50 (0.96 − 2.04)	1.70 (1.60 − 1.80)
Libya	0.14 (0.13 − 0.15)	1.62 (1.37 − 1.87)	2.76 (2.49 − 3.04)	0.59 (0.42 − 0.76)	0.91 (0.78 − 1.04)	0.55 (0.46 − 0.63)
Lithuania	0.05 (0.04 − 0.06)	-0.44 (-1.23 to 0.36)	-0.90 (-1.2 to -0.60)	-1.05 (-1.71 to -0.39)	2.00 (1.43 − 2.57)	2.36 (1.83 − 2.89)
Luxembourg	0.49 (0.44 − 0.54)	2.09 (1.82 − 2.37)	-0.54 (-1.09 to 0.01)	-1.42 (-1.54 to -1.30)	-0.83 (-1.02 to -0.64)	-2.19 (-2.31 to -2.08)
Madagascar	0.01 (0.00 − 0.02)	-0.77 (-0.87 to -0.67)	-0.33 (-0.38 to -0.28)	-0.70 (-0.81 to -0.60)	-0.13 (-0.30 to 0.04)	-0.54 (-0.67 to -0.41)
Malawi	0.05 (0.04 − 0.05)	-0.44 (-0.63 to -0.26)	-0.28 (-0.45 to -0.10)	0.29 (0.12 − 0.47)	0.01 (-0.06 to 0.08)	1.13 (0.98 − 1.27)

Location	BPH	UTI	Urolithiasis	Bladder cancer	Kidney cancer	Prostate cancer
Malaysia	0.07 (0.06 − 0.08)	0.48 (0.34 − 0.62)	0.22 (0.15 − 0.29)	-0.16 (-0.30 to -0.01)	0.22 (0.04 − 0.40)	0.25 (0.10 − 0.40)
Maldives	0.06 (0.05 − 0.07)	-2.45 (-2.62 to -2.28)	0.00 (-0.03 to 0.03)	-2.22 (-2.33 to -2.10)	-0.94 (-1.07 to -0.82)	-0.83 (-0.95 to -0.70)
Mali	0.05 (0.04 − 0.06)	-1.38 (-1.55 to -1.22)	-1.01 (-1.11 to -0.90)	-0.03 (-0.11 to 0.04)	-0.07 (-0.17 to 0.03)	0.67 (0.57 − 0.77)
Malta	0.18 (0.16 − 0.20)	1.88 (1.57 − 2.20)	-0.10 (-0.49 to 0.30)	-1.67 (-1.82 to -1.52)	-0.03 (-0.18 to 0.13)	-1.90 (-2.09 to -1.72)
Marshall Islands	0.24 (0.23 − 0.26)	0.25 (0.12 − 0.37)	-0.15 (-0.24 to -0.06)	0.91 (0.77 − 1.04)	0.99 (0.90 − 1.07)	0.84 (0.76 − 0.92)
Mauritania	0.03 (0.02 − 0.05)	-1.48 (-1.59 to -1.37)	-1.31 (-1.45 to -1.16)	-1.14 (-1.27 to -1.00)	1.60 (1.51 − 1.70)	1.71 (1.62 to 1.80)
Mauritius	0.17 (0.15 − 0.18)	5.81 (5.40 − 6.22)	0.26 (-0.08 to 0.61)	-1.52 (-2.30 to -0.74)	-1.82 (-3.46 to -0.16)	0.31 (0.02 − 0.59)
Mexico	0.09 (0.04 − 0.14)	2.95 (2.15 − 3.77)	-0.23 (-0.45 to -0.02)	-0.42 (-0.58 to -0.26)	0.91 (0.82 − 1.00)	-0.31 (-0.46 to -0.16)
Mongolia	0.02 (0.00 − 0.04)	-1.71 (-2.00 to -1.42)	-1.14 (-1.32 to -0.96)	-2.94 (-3.40 to -2.49)	4.82 (4.35 − 5.30)	1.44 (1.36 − 1.52)
Montenegro	0.14 (0.12 − 0.16)	-0.02 (-0.11 to 0.07)	-0.27 (-0.32 to -0.22)	0.52 (0.41 − 0.62)	0.9 (0.78 − 1.02)	0.79 (0.48 − 1.10)
Morocco	0.10 (0.09 − 0.11)	0.71 (0.64 − 0.77)	2.02 (1.81 − 2.23)	0.39 (0.24 − 0.55)	1.13 (1.02 − 1.24)	0.91 (0.76 − 1.07)
Mozambique	0.04 (0.03 − 0.05)	0.85 (0.64 − 1.05)	1.12 (0.91 − 1.32)	0.66 (0.55 − 0.78)	1.01 (0.88 − 1.14)	1.05 (0.97 − 1.13)
Myanmar	0.09 (0.09 − 0.10)	-1.07 (-1.25 to -0.88)	-1.00 (-1.13 to -0.86)	-1.27 (-1.36 to -1.17)	0.13 (0.08 − 0.18)	0.57 (0.54 − 0.59)
Namibia	0.04 (0.02 − 0.05)	-0.07 (-0.41 to 0.26)	-0.12 (-0.37 to 0.13)	0.08 (-0.16 to 0.33)	1.05 (0.94 − 1.16)	2.26 (2.04 − 2.48)
Nepal	0.09 (0.02 − 0.15)	0.45 (0.09 − 0.81)	-0.15 (-0.47 to 0.17)	-0.34 (-0.59 to -0.10)	0.77 (0.59 − 0.95)	0.20 (0.04 − 0.37)

Location	BPH	UTI	Urolithiasis	Bladder cancer	Kidney cancer	Prostate cancer
Netherlands	0.23 (0.20 − 0.26)	0.29 (-0.24 to 0.83)	-0.78 (-0.94 to -0.62)	-1.23 (-1.39 to -1.08)	-0.55 (-0.69 to -0.40)	-1.72 (-1.91 to -1.52)
New Zealand	-0.33 (-0.44 to -0.21)	0.20 (-0.67 to 1.07)	-0.48 (-0.88 to -0.08)	-1.41 (-1.71 to -1.10)	-0.25 (-0.37 to -0.13)	-1.96 (-2.16 to -1.76)
Nicaragua	0.18 (0.17 − 0.20)	1.14 (0.93 − 1.34)	0.12 (0.03 − 0.21)	0.26 (0.03 − 0.49)	0.78 (0.62 − 0.94)	0.13 (-0.16 to 0.43)
Niger	0.06 (0.04 − 0.07)	-2.17 (-2.39 to -1.96)	-1.50 (-1.62 to -1.39)	-0.95 (-1.06 to -0.85)	0.52 (0.41 − 0.64)	1.88 (1.76 − 2.01)
Nigeria	0.04 (0.03 − 0.06)	-0.83 (-0.97 to -0.69)	-0.84 (-0.91 to -0.77)	0.13 (0.08 − 0.18)	0.76 (0.65 − 0.88)	0.61 (0.55 − 0.68)
Northern Mariana Islands	0.12 (0.11 − 0.13)	-0.62 (-0.99 to -0.25)	-0.52 (-0.60 to -0.43)	3.91 (3.32 − 4.50)	0.92 (0.66 − 1.18)	1.20 (0.99 − 1.41)
Norway	0.09 (0.08 − 0.10)	0.88 (0.51 − 1.25)	-0.23 (-0.47 to 0.01)	-1.62 (-1.77 to -1.47)	-0.96 (-1.26 to -0.65)	-1.69 (-1.95 to -1.43)
Oman	0.11 (0.09 − 0.13)	0.83 (0.51 − 1.14)	0.95 (0.80 − 1.09)	-0.49 (-0.71 to -0.27)	1.32 (1.06 − 1.57)	0.40 (0.20 − 0.61)
Pakistan	0.16 (0.15 − 0.17)	0.79 (0.58 − 1.01)	-0.11 (-0.31 to 0.10)	0.13 (-0.04 to 0.31)	1.10 (0.96 − 1.25)	0.73 (0.54 − 0.91)
Palestine	0.10 (0.10 − 0.11)	-0.35 (-0.45 to -0.25)	-0.13 (-0.22 to -0.04)	-0.80 (-1.00 to -0.60)	-0.09 (-0.17 to -0.01)	-0.15 (-0.39 to 0.10)
Panama	0.18 (0.17 − 0.19)	2.98 (2.65 − 3.30)	-0.14 (-0.22 to -0.06)	-0.12 (-0.31 to 0.07)	0.86 (0.69 − 1.03)	0.17 (-0.33 to 0.67)
Papua New Guinea	0.14 (0.12 − 0.15)	-0.43 (-0.48 to -0.39)	-0.22 (-0.27 to -0.17)	0.48 (0.43 − 0.52)	0.44 (0.41 − 0.48)	0.83 (0.79 − 0.87)
Paraguay	0.11 (0.09 − 0.12)	1.00 (0.82 − 1.19)	2.45 (2.29 − 2.60)	1.09 (1.01 − 1.17)	1.70 (1.54 − 1.85)	1.51 (1.16 − 1.86)
Peru	0.19 (0.17 − 0.21)	0.56 (0.28 − 0.85)	0.04 (-0.03 to 0.10)	-0.82 (-1.05 to -0.59)	-0.45 (-0.72 to -0.19)	-0.24 (-0.36 to -0.12)
Philippines	0.06 (0.03 − 0.09)	0.62 (0.49 − 0.74)	0.42 (0.34 − 0.49)	0.29 (0.24 − 0.33)	0.80 (0.74 − 0.86)	0.69 (0.63 − 0.74)

Location	BPH	UTI	Urolithiasis	Bladder cancer	Kidney cancer	Prostate cancer
Plurinational State of Bolivia	0.18 (0.16 − 0.20)	0.30 (0.23 − 0.37)	-0.13 (-0.16 to -0.10)	-0.45 (-0.51 to -0.40)	0.40 (0.35 − 0.45)	0.11 (0.07 − 0.16)
Poland	-0.80 (-1.22 to -0.37)	2.37 (0.13 − 4.66)	-4.94 (-5.53 to -4.34)	0.34 (0.18 − 0.49)	-0.75 (-0.92 to -0.59)	0.82 (0.49 − 1.14)
Portugal	0.34 (0.30 − 0.38)	5.14 (4.54 − 5.73)	-0.23 (-0.37 to -0.09)	-0.55 (-0.65 to -0.44)	-0.14 (-0.37 to 0.08)	-1.81 (-2.03 to -1.59)
Principality of Monaco	0.29 (0.26 − 0.33)	0.86 (0.64 − 1.08)	-0.02 (-0.12 to 0.07)	0.11 (0.08 − 0.14)	1.20 (0.95 − 1.45)	-0.14 (-0.19 to -0.10)
Puerto Rico	0.20 (0.17 − 0.22)	2.22 (1.41 − 3.05)	1.72 (1.41 − 2.04)	-0.64 (-0.82 to -0.47)	0.14 (-0.06 to 0.33)	-1.78 (-1.99 to -1.57)
Qatar	0.26 (0.22 − 0.29)	-3.60 (-4.30 to -2.89)	-0.74 (-1.17 to -0.31)	-1.71 (-2.15 to -1.27)	-0.75 (-1.00 to -0.49)	-0.44 (-0.82 to -0.05)
Republic of Cabo Verde	0.07 (0.06 − 0.08)	-0.59 (-0.75 to -0.44)	0.53 (0.30 − 0.75)	5.51 (4.05 − 6.99)	7.94 (6.11 − 9.80)	2.24 (1.79 − 2.69)
Republic of Côte d'Ivoire	0.09 (0.08 − 0.10)	-0.74 (-0.86 to -0.62)	-0.63 (-0.78 to -0.48)	-0.36 (-0.49 to -0.24)	0.52 (0.39 − 0.64)	0.23 (0.13 − 0.32)
Republic of Korea	0.11 (0.10 − 0.12)	0.24 (-0.15 to 0.62)	-0.23 (-0.28 to -0.18)	-1.79 (-1.88 to -1.69)	0.13 (-0.18 to 0.43)	0.72 (0.40 − 1.04)
Republic of Moldova	0.02 (0.01 − 0.04)	-1.02 (-1.53 to -0.50)	0.09 (-0.05 to 0.24)	-0.42 (-0.64 to -0.21)	0.74 (0.43 − 1.05)	2.38 (1.82 − 2.94)
Republic of Nauru	0.14 (0.13 − 0.16)	-0.32 (-0.47 to -0.18)	-0.67 (-0.74 to -0.60)	0.17 (0.04 to 0.30)	-0.23 (-0.36 to -0.11)	0.65 (0.58 − 0.71)
Republic of Niue	0.22 (0.21 − 0.23)	-0.18 (-0.31 to -0.05)	-0.14 (-0.19 to -0.10)	0.53 (0.46 − 0.60)	1.17 (0.99 − 1.36)	0.57 (0.53 − 0.61)
Republic of Palau	0.17 (0.16 − 0.18)	-0.22 (-0.28 to -0.17)	-0.14 (-0.19 to -0.08)	-0.10 (-0.17 to -0.03)	0.39 (0.25 − 0.53)	-0.54 (-0.58 to -0.49)
Republic of San Marino	0.29 (0.25 − 0.32)	0.44 (0.15 − 0.72)	-0.40 (-0.48 to -0.31)	-1.22 (-1.59 to -0.86)	-0.52 (-0.89 to -0.16)	-1.31 (-1.71 to -0.91)
Republic of the Gambia	0.04 (0.03 − 0.06)	-0.52 (-0.73 to -0.31)	-0.26 (-0.41 to -0.11)	0.03 (-0.09 to 0.16)	0.35 (0.14 − 0.56)	0.74 (0.65 − 0.84)

Location	BPH	UTI	Urolithiasis	Bladder cancer	Kidney cancer	Prostate cancer
Romania	0.15 (0.14 − 0.16)	-1.66 (-2.49 to -0.81)	0.19 (-0.12 to 0.50)	0.53 (0.41 − 0.65)	1.73 (1.49 − 1.97)	1.36 (1.22 − 1.50)
Russian Federation	0.02 (0.00 − 0.04)	-0.19 (-0.97 to 0.60)	-0.54 (-0.85 to -0.23)	-1.05 (-1.39 to -0.70)	-0.41 (-0.63 to -0.20)	2.16 (2.03 − 2.29)
Rwanda	0.00 (-0.01 to 0.01)	-2.81 (-3.16 to -2.46)	-2.07 (-2.36 to -1.78)	-2.01 (-2.26 to -1.75)	-0.63 (-0.82 to -0.44)	-0.49 (-0.62 to -0.36)
Saint Kitts and Nevis	0.14 (0.13 − 0.16)	3.60 (3.01 − 4.20)	0.90 (0.76 − 1.03)	0.09 (-0.14 to 0.32)	0.10 (-0.15 to 0.36)	0.73 (0.53 − 0.92)
Saint Lucia	0.19 (0.17 − 0.21)	2.95 (2.41 − 3.49)	1.55 (1.19 − 1.92)	-0.90 (-1.13 to -0.67)	0.04 (-0.22 to 0.30)	-0.75 (-1.04 to -0.47)
Saint Vincent and the Grenadines	0.18 (0.16 − 0.19)	3.15 (2.88 − 3.43)	0.87 (0.59 − 1.15)	0.35 (0.12 − 0.59)	0.17 (-0.03 to 0.38)	0.59 (0.32 − 0.87)
Samoa	0.13 (0.11 − 0.14)	-0.68 (-0.79 to -0.56)	-0.20 (-0.23 to -0.16)	-0.27 (-0.32 to -0.21)	-0.09 (-0.16 to -0.02)	-0.17 (-0.23 to -0.11)
Sao Tome and Principe	0.06 (0.05 − 0.06)	-0.6 (-0.73 to -0.46)	-0.29 (-0.42 to -0.17)	0.74 (0.64 − 0.84)	-0.32 (-0.82 to 0.18)	1.14 (1.01 − 1.27)
Saudi Arabia	0.15 (0.14 − 0.16)	-0.21 (-0.47 to 0.06)	0.48 (0.41 − 0.55)	-0.65 (-0.79 to -0.52)	2.03 (1.85 − 2.21)	-0.25 (-0.53 to 0.03)
Senegal	0.09 (0.07 − 0.10)	-1.13 (-1.28 to -0.98)	-0.77 (-0.88 to -0.66)	-0.67 (-0.76 to -0.58)	1.36 (1.18 − 1.55)	2.14 (2.01 − 2.27)
Serbia	0.18 (0.17 − 0.19)	-1.01 (-1.25 to -0.78)	-0.39 (-0.67 to -0.10)	-0.29 (-0.37 to -0.21)	0.17 (0.07 − 0.26)	0.11 (-0.05 to 0.27)
Seychelles	0.14 (0.12 − 0.16)	2.61 (2.18 − 3.03)	0.99 (0.71 − 1.27)	-0.49 (-0.69 to -0.29)	-0.75 (-1.42 to -0.08)	0.90 (0.52 − 1.28)
Sierra Leone	0.08 (0.07 − 0.09)	-1.39 (-1.49 to -1.28)	-0.57 (-0.63 to -0.51)	-0.69 (-0.76 to -0.62)	1.39 (1.22 − 1.56)	1.81 (1.68 − 1.94)
Singapore	0.06 (0.04 − 0.08)	-0.86 (-1.28 to -0.43)	0.13 (0.05 − 0.20)	-1.82 (-2.03 to -1.61)	-0.45 (-0.83 to -0.06)	-0.53 (-0.74 to -0.31)
Slovakia	0.12 (0.12 − 0.13)	-1.18 (-1.42 to -0.94)	-1.77 (-2.28 to -1.26)	-0.53 (-0.60 to -0.45)	0.87 (0.60 − 1.15)	0.42 (0.32 − 0.51)

Location	BPH	UTI	Urolithiasis	Bladder cancer	Kidney cancer	Prostate cancer
Slovenia	0.11 (0.09 − 0.13)	-0.40 (-1.74 to 0.95)	-1.94 (-2.24 to -1.63)	-0.41 (-0.60 to -0.23)	0.75 (0.39 − 1.11)	0.26 (-0.10 to 0.62)
Socialist Republic of Viet Nam	0.19 (0.12 − 0.25)	0.06 (0.00 − 0.13)	0.64 (0.49 − 0.80)	0.46 (0.42 − 0.51)	2.17 (1.98 − 2.36)	0.89 (0.76 − 1.03)
Solomon Islands	0.18 (0.16 − 0.20)	-0.13 (-0.17 to -0.09)	-0.22 (-0.26 to -0.19)	0.58 (0.51 − 0.65)	0.55 (0.49 − 0.62)	0.71 (0.66 − 0.76)
Somalia	0.00 (-0.01 to 0.01)	-0.07 (-0.19 to 0.06)	-0.12 (-0.20 to -0.04)	-0.31 (-0.36 to -0.27)	-0.35 (-0.47 to -0.22)	-0.15 (-0.20 to -0.10)
South Africa	0.07 (0.05 to 0.10)	-0.22 (-0.82 to 0.39)	-0.05 (-0.41 to 0.31)	0.25 (0.05 − 0.46)	0.94 (0.84 − 1.04)	0.97 (0.77 − 1.16)
South Sudan	-0.01 (-0.03 to 0.01)	0.24 (-0.10 to 0.57)	-0.16 (-0.29 to -0.02)	-0.25 (-0.41 to -0.1)	0.62 (0.38 − 0.86)	-0.12 (-0.20 to -0.05)
Spain	0.53 (0.38 − 0.68)	2.36 (2.07 to 2.66)	0.26 (0.05 − 0.47)	-1.21 (-1.36 to -1.06)	0.38 (0.29 − 0.48)	-2.05 (-2.19 to -1.91)
Sri Lanka	0.14 (0.13 − 0.16)	1.57 (1.20 − 1.95)	0.03 (-0.08 to 0.15)	-0.21 (-0.41 to -0.01)	-6.82 (-7.94 to -5.69)	0.14 (0.01 − 0.26)
Sudan	0.13 (0.11 − 0.14)	-0.12 (-0.19 to -0.06)	1.68 (1.45 − 1.90)	-0.56 (-0.64 to -0.49)	0.97 (0.87 − 1.07)	0.70 (0.63 − 0.77)
Suriname	0.25 (0.22 − 0.27)	2.16 (1.79 − 2.53)	1.77 (1.47 − 2.08)	0.21 (0.03 − 0.39)	0.47 (0.35 − 0.60)	0.82 (0.59 − 1.05)
Sweden	-0.12 (-0.21 to -0.02)	-1.56 (-2.00 to -1.11)	-0.10 (-0.50 to 0.31)	-0.43 (-0.68 to -0.18)	-1.86 (-2.06 to -1.67)	-1.62 (-1.91 to -1.34)
Switzerland	0.14 (0.13 − 0.14)	2.01 (1.45 − 2.57)	-0.66 (-1.11 to -0.21)	-0.08 (-0.35 to 0.19)	-0.04 (-0.40 to 0.32)	-2.53 (-2.66 to -2.40)
Syrian Arab Republic	0.08 (0.07 − 0.09)	-1.74 (-2.14 to -1.33)	-0.67 (-0.82 to -0.52)	-0.18 (-0.27 to -0.08)	0.25 (0.13 − 0.36)	0.34 (0.24 − 0.44)
Taiwan (Province of China)	0.09 (0.08 − 0.10)	3.90 (3.00 − 4.80)	0.94 (0.46 − 1.42)	-1.37 (-1.63 to -1.12)	2.09 (1.40 − 2.78)	1.69 (1.33 − 2.06)
Tajikistan	0.10 (0.08 − 0.12)	-0.99 (-1.22 to -0.75)	-0.82 (-1.01 to -0.64)	-1.09 (-1.35 to -0.83)	-0.89 (-0.99 to -0.78)	-1.5 (-1.70 to -1.31)

Location	BPH	UTI	Urolithiasis	Bladder cancer	Kidney cancer	Prostate cancer
Thailand	0.04 (0.02 − 0.06)	-0.35 (-0.72 to 0.02)	-1.92 (-2.20 to -1.64)	-1.22 (-1.37 to -1.06)	1.06 (0.96 − 1.16)	0.02 (-0.09 to 0.13)
The former Yugoslav Republic of Macedonia	0.18 (0.16 − 0.21)	-0.88 (-1.01 to -0.76)	-0.49 (-0.52 to -0.45)	-0.02 (-0.28 to 0.24)	1.67 (1.31 − 2.04)	0.77 (0.47 − 1.08)
Timor-Leste	0.09 (0.08 − 0.10)	-0.16 (-0.37 to 0.05)	-0.06 (-0.17 to 0.06)	-0.10 (-0.31 to 0.11)	0.15 (-0.07 to 0.37)	1.00 (0.95 − 1.06)
Togo	0.05 (0.03 − 0.06)	-0.62 (-0.70 to -0.55)	-0.25 (-0.35 to -0.16)	-0.64 (-0.79 to -0.49)	1.52 (1.46 − 1.58)	2.10 (1.96 − 2.25)
Tokelau	0.15 (0.13 − 0.17)	-0.40 (-0.54 to -0.26)	-0.20 (-0.23 to -0.17)	0.11 (0.05 − 0.16)	1.34 (0.85 − 1.84)	0.30 (0.25 − 0.35)
Tonga	0.13 (0.11 − 0.14)	0.08 (-0.12 to 0.29)	-0.06 (-0.12 to -0.01)	0.61 (0.47 − 0.75)	0.83 (0.66 − 0.99)	0.28 (0.17 − 0.38)
Trinidad and Tobago	0.13 (0.11 − 0.15)	3.00 (2.35 − 3.65)	2.28 (1.83 − 2.73)	-0.21 (-0.36 to -0.06)	0.13 (-0.01 to 0.26)	-0.57 (-0.81 to -0.34)
Tunisia	0.11 (0.10 to 0.12)	-0.10 (-0.25 to 0.05)	1.23 (1.06 to 1.41)	-0.32 (-0.41 to -0.22)	0.42 (0.39 − 0.44)	0.29 (0.21 − 0.37)
Turkey	0.10 (0.08 − 0.12)	-0.60 (-0.95 to -0.25)	-0.36 (-0.50 to -0.22)	-0.90 (-1.12 to -0.67)	-0.53 (-0.64 to -0.41)	-0.69 (-0.94 to -0.45)
Turkmenistan	0.07 (0.05 to 0.10)	4.88 (4.59 − 5.18)	0.55 (0.44 − 0.66)	-1.50 (-1.84 to -1.16)	3.47 (2.90 − 4.05)	0.85 (0.55 − 1.14)
Tuvalu	0.18 (0.16 − 0.19)	-0.57 (-0.61 to -0.53)	-0.26 (-0.29 to -0.24)	0.55 (0.49 − 0.61)	0.96 (0.91 − 1.00)	0.66 (0.59 − 0.73)
Uganda	0.02 (0.00 − 0.03)	-0.25 (-0.48 to -0.01)	-0.27 (-0.46 to -0.09)	-0.55 (-0.74 to -0.36)	1.25 (1.12 − 1.39)	0.34 (0.17 − 0.51)
Ukraine	0.02 (0.00 − 0.05)	-0.76 (-1.03 to -0.49)	-1.26 (-1.40 to -1.11)	-0.07 (-0.35 to 0.21)	0.23 (-0.02 to 0.48)	0.03 (-0.13 to 0.20)
United Arab Emirates	0.15 (0.12 − 0.17)	1.59 (0.95 − 2.24)	1.03 (0.76 − 1.29)	0.09 (-0.39 to 0.57)	1.85 (1.44 − 2.27)	1.05 (0.54 − 1.56)
United Kingdom of Great Britain and Northern Ireland	0.31 (0.25 − 0.37)	3.12 (2.02 − 4.23)	0.73 (0.56 − 0.90)	-1.61 (-1.75 to -1.47)	0.06 (-0.05 to 0.17)	-1.21 (-1.30 to -1.12)

Location	BPH	UTI	Urolithiasis	Bladder cancer	Kidney cancer	Prostate cancer
United Republic of Tanzania	0.05 (0.04 − 0.06)	-0.69 (-0.74 to -0.64)	-0.74 (-0.79 to -0.70)	-0.87 (-0.94 to -0.80)	0.58 (0.45 − 0.71)	-0.44 (-0.53 to -0.36)
United States of America	0.20 (0.12 − 0.27)	0.25 (0.03 − 0.47)	-0.32 (-0.65 to 0.00)	-0.24 (-0.35 to -0.13)	-0.89 (-1.01 to -0.77)	-2.13 (-2.28 to -1.99)
United States Virgin Islands	0.19 (0.17 − 0.22)	-0.06 (-0.36 to 0.25)	0.64 (0.33 − 0.95)	-1.11 (-1.31 to -0.91)	0.08 (-0.10 to 0.25)	-1.53 (-1.90 to -1.16)
Uruguay	0.26 (0.20 − 0.32)	6.21 (5.35 − 7.07)	0.66 (0.42 − 0.90)	-0.84 (-0.96 to -0.72)	0.81 (0.71 − 0.91)	-0.41 (-0.70 to -0.11)
Uzbekistan	0.09 (0.07 − 0.10)	0.72 (-0.08 to 1.52)	0.05 (-0.01 to 0.10)	0.20 (-0.01 to 0.41)	0.97 (0.84 − 1.09)	0.18 (-0.61 to 0.97)
Vanuatu	0.17 (0.15 − 0.19)	-0.11 (-0.17 to -0.04)	-0.21 (-0.26 to -0.16)	0.43 (0.38 − 0.48)	0.5 (0.45 − 0.56)	0.75 (0.70 − 0.80)
Yemen	0.08 (0.07 − 0.09)	-0.01 (-0.08 to 0.06)	1.57 (1.37 − 1.78)	-0.22 (-0.27 to -0.16)	0.26 (0.17 − 0.35)	0.87 (0.82 − 0.92)
Zambia	0.00 (0.00 − 0.01)	0.01 (-0.15 to 0.18)	-0.41 (-0.61 to -0.20)	0.43 (0.30 − 0.55)	1.96 (1.58 − 2.35)	2.58 (2.24 − 2.92)
Zimbabwe	0.10 (0.09 − 0.12)	1.84 (1.37 − 2.32)	1.19 (0.78 − 1.60)	0.93 (0.60 to 1.27)	2.94 (2.17 − 3.72)	1.16 (0.86 − 1.46)

BPH benign prostatic hyperplasia, *UTI* urinary tract infections, *EAPC* estimated annual percentage change, *ASDR* age-standardized DALYs rate

High-middle SDI	30.82 (26.61 – 35.17)	7.23 (-0.90 – 16.47)	21.90 (8.72 – 34.55)	11.75 (7.36 – 16.16)	0.05 (0.01 – 0.09)	3.89 (1.86 – 6.16)

Table S11 Percentage of urologic cancers deaths attributed to risk factors in 2021

Location	Bladder cancer		Kidney cancer			Prostate cancer
	**Smoking**	**High FPG**	**High BMI**	**Smoking**	**Occupational exposure to trichloroethylene**	**Smoking**
Global	26.48 (22.78 – 30.40)	7.91 (-0.99 to 18.02)	20.07 (7.96 – 31.73)	10.06 (6.05 – 14.35)	0.05 (0.01 – 0.09)	3.00 (1.42 – 4.92)
High SDI	23.58 (19.74 – 28.00)	8.88 (-1.13 to 20.00)	22.21 (8.91 – 34.86)	10.83 (6.10 – 16.42)	0.02 (0.00 – 0.04)	3.00 (1.33 – 5.08)
Middle SDI	29.67 (25.63 – 33.97)	7.25 (-0.90 to 16.74)	17.44 (6.84 – 27.64)	8.8 (5.47 – 12.12)	0.10 (0.02 – 0.18)	2.99 (1.40 – 4.76)
Low-middle SDI	23.86 (20.65 – 27.50)	8.28 (-1.01 to 19.02)	13.62 (5.23 – 22.18)	5.99 (3.77 – 8.38)	0.08 (0.02 – 0.14)	2.54 (1.20 – 4.02)
Low SDI	12.10 (10.25 – 13.97)	6.42 (-0.76 to 14.94)	7.63 (2.90 – 12.36)	2.02 (1.25 – 2.90)	0.05 (0.01 – 0.09)	1.26 (0.60 – 2.06)
Andean Latin America	10.24 (8.16 – 12.31)	7.71 (-0.94 to 17.75)	21.99 (8.66 – 35.18)	2.52 (1.37 – 3.99)	0.12 (0.03 – 0.22)	1.69 (0.76 – 2.93)
Australasia	13.97 (10.79 – 17.66)	6.58 (-0.93 to 14.04)	25.69 (10.6 – 40.79)	6.94 (3.65 – 11.28)	0.02 (0.00 – 0.03)	1.50 (0.63 – 2.71)
Caribbean	20.80 (17.29 – 24.48)	9.71 (-1.18 to 22.27)	20.41 (8.14 – 32.91)	7.54 (4.46 – 11.31)	0.08 (0.02 – 0.16)	2.44 (1.11 – 4.04)
Central Asia	32.86 (29.17 – 36.96)	6.53 (-0.77 to 15.08)	22.66 (9.09 – 36.14)	9.92 (6.41 – 13.66)	0.08 (0.02 – 0.15)	5.20 (2.52 – 8.00)
Central Europe	27.30 (23.57 – 31.40)	9.34 (-1.18 to 21.08)	25.22 (10.2 – 40.19)	11.52 (7.04 – 16.46)	0.02 (0.00 – 0.03)	3.25 (1.53 – 5.16)
Central Latin America	12.88 (10.82 – 15.27)	9.40 (-1.16 to 21.77)	25.90 (10.58 – 40.74)	3.02 (1.78 – 4.46)	0.12 (0.03 – 0.21)	1.45 (0.67 – 2.39)
Central Sub-Saharan Africa	8.96 (7.51 – 10.58)	7.28 (-0.93 to 16.39)	12.42 (4.62 – 19.93)	2.10 (1.26 – 3.14)	0.06 (0.01 – 0.10)	1.19 (0.55 – 1.92)
East Asia	37.55 (32.93 – 42.44)	5.94 (-0.78 to 13.48)	14.41 (5.52 – 23.26)	13.70 (8.61 – 18.29)	0.10 (0.02 – 0.19)	5.04 (2.34 – 7.69)
Eastern Europe	31.60 (27.66 – 35.75)	5.19 (-0.65 to 11.68)	25.55 (10.39 – 40.61)	11.85 (7.61 – 16.51)	0.01 (0.00 – 0.02)	5.02 (2.39 – 8.08)
Eastern Sub-Saharan Africa	8.61 (7.02 – 10.16)	4.56 (-0.52 to 10.82)	8.52 (3.23 – 14.02)	1.30 (0.79 – 1.87)	0.07 (0.01 – 0.12)	1.30 (0.61 – 2.04)
High-income Asia Pacific	22.43 (18.53 – 26.51)	8.12 (-1.10 to 17.33)	10.72 (4.05 – 17.20)	9.80 (5.70 – 14.30)	0.02 (0.00 – 0.03)	2.84 (1.27 – 4.80)
High-income North America	22.93 (18.80 – 28.01)	11.44 (-1.51 to 25.55)	27.82 (11.50 – 43.45)	11.68 (6.26 – 18.33)	0.02 (0.00 – 0.03)	3.14 (1.33 – 5.61)
North Africa and Middle East	31.01 (26.98 – 34.98)	10.01 (-1.24 to 22.65)	26.72 (11.01 – 41.28)	10.63 (6.66 – 14.93)	0.07 (0.01 – 0.12)	3.59 (1.70 – 5.75)
Oceania	16.62 (13.67 – 19.85)	9.33 (-1.18 to 21.36)	17.53 (7.10 – 28.68)	4.43 (2.83 – 6.54)	0.06 (0.01 – 0.10)	1.90 (0.82 – 3.11)
South Asia	21.37 (18.31 – 24.34)	8.74 (-1.08 to 19.81)	8.79 (3.22 – 14.46)	5.14 (3.21 – 7.16)	0.07 (0.02 – 0.12)	2.67 (1.25 – 4.18)
Southeast Asia	28.95 (25.33 – 32.34)	7.67 (-0.88 to 18.18)	10.41 (3.89 – 16.82)	7.78 (4.89 – 10.69)	0.10 (0.02 – 0.19)	3.4 (1.62 – 5.18)

Location	Bladder cancer		Kidney cancer			Prostate cancer
	Smoking	High FPG	High BMI	Smoking	Occupational exposure to trichloroethylene	Smoking
Southern Latin America	19.46 (16.03 − 23.26)	8.51 (-1.02 to 19.33)	26.02 (10.61 − 41.20)	8.72 (5.02 − 12.86)	0.09 (0.02 − 0.17)	1.87 (0.83 − 3.14)
Southern Sub-Saharan Africa	16.75 (14.22 − 19.36)	7.08 (-0.83 to 16.78)	22.87 (9.20 − 36.46)	4.05 (2.52 − 5.68)	0.03 (0.01 − 0.05)	1.72 (0.78 − 2.77)
Tropical Latin America	21.34 (17.48 − 25.7)	7.85 (-1.00 to 18.06)	23.05 (9.09 − 36.99)	8.69 (4.76 − 13.54)	0.09 (0.02 − 0.17)	3.16 (1.40 − 5.57)
Western Europe	23.54 (19.52 − 27.97)	7.66 (-0.92 to 17.75)	21.96 (8.69 − 35.63)	10.58 (5.88 − 16.22)	0.01 (0.00 − 0.03)	2.98 (1.33 − 5.12)
Western Sub-Saharan Africa	7.66 (6.25 − 9.28)	6.35 (-0.76 to 14.94)	9.28 (3.64 − 15.17)	0.86 (0.49 − 1.3)	0.03 (0.01 − 0.07)	0.89 (0.40 − 1.50)

BMI body mass index, *FPG* fasting plasma glucose, *SDI* sociodemographic index

Table S12 Percentage of urologic cancers DALYs attributed to risk factors in 2021

Location	Bladder cancer		Kidney cancer			Prostate cancer
	Smoking	High FPG	High BMI	Smoking	Occupational exposure to trichloroethylene	Smoking
Global	28.15<br>(24.42 − 31.95)	7.36<br>(-0.93 to 16.74)	19.46<br>(7.76 − 31.03)	9.53<br>(5.92 − 13.42)	0.06<br>(0.01 − 0.12)	3.46<br>(1.65 − 5.56)
High SDI	26.51<br>(22.51 − 30.86)	8.62<br>(-1.11 to 19.36)	23.23<br>(9.38 − 36.48)	11.37<br>(6.61 − 16.82)	0.03<br>(0.01 − 0.05)	3.55<br>(1.62 − 5.88)
High-middle SDI	26.08<br>(21.90 − 30.81)	6.79<br>(-0.85 to 15.30)	21.92<br>(8.76 − 34.43)	11.97<br>(7.64 − 16.42)	0.06<br>(0.01 − 0.12)	4.62<br>(2.23 − 7.22)
Middle SDI	29.81<br>(25.84 − 33.98)	6.67<br>(-0.84 to 15.31)	17.16<br>(6.75 − 27.38)	7.78<br>(4.88 − 10.64)	0.11<br>(0.02 − 0.20)	3.36<br>(1.60 − 5.30)
Low-middle SDI	23.36<br>(20.34 − 26.73)	7.39<br>(-0.91 to 16.80)	12.36<br>(4.77 − 20.19)	4.67<br>(2.98 − 6.47)	0.08<br>(0.02 − 0.14)	2.77<br>(1.31 − 4.34)
Low SDI	11.63<br>(9.86 − 13.45)	5.52<br>(-0.66 to 12.67)	5.32<br>(2.06 − 8.63)	1.18<br>(0.72 − 1.70)	0.04<br>(0.01 − 0.08)	1.41<br>(0.67 − 2.29)
Andean Latin America	10.18<br>(8.31 − 12.16)	6.82<br>(-0.84 to 15.62)	21.81<br>(8.67 − 34.89)	1.96<br>(1.13 − 3.01)	0.14<br>(0.03 − 0.25)	1.85<br>(0.85 − 3.10)
Australasia	16.32<br>(13.06 − 20.24)	6.53<br>(-0.92 to 13.82)	26.43<br>(10.91 − 41.64)	7.20<br>(3.99 − 11.22)	0.02<br>(0.01 − 0.04)	1.86<br>(0.82 − 3.14)
Caribbean	22.09<br>(18.64 − 25.60)	9.02<br>(-1.12 to 20.60)	19.69<br>(7.96 − 31.87)	6.37<br>(3.84 − 9.24)	0.09<br>(0.02 − 0.17)	2.81<br>(1.29 − 4.53)
Central Asia	33.26<br>(29.62 − 37.28)	5.94<br>(-0.72 to 13.64)	21.61<br>(8.73 − 34.47)	9.15<br>(5.93 − 12.51)	0.09<br>(0.02 − 0.16)	5.70<br>(2.77 − 8.74)
Central Europe	30.75<br>(26.86 − 34.80)	8.9<br>(-1.13 to 20.01)	25.48<br>(10.32 − 40.45)	12.3<br>(7.71 − 17.07)	0.02<br>(0.00 − 0.04)	3.79<br>(1.78 − 5.94)
Central Latin America	12.69<br>(10.76 − 14.91)	8.72<br>(-1.09 to 19.94)	25.81<br>(10.61 − 40.36)	2.47<br>(1.46 − 3.56)	0.13<br>(0.03 − 0.24)	1.64<br>(0.77 − 2.65)
Central Sub-Saharan Africa	9.17<br>(7.69 − 10.72)	6.39<br>(-0.84 to 14.15)	10.61<br>(3.85 − 17.13)	1.61<br>(0.95 − 2.39)	0.05<br>(0.01 − 0.10)	1.36<br>(0.62 − 2.18)
East Asia	38.19<br>(33.45 − 42.54)	5.57<br>(-0.73 to 12.50)	14.55<br>(5.58 − 23.63)	12.9<br>(8.29 − 17.22)	0.12<br>(0.03 − 0.22)	5.82<br>(2.72 − 8.81)
Eastern Europe	34.83<br>(30.73 − 38.95)	4.87<br>(-0.62 to 10.90)	25.48<br>(10.38 − 40.33)	12.57<br>(8.18 − 17.31)	0.02<br>(0.00 − 0.03)	5.81<br>(2.77 − 9.26)
Eastern Sub-Saharan Africa	8.30<br>(6.75 − 9.77)	3.71<br>(-0.43 to 8.71)	6.19<br>(2.35 − 10.14)	0.79<br>(0.48 − 1.15)	0.05<br>(0.01 − 0.10)	1.45<br>(0.67 − 2.27)
High-income Asia Pacific	25.16<br>(21.24 − 29.17)	7.77<br>(-1.05 to 16.78)	11.33<br>(4.29 − 18.18)	10.53<br>(6.33 − 15.17)	0.02<br>(0.01 − 0.04)	3.22<br>(1.48 − 5.23)
High-income North America	26.08<br>(21.90 − 30.81)	11.06<br>(-1.48 to 24.36)	28.64<br>(11.91 − 44.45)	12.16<br>(6.92 − 18.55)	0.02<br>(0.01 − 0.04)	3.81<br>(1.70 − 6.51)
North Africa and Middl East	32.04<br>(28.07 − 35.94)	9.06<br>(-1.13 to 20.33)	25.24<br>(10.42 − 39.06)	9.43<br>(6.02 − 13.04)	0.08<br>(0.02 − 0.14)	4.12<br>(1.96 − 6.55)
Oceania	17.25<br>(14.28 − 20.65)	8.27<br>(-1.05 to 18.70)	15.45<br>(6.21 − 24.23)	3.43<br>(2.07 − 5.10)	0.06<br>(0.01 − 0.10)	2.23<br>(0.95 − 3.6)
South Asia	20.44<br>(17.56 − 23.21)	7.92<br>(-1.00 to 17.95)	8.46<br>(3.11 − 13.82)	4.10<br>(2.61 − 5.66)	0.07<br>(0.02 − 0.13)	2.81<br>(1.31 − 4.35)

Location	Bladder cancer	Kidney cancer				Prostate cancer
	Smoking	High FPG	High BMI	Smoking	Occupational exposure to trichloroethylene	Smoking
Southeast Asia	28.37 (24.97 − 31.73)	6.53 (-0.76 to 15.29)	10.34 (3.89 − 16.58)	6.46 (4.08 − 8.91)	0.11 (0.03 − 0.21)	3.78 (1.82 − 5.72)
Southern Latin America	22.85 (19.16 − 26.48)	7.99 (-0.96 to 18.07)	26.26 (10.72 − 41.35)	8.71 (5.18 − 12.6)	0.11 (0.03 − 0.21)	2.34 (1.08 − 3.81)
Southern Sub-Saharan Africa	16.72 (14.28 − 19.23)	5.93 (-0.70 to 14.03)	20.68 (8.41 − 32.64)	3.37 (2.13 − 4.76)	0.03 (0.01 − 0.06)	2.01 (0.91 − 3.19)
Tropical Latin America	22.02 (18.29 − 26.13)	7.39 (-0.94 to 16.92)	22.71 (8.98 − 36.15)	7.63 (4.3 − 11.72)	0.11 (0.02 − 0.19)	3.51 (1.56 − 6.01)
Western Europe	26.62 (22.53 − 31.04)	7.37 (-0.89 to 16.99)	22.38 (8.89 − 36.24)	11.18 (6.57 − 16.52)	0.02 (0.00 − 0.04)	3.48 (1.59 − 5.74)
Western Sub-Saharan Africa	7.58 (6.20 − 9.12)	5.34 (-0.64 to 12.40)	4.96 (2.00 − 8.18)	0.42 (0.24 − 0.65)	0.02 (0.00 − 0.04)	1.04 (0.48 − 1.74)

BMI body mass index, *FPG* fasting plasma glucose, *SDI* sociodemographic index, *DALYs* disability-adjusted life-years

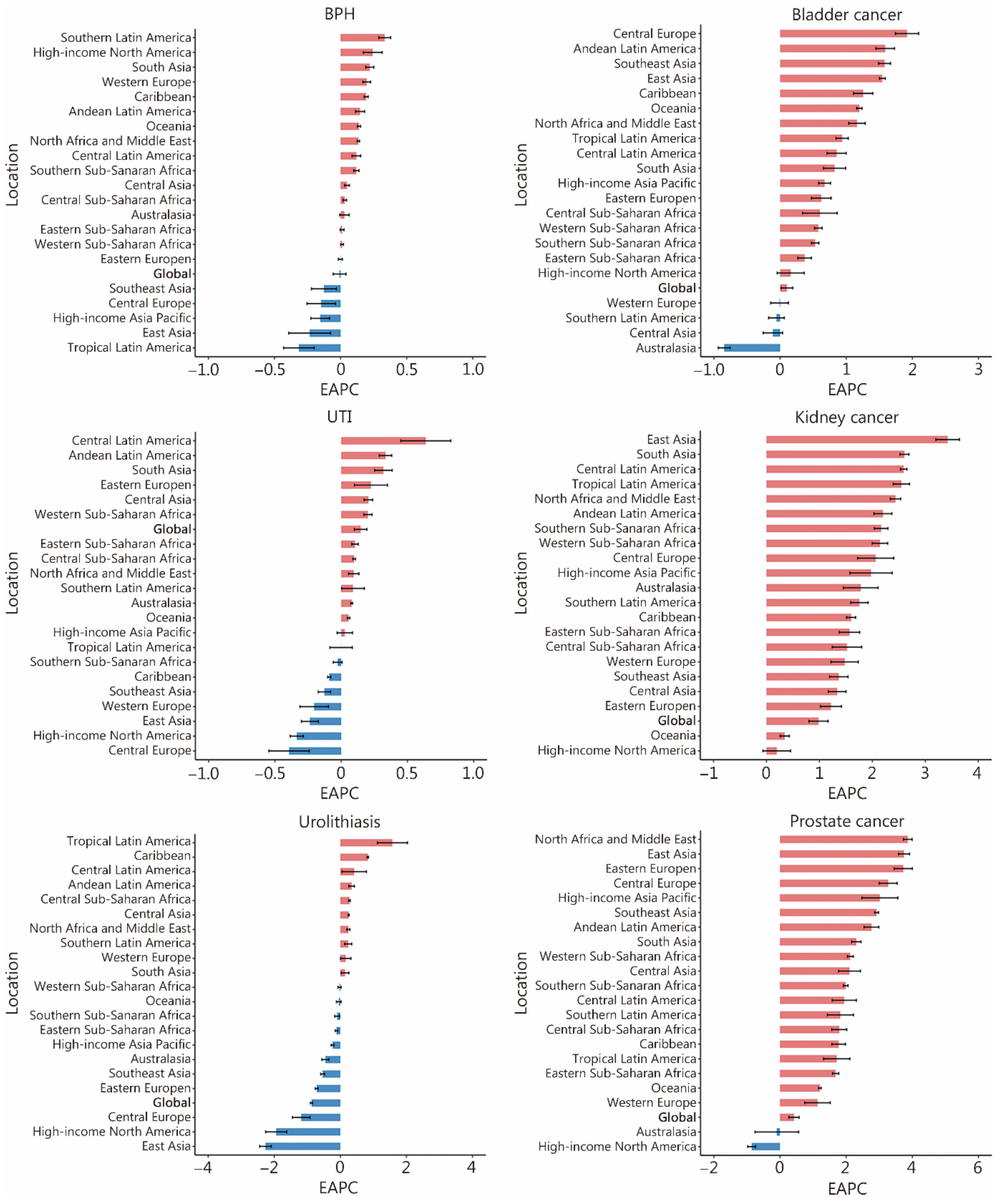

Fig. S1 The EAPC of ASPR for 6 urologic diseases in global and 21 regions. ASPR age-standardized prevalence rate, EAPC estimated annual percentage change, BPH benign prostatic hyperplasia, UTI urinary tract infections

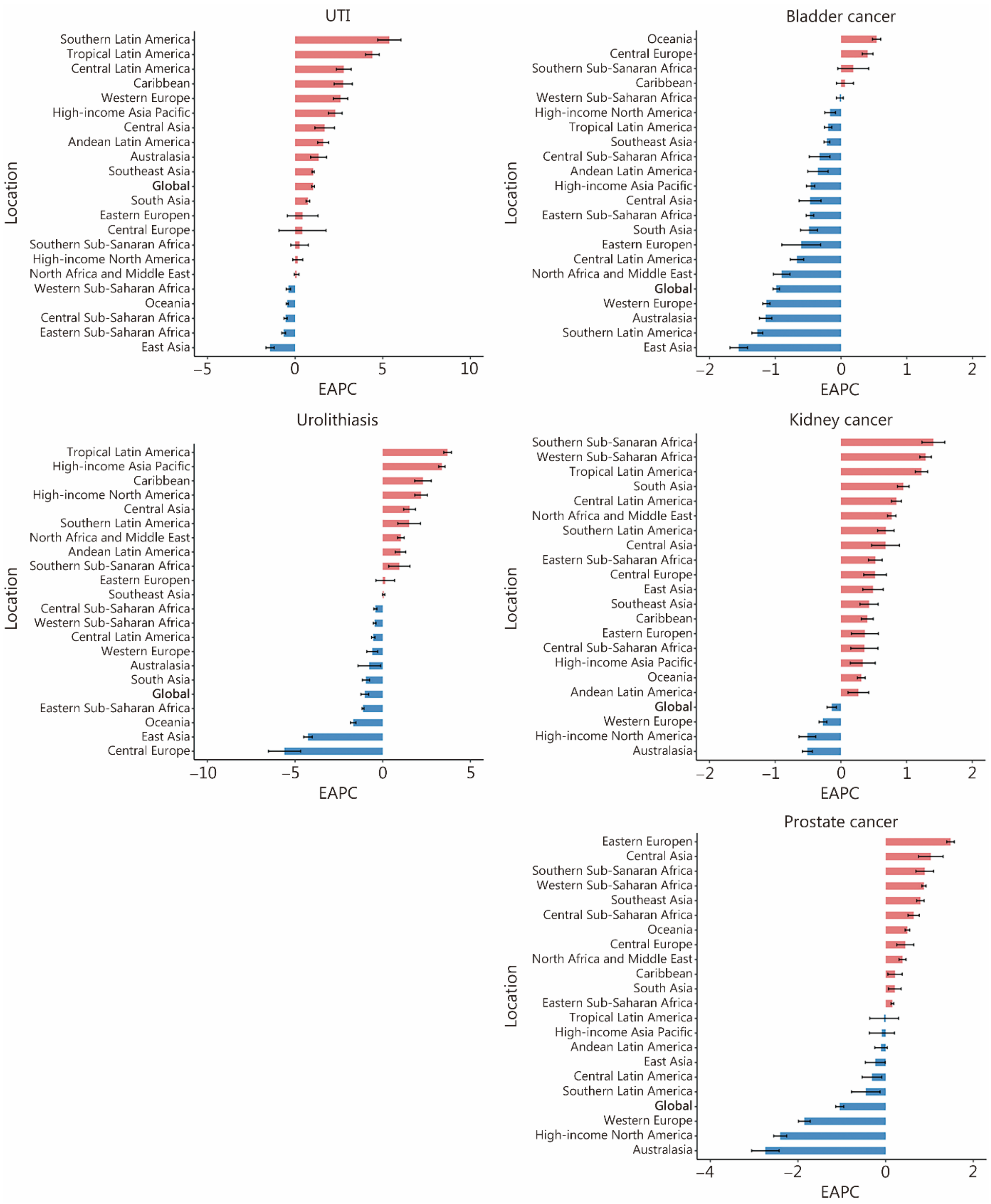

Fig. S2 The EAPC of ASMR for 5 urologic diseases in global and 21 regions. ASMR age-standardized mortality rate, EAPC estimated annual percentage change, UTI urinary tract infections

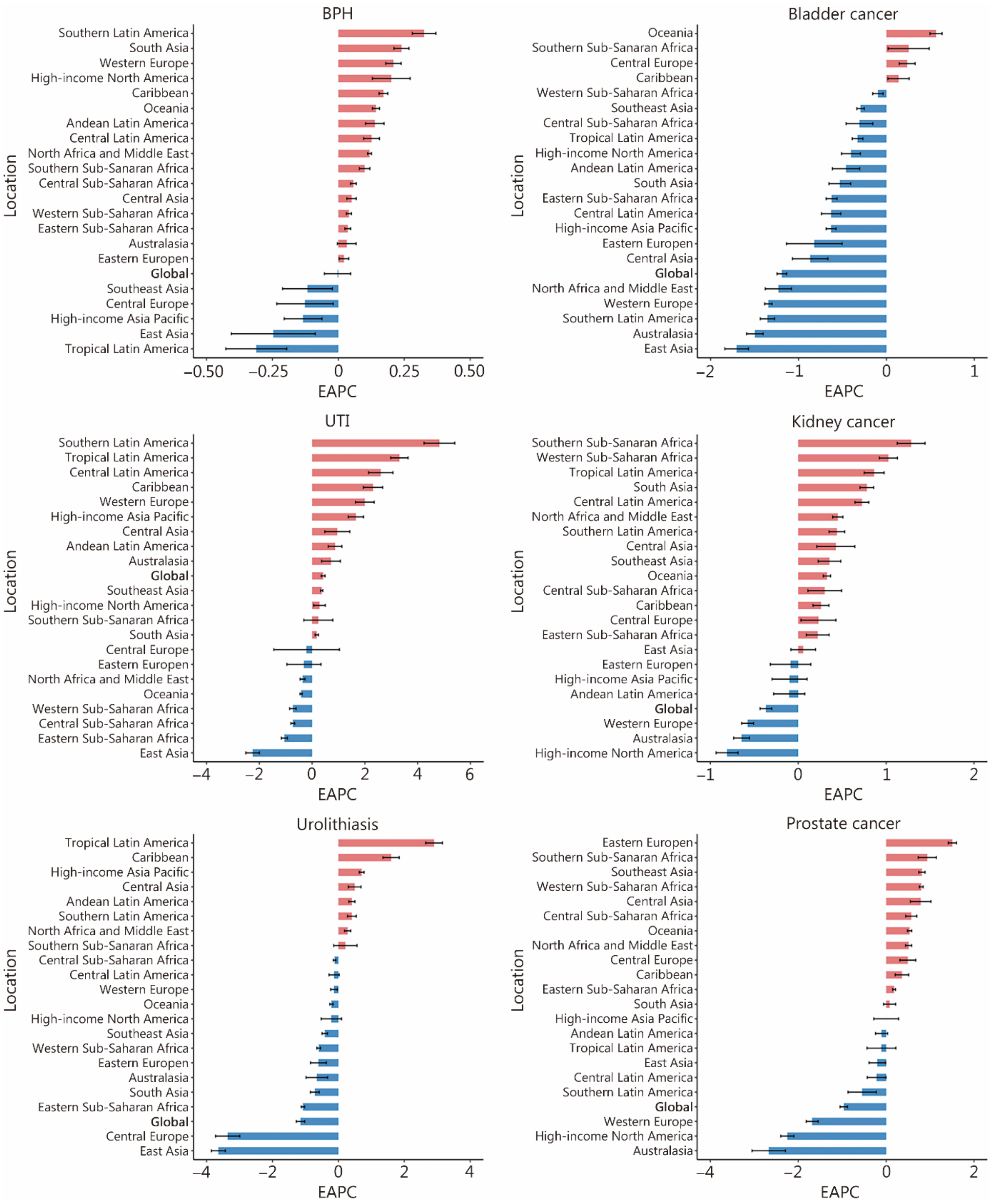

Fig. S3 The EAPC of ASDR for 6 urologic diseases in global and 21 regions. ASDR age-standardized DALYs rate, DALYs disability-adjusted life-years, EAPC estimated annual percentage change, BPH benign prostatic hyperplasia, UTI urinary tract infections

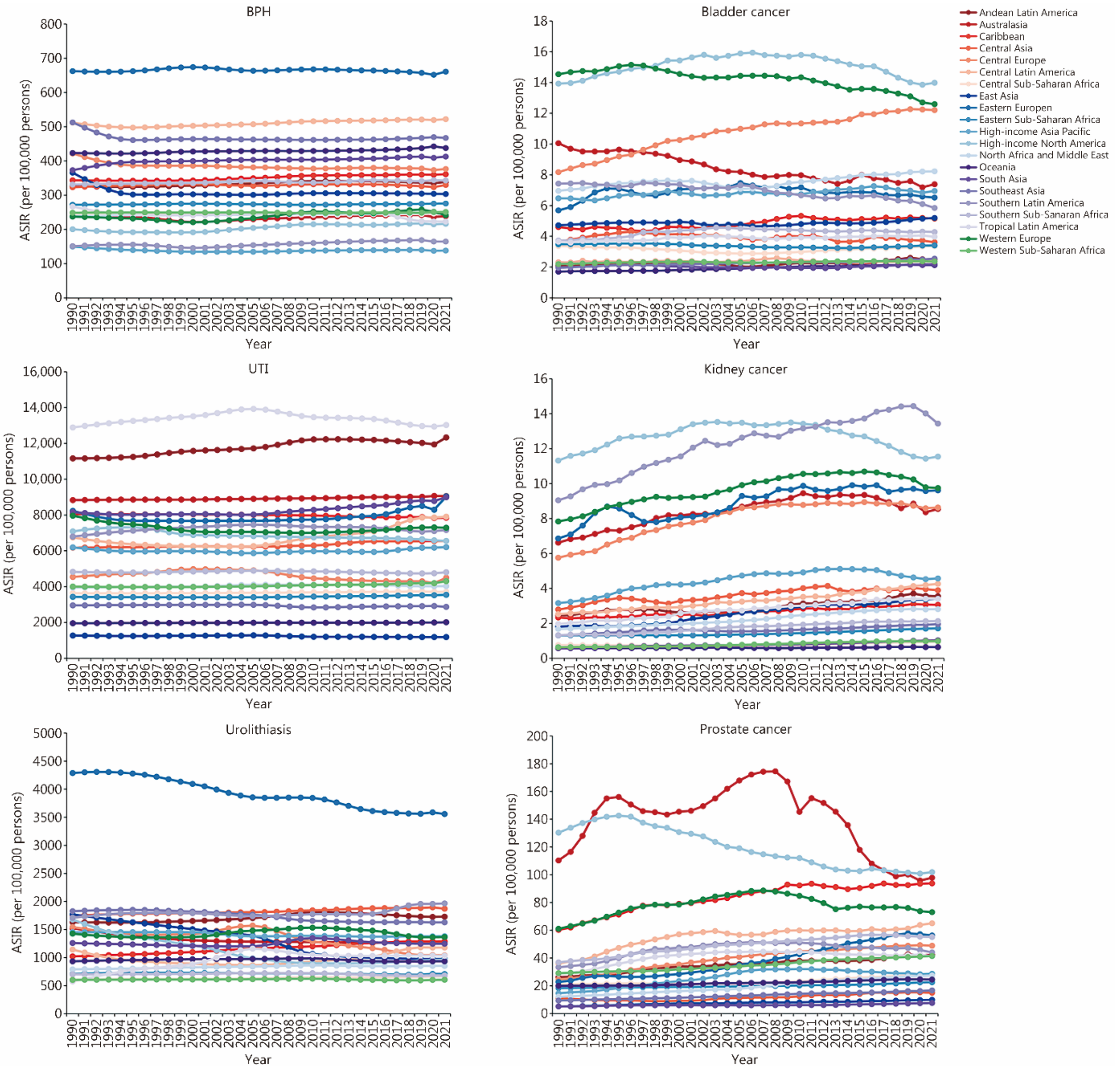

Fig. S4 The ASIR for 6 urologic diseases in 21 regions from 1990 to 2021. ASIR age-standardized incidence rate, BPH benign prostatic hyperplasia, UTI urinary tract infections

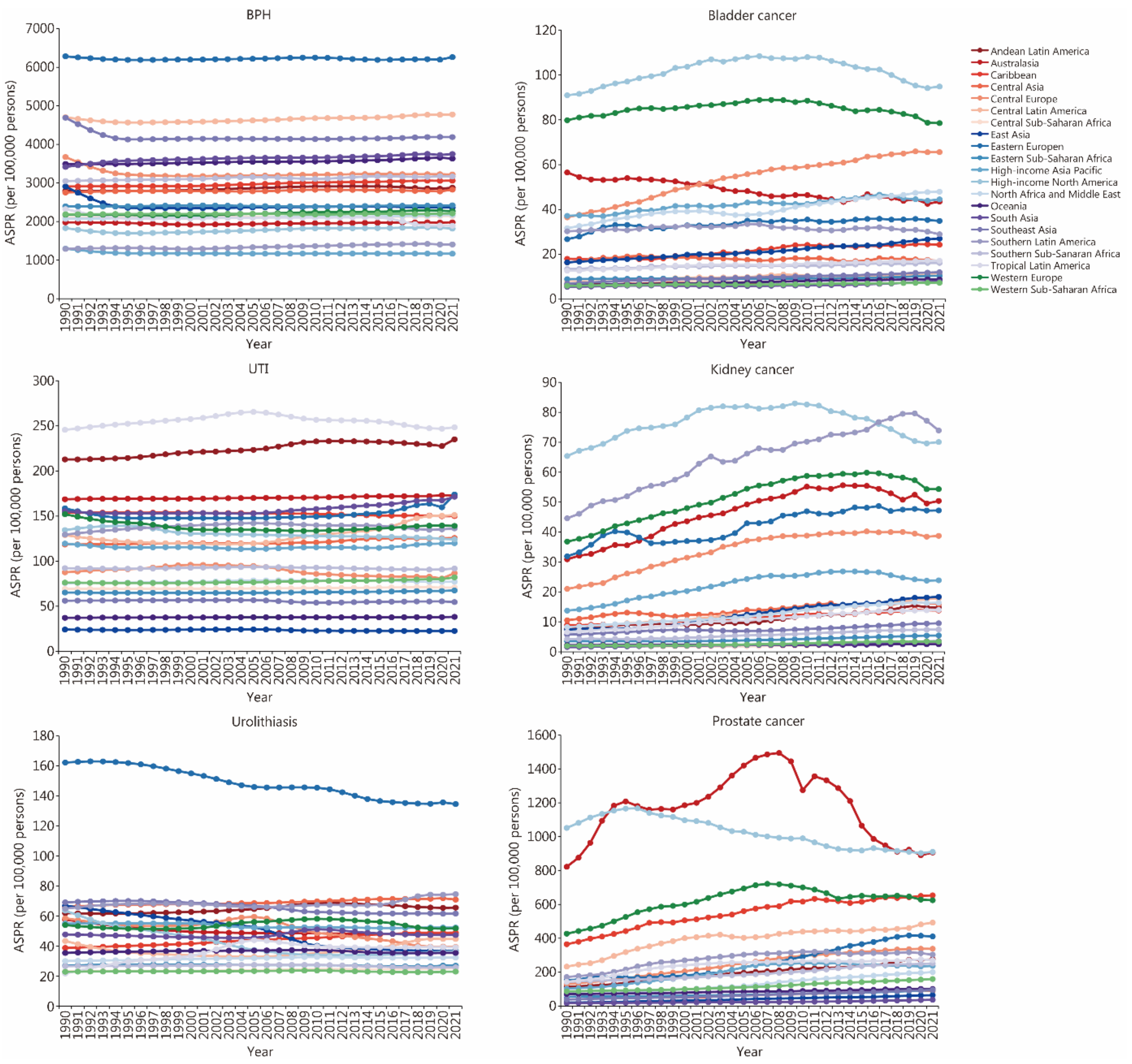

Fig. S5 The ASPR for 6 urologic diseases in 21 regions from 1990 to 2021. ASPR age-standardized prevalence rate, BPH benign prostatic hyperplasia, UTI urinary tract infections

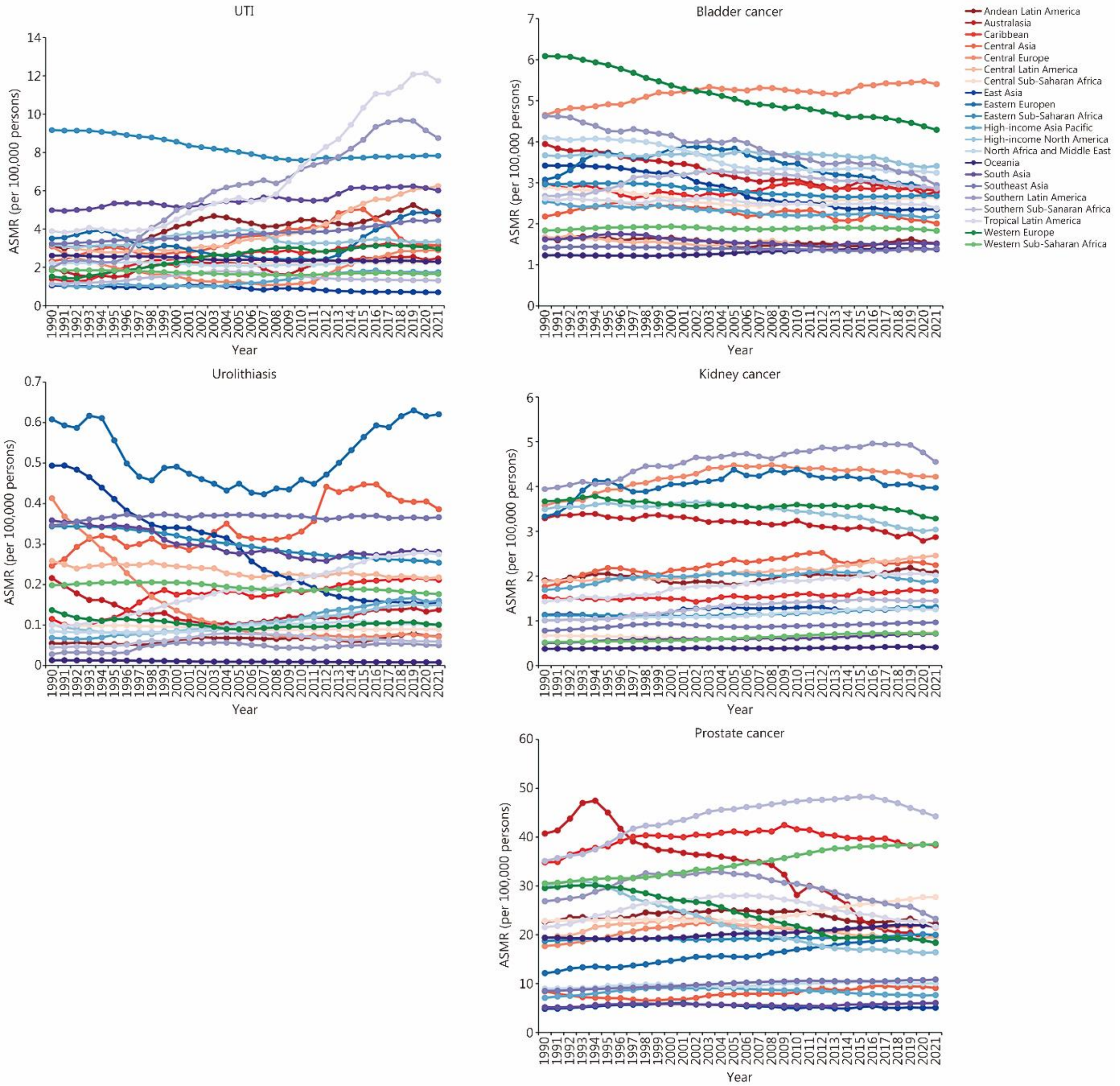

Fig. S6 The ASMR for 5 urologic diseases in 21 regions from 1990 to 2021. ASMR age-standardized mortality rate, UTI urinary tract infections

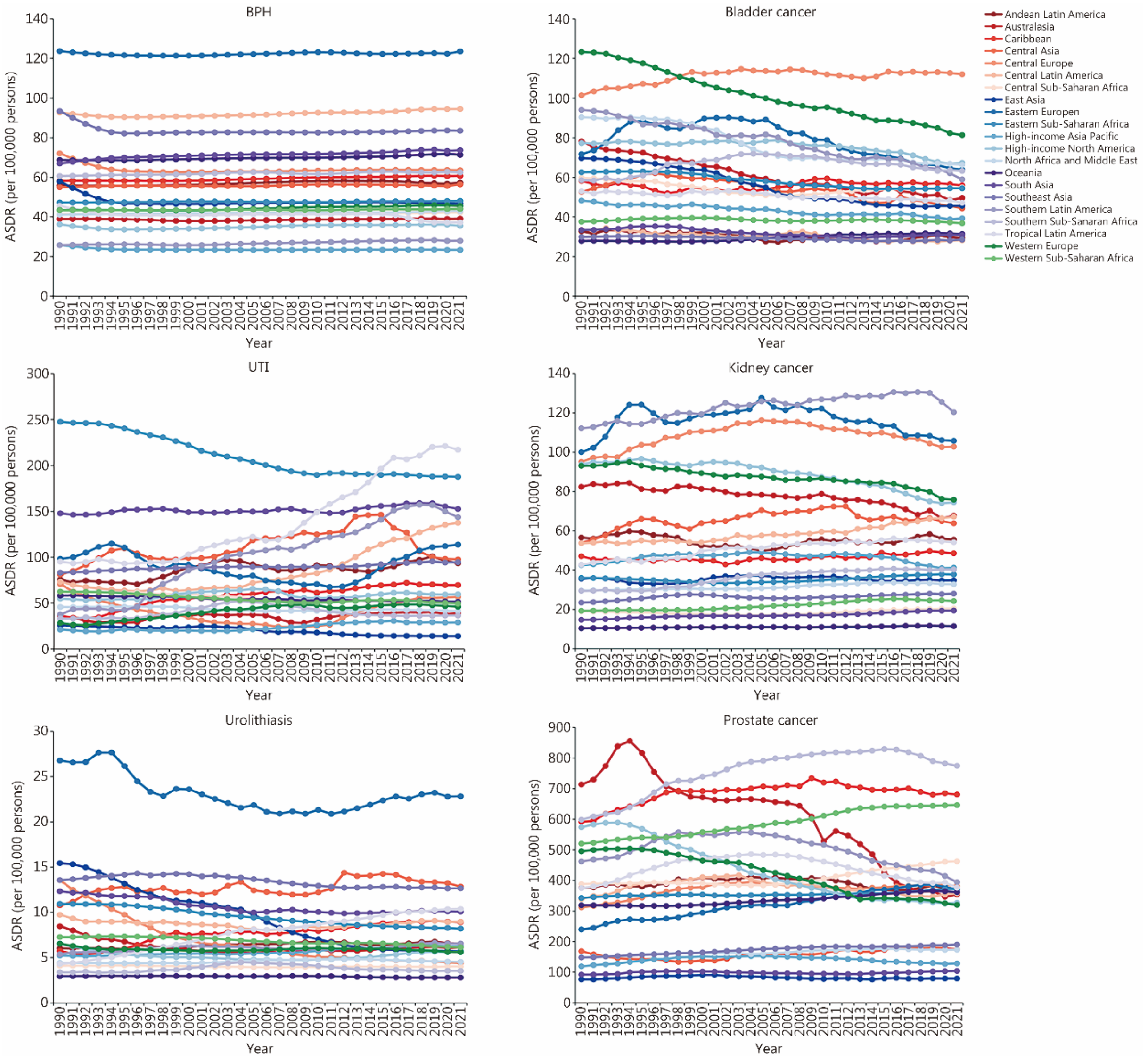

Fig. S7 The ASDR for 6 urologic diseases in 21 regions from 1990 to 2021. ASDR age-standardized DALYs rate, DALYs disability-adjusted life-years, BPH benign prostatic hyperplasia, UTI urinary tract infections

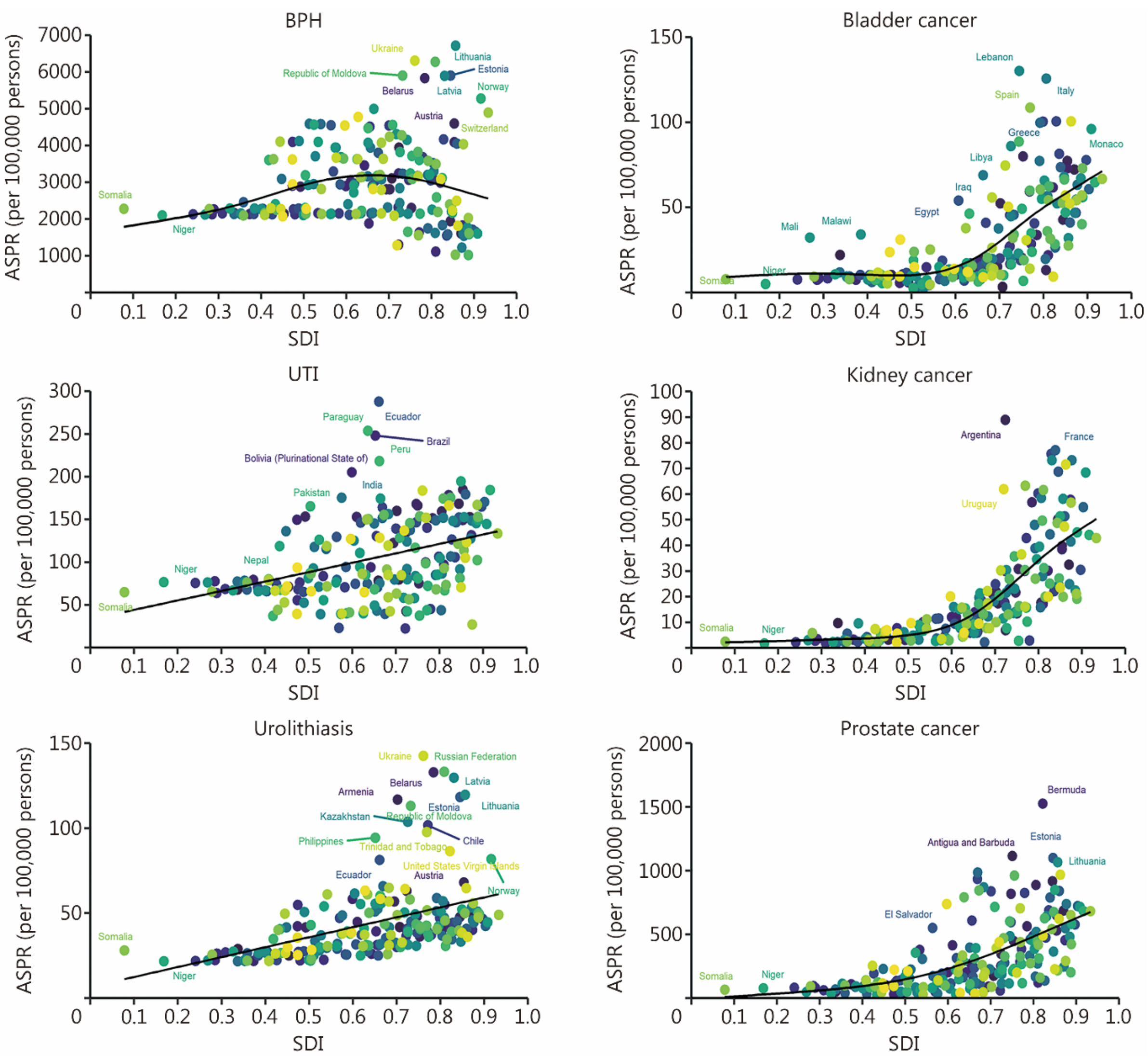

Fig. S8 ASPR of 6 urologic diseases for 204 countries and territories by SDI. ASPR age-standardized prevalence rate, SDI sociodemographic index, BPH benign prostatic hyperplasia, UTI urinary tract infections

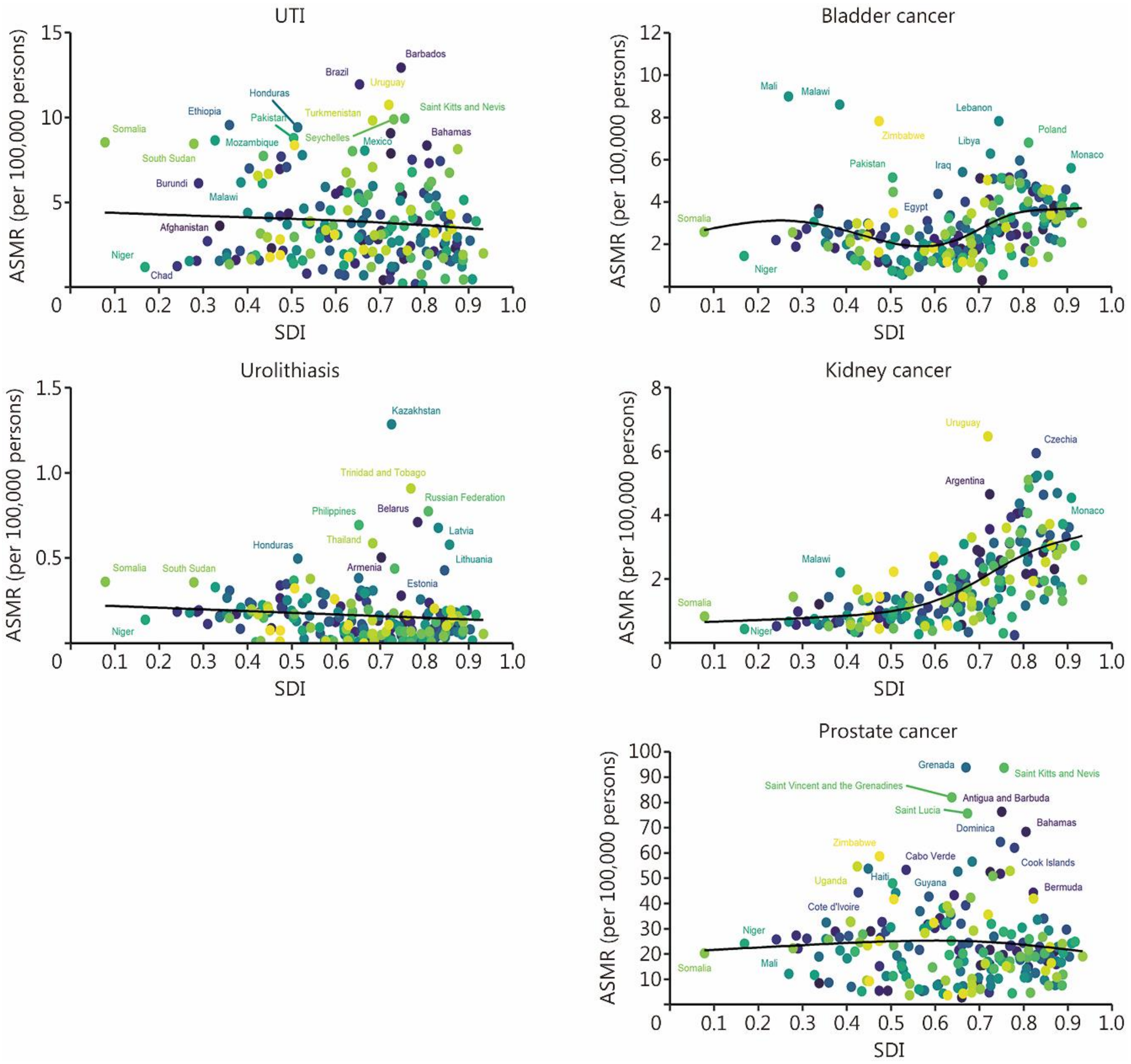

Fig. S9 ASMR of 5 urologic diseases for 204 countries and territories by SDI. ASMR age-standardized mortality rate, SDI sociodemographic index, UTI urinary tract infections

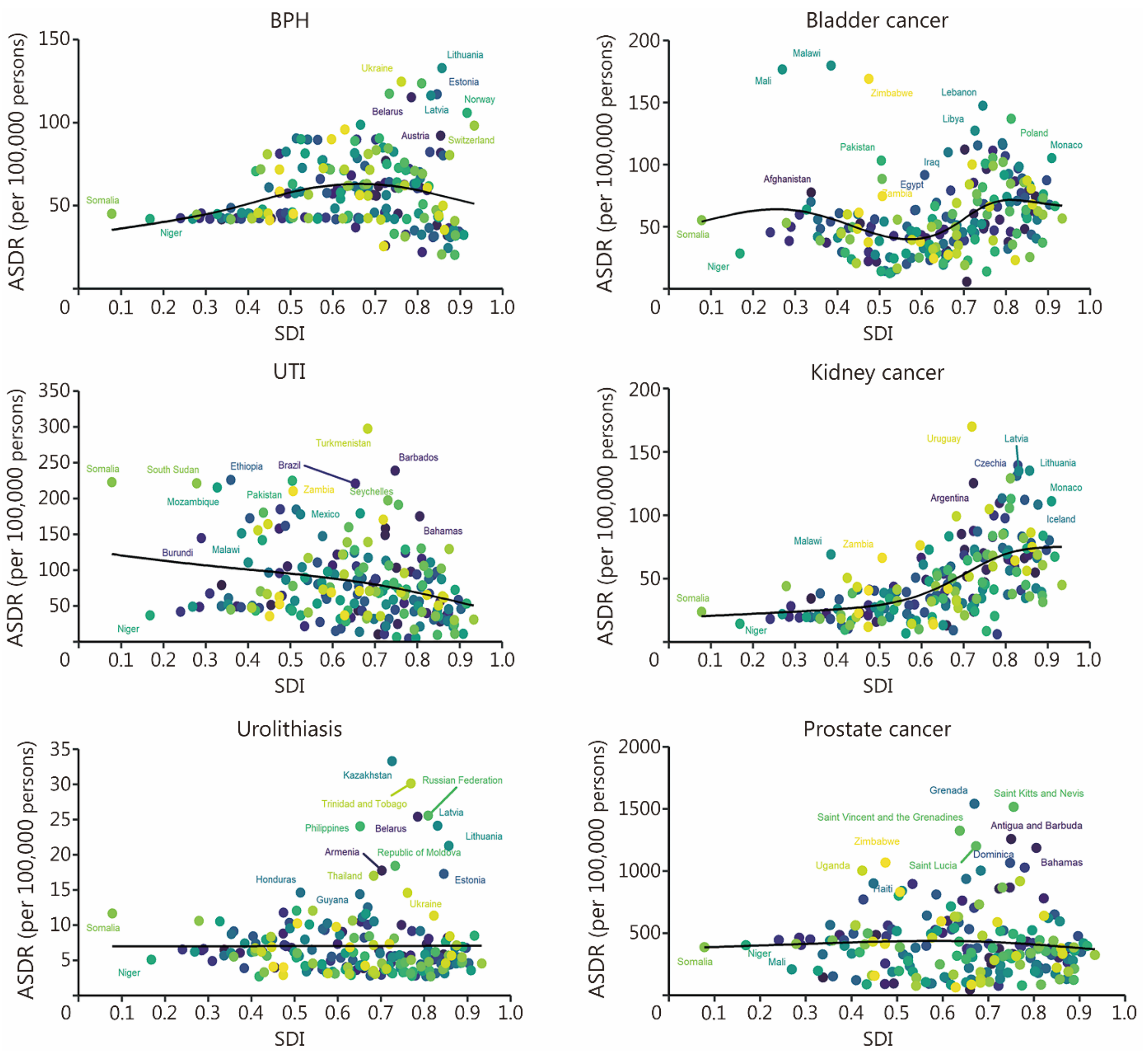

Fig. S10 ASDR of 6 urologic diseases for 204 countries and territories by SDI. ASDR age-standardized DALYs rate, DALYs disability-adjusted life-years, SDI sociodemographic index, BPH benign prostatic hyperplasia, UTI urinary tract infections

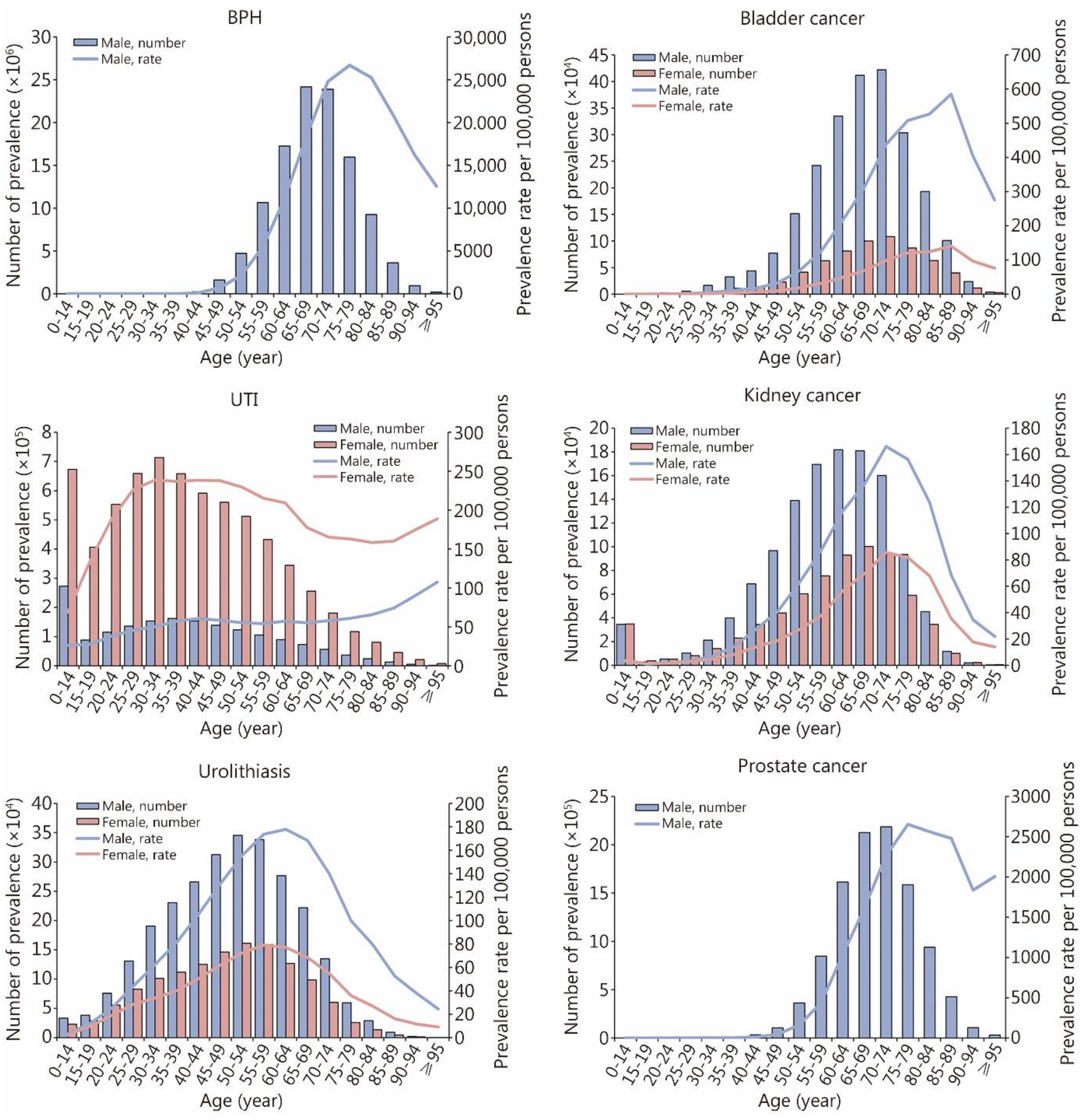

Fig. S11 Global prevalence of 6 urologic diseases by age and sex in 2021. BPH benign prostatic hyperplasia, UTI urinary tract infections

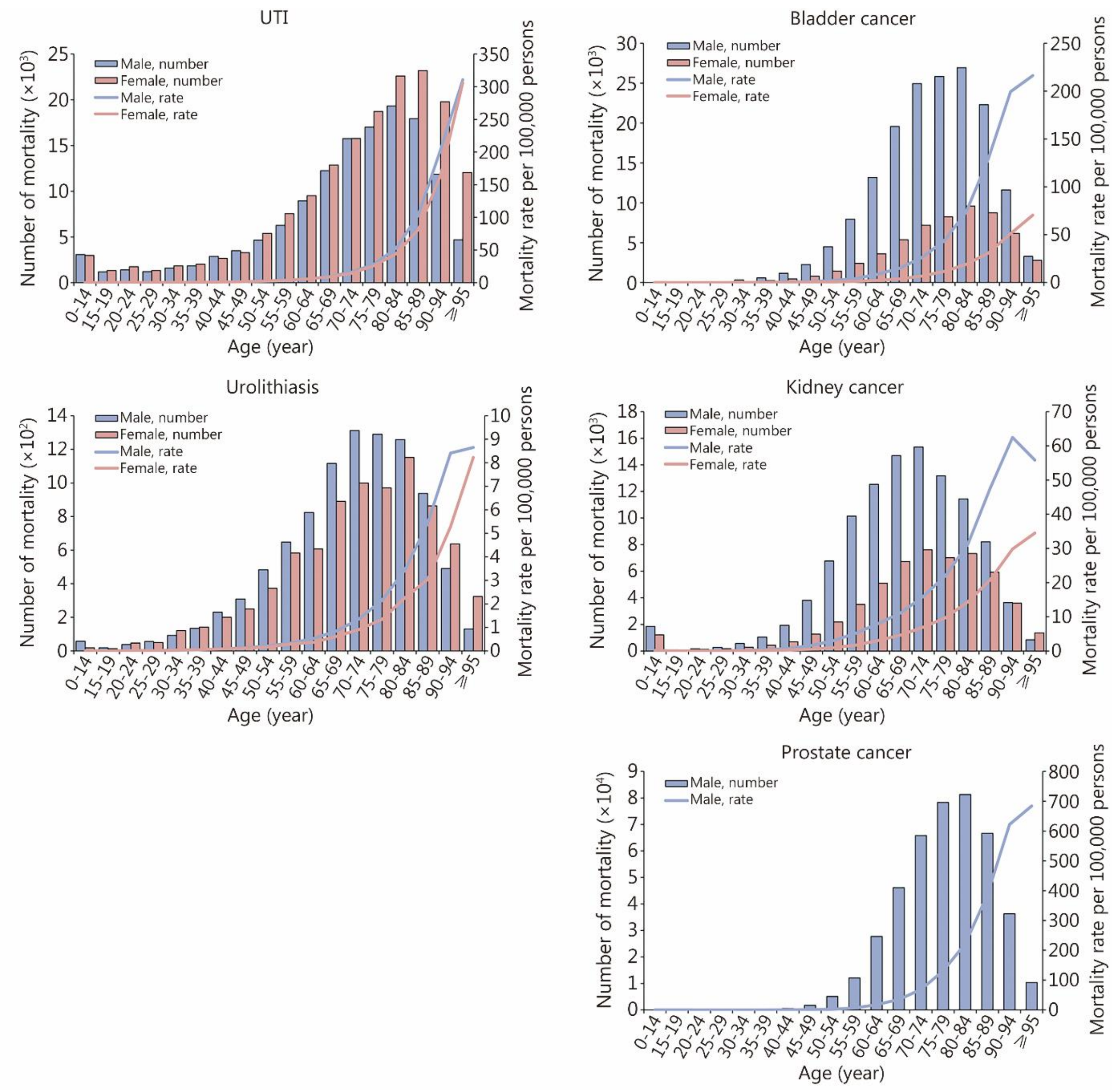

Fig. S12 Global mortality of 5 urologic diseases by age and sex in 2021. UTI urinary tract infections

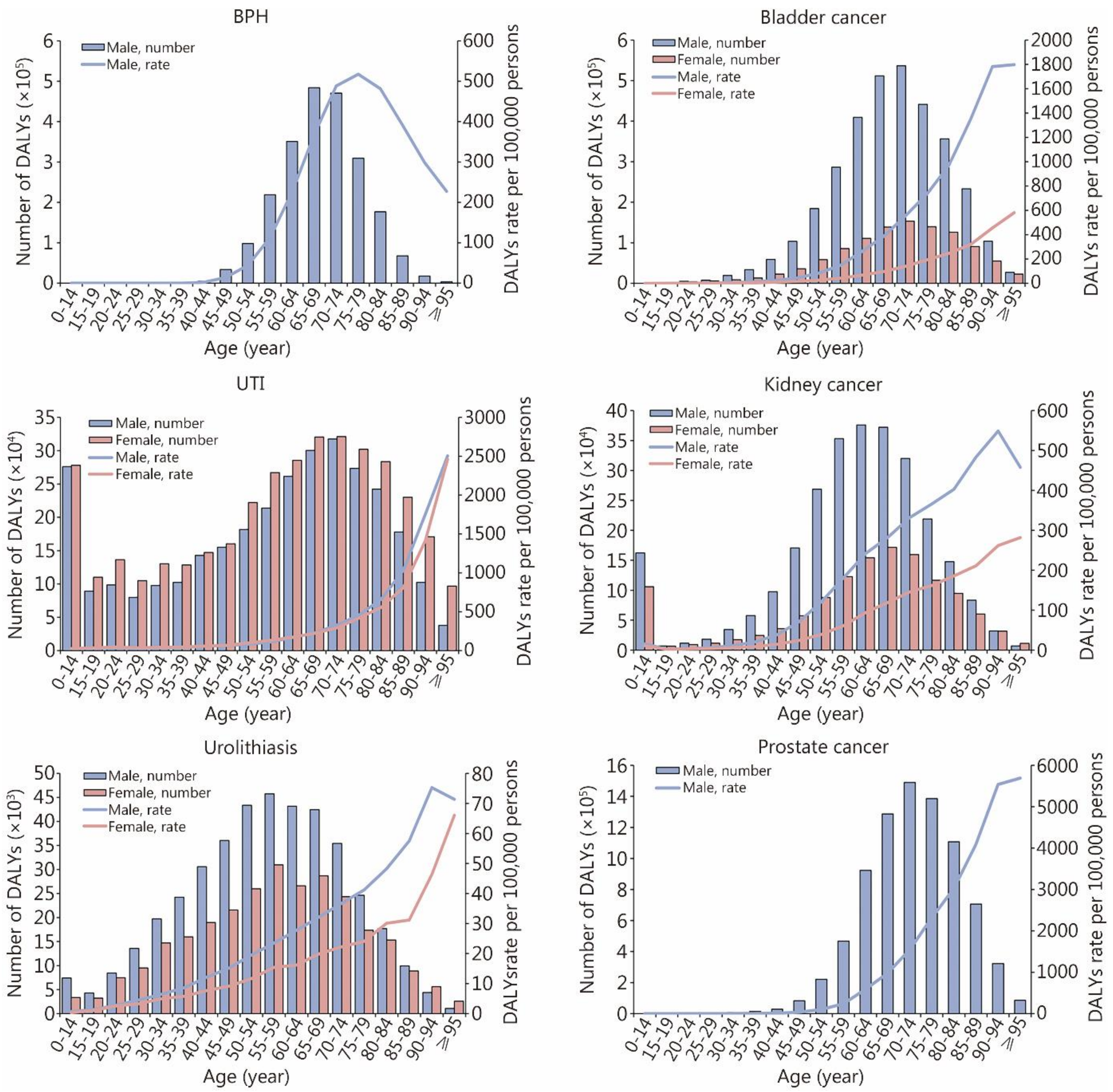

Fig. S13 Global DALYs of 6 urologic diseases by age and sex in 2021. DALYs disability-adjusted life-years, BPH benign prostatic hyperplasia, UTI urinary tract infections
